# Ancient DNA reveals pervasive directional selection across West Eurasia

**DOI:** 10.1038/s41586-026-10358-1

**Published:** 2026-04-15

**Authors:** Ali Akbari, Annabel Perry, Alison R. Barton, Mohammadreza Kariminejad, Steven Gazal, Zheng Li, Yating Zeng, Alissa Mittnik, Nick Patterson, Matthew Mah, Xiang Zhou, Alkes L. Price, Eric S. Lander, Ron Pinhasi, Nadin Rohland, Swapan Mallick, David Reich

**Affiliations:** 1 Department of Genetics, Harvard Medical School, Boston, MA, USA; 2 Department of Human Evolutionary Biology, Harvard University, Cambridge, MA, USA; 3 Broad Institute of MIT and Harvard, Cambridge, MA, USA; 4 Shamsipour Technical and Vocational College, Tehran, Iran; 5 Department of Population and Public Health Sciences, Keck School of Medicine, University of Southern California, Los Angeles, CA, USA; 6 Center for Genetic Epidemiology, Keck School of Medicine, University of Southern California, Los Angeles, CA, USA; 7 Department of Quantitative and Computational Biology, University of Southern California, Los Angeles, CA, USA; 8 Department of Biostatistics, School of Public Health, University of Michigan, Ann Arbor, MI, USA; 9 Department of Biostatistics and Data Science, School of Public Health, The University of Texas Health Science Center at Houston, Houston, TX, USA; 10 Department of Archaeogenetics, Max Planck Institute for Evolutionary Anthropology, 04103 Leipzig, Germany; 11 Howard Hughes Medical Institute, Harvard Medical School, Boston, MA, USA; 12 Department of Statistics and Data Science, Yale University, New Haven, CT, USA; 13 Department of Epidemiology, Harvard T.H. Chan School of Public Health, Boston, MA, USA; 14 Department of Biostatistics, Harvard T.H. Chan School of Public Health, Boston, MA, USA; 15 Department of Systems Biology, Harvard Medical School, Boston, MA, USA; 16 Department of Biology, Massachusetts Institute of Technology (MIT), Cambridge, MA, USA; 17 Department of Evolutionary Anthropology, University of Vienna, Vienna, Austria; 18 Human Evolution and Archaeological Sciences, University of Vienna, Vienna, Austria

## Abstract

Ancient DNA has transformed our understanding of population history^1^, but its potential to reveal as much about human evolutionary biology has not been realized because of limited sample sizes and the difficulty of distinguishing sustained rises in allele frequency increasing fitness—directional selection—from shifts due to migrations, population structure, or non-adaptive purifying or stabilizing selection^2–7^. We present a method for detecting directional selection in ancient DNA time-series data that tests for consistent trends in allele frequency change over time, and apply it to 15836 West Eurasians (10016 with new data). Previous work showed that classic hard sweeps driving advantageous mutations to fixation have been rare over the broad span of human evolution^8,9^. In contrast, in the last ten millennia, we find that many hundreds of alleles have been affected by strong directional selection. We also document one standard deviation changes on the scale of modern variation in combinations of alleles that today predict complex traits. This includes decreases in predicted body fat and schizophrenia, and increases in measures of cognitive performance. These effects were measured in industrialized societies, and it remains unclear how these relate to phenotypes that were adaptive in the past. We estimate selection coefficients at 9.7 million variants, enabling study of how Darwinian forces couple to allelic effects and shape the genetic architecture of complex traits.

Ancient DNA data hold extraordinary promise for revealing adaptation, making it possible to track effects across time and obtain direct measurements of selection coefficients^10,11^. Rather than being trapped in the present and studying the scars left by selection on the genomes of descendants^12,13^, ancient DNA makes it possible to test directly if frequencies of variants shifted more than could be expected by chance^2–5^. Such data also make it easier to measure selection on variants not of recent mutational origin (standing variation), which is challenging to detect using retrospective methods^14^. Previous ancient DNA selection studies in West Eurasia^2–5^ (Europe and its neighbors in the Near East) have identified dozens of alleles influenced by selection, but despite growth in reported ancient individuals to more than 10,000 today^15^, the number of genome-wide significant loci reported in a single study grew only from 12 in the first genome scan in 2015^2^, to 21 in 2024^4^, raising the concern that this approach might not deliver broad insights into human adaptation. Here we increase the yield of discoveries by twenty-fold by adding more statistical power (due to a qualitatively new method and larger sample size), and reducing artifactual signals (through intensive data cleaning).

First, we increased power by testing for a consistent trend in allele-frequency change over time. Several past studies dealt with the challenge posed by population mixture by treating more recent populations as linear combinations of more ancient ones, then searching for alleles whose frequencies were outliers compared to what would be expected from this history^2,3^. However, changes in frequency due to selection are often less than what can be expected from gene flow and random genetic drift^6^, and in this context, increasing sample size helps little. We employed a qualitatively different approach, using the genetic similarity of each individual to every other, and testing if the date when they lived provides additional predictive power for the allele frequencies of their population beyond what is expected from the empirical population structure. Our test is simple: at each variant we ask if hypothesizing a non-zero selection coefficient *s*—causing allele frequency to trend in the same direction over all times and places—predicts frequency differences across populations significantly better than empirically measured population structure alone.

Second, we increased power through a 14-fold increase in sample size, driven by 10016 ancient individuals for which we report new data, which combined with previously reported data yields a dataset of 15836 people spanning 18000 years (Supplementary Tables 1–3). We co-analyzed with 6438 modern people, sub-sampled so their countries of origin were spread evenly over West Eurasia (Extended Data Figures 1a,b).

Third, we increased power by imputing diploid genotypes^16^, leveraging known patterns of allelic correlation in a modern reference panel to fill in missing data in all individuals. We also carried out intensive data cleaning (Supplementary Information section 1), filtering out SNPs susceptible to false-positives. The final dataset included 8,074,573 single nucleotide polymorphisms (SNPs) and 1,665,051 insertions/deletions (indels) on chromosomes 1-22 .

## Test of selection on single variants

**Extended Data Figure 1:**
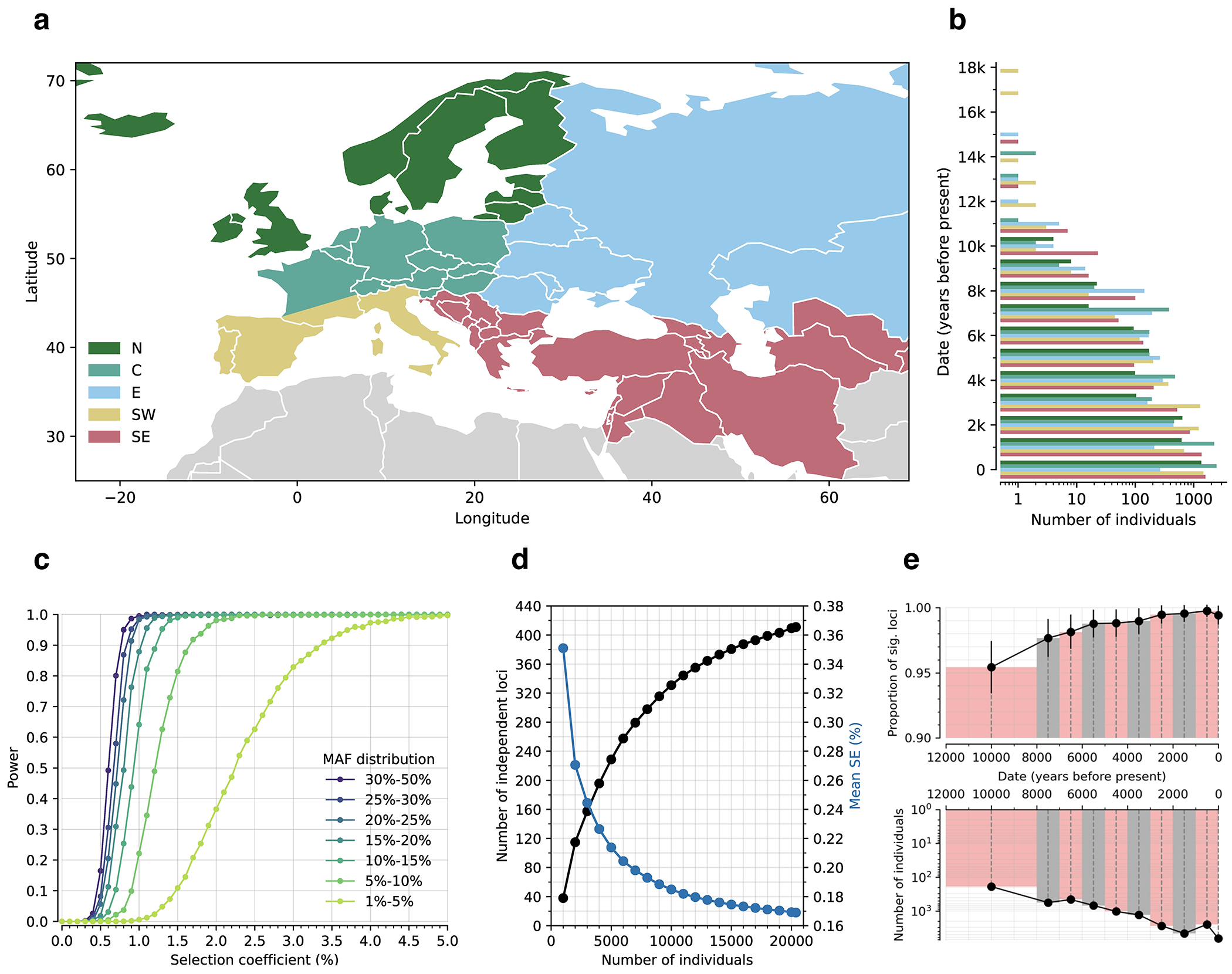
Spatiotemporal distribution of individuals and effect on power. **(a)** Geographic origin: North (N), Central (C), East (E), Southwest (SW) and Southeast (SE). **(b)** Temporal distribution (x-axis on a logarithmic scale). **(c)** Power analysis based on simulations. Sample size, dates, and pattern of genetic relatedness are matched to real data. Power is defined as proportion of true positives expected at P<5x10^−8^, based on two sided P values. We ran 20000 simulations for each selection coefficient, with minor allele frequency (MAF) at present (time=0) randomly drawn from the MAF distribution in modern Europeans. **(d)** Number of independent and significant loci and standard error in selection coefficients as a function of sample size (from downsampling). **(e)** Effect of age on power. Data are divided into 10 non-overlapping periods; modern individuals are a separate bin. Top panel: proportion of 479 loci remaining significant after excluding 100 random individuals from each bin; error bars indicate 95% confidence intervals. Bottom panel: number of individuals per bin.

**Extended Data Figure 2:**
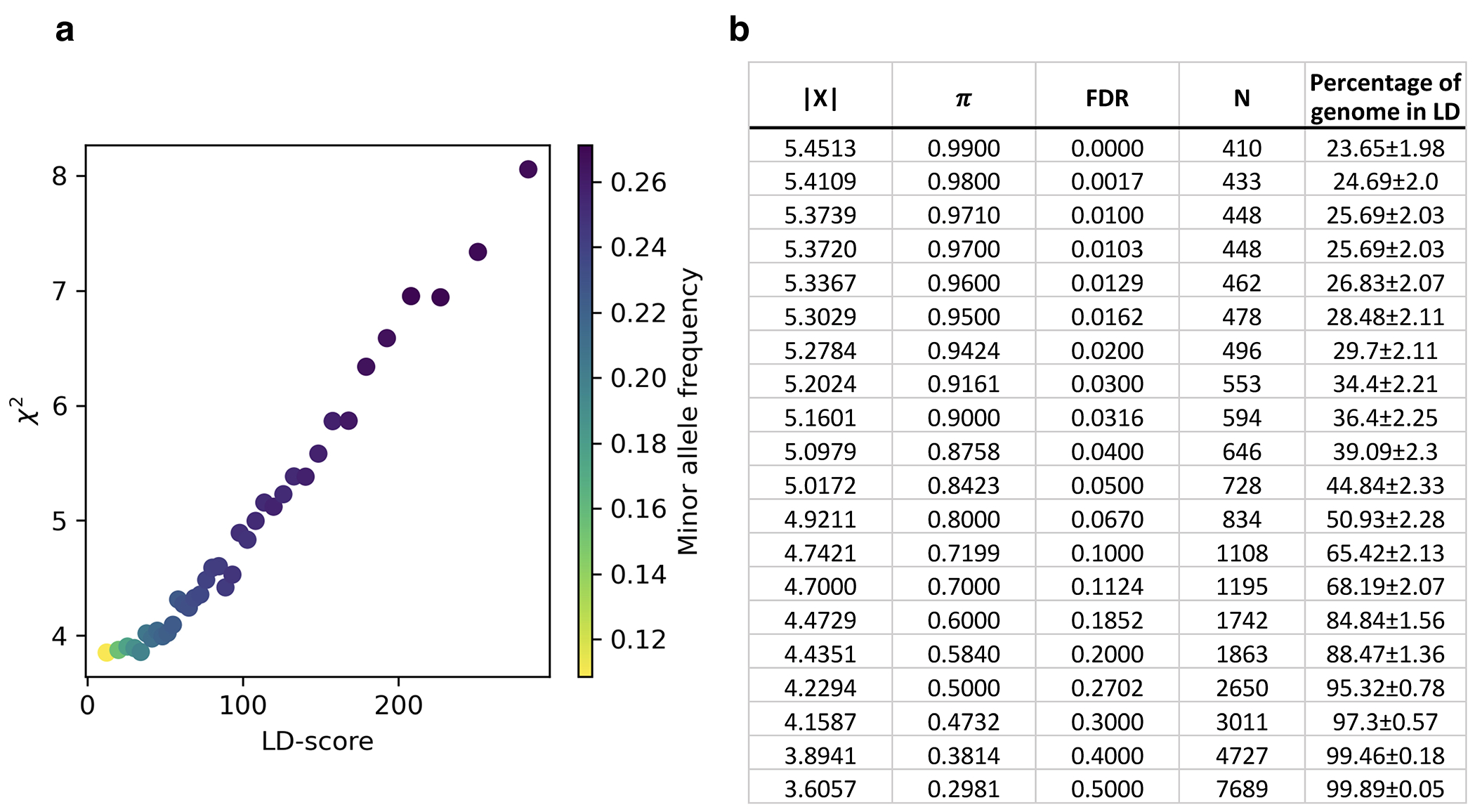
High proportion of genome affected by directional selection. **(a)** LD score plot for nominal χ^2^ statistics, with each point representing an LD score quantile. Values are averaged across each bin. **(b)** Mapping X-score to posterior probability (π), False Discovery Rate (FDR), number of independent loci excluding the HLA region (N), and the fraction of the genome (in base pairs) covered by stretches of LD (r^2^ > 0.05) around tag SNPs representing these loci. The stretches of LD are calculated in the modern genome using European populations from the 1000 Genomes Project.

For each SNP in the genome, we estimate a selection coefficient, which we found has a standard error of typically 0.17% for common variants (Extended Data Figures 1c,d). In theory, a valid test for selection should be *Z*, the number of standard errors this quantity is from zero, and we should be able to use a normal distribution to identify scores that pass the standard threshold of genome-wide significance (P<5x10^−8^). In practice, the median χ^2^ statistic (squared *Z*-score for number of standard errors *s* is from zero) is inflated by 4.75-fold relative to a χ^2^ distribution with one degree of freedom. In human genetics studies, such inflation can arise due to uncorrected population structure, or non-normality of the null distribution due to case/control sample imbalance^17^. Inflation like this is often addressed by rescaling χ^2^ statistics by the median inflation across the genome^18,19^. But if the null is not normally distributed, or a substantial fraction of the genome carries real signal^18^, random sites will not provide an appropriate neutral baseline. These concerns apply here: the null distribution is not normal due to frequency-dependent biases from imputation and the non-linear transformation of allele frequencies (Supplementary Information section 2), and most of the genome is in linkage disequilibrium (LD) with sites showing evidence of directional selection (Supplementary Information section 3, Extended Data Figure 2b).

**Extended Data Figure 3:**
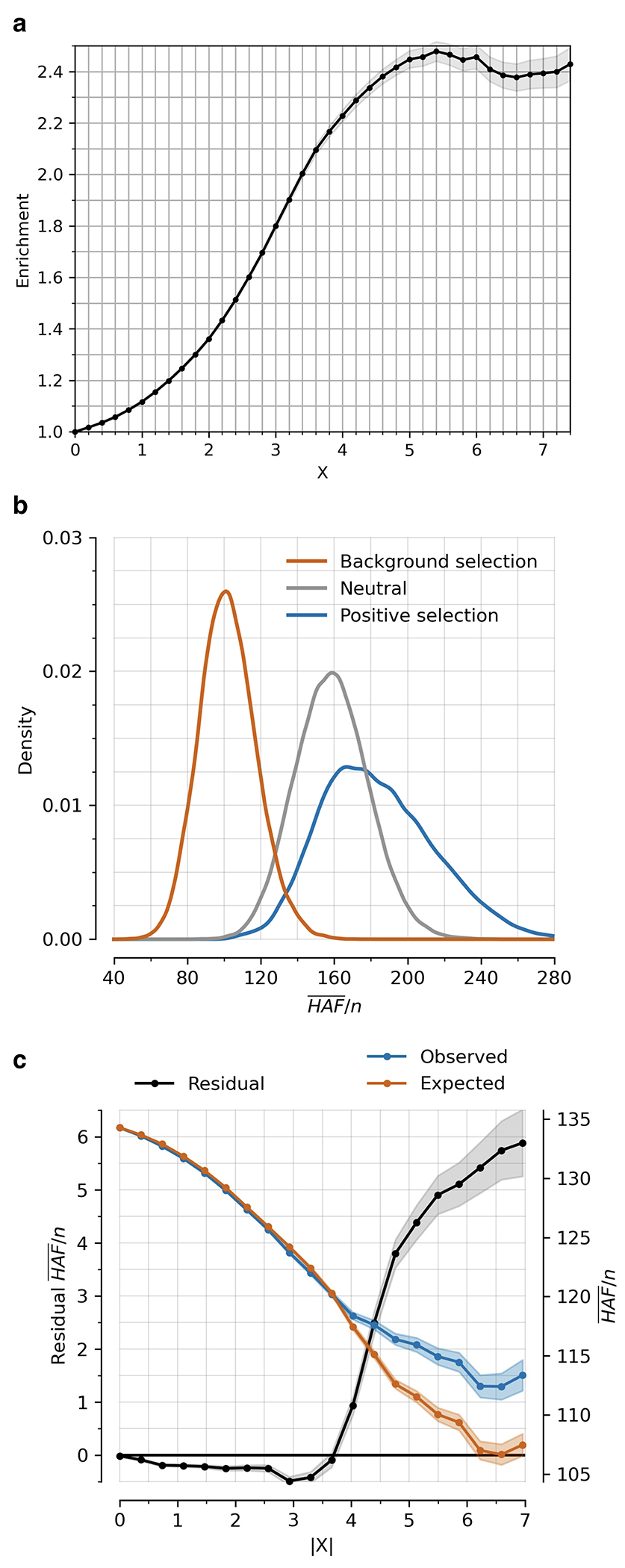
Robustness of directional selection signals (related to Figures 1a,c). **(a)** Enrichment of SNPs significant in any of 452 UK Biobank GWAS studies for X-statistics with magnitudes larger than the threshold on the x-axis, adjusted for minor allele frequency and measures of background selection (McVicker-B, Murphy-phastCons, and Murphy-CADD). Background selection tends to be higher in functional genomic regions, so SNPs with higher |X| are more penalized than in Figure 1a hence the lower plateau. **(b)** Simulating neutral, background, and positive selection for a 200 kb window around a focal SNP, with derived allele frequency drawn uniformly from [0,1]. Under positive selection, the focal SNP has a selection coefficient of s = 0.01 for the favored allele. Under background selection, 20% of mutations are drawn from a gamma distribution with mean −0.03 and shape parameter 0.206, and the remainder are neutral. The population size is constant at 20000 diploid individuals, mutation rate per base pair per generation is 2x10^−8^, and recombination rate is 1 cM per 1 Mb. **(c)** Residual mean (HAF)/n for a haploid sample size n over 200 bp windows is observed minus expected value. Expected value is determined using a linear regression model with explanatory variables McVicker-B, Murphy-phastCons, Murphy-CADD, number of SNPs, and total heterozygosity, providing the expected mean (HAF)/n conditioned on them. Shaded areas show 95% confidence intervals.

**Figure 1:**
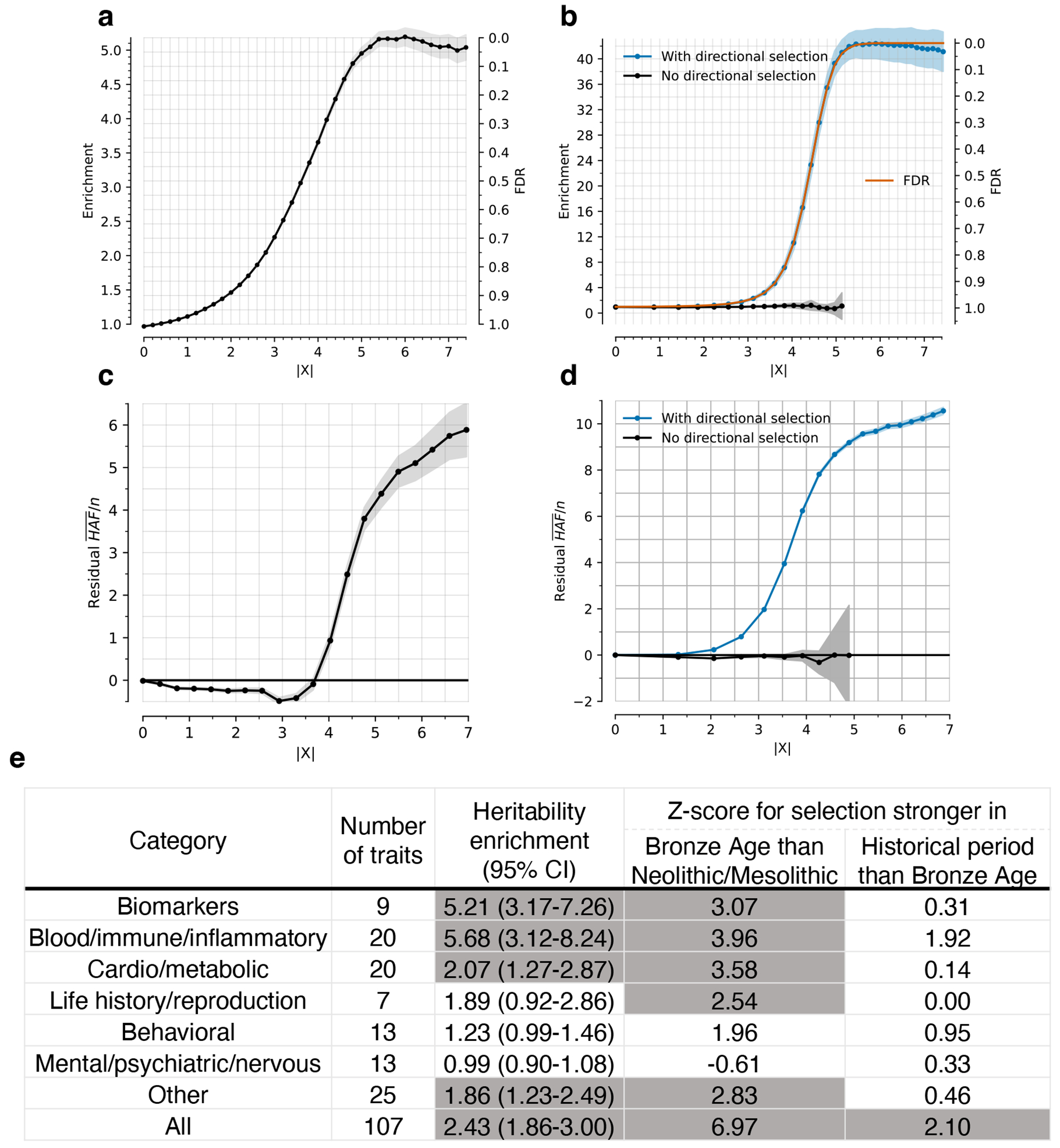
Multiple lines of evidence show we are detecting genuine directional selection. **(a)** Enrichment of SNPs significant in at least one of 452 UK Biobank GWAS, for SNPs with |X| above the value on the x-axis, controlling for allele frequency. (**b**) Simulations reveal enrichment of GWAS hits (blue, left axis) closely aligns with 1-FDR (black, right axis) until a plateau as a function of |X|: 800 simulations with directional selection (200 each for selection coefficients of 0.01, 0.02, or 0.03 for Model 2.1, soft sweep; and 0.05 for Model 2.2, hard sweep), and 800 simulations without it. **(c)** Residual mean HAF-score [(HAF)/n], for SNPs with |X| above the value on the x-axis, computed as observed minus expected, with n haploid sample size, from a linear regression correcting for background selection using as explanatory variables McVicker-B, Murphy-phastCons, Murphy-CADD, number of SNPs, and heterozygosity in a 200 kb window. (**d**) Residual mean HAF-score in simulation, for SNPs with |X| above the value on the x-axis, computed as observed minus expected, with n the haploid sample size, from a linear regression correcting for background selection using as explanatory variables McVicker-B, coding region annotation, functional noncoding region annotation, number of SNPs, and heterozygosity in a 200 kb window. **(e)** The heritability enrichment is a meta-analysis for annotations based on a binary selection annotation, with FDR either below 1% (=1) or above 1% (=0). Z-score for change in selection intensity over time is based on a meta-analysis of heritability enrichment comparing key cultural transitions: Mesolithic-Neolithic (MN) to Bronze Age (BA); and Bronze Age (BA) to Historical Era (HE). We annotate each SNP according to whether it is among the top 5% with the highest probability of a stronger magnitude of selection coefficient in one time transect vs. another. Shaded areas show 95% confidence intervals.

Instead, we calibrated our test by taking advantage of a finding about the connection between selection coefficients and associations to phenotypes in living people. The proportion of SNPs showing significant association to a phenotype in a Genome-Wide Association Study (GWAS) increases with our selection statistic, plateauing at around 5.2-times the rate for random SNPs (Figure 1a) (this analysis relies on 1,317,022 SNPs with a genome-wide significant association to at least one phenotype for 452 traits in the UK Biobank, and conditions on minor allele frequency (MAF) to remove bias due to signals being easier to detect for higher MAFs). The plateau occurs at the same place when we control for “background selection”, the loss of neutral variation linked to deleterious mutations removed by purifying selection, although the enrichment in GWAS variants is reduced (~2.4-times; Extended Data Figure 3a). This is the pattern expected for a true threshold for genome-wide significance: if SNPs beyond this threshold reflect a combination of true signal and false discoveries, we would expect enrichment to continue beyond it. This threshold occurs at Z=8.23, larger than the standard threshold (5.45) for genome-wide significance for a normal distribution, so we rescale the naïve score by this quantity to obtain an X-statistic (Z/1.51) whose significance threshold matches the standard threshold.

**Extended Data Figure 4:**
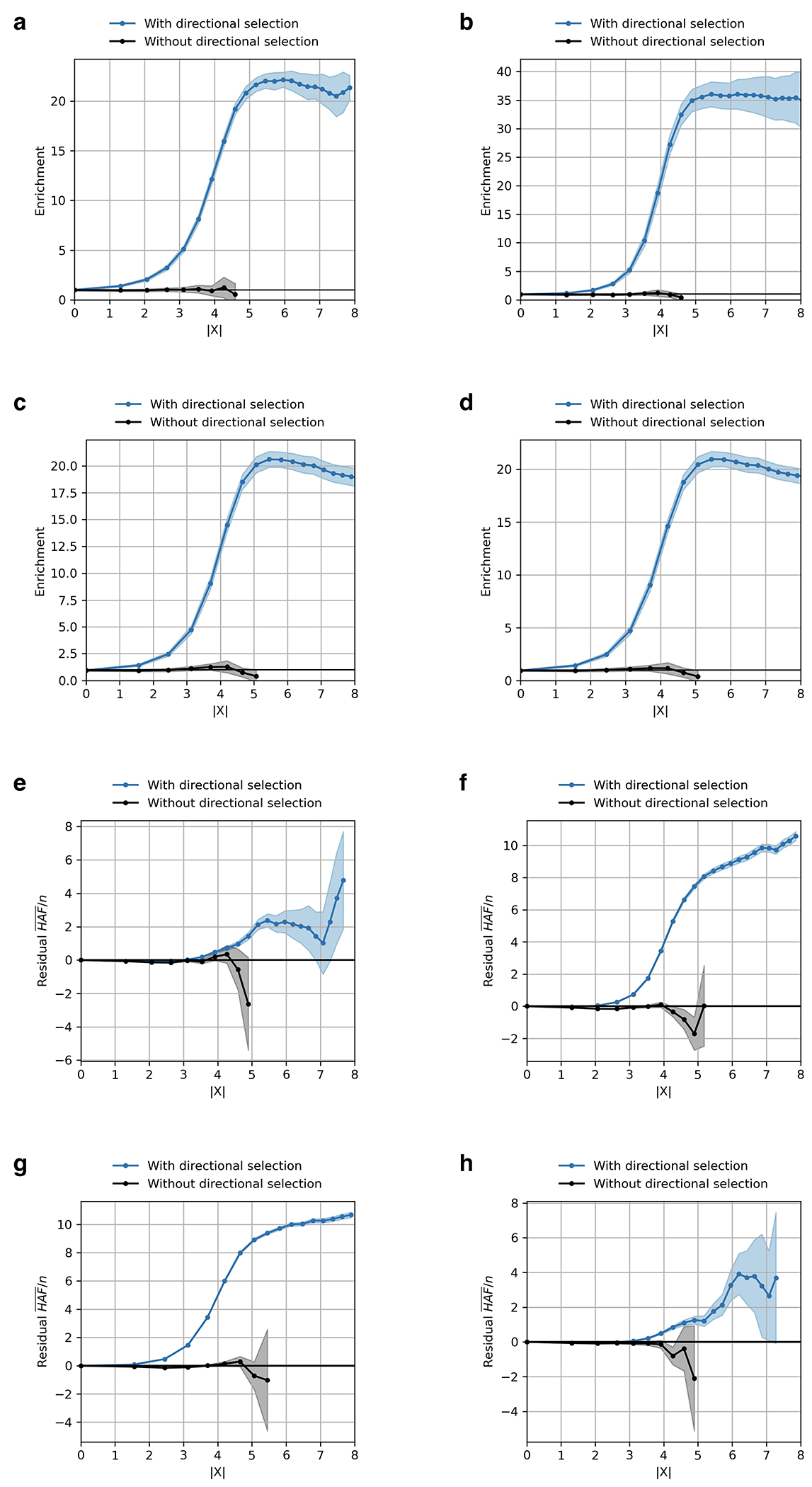
GWAS enrichment and residual HAF of selection signal under simulated models of selection. **(a-d)** GWAS enrichment by X-score across four simulated models: **(a)** Model 1, polygenic directional selection with a stabilizing selection mechanism; **(b)** Model 2.1, soft sweep with a purifying selection mechanism; **(c)** Model 2.2, hard sweep with a purifying selection mechanism; **(d)** Model 3, polygenic directional selection with both stabilizing and purifying selection mechanisms. Shaded areas show 95% confidence intervals around the estimated enrichment. Each experiment includes 800 simulations (400 with directional selection and 400 without); blue denotes all simulations, and black denotes those without directional selection. **(e-h)** Residual mean (HAF)/n, for SNPs with |X| above the value on the x-axis, for a haploid sample size n over 200 bp windows across four simulated models: **(e)** Model 1, polygenic directional selection with a stabilizing selection mechanism; **(f)** Model 2.1, soft sweep with a purifying selection mechanism; **(g)** Model 2.2, hard sweep with a purifying selection mechanism; **(h)** Model 3, polygenic directional selection with both stabilizing and purifying selection mechanisms. For each SNP, we compute mean (HAF)/n across haplotypes and residualize it as observed minus expected from a linear regression correcting for background selection using explanatory variables McVicker-B, coding region annotation, functional noncoding region annotation, number of SNPs, and total heterozygosity in a 200 kb window. Solid lines show the mean of this SNP-level residual statistic across SNPs meeting the |X| threshold; shaded areas show 95% confidence intervals. Each experiment includes 800 simulations (400 with directional selection and 400 without); blue denotes all simulations, and black denotes those without directional selection.

To validate our procedure, we conducted realistic forward-in-time simulations (Figure 1b, Extended Data Figure 4, Supplementary Information section 2). The simulations specify a demographic history inspired by that of Europe, and produce patterns of structure matching important features of real data. The simulations include coding, functional noncoding, and neutral regions, recapitulating varying degrees of background selection. The simulations also permit exploration of the relationship between selected alleles and traits, in particular the phenomenon of “stabilizing” selection to remove genetic variation when the average genetic predictor of a trait is at a fitness optimum. We verified that these simulations produce a deficiency of common polymorphisms influencing traits, matching patterns in real data^20^.

Our test for directional selection is robust to every confounding factor we considered, including population structure, background selection due to purifying or stabilizing selection, sampling bias, and selection in the ancestral population but not during the time-transect itself (Supplementary Information section 2). None of these produces even a hint of the enrichment in GWAS signals we observe in data. In contrast, in simulations with directional selection, including where we shift the trait optimum leading to deterministic changes in allele frequency to bring the population closer to that optimum^21^, we observe exactly this signal and can use it to provide a valid calibration of genome-wide significance (Figure 1b, Extended Data Figure 4 , Supplementary Information section 2). To translate X to a posterior probability of selection *π*, we use a False Discovery Rate (FDR) approach (Figures 1a, 1b, and Supplementary Information section 3). We fit a smooth function to the enrichment curve for GWAS signals and estimate that at X greater than our threshold of 5.45, *π*>99%.

A second line of evidence that these signals are real comes from computing the mean “Haplotype Allele Frequency” (HAF) score, the sum of squared derived allele frequencies scaled by sample size in 200-kilobase windows surrounding each tested variant. Previous work^22,23^ showed that directional positive selection on derived alleles is expected to increase HAF scores, while background selection is expected to decrease it (Extended Data Figure 3b, Supplementary Information section 4). After computing the residual HAF score for each variant controlling for background selection^24,25^, we find it increases with the X-statistic and begins to rise before 5.45, the standard threshold for genome-wide significance in GWAS (Figure 1c, Extended Data Figure 3c). Our forward-in-time simulations (Supplementary Information section 2) confirm that the rise in HAF score occurs only for directional selection, with no hint of this pattern for other scenarios (Figure 1d, Extended Data Figure 4).

**Extended Data Figure 5:**
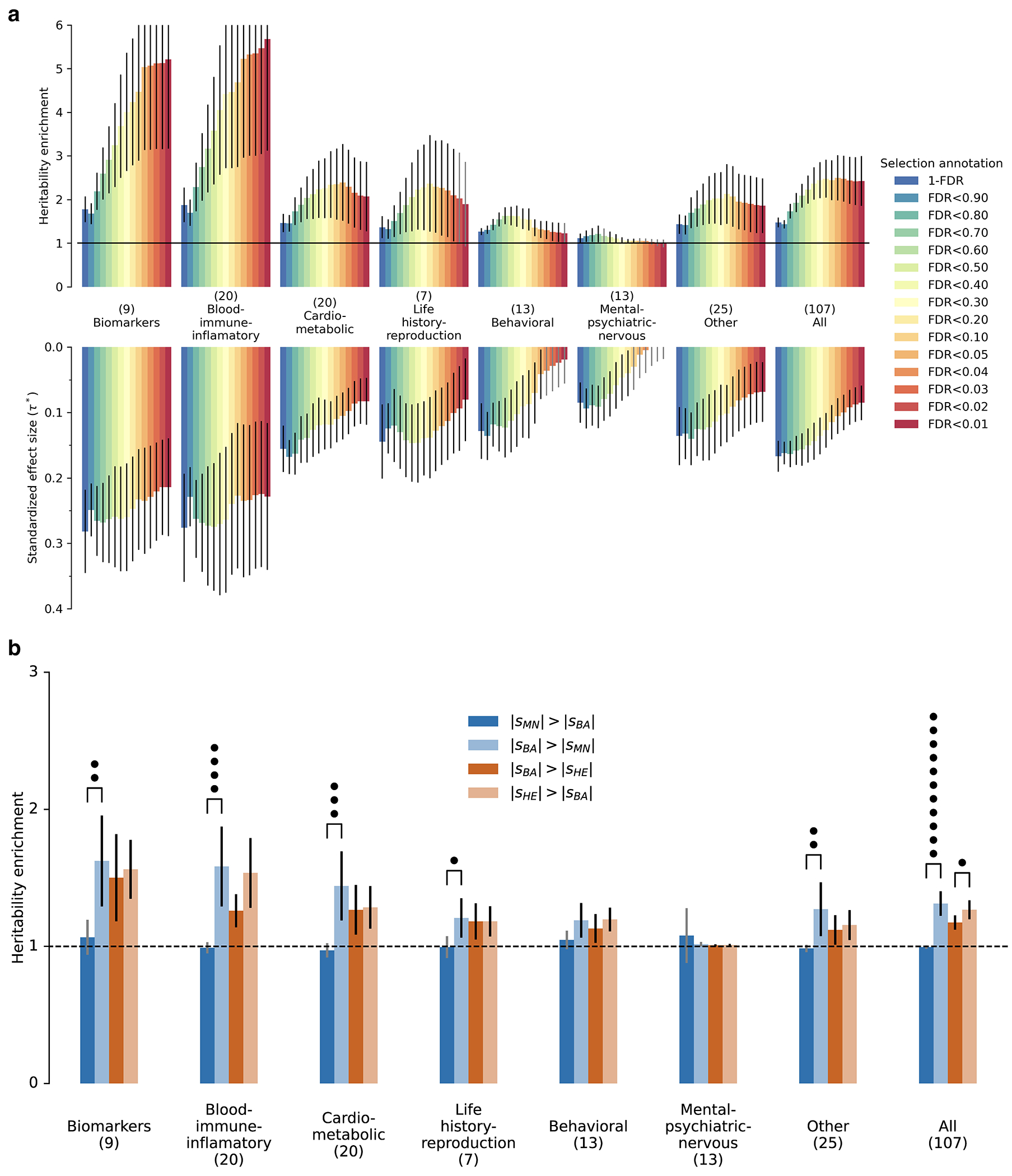
Stratified LD Score Regression shows that alleles affecting blood-immune-inflammatory and cardio-metabolic traits were unusually affected by selection, and that selection intensity increased in the Bronze Age (related to Figure 1e). **(a)** We annotated sites based on their inferred strength of selection—based on their FDR being above a specified threshold, or 1-FDR as a continuous annotation—and used Stratified LD Score Regression (S-LDSC) to study enrichment of GWAS signals and standardized effect sizes (τ*) for traits in different functional categories. Our analysis adjusts for 97 annotations that are known to affect heritability and are part of the standard correction in S-LDSC **(b)** Tests for changes in selection intensity during different cultural transitions: Mesolithic-Neolithic (MN) to Bronze Age (BA); and Bronze Age (BA) to Historical Era (HE). Each annotation is binary, identifying SNPs among the top 5% with the highest probability of experiencing stronger selection during one time period compared to another. This is determined using the estimated selection coefficient and standard error from models separately fit to each cultural period. Error bars show 95% confidence intervals of the estimated standardized effect sizes (τ*) or heritability enrichment; dots represent significance of the pairwise comparisons of heritability enrichment.

We obtained a third line of evidence that we are detecting real signals of adaptation by showing that |X|>5.45 variants are associated with some classes of traits much more than others. We find enrichment for SNPs contributing to blood-immune-inflammatory traits (95% confidence interval (CI) 3.12-8.24)^3,5^, compared to random SNPs with matched characteristics defining the baseline. For mental-psychiatric-nervous and behavioral traits, we do not detect enrichment (95% CI of 0.90-1.08 and 0.99-1.46) (Figure 1e, Extended Data Figure 5a). This cannot be explained by differences in allele frequencies or background selection since we control for these factors. The intensity of selection on blood-immune-inflammatory and cardio-metabolic traits both increased significantly in the Bronze Age relative to the pre-farming period (Figure 1e, Extended Data Figure 5b), plausibly reflect adaptation to new diets, larger population, or living closer to domesticated animals^26^.

## Hundreds of cases of directional selection

**Figure 2:**
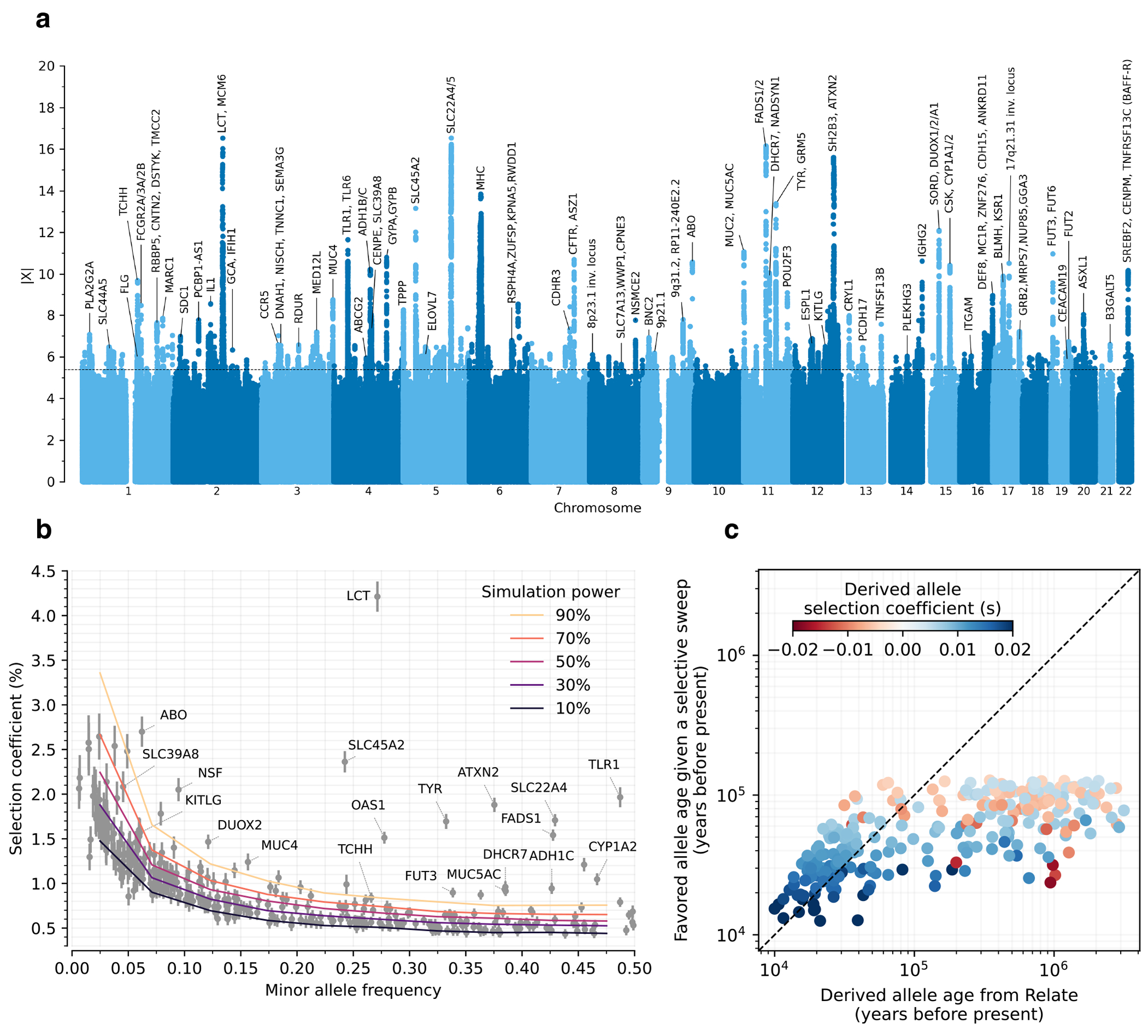
Genome scan for directional selection. **(a)** The x-axis is chromosomal position, and the y-axis the selection signal for each variant. The dotted line indicates our genome-wide significance threshold of |X|=5.45. For clarity, only a subset of loci are annotated. **(b)** Selection coefficient (*s*) estimated from our scan plotted against minor allele frequency of tagging SNPs at independent loci with FDR<5%. Overlaid grids are simulation-based power estimates (90%, 70%, 50%, 30%, and 10% probability of detection). Error bars indicate standard errors from 20,374 unrelated individuals. **(c)** The estimated age of the favored allele in a selective sweep versus the date of origin of the mutation inferred from RELATE^58^, for tagging SNPs with FDR<5% at independent loci. The age of the sweep is defined as the time in the past when the frequency of the favored allele is expected to have been 0.0001 given the present-day frequency in 1000 Genomes Project European populations and assuming the selection coefficient has been constant over time.

We found evidence of 479 independent loci (410 excluding the HLA region) with |X|>5.45, corresponding to a *π*>99% probability of selection (Figure 2a). To produce this list, we identified the strongest signal in the genome and considered all SNPs in LD with it in unrelated Western-European individuals (r^2^>0.05) to potentially reflect the same signal. We then found the second-strongest signal excluding these positions, and so on until no more variants pass this threshold (Extended Data Figure 2b). Visualizations of the trajectories for these loci (Supplementary Information section 5), and summary statistics for 9.7 million variants (Supplementary Table 4), can be cross-referenced with GWAS and with their frequency trajectories at the AGES browser https://reich-ages.rc.hms.harvard.edu.

The actual number of loci under selection is likely to be much larger. Using a threshold of |X|>3.61 (FDR=50%), we identify 7689 non-HLA loci, implying more than 3800 independent episodes of selection. The exact number of distinct signals is difficult to count: residual LD exists among candidate loci which at some loci may cause some overestimation of signals (Supplementary Information section 5), while truly selected alleles in LD with nearby stronger ones will be undercounted. Down-sampling analyses show that further increases in sample size are expected to increase the number of detected loci further, with people living >8000 years ago providing the most added power (Extended Data Figure 1d,e).

To obtain insight into the phenotypes shaped by directional selection, we take advantage of the fact that a high proportion (62%) of the variants with genome-wide evidence of selection are independently associated to a phenotype in at least one UK Biobank GWAS. However, biological interpretation is complicated since the allele that was the target of selection may differ from the tag SNP we are using to represent the locus (and may even be in a neighboring gene); because some alleles affect multiple phenotypes; because the relevant modern trait may not be measured in one of the GWAS we are analyzing; or because the phenotype in modern societies may not have existed in the ancient ones where selection acted. The median selection magnitude |*s*| at the tag SNPs is 0.86% (range 0.43-4.3%), and the median minor allele frequency (MAF) is 13%. Standard errors in our estimates of |*s*| for common alleles are 0.17% (Figure 2b and Extended Data Figure 1c).

We compared our results to those of five previous scans for selection in Holocene West Eurasia (four based on ancient DNA)^2–5,13^ (Extended Data Table 1 and Supplementary Information section 6). Of 38 unique non-HLA loci that met the formal threshold for genome-wide significance in at least one previous study, 18 pass our *π*>0.99 threshold. The other 20 do not replicate, in most cases due to what appears to be incompletely controlled population structure driven by mixtures of populations with different allele frequencies before they came together and in a few cases due to failing quality control or fluctuating selection in space or time.

**Extended Data Figure 6:**
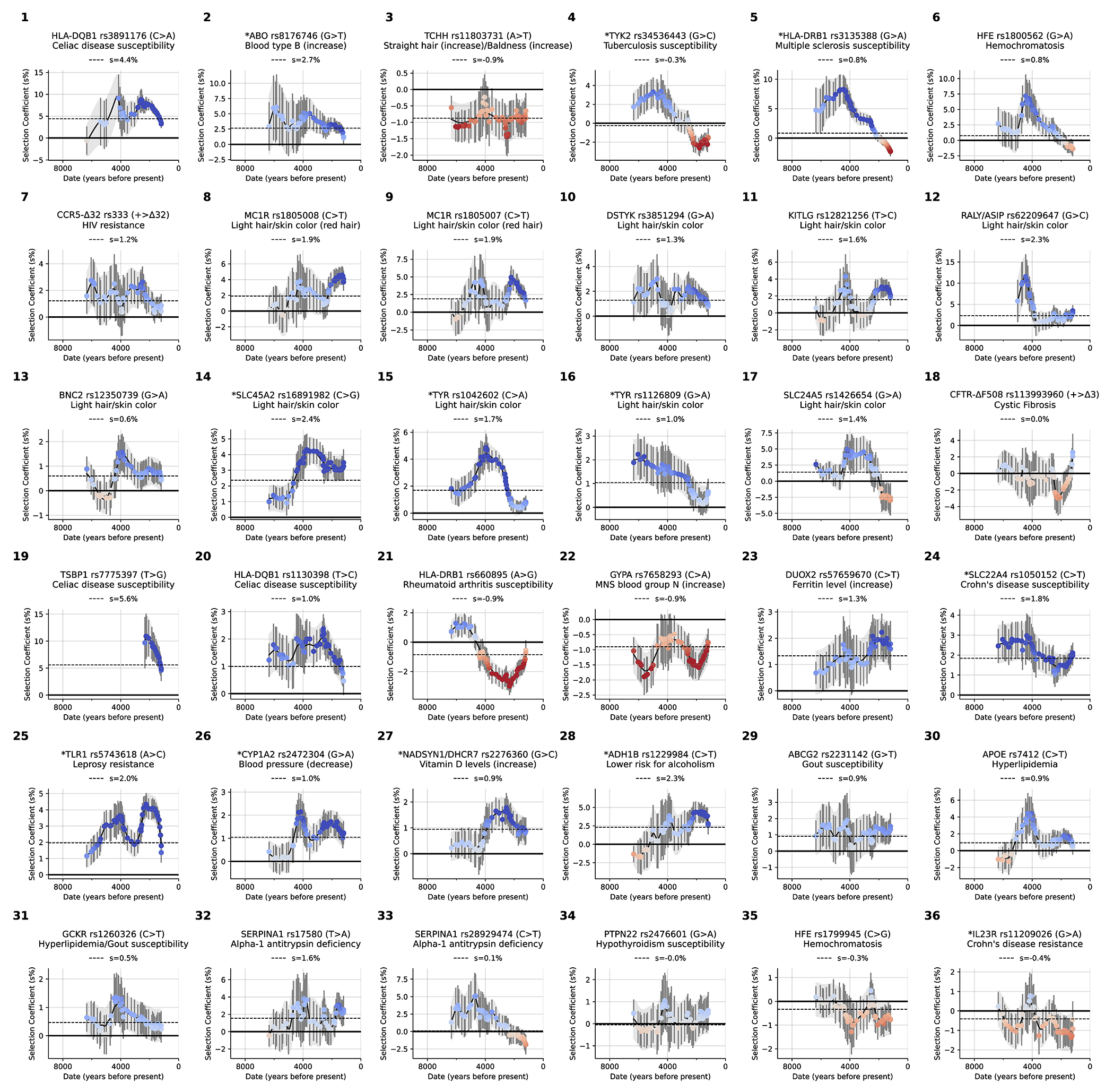
How selection coefficients on single variants changed in intensity over time (for the gallery of 36 loci also highlighted in Figure 3). Time-variant selection coefficients are estimated by refitting our model in sliding windows of 2000 years, with a step size of 100 years, and a minimum of 2000 people per window. Color map represents the Z-score for the selection coefficient being non-zero in that window, ranging from −5 (dark red) to 5 (dark blue). The solid horizontal black line indicates 0, and the dashed horizontal black line represents the estimated selection coefficient using all data in this study (Figure 3). Error bars show the 95% confidence interval, and shaded areas represent smoothed point estimates with 95% confidence intervals, obtained using the GaussianProcessRegressor function from the Scikit-learn library in Python. We parameterize this function with alpha = 1e-5 and a 1*RationalQuadratic kernel, with length_scale_bounds set to (1, 1e6). The x-values correspond to the median date of samples in each bin. Present-day samples are excluded because their disproportionate density shifts the median to the present, forcing the bin value to 0 and creating a ~1000-year jump on the x-axis, causing computational and visual inconsistencies.

**Figure 3:**
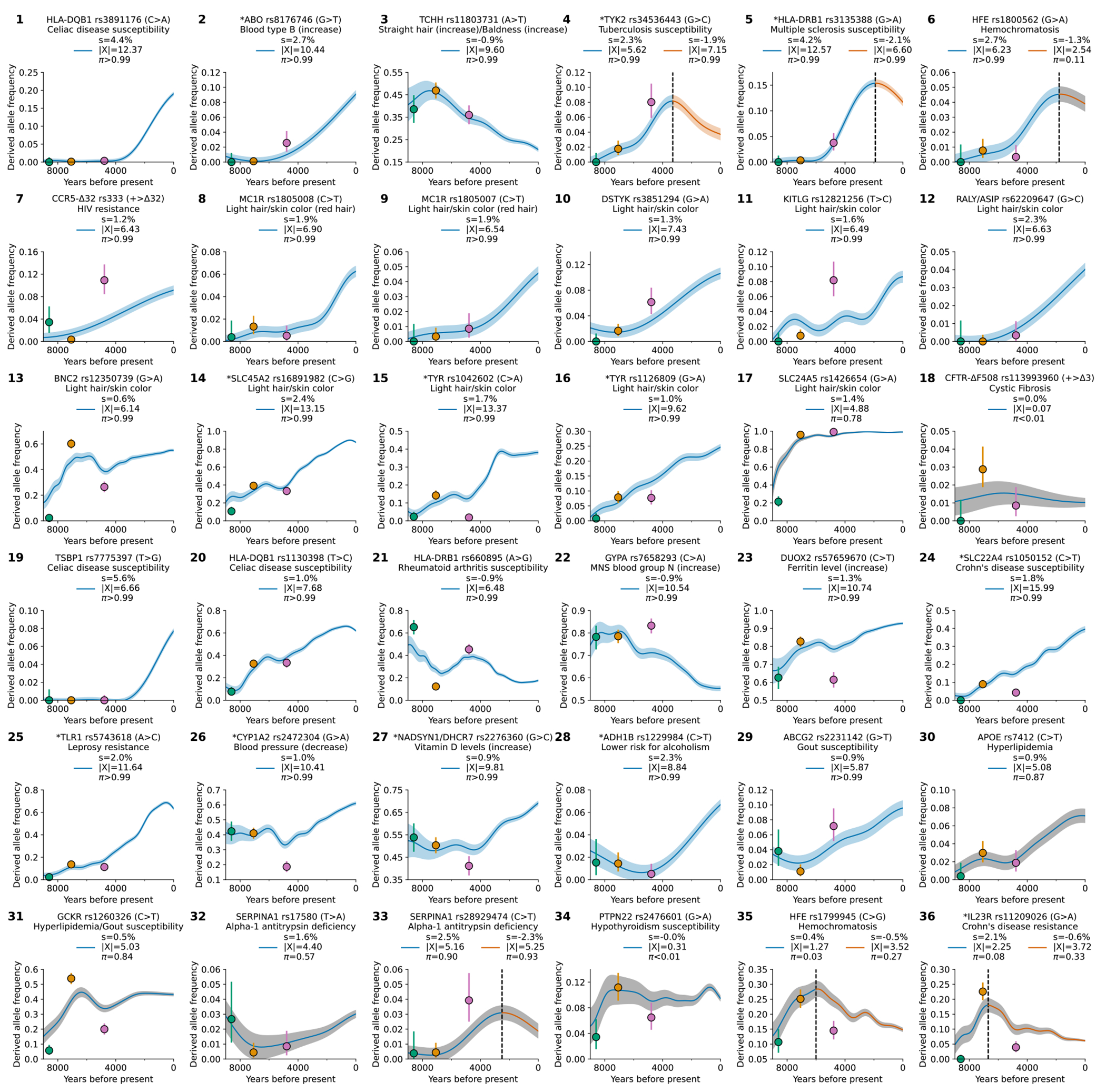
Gallery of single variant allele frequency trajectories. Each panel displays the derived allele frequency trajectory (solid line) over time for a variant (uncorrected for structure), along with selection coefficient (*s*), selection statistic (X), and posterior probability of selection (*π*). Shaded regions represent 95% confidence intervals around the estimated trajectory. Circles represent frequencies in Western Hunter-Gatherers (green, n=131), Early European Farmers (orange, n=452), and Steppe Pastoralists (pink, n=293); error bars indicate 95% confidence intervals. The highlighted variants are not necessarily those with the strongest signals and may include negative results or variants in partial linkage disequilibrium. We highlight them here because of their biological interest and because they speak to long-standing debates. For Panels 4, 5, 6, 33, 35, and 36, separate analyses are shown for transects before and after a manually selected peak (marked by a black line). In cases where *π*>99%, the confidence interval is shaded blue (or blue before and red after the split); otherwise, the shading is gray. Variants reported in other ancient DNA studies are marked with an asterisk.

We present a gallery of 36 single-allele trajectories of particular interest (Figure 3) as well as estimates of how their selection coefficients changed over time (Extended Data Figure 6). These loci are not necessarily those with the largest X-scores, but are highlighted as they address long-standing debates. They include 27 passing the *π*>99% threshold, 5 with probable evidence of selection (57%<*π*<93%), and 4 with surprising negative findings.

### *HLB-DQB1:* Selection in favor of the major risk factor for celiac disease (panel 1).

**Extended Data Figure 7:**
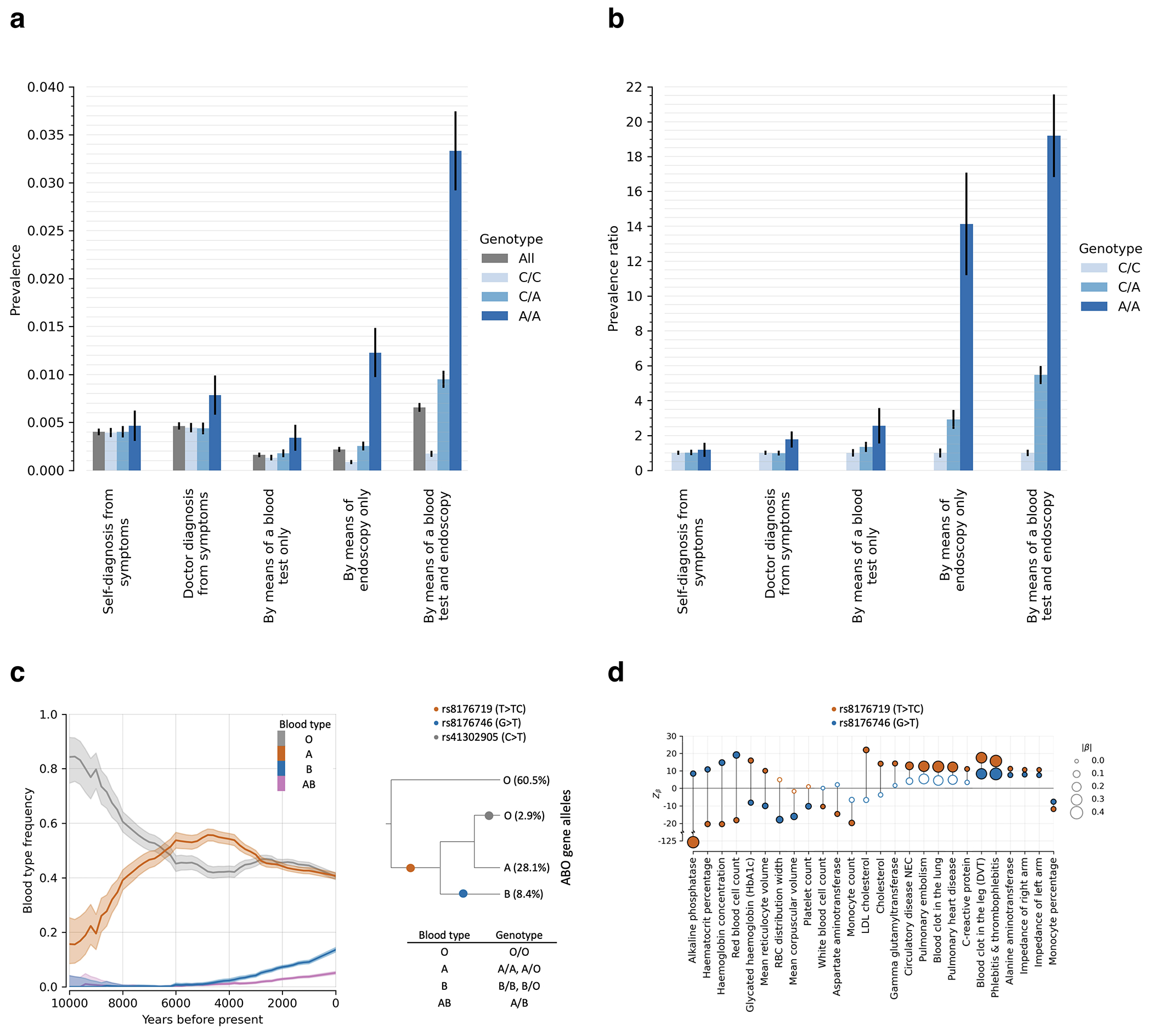
Genotype-phenotype correlations for the signals of selection for Celiac disease at HLA and the ABO blood group locus. **(a)** Prevalence and **(b)** prevalence ratio of celiac disease or gluten sensitivity (UK Biobank field 21068), conditioned on the genotype of rs3891176 (C>A) in 337,391 individuals of European ancestry from UK Biobank. The prevalence ratios are compared to the A/A genotype as a baseline. Bars show prevalence or prevalence ratio estimates; error bars are 95% confidence intervals. **(c)** Left: Blood type frequency trajectories for O, A, B, and AB estimated from our aDNA time series. Right: Genealogy of the ABO alleles approximated by Shelton et al. 2021^268^. The allele frequencies are estimated from Europeans in the 1000 Genomes Project; shading gives 95% confidence interval around the estimated allele frequency trajectory. **(d)** Significant association to traits in UK Biobank for the two base pair insertion rs8176719 (T>TC) and SNP rs8176746 (G>T), approximating the alleles A and B.

At the HLA region of chromosome 6, densely packed genes play key roles in microbe recognition. rs3891176 (C>A, meaning that the ancestral allele is C and the newly arising mutation is A) is an excellent tag for HLA-DQB1*02/DQ2, with individuals carrying two A alleles having a 19-fold higher susceptibility for celiac disease or gluten sensitivity (Extended Data Figure 7a,b). The A allele has a selection coefficient of *s*=4.4% (*π*>99%), rising from ~0% to ~20% in the last 4000 years. These findings suggest that the pathogenic exposures that drove its rise were not a phenomenon only or largely of the rise of agriculture^27^.

### *ABO*: Positive selection for B at the expense of A (panel 2).

The *ABO* gene modifies oligosaccharides in glycoproteins on the surface of red blood cells; its A, B, and null (O) alleles encode distinct glycoprotein alterations that modulate resistance and susceptibility to diverse pathogens^28^. The B allele rose from ~0% to ~10% over the last ~6000 years (*s*=2.7%, *π*>99%), and was matched by a concomitant decrease in A frequency. The two alleles are associated with opposite effects on many phenotypes, suggesting that with changing pathogenic exposures, the optimal balance changed (Extended Data Figure 7c,d).

### *TCHH*: Selection for an allele that reduced male pattern baldness (panel 3).

An allele at missense SNP rs11803731 (A>T) in *TCHH* is a strong predictor of straight hair and male pattern baldness in Europeans. The derived allele T is rare in African and East Asians, and has been hypothesized to have been positively selected^29^, but we detect negative selection (*s* = −0.9%, *π*>99%), decreasing from ~50% to ~20% in the past 7000 years. This is expected to have decreased baldness by 1.8% compared to the no-selection expectation.

### *TYK2:* Reversal of selection at a major risk factor for tuberculosis (panel 4).

Individuals carrying two copies of the rs34536443 G>C allele have >80% prevalence of clinically significant tuberculosis^30^. Previous work^30^ found evidence of negative selection on the C allele and hypothesized it was associated with the time tuberculosis became endemic in Europe. We confirm a decrease from ~9% to ~3% in the last ~3000 years (*s* = −1.9%, *π*>99%), but also positive selection from ~9000 to ~3000 years ago, from ~0% to ~9% (*s*=2.3%, *π*>99%). This may reflect changing endemicity of different pathogens over time.

### *HLA-DRB1:* Elevated MS risk in north Europe is not due to selection on the steppe (panel 5).

A previous study^31^ discovered positive selection at the rs3135388 G>A tag SNP for the HLA-DRB1*15:01 risk factor for multiple sclerosis (MS). Because selection was already occurring in Yamnaya steppe pastoralists, and Yamnaya ancestry is most common in north Europeans, the authors argued that the higher risk for MS in northern Europeans was driven by Yamnaya ancestry and selection on the steppe. We confirm positive selection at this allele, rising from ~0% to ~16% ~6000-2000 years ago (*s*=4.2%, *π*>99%). But the primary driver of the north/south differential was not selection on the steppe (Supplementary Information section 7). First, selection began south of the Caucasus mountains in people without steppe ancestry. Second, after Yamnaya ancestry spread west, selection was stronger in northern Europe at *s* = 11.1±2.5% than in southwest Europe at *s* = 6.1±2.1% (>3500 BP); this was the main driver of the north/south differential. Third we detect negative selection in the last ~2000 years missed by previous work (*s* = −2.1%, *π*>99%), plausibly reflecting new pathogen exposures.

### *HFE*: Reversal of selection at the major risk factor for hemochromatosis (panel 6).

The rs1800562 (G>A) allele predicts pathogenic iron buildup in cells in individuals with two copies, and we find evidence of positive selection from ~5000-2000 years ago, rising from ~1% to ~5% (*s* =2.7%, *π*>99%), then dropping to ~4% today (*s* = −1.3%, by itself not significantly different from zero at *π*=11%, but the decrease relative to the earlier positive selection is significant at P = 2.8x10^−9^). It was hypothesized that the causal allele protected against *Yersinia pestis* (the agent of Black Death)^32^, but this is unlikely as its frequency was decreasing by the time of the Justinianic and Medieval pandemics^33^.

### *CCR5-Δ32*: Positive selection at an allele conferring immunity to HIV-1 infection (panel 7).

The *CCR5-Δ32* allele confers complete resistance to HIV-1 infection in people who carry two copies^34^. An initial study dated the rise of this allele to medieval times and hypothesized it was selected for resistance to Black Death^35^. But improved genetic maps revised its date to >5000 years ago and the signal became non-significant^36^. We find that the allele was positively selected ~6000-2000 years ago, increasing from ~2% to ~8% (*s* =1.2%, *π*>99%). This is too early to be explained by the medieval pandemic, but ancient pathogen studies show *Yersinia* was endemic in West Eurasia for the last ~5000 years^37^, resurrecting the possibility that it was the cause, although other pathogens are possible.

### Selection for light skin at ten loci (panels 8-17).

We find nine loci with genome-wide signals of selection for light skin, one probable signal, and no loci showing selection for dark skin.

### *CFTR*: No evidence of selection for the major cystic fibrosis risk allele ΔF508 (panel 18).

The major risk allele for this recessive disease in Europeans has been hypothesized to be an example of heterozygote advantage due to conferring resistance to cholera in carriers^38^, keeping its frequency substantial despite its strong deleterious effects in homozygotes. However, we find no evidence of directional selection over the time frame that that cholera was likely endemic in West Eurasia (*π*<1%), with the earliest direct observation ~6550 BP in Croatia and the earliest imputed one ~15411 BP in Anatolia. Another explanation is needed for the allele’s persistence, plausibly by balancing selection.

Fifteen other selection discoveries are highlighted in panels 19-33 of Figure 3. Most are highly significant at π>99%: *TSBP1* (Celiac disease, *s*=5.6%); a second allele at *HLA-DQB1* (Celiac disease, *s*=1.0%); *HLA-DRB1* (Rheumatoid arthritis, *s*=−0.9%); *GYPA* (increases MNS blood group N, *s*=−0.9%); *DUOX2* (increases Ferritin level, *s*=1.3%); *SLC22A4* (Crohn’s disease, *s*=1.8%); *TLR1* (Leprosy resistance, *s*=2.0%); *CYP1A2* (decreases blood pressure, *s*=1.0%); *NADSYN1/DHCR7* (increases vitamin D, *s*=0.9%); *ADH1B* (lower risk for alcoholism, *s*=2.3%); and *ABCG2* (gout, *s*=0.9%). Four more signals are probable: *APOE* (hyperlipidemia, *s*=0.9%, *π*=87%); *GCKR* (hyperlipidemia/gout, *s*=0.5%, *π*=84%); *SERPINA1* (alpha-1 antitrypsin deficiency, *s*=1.6%, *π*=57%), and a second locus at *SERPINA1* (alpha-1 antitrypsin deficiency) which shows positive selection from ~7000–2500 years ago (*s*=2.5%, *π*=90%) followed by a reversal after ~2500 years ago (*s*=−2.4%, *π*=93%).

Panels 34-36 highlight null signals at loci previously hypothesized to be selected: *PTPN22* (hypothyroidism); a second allele at *HFE* (hemochromatosis); and *IL23R* (Crohn’s disease).

## Tests for selection on complex traits

We searched for evidence that groups of alleles with similar influence on traits today trended in the same direction in the past, as expected if a phenotype with a similar genetic underpinning was the target of selection. We leveraged 563 GWAS results in people of European ancestry: 452 mostly quantitative traits in the UK Biobank, and 111 curated traits from studies especially of common disease (Supplementary Table 5). How phenotypes manifest today may be very different from how they manifested in past populations living in different environments with different lifestyles, so any signals discovered by this approach should not be interpreted as evidence for selection on the exact phenotype being tested.

**Extended Data Figure 8:**
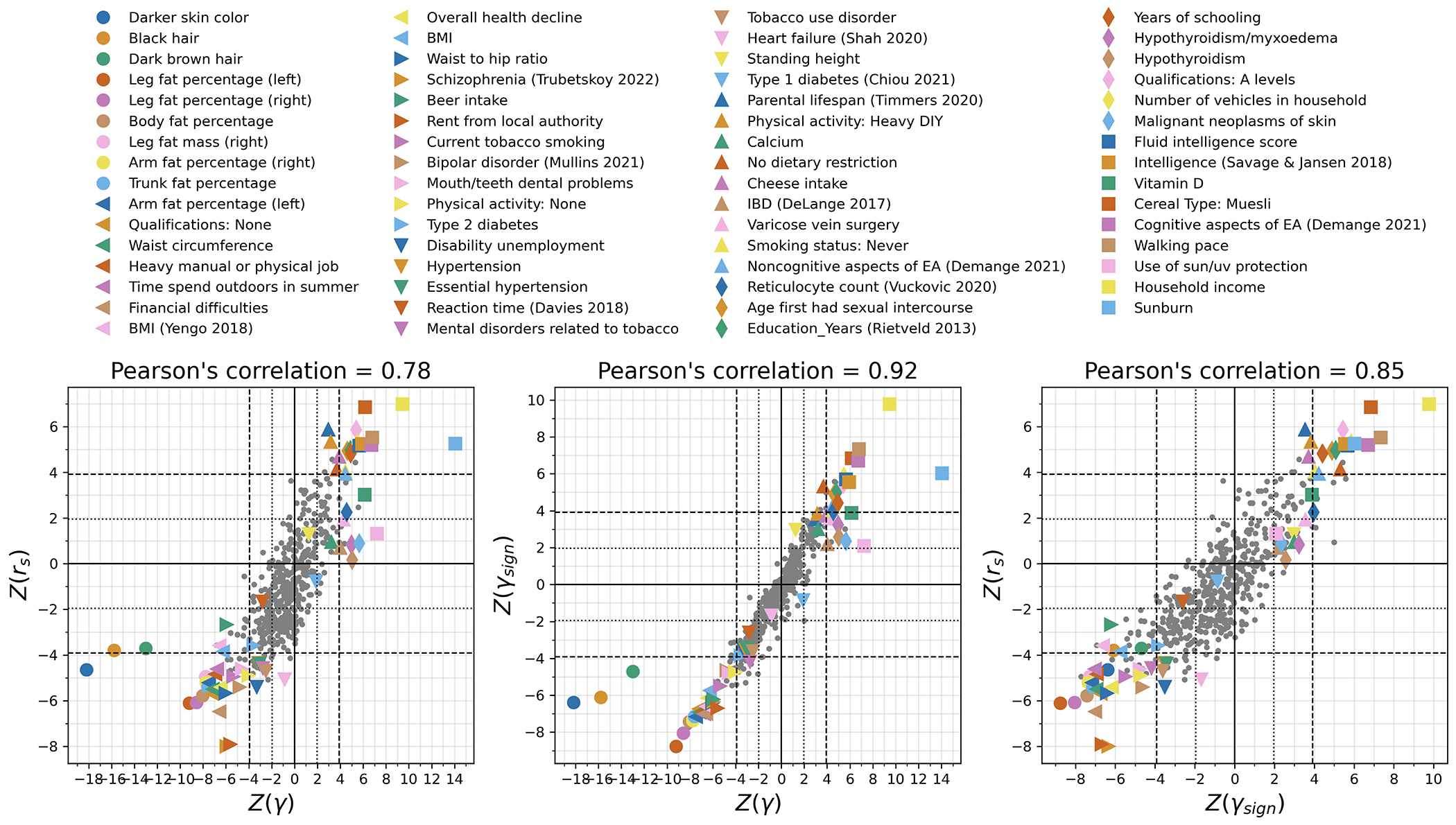
High correlation of 3 tests for polygenic selection (*γ*, *γ*_sign_, r_s_). Each dot represents a phenotype, some annotated by colors. Pearson’s correlation for x and y axes at top. The dashed line indicates the Bonferroni-corrected significance threshold (P = 8.9x10^−5^, correcting for 563 GWAS tested), and the dotted line indicates the uncorrected nominal significance threshold (P = 0.05). All P values are two sided.

We used three statistics to test for coordinated selection on alleles affecting the same trait (we excluded variants at the HLA locus as we were interested in polygenic signals, not ones dominated by large effect variants). First, we computed a polygenic score (PGS) for each GWAS: a linear combination of allelic values, weighted by estimated effect size. We evaluated whether the change in PGS over time χ (scaled so one unit corresponds to a standard deviation change over ten millennia) is more than could be expected by genetic drift or population structure, using the genetic relatedness matrix to control for these effects as in our single allele tests. To test if the observed deviation is significant, we repeated the test 100 times with randomly flipped signs of GWAS effects, to correct for residual inflation. Second, we repeated the procedure without using magnitudes of GWAS effects, and instead only the sign, generating a statistic *χ*_sign_ that may be less affected by concerns about transferability of PGS across groups^3,39^. Third, we performed a SNP-by-SNP comparison for each trait, using cross-trait LD Score Regression (LDSC)^40^ to estimate genetic correlation (*r_s_*) between selection coefficients (s) and trait effect sizes. We computed a standard error from a Block Jackknife to test if this correlation is different from zero, accounting for non-independence of SNPs. We find high Pearson’s correlation for all three tests (77-90%; Extended Data Figure 8).

For 31 traits, we repeated analyses using effect sizes measured in East Asian GWAS. Population structure in East Asia is uncorrelated to that in ancient West Eurasians, so any validation by this test must reflect a real signal of selection^3,39^.

For 34 traits repeated analyses using effect sizes estimated from family-based GWAS, which distinguish “direct effects” of genetic variants on the biology of the individual being examined, from “indirect effects” that affect the trait through modulating the behavior of family members and thus changing the individual’s environment. Despite much reduced sample sizes, standard errors are small enough for some traits to validate our findings.

Multiple lines of evidence point to the robustness of our tests of polygenic selection to population structure. First, we carried out ordinary linear regression searching for evidence of a change in the polygenic predictor of height over time and found a significant signal without correction for structure (P =7x10^−159^)^41,42^, which disappeared after our correction: (γ, P=0.21). Second, we observed strong Pearson’s correlations between Z-scores for European and East Asian GWAS of 0.43 (γ), 0.75 (γ_sign_), and 0.81 (*r_s_*) (Extended Data Figure 12a); population structure is uncorrelated for Europeans and East Asians, and thus structure artifacts cannot explain this signal. A third line of evidence comes from positive correlations between Z-scores from population GWAS and direct genetic effect GWAS from family-based studies: 0.51 (γ), 0.51 (γ_sign_), and 0.76 (*r_s_*)) (Extended Data Figure 12b). Fourth, we do not observe false-positives in realistic simulations of European structure without directional selection (Supplementary Information section 2).

## Directional selection on complex traits

**Extended Data Figure 9:**
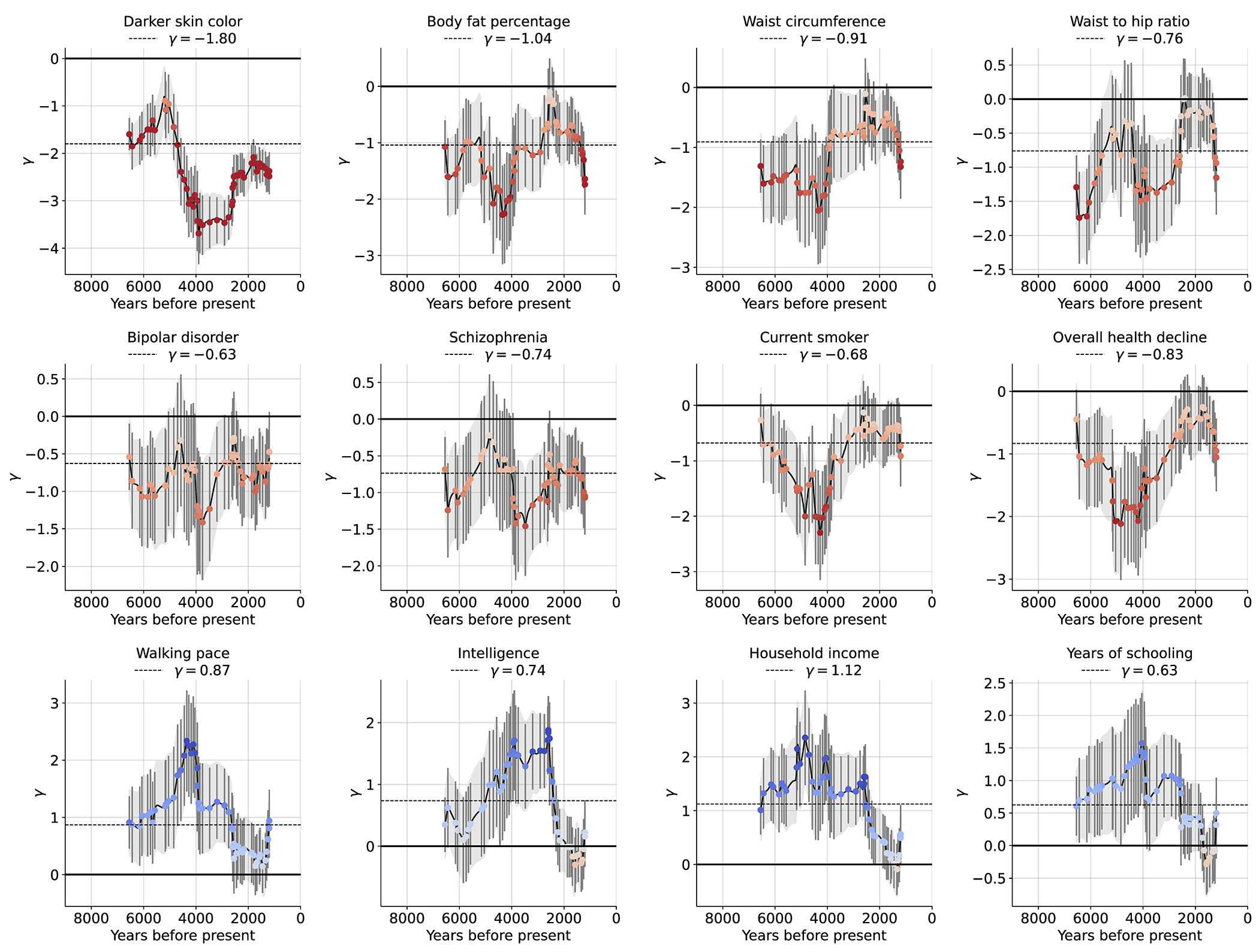
How coordinated selection on alleles affecting the same traits changed in intensity over time (gallery of 12 complex traits also highlighted in Figure 4). We estimate time-variant polygenic selection intensity *γ* by refitting our model in sliding windows of 2000 years, with a step size of 100 years, and a minimum of 2000 people per window. Color map represents the Z-score for the selection coefficient being non-zero in that window, ranging from −5 (dark red) to 5 (dark blue). The solid horizontal black line indicates 0, and the dashed horizontal black line represents the estimated *γ* using all data in this study (Figure 4). Error bars show the 95% confidence interval around the estimated *γ* in each window, and shaded areas represent smoothed point estimates with 95% confidence intervals, obtained using the GaussianProcessRegressor function from the Scikit-learn library in Python. We parameterize this function with alpha = 1e-3 and a 1*RationalQuadratic kernel, with length_scale_bounds set to (1, 1e6). The x-values correspond to the median date of samples in each bin. Present-day samples are excluded because their disproportionate density shifts the median to the present, forcing the bin value to 0 and creating a ~1000-year jump on the x-axis, causing computational and visual inconsistencies.

**Figure 4:**
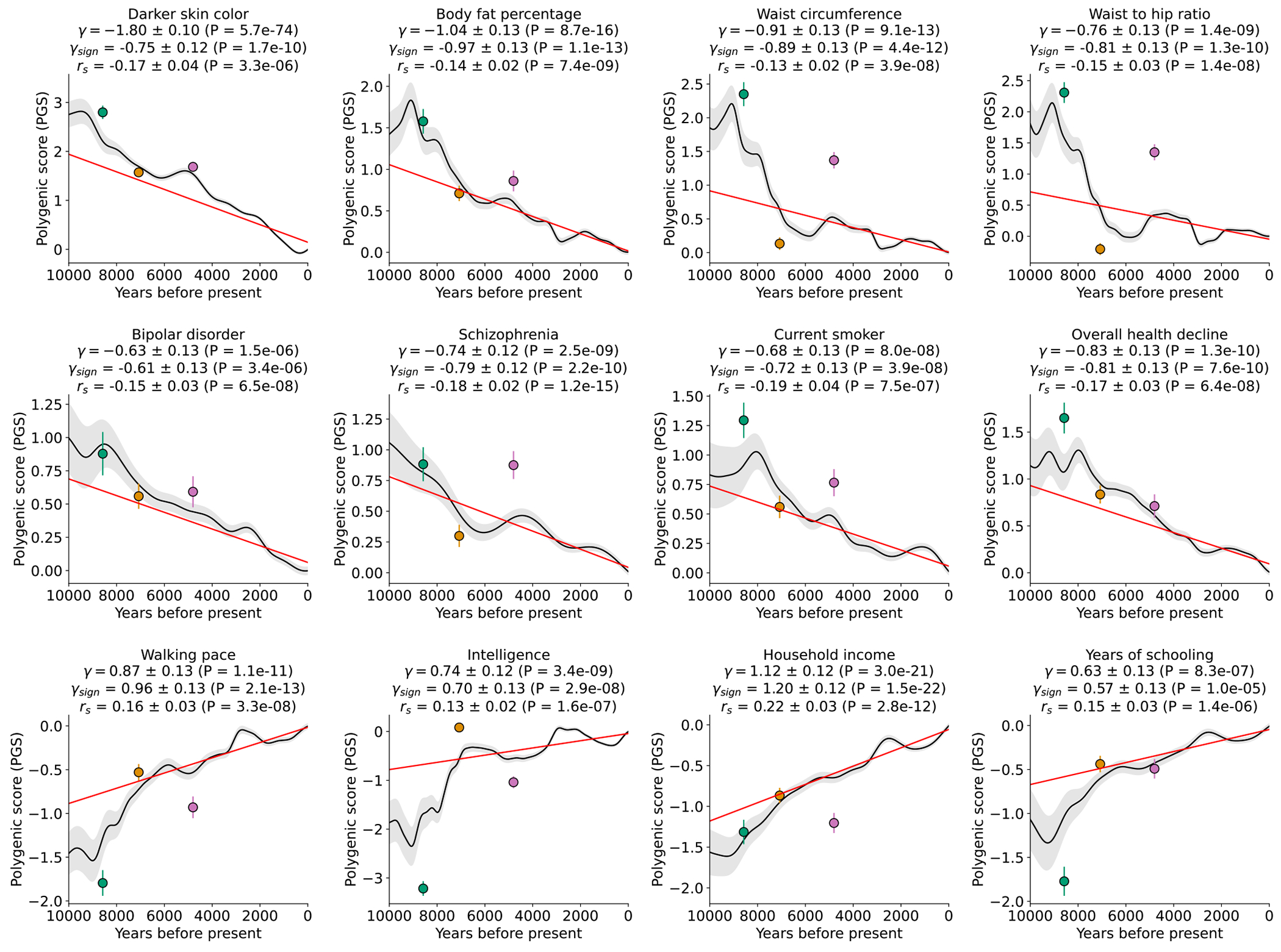
Notable signals of directional polygenic selection. We show twelve instances where tests of polygenic selection—*γ*_sign_, and *r*_*s*_—are all significant, with the relevant statistics at the top of each panel. The mean polygenic score of Western Eurasians over 10000 years is shown in solid black line, with 95% confidence interval in gray. Red represents the linear mixed model regression, adjusted for population structure, with slope *γ*. Circles represent mean polygenic score in Western Hunter-Gatherers (green, n=131), Early European Farmers (orange, n=452), and Steppe Pastoralists (pink, n=293); error bars indicate 95% confidence intervals. All P values are two sided and evaluated against a Bonferroni-corrected threshold for multiple testing (P < 8.9x10^−5^).

In total, 44 of 563 GWAS show significant signals across all three tests after correction for the number of GWAS examined (P < 8.9x10^−5^), and 100 GWAS show signals in at least one test after correction for multiple hypothesis testing ( P < 3.0x10^−5^) (Supplementary Table 5). We focus on 12 traits of particular interest with significant signals from all three tests (Figure 4, Extended Data Figure 9).

**Extended Data Figure 10:**
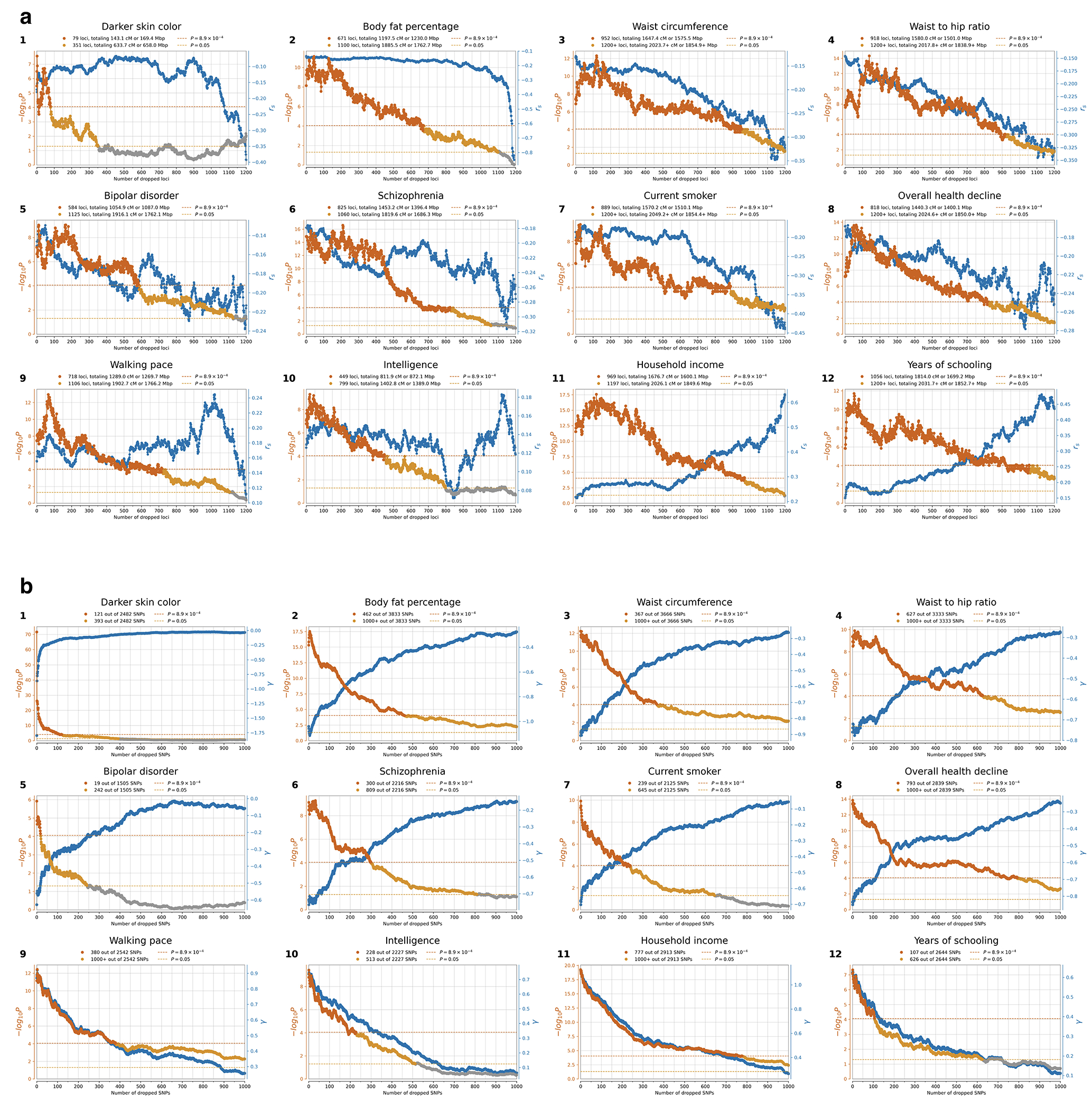
Estimating the minimum number of SNPs affected by selection for each trait (gallery of 12 traits also highlighted in Figure 4). **(a)** Each panel shows the LDSC genetic correlation (*r*_*s*_) between the trait effect sizes and the selection coefficients (s) as a function of number of dropped loci. The right axis displays *r*_*s*_ in blue; P-value on the left axis in orange. For each SNP, we define a priority score | β × s × f × (1-f) |, where β is the GWAS effect size, *s* the selection coefficient, and *f* allele frequency. SNPs are sorted by priority score, and in each iteration, a 2cM region around the highest priority SNP is dropped, *r*_*s*_ is recalculated for the remaining genome, and this continues until no SNPs are left. **(b)** We similarly show *γ* estimates at right as a function of number of dropped SNPs (blue), and P-value for polygenic selection at left, with dark orange indicating P<8.9x10^−5^ (Bonferroni-corrected threshold for 563 GWAS tests), light orange P<0.05, and gray otherwise. All P values are two sided.

**Extended Data Figure 11:**
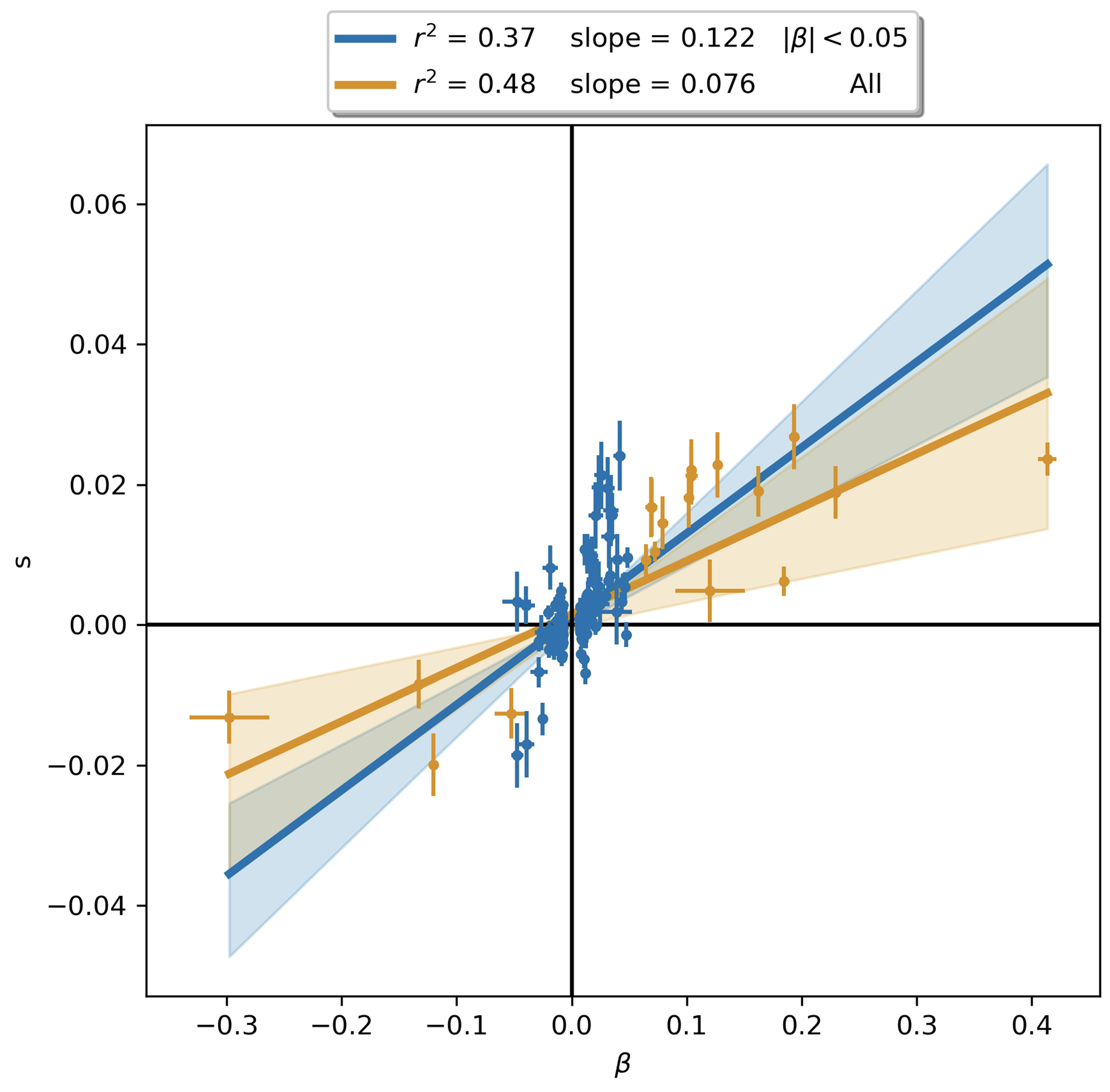
Pigmentation is oligogenic but selection on it was polygenic. Selection coefficient (s) and effect size (β) from the UK Biobank skin color phenotype for 150 independent SNPs passing the GWAS P-value threshold of p<5x10^−8^. Following ^43^, which applied ordinary least squares regression, we instead used orthogonal distance regression (ODR) to account for uncertainty in both variables. The orange line was fit to all SNPs (131 blue and 19 orange markers), whereas the blue line was fit only to SNPs with |β| < 0.05 (131 blue markers). Neither the correlations (difference between Fisher Z-transformed Pearson’s correlation, P = 0.22) nor the slopes (P = 0.11) differ significantly, consistent with a model in which selection for pigmentation had an equal impact on all variants in proportion to effect size. Shaded areas indicate 95% confidence bands. Points show β estimated in 415,018 UK Biobank individuals (x-axis) and s estimated from 20,374 unrelated individuals (y-axis); error bars show 95% confidence intervals. All P values are two sided.

One of the strongest signals is an increase over time in the PGS for light skin pigmentation (*χ* =1.80**±**0.10 standard deviations increase in mean PGS in ten millennia, P=5.7x10^−74^; Figure 4, Extended Data Figure 9). This plausibly reflects selection for increased synthesis of vitamin D in regions of low sunlight in farmers with little of it in their diets. Most of the phenotypic shift is driven by a handful of loci^43^: 52% due to *SLC45A2* alone, and 75% to the top 10 loci, but the signal is highly polygenic: we need to drop the top 79 loci before the signal disappears (Extended Data Figure 10). A model in which selection for pigmentation impacted all variants in proportion to their effect size fits (P=0.11; Extended Data Figure 11).

Type 2 diabetes risk factors give compelling signals of negative selection. Thus, we observe negative selection on combinations of alleles that today increase body fat percentage (*χ* = −1.04**±**0.13), waist circumference (*χ* = −0.91**±**0.13), and waist-to-hip ratio (*χ* = −0.76**±**0.13) (Figure 4), which could be consistent with the “Thrifty Genes” hypothesis^44^ that a genetic adaptation to store fat to promote survival during periods of scarcity in hunter-gatherers, became deleterious after the transition to food-production. Skeletal evidence for nutritional stresses in early European farmers^45–48^ could be viewed as in tension with this hypothesis, but is consistent with it if a farming lifestyle was associated with famines that occurred on too-infrequent a time scale to be effectively buffered by higher fat. The signal of selection against the polygenic predictor of type 2 diabetes itself (*χ* = −0.43**±**0.12) just misses the multiple hypothesis-testing corrected threshold, but the other two tests are highly significant (*χ*_sign_ = −0.48**±**0.12; *r_s_* = −0.14±0.04).

We detect negative polygenic selection against alleles associated today with psychoses such as bipolar disorder (*χ* = −0.63**±**0.13) and schizophrenia (*χ* = −0.74**±**0.12) (Figure 4). These results might at first seem surprising given our finding that significant signals of selection are not enriched for variants with genome-wide significant evidence of modulating psychiatric traits in GWAS (Figure 1d). But for variants with sub-genome-wide-significant signals in GWAS, we do observe heritability enrichment^49^ (Extended Data Figure 5a). Brain traits have qualitatively different genetic architectures from blood-immune-inflammatory traits, with a higher proportion of sites modulating them and smaller effect sizes on average per allele^50^. Thus, our observations likely reflects a situation whereby alleles with strong enough selection coefficients to be significant are concentrated in immune traits, while selection on brain traits is so polygenic that most individual contributing alleles have too-small selection coefficients to be detected. Indeed, directional selection against the genetic predictors today predisposing to psychosis is extraordinarily polygenic: we have to drop 584 loci for bipolar disorder and 825 loci for schizophrenia for the signals to disappear (Extended Data Figure 10).

We observe signals of selection for combinations of alleles that are today associated with healthy lifestyles into old age. This includes selection against alleles today associated with smoking (*χ* = −0.68**±**0.13), against alleles contributing to overall health decline (*χ* =−0.83**±**0.13), and for alleles that promote faster walking pace (*χ* = 0.87**±**0.13).

We finally observe signals of selection for combinations of alleles that today are associated with three correlated behavioral traits: scores on intelligence tests (increasing *χ* = 0.74±0.12), household income (increasing *χ* = 1.12±0.12), and years of schooling (increasing *χ* = 0.63±0.13). These signals are all highly polygenic, and we have to drop 449 to 1056 loci for the signals to become nonsignificant (Extended Data Figure 10). The signals are largely driven by selection before ~2000 BP, after which *χ* tends toward zero (Extended Data Figure 9).

**Extended Data Figure 12:**
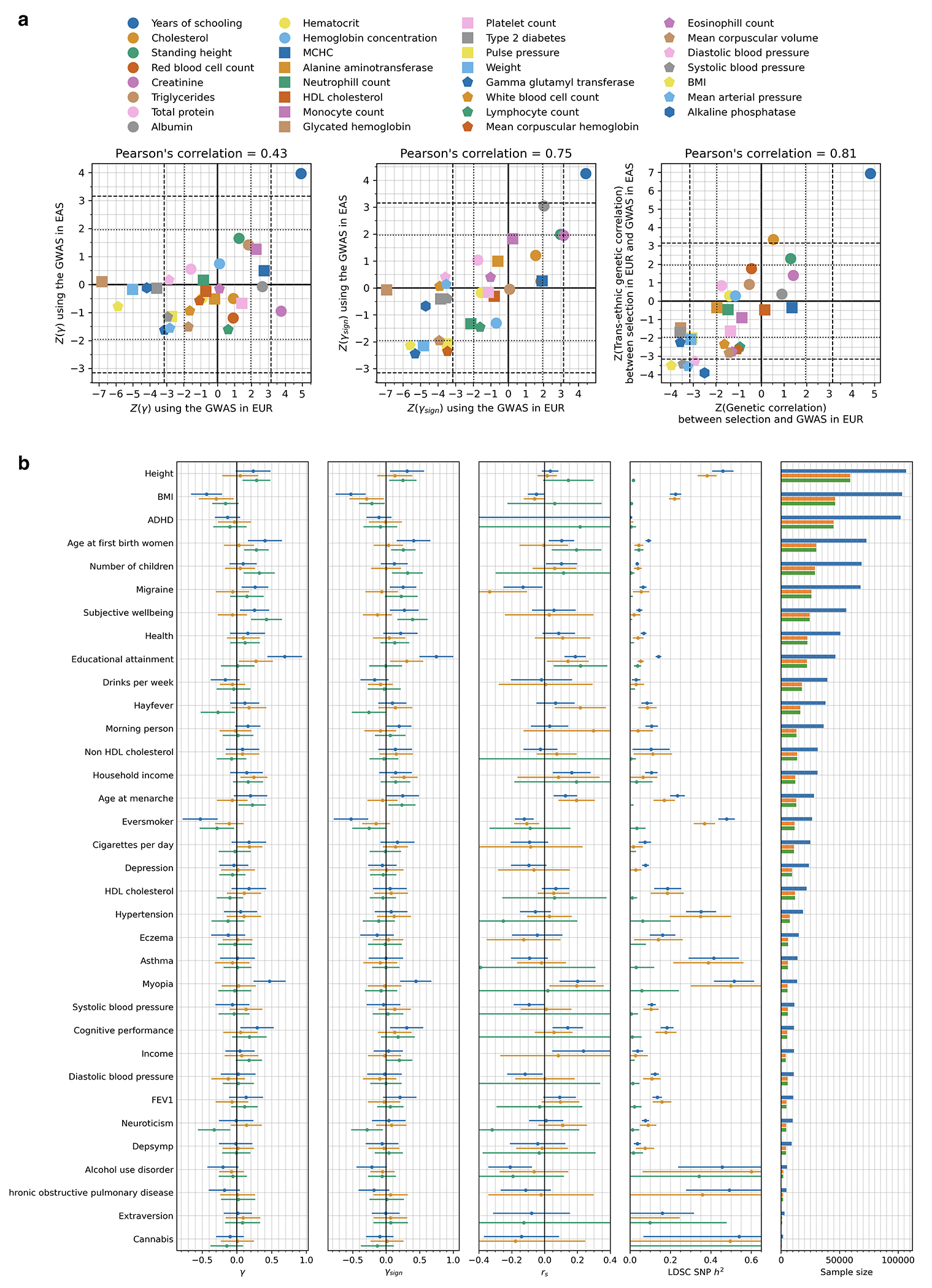
Replication of polygenic selection signals using effect sizes from East Asian GWAS and consistency between population-based and family-based GWAS. **(a)** Replication of signals of polygenic selection using effect size estimates in East Asians whose population structure is uncorrelated to West Eurasians. We applied our polygenic selection test to 31 traits using pairs of GWAS studies for the trait, one from Europe and one from East Asia. We assessed *γ*, *γ*_sign_, and *r*_*s*_ in the two analyses and found them to be consistent. It is extremely difficult to believe that an estimate based on effect size estimates in East Asians could be an artifact of population structure, since structure within Europe and within East Asia are uncorrelated. This comes at the cost of reduced power relative to using European GWAS directly, driven by the smaller sample sizes of East Asian GWAS (median 30% of European GWAS), differences in LD structure between populations, and divergence in genetic architecture (allele frequencies and causal effect sizes)^51–53^. Despite reduced power, Pearson’s correlations between Z-scores for European and East Asian GWAS are 0.43, 0.75, and 0.81 for γ, γ_sign_, and *r*_*s*_, respectively, a positive correlation that must reflect real selection and would not be expected due to population structure. The dashed line indicates the Bonferroni-corrected significance threshold (P = 1.6x10^−3^, correcting for 31 traits), and the dotted line indicates the nominal significance threshold (P = 0.05). Years of schooling is the only trait that reaches Bonferroni-corrected significance threshold for all three tests. **(b)** Consistency of polygenic selection signals using effect sizes estimated from GWAS of unrelated people and family-based GWAS. We analyze the GWAS results reported in ^54^, who compiled whole genotype-phenotype correlation data in families for 34 phenotypes, and report three GWAS for each phenotype: unrelated people (“population GWAS”), and direct and indirect effect GWAS obtained by differentiating between transmitted and untransmitted chromosomes within families. The first three columns show estimates for three polygenic tests of selection. The fourth column displays the estimated SNP heritability (h^2^) by LDSC. The fifth column shows the sample size, with the median sample size for family GWAS ~13,000 and for population GWAS ~31,000. Both are only a fraction of the UK Biobank cohort of ~490,000 individuals of non-Finnish European ancestry^269^, explaining the reduced power and why confidence intervals are wide and often overlap zero. The Pearson’s correlations between Z-scores from population GWAS and direct genetic effect GWAS are 0.51, 0.51, and 0.76 for γ, γ_sign_, and *r*_*s*_, respectively. When restricted to population GWAS signals with nominal significance (P < 0.05), the correlations increase to 0.84, 0.78, and 0.81, respectively. Among population GWAS in ^54^, educational attainment, ever-smoker, and myopia reach the Bonferroni-corrected significance (P < 1.5x10^−3^ , correcting for 34 traits) for all three tests. For direct genetic effects, educational attainment is the only trait with all three tests nominally significant (P < 0.05). Blue indicates population GWAS, orange represents direct genetic effects from family GWAS, and green represents the average of paternal and maternal non-transmitted coefficients (NTCs) from family GWAS. Points show the estimated statistic; error bars indicate 95% confidence intervals. All P values are two sided.

We were concerned that the evidence of polygenic selection for behavioral traits might be an artifact of uncorrected population structure in West Eurasia, despite our simulations showing that our methodology corrects for such structure. To explore this, we first focused on years of schooling, for which available GWAS were largest. We tested for a correlation of East Asian GWAS effect size measurements to West Eurasian selection coefficients. Despite reduced power—driven by the smaller sample sizes of East Asian GWAS, and differences in LD structure and genetic architecture across populations^51–53^—we replicate the signal of selection which cannot be explained by imperfectly corrected West Eurasian population structure, which is uncorrelated to that in East Eurasia: γ (P=7.3x10^−5^), γ_sign_ (P=2.1x10^−5^) and *r_s_* (P=3.9x10^−12^) (Extended Data Figure 12a). We also replicate using family-based GWAS^54^ which is immune to population structure (Extended Data Figure 12b). Despite limited power of these studies due to small sample sizes, the polygenic score for direct effects on years of schooling showing nominal evidence of positive selection in West Eurasians by all three tests: γ(P=0.027), γ_sign_ (P=0.015), and *r_s_* (P=0.029). The score capturing indirect genetic effects—parental alleles not passed to offspring and thus modulating parental behavior and family environment—shows a signal in one test: γ(P=0.920), γ_sign_ (P=0.945), and *r_s_* (P=0.009).

We could not obtain insight into the phenotype that was being selected in the past through functional stratification of the years of schooling signal. Thus, when we compared GWAS for the cognitive and non-cognitive components of the years of schooling trait^55^, both showed significant signals individually, but their pairwise differences were not significant: all three tests were P>0.05 (Supplementary Table 5). We also analyzed brain volume^56^, which is correlated with years of schooling (LDSC genetic correlation, *r_g_* = 0.26±0.03) and found no signals (all P > 0.05).

**Extended Data Figure 13:**
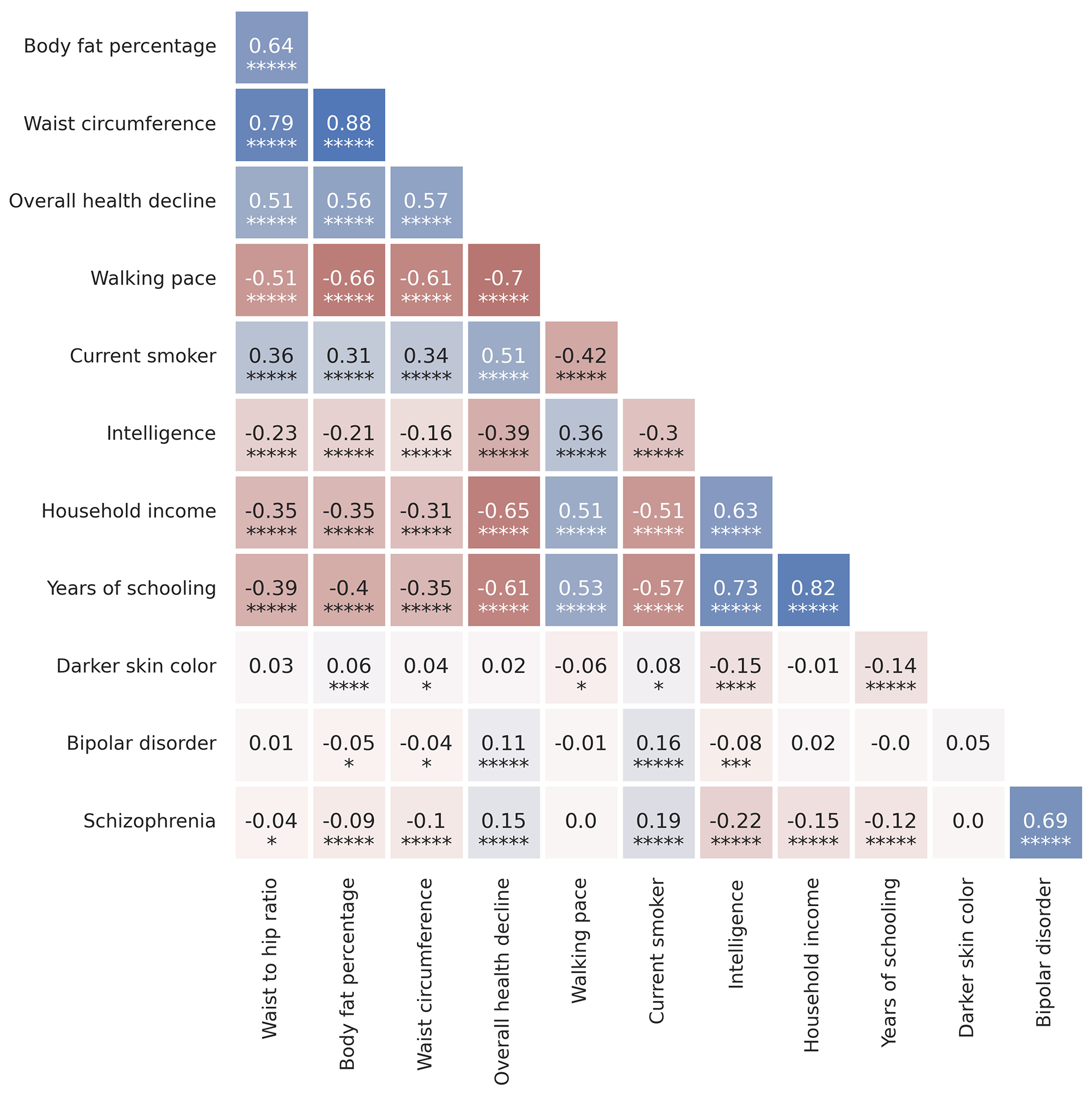
Correlations of polygenic scores for complex traits with strong evidence of coordinated selection (the same 12 traits highlighted in Figure 4). Genetic correlations of traits are computed using LDSC based on modern UK Biobank data (no ancient DNA are used in this analysis). Asterisks indicate significance level (n asterisks represent a jackknife estimated P<0.5x10^−n^). All P values are two sided. A caution is that some of these genetic correlations may be inflated due to assortative mating.

There are caveats when interpreting signals of polygenic adaptation, especially for the three genetically correlated traits of scores on intelligence tests, household income, and years of schooling. These traits are only relevant to modern societies, and would have been unmeasurable in preliterate societies over the vast majority of the period during which selection acted. The difficulty of interpretation is enhanced by the fact that the alleles driving down the frequency of type 2 diabetes-related traits, are highly correlated to those contributing to the increased scores for years of schooling, household income, and intelligence (Extended Data Figure 13). Finally, there is evidence of change in these selection pressures over time^57^, for example, in Iceland in the last century where there was significant selection to decrease the predictor of years of schooling, opposite to the long-term increase we detect.

## Discussion

Previous work has shown that classic selective sweeps driving alleles to fixation have been rare over the broad span of human evolution^8,9^. Thus, we were surprised that over the last 10,000 years in West Eurasia, there have been many hundreds of instances of directional selection with coefficients on the order of 0.5% or more (Figure 2b). This is large enough that if a similarly dense landscape of directionally selected variants had existed tens of thousands of years ago, and if the selection coefficients had been constant since then, we would expect many fixed differences across populations, despite the fact that previous studies have shown there are only a handful, hardly more than would be expected based on random drift^9^.

The simplest way to resolve this paradox is to recognize that selection coefficients are unlikely to have been constant over time, even though we make this simplifying assumption to enable detection of selection. By sliding a 2000-year window through our time transect and re-estimating selection coefficients within each window (Extended Data Figure 6, Figure 3), we can see that there have in fact been changes in selection pressures at a number of the loci we analyze, including at *HLA-DRB1, TYK2* and *HFE*. By comparing the estimated age of the mutation that contributed each selected allele^58^, to the extrapolated time to reach fixation given its estimated *s*-value, we find that about one third of the favored mutations are ancestral alleles and another third are derived alleles with true ages an order of magnitude older than the expected sweep age, indicating that selection coefficients must have shifted over time at these variants (Figure 2c). The remaining up to a third of the signals we detect are consistent with classic sweeps where the newly arising variants have been under consistent positive selection since they arose (but they arose recently enough that the sweeps are not complete).

A second explanation for this paradox is that West Eurasians have been experiencing qualitatively more and different natural selection in the Holocene than in earlier periods because of rapidly changing lifestyles and economies. This hypothesis is supported by our evidence of intensifying selection for traits including blood-immune-inflammatory ones in the Bronze Age compared with earlier periods (Figure 1e, Extended Data Figure 5b).

Although the exact number of independent loci cannot be estimated with high confidence, we project that there are at least 3800 independent signals of directional selection (half of the 7689 non-HLA loci at the FDR=50% threshold) that are in linkage disequilibrium with the overwhelming majority of variants in the genome (Extended Data Figure 2b) superficially, this appear to be at odds with findings that there has been relatively little contribution from directional selection to allele frequency changes in genome compared to the much larger forces of gene flow, genetic drift, and purifying or stabilizing selection^6^. In fact, there is no conflict. Our method allows us to partition the effects of selection at each SNP into the influences of directional selection (*s*), and the combined effects of fluctuating selection and drift (*σ*^2^). We estimate that only 2.16±0.11% (jackknife standard deviation) of allele frequency changes are due to directional selection. Allele frequency change in human populations has been so rampant that even if a small fraction of allele-frequency change is due to directional selection, this corresponds to many thousands of loci.

We ruled out the possibility that our results are reflecting not directional selection but stabilizing selection—selection to reduce genetic variability even if the population is near a fitness optimum—a major force shaping patterns of variation in living people^6,8,20,25,59^. Previous work showed that if there is a shift in a trait optimum, there is expected to be a deterministic directional selection phase during which the population shifts toward the optimum, following by a stochastic stabilizing selection phase when underdominant selection pushes common variants toward fixation or loss^21^. Equation 7 of ^21^ shows that the selection coefficient (*s*) in the deterministic phase scales linearly with the phenotypic effect of the allele, while in the stochastic phase it scales with the square. Assuming a phenotypic effect of ~0.01, plausible based on typical GWAS, yields a selection coefficient on the order of s ~ 0.0001. Any changes in frequency would be expected to occur at a time scale of 1/*s* (Figure 3 of ^21^), and thus, while the deterministic phase unfolds over about 100 generations, the stochastic phase spans 10,000 to 100,000 generations, implying that underdominance due to stabilizing selection is effectively indistinguishable from neutral evolution in the context of our study. Confirming this, our simulations of diverse scenarios of stabilizing selection do not produce the enrichment of GWAS signals and HAF score we observe empirically (Figures 1b,d, Extended Data Figure 4, Supplementary Information section 2).

We considered and ruled out the possibility that our observations are not evidence of directional selection, but instead a process noted by ^59,60^, whereby in a panmictic population, purifying selection can generate positive covariance between frequency changes in adjacent time intervals for neutral loci linked to newly arising deleterious mutations. Three theoretical reasons indicate that this cannot be explaining our observations. First, we lack the temporal resolution to detect such signals which have a time scale of 10-20 generations, on the order of the standard error of estimated dates in ancient DNA. Second, our study spans hundreds of generations, so the signals we detect show autocovariance on a far longer time scale than can be explained by this phenomenon. Third, the population in our study is not panmictic as in ^60^; on a time scale of 10-20 generations, it contains many effective replicate populations isolated by minimal migration, a scenario that ^59^ shows reduces autocorrelations, consistent with ^6^, who analyzed two parallel ancient human time transects and found no genome-wide signal of linked selection. Supporting these arguments, our simulations of purifying selection did not show enrichment of GWAS signals and HAF score as we observe in real data (Figure 1b,d, Extended Data Figure 4, Supplementary Information section 2).

It will be of interest to apply similar approaches to ancient DNA time series over longer times and to other world regions. This would allow more generalizable insights by identifying which patterns of selection are shared and which are distinctive to Holocene West Eurasia.

## Methods

### Testing for selection while correcting for population structure

**Extended Data Figure 14:**
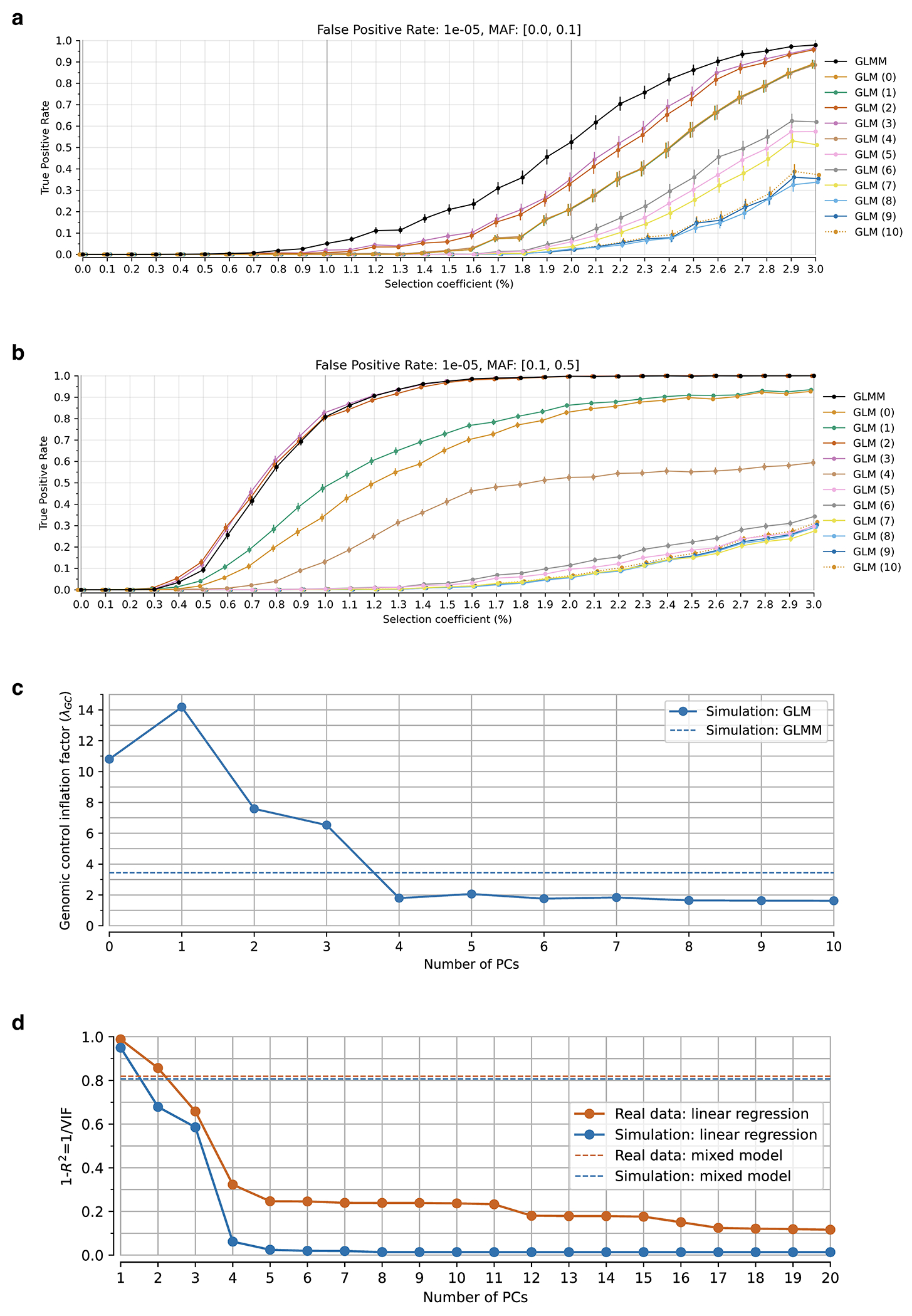
Comparison of GLMM and GLM performance and the impact of principal component covariates on inflation and power. **(a, b)** Comparing the performance of GLMM and a generalized linear model (GLM) with PC covariates. True positive rate at a false positive rate of 10^−5^ versus selection coefficient for MAF ranges (**a**) [0, 0.1] and (**b**) [0.1, 0.5]. The black line shows the GLMM model, and the colored lines show the GLM model (with the number of PCs in parentheses). Selection coefficients range from 0.0 to 0.03 in 0.001 increments, with ~4,000 simulation replicates per selection coefficient. Error bars indicate 95% confidence intervals around estimated true positive rate. **(c, d)** Inclusion of additional principal components as covariates inflates variance and reduces power due to collinearity with time. **(c)** Genomic control inflation factor (λ_GC_) for the GLM and GLMM in simulation. λ_GC_ is defined as median(Z^2^)/0.455, which quantifies the median inflation of the Z^2^ statistics relative to a chi-square distribution with one degree of freedom. Various factors, including polygenic signal, confounding due to population structure, and SNP assessment using a modern reference panel, may contribute to this inflation (see section “SNP ascertainment using a modern reference panel”). **(d)** Loss of power due to collinearity between time and principal components. The y-axis shows 1 − R^2^ (equal to 1/VIF – variance inflation factor), a proxy for statistical power—lower values indicate greater variance inflation and reduced power. Each dot represents a linear regression with time as the response and the number of PCs (x-axis) as covariates. Both simulated and real data reveal that including more PCs increases collinearity with time, diminishing power. In simulations, power drops sharply after three PCs, while in real data the decline is more gradual due to complex structure not captured in the simulation (so continued correction adds value). R^2^ is the coefficient of determination, indicating the proportion of variance in time explained by the PCs. VIF quantifies the inflation of the variance of the estimated coefficient due to collinearity with time. The dashed lines indicate $1-R_{c}^{2}$, where $R_{c}^{2}$ is the conditional *R*^*2*^ (see ^270^). $R_{c}^{2}$ is the proportion of variance in time explained by the fixed effects and the genetic random effect in a linear mixed model, where time is the response variable. The model includes an intercept as the fixed effect and a random effect with a GRM as its covariance structure. In this model, since the only fixed effect is the intercept, which contributes no variance, $R_{c}^{2}$ effectively reflects the variance explained by the genetic random effect. It measures the model’s goodness of fit and is analogous to *R*^*2*^ in ordinary linear regression.

We used a generalized linear mixed model (GLMM) approach to correct for population structure, a major confounder in scans for significant changes in frequency over time especially as migration and population mixture have been common in almost all parts of the world. Previous studies have corrected for structure in ancient DNA time transects by modeling the population history and estimating mixture proportions, which works optimally only if there are data from the true source population, which is rarely the case. It is tempting to use an unsupervised approach like Principal Components (PC) to address population structure. However, after experimentation we found this is not effective as PCs are correlated with sample dates which creates collinearity with the quantity we are most interested in (the time-varying component), inflating the empirically estimated variance and reducing power (Extended Data Figure 14).

The mixed model approach, which is often deployed in the context of genetic association studies and occasionally applied in selection studies in modern populations^61^ to address similar challenges^62^, offers a way to address these issues by combining structured data in an unsupervised manner and estimating fewer parameters over a wider span of time which results in greater power compared to employing separate regression analyses for each population or comparing the estimated means from different groups. Our simulations show that under simplifying assumptions, a GLMM is more powerful in controlling for population structure and detecting change in allele frequency compared to a generalized linear model using the top principal components (PC) as covariates (Extended Data Figure 14). Thus, the method has advantages despite the fact that the model fitted by the GLMM makes the unrealistic assumption of constant selective pressure across space and time, and is thus susceptible to missing real signals of fluctuating selection like *TYK2* rs34536443.

We use our GLMM to fit a linear time-varying component to the logit (log-odds) transformation of allele frequency at each position in the genome, and then to test if there is evidence for a consistent trend in allele frequency change over time for all populations. We search for evidence of such a trend beyond the prediction based on population structure and associated genetic drift relating sampled individuals in space and time as measured by the covariance of genotypes over all the individuals, known as the Genetic Relationship Matrix (GRM). In our GLMM, the response variable for each tested allele *j* is the allele count. The allele counts for an individual *i* are drawn from a binomial distribution *B*(2, *p_ij_*), where 2 is the number of chromosomes each person carries at each position, and *p_ij_* is the unknown frequency of allele *j* in the population in which the tested individual *i* lives. A logit link function allows the frequency *p_ij_* to be modeled as a linear combination of covariates. This is a generalization of the Logistic Mixed Model where the response variable is binary:

(1) $$ln\left(\frac{p_{ij}}{1-p_{ij}}\right)=\alpha_{j}+s_{j}t_{i}+MVN\left(0,{\sigma_{j}}^{2}K\right),$$

The logit function, $ln\left(\frac{p_{ij}}{1-p_{ij}}\right)$, transforms allele frequency so its expected change per generation is proportional to the selection coefficient $s_{j}$ (regardless of *p_ij_*)^63,64^. $\alpha_{j}$ is a constant related to the average logit transformation of allele frequency in sampled individuals at time *t*=0 today. $s_{j}$ is the per-generation selection strength at the allele, assumed constant over time and space during the period of our time transect; our test for selection is simply a test for whether the equation fits significantly better if $s_{j}$ is non-zero than if it is zero. $t_{i}$ is the negative sampling date in the past, in units of twice the generation interval^63,64^ (assuming 29 years per generation). $g_{ij}$ is a random effect, an error term capturing individual-specific variability not explained by fixed effects ($\alpha_{j}+s_{j}t_{i}$). It differs from the error term in a Generalized Linear Model, which is independently and identically distributed following a normal distribution. In our GLMM, the error term is drawn from the vector $g_{j}\sim MVN\left(0,{\sigma_{j}}^{2}K\right)$, following a multivariate normal distribution, where **K** is the covariance matrix structure (the GRM), the empirically observed relatedness of all individuals to each other, and ${\sigma_{j}}^{2}$ measures the drift at that variant, constrained to be no smaller than the genome-wide expectation conditional on minor allele frequency.

$s_{j}$, ${\sigma_{j}}^{2}$ and $\alpha_{j}$ are independently estimated for each of 9.7 million variants. Refitting ${\sigma_{j}}^{2}$ and $\alpha_{j}$ at each analyzed site makes the method robust to false-positives due to processes that vary across SNPs such as degree of background selection which increases the effective amount of random genetic drift or variation in minor allele frequency (MAF). These nuisance random effects are soaked up by allowing ${\sigma_{j}}^{2}$ and $\alpha_{j}$ to vary, allowing us to test for a time-dependent influence on allele frequency fluctuations $s_{j}$ beyond what can be explained by the GRM. We placed a minimum value on ${\sigma_{j}}^{2}$ constrained by genome-wide estimates conditional on minor allele frequency, as we were concerned that if we did not do this the method might assume an unreasonably low ${\sigma_{j}}^{2}$ and ascribe due too much allele frequency change to the $s_{j}$ term, again contributing to false-positives. Our test for a non-zero $s_{j}$ is thus a test for selection above and beyond what could be explained not just by structure but also other non-time-dependent processes. The penalty we pay for estimating variance components at millions of SNPs—in contrast to the constant variance component assumption used in mixed model analysis in Genome-Wide Association Studies (GWAS)^62^—is computational load. We grouped individuals with similar ancestry and dates into 2000 clusters (Supplementary Information section 8); at this resolution, our analysis required ~130,000 CPU hours.

Using the GLMM, we obtain a point estimate for the selection coefficient at each variant and its standard error, and a Z-score for the number of standard errors this is from zero, a naïve test for selection. In practice, the statistic needs recalibration as it is inflated due to unmodeled features of the data, so we empirically assess significance from enrichment of signals in independent GWAS (Supplementary Information section 3).

### Fitting the generalized linear mixed model (GLMM)

We developed PQLseqPy, a faster implementation of PQLseq^65^ for fitting the GLMM to count data. Despite an 82-fold speed increase, running a GLMM on ~20,000 individuals for ~9.7 million variants was infeasible (~6500 CPU years). To make analysis tractable, we grouped individuals into clusters with similar ancestry and coming from similar times. For the single variant selection tests, we restricted to 13,936 ancient individuals who were unrelated up to the second degree to make computation tractable (for the polygenic tests we included related individuals).

To identify the T = 2000 clusters we analyze, we required there to be a maximum date gap G = 500 years between any two individuals in each cluster. We initialized the interval I = (l=2, r=T) with midpoint m. We applied hierarchical clustering on the top 30 principal components (PCs) using the sklearn.cluster.AgglomerativeClustering function in Python with default parameters and n_clusters = m. For each of the S clusters from the previous step, we performed hierarchical clustering on the dates with distance_threshold = G and n_clusters = None. If the resulting number of clusters was larger than T + 1, we repeated the process with I = (l, m). If it was less than T-1, we updated I = (m, r). We repeated these steps until the final number of clusters was within T-1 to T+1, or l = r. Across 2000 clusters, individuals per cluster has a first quartile of 2, a median of 4, a third quartile of 10, and a maximum of 328.

We used the GLMM model in Equation 1. However, the cluster can include more than one individual. The allele counts for each cluster *i* are drawn from a binomial distribution *B(2n_i_, p_ij_)*, where *n_i_* is the number of diploid individuals in the cluster, and *p_ij_* unknown frequency of allele *j* in the population where individuals in cluster *i* reside.

### Proportion of variance explained by directional selection

The proportion of variance in allele frequency on the logit scale for each SNP *j* is:

(2) $$\text{Proportion of variance for SNP j}=\frac{s_{j}^{2}\cdot var\left(t\right)}{s_{j}^{2}\cdot var\left(t\right)+\sigma_{j}^{2}}$$

We use 1000 independent SNPs, randomly selected across the genome with pairwise LD (r^2^) less than 0.05, to estimate that directional selection explains an average of 2.16% of the variance in allele frequency, with a standard error of 0.11% based on jackknife estimation. The GLMM used for this analysis is based on the full sample size of unrelated individuals, rather than clustering individuals according to their ancestry and date.

### Covariance structure for the GLMM

The covariance structure matrix **K** for clusters m and n is defined as:

(3) $$K_{mn}=\frac{1}{N_{m}N_{n}}\sum_{i\in c_{m}}\sum_{j\in c_{n}}A_{ij}$$

Where $c_{m}$ is the set of individuals in cluster *m*, $N_{m}$ is the number of individuals in cluster m, and $A_{ij}$ is the genetic relationship matrix (GRM) between individuals i and j and defined as^17^:

(4) $$A_{ij}=\frac{1}{M}\sum_{k=1}^{M}\frac{{(G}_{ik}-2f_{k})(G_{jk}-2f_{k})}{2f_{k}\left(1-f_{k}\right)}$$

Here $G_{ik}$ is the genotype for SNP k of individual i, $f_{k}$ is the allele frequency of SNP k across all individuals, and M is the number of SNPs. We create a GRM using all autosomal SNPs passing quality control with MAF>0 across all individuals, and apply a leave-one-chromosome-out (LOCO) scheme to prevent proximal contamination^66,67^, creating a separate GRM for each chromosome.

### Polygenic score computation

The polygenic score (PGS) we analyze is a weighted average of genotypes for M independent variants:

(5) $$PGS_{i}=\sum_{j=1}^{M}w_{j}G_{ij}$$

Here, $G_{ij}$ is the genotype for SNP *j* of individual *i* and $w_{j}$ is the SNP weight. We generated four variations of the PGS score by using the GWAS effect values (*β_i_*) or only the sign of the effects (sign(*β_i_*)) as weights, and by including or excluding the HLA region (the results quoted in the main text exclude the HLA region). For each phenotype, we generated an independent set of SNPs using a two-step clumping and thresholding process. To avoid ascertainment bias^68^, SNP selection and effect size estimation for polygenic score calculation were both derived from the same GWAS, independent of other GWAS or selection signals. Initially, we clumped SNPs with PLINK using a P-value <10^−3^, r^2^<0.05, and a 500 kb window. Then, we selected the SNP with the smallest P-value as the index SNP, removed SNPs with D′ > 0.2 within 500 kb, and repeated until no SNP remained. Consequently, all remaining SNPs had P<0.001, and if two SNPs were within 500 kb, their r^2^ < 0.05 and D′ < 0.2. To minimize residual population structure, we used a linear mixed model (LMM):

(6) $$y_{i}=\alpha +t_{i}\gamma +g_{i}+e_{i}$$

Here, $y_{i}$ is the polygenic score of the sample i, centered at zero and scaled by the standard error of PGS of the modern samples to make it interpretable as the strength of polygenic selection and comparable across different traits^21^; $t_{i}$ is the date scaled down by −10,000 (so it is in units of ten millennia); α is the intercept; $g\sim MVN\left(0,\sigma_{g}^{2}K\right)$ is a vector of random effects where the covariance structure matrix **K** is the genetic relationship matrix; and $e=MVN\left(0,\sigma_{e}^{2}I\right)$ is a vector of residual errors where **I** is the identity matrix. The coefficient γ is the change of the polygenic score over 10,000 years in unit of standard deviation from the zero-centered PGS of the modern samples. We used the coefficient γ as a proxy for directional polygenic selection.

### Fitting the linear mixed model (LMM)

We used GEMMA (v0.98.5)^69^ to fit the LMM and estimate the polygenic selection coefficient $\left(\gamma \right)$. The running time was tractable, so we did not apply the clustering algorithm used in the GLMM analysis, and also included related individuals allowing the sample size of ancient individuals to increase from 13,936 for our single-allele tests to 15,836 to polygenic tests. We used the genetic relationship matrix as the covariance structure matrix **K**. Here, PGS is calculated over all autosomes, and so we could not use the LOCO approach from single-variant GLMM to avoid influence from neighboring positions in LD. Instead, we used 112,683 high-quality, independent SNPs generated by the ‘indep-pairwise 1000 1 0.05’ option of PLINK2 to calculate a GRM, using this as a covariance structure in the LMM to handle population structure and reduce influence from sites whose alleles are correlated to each other.

### Adjusting for residual inflation in directional polygenic analysis

To adjust for residual inflation in the estimated $Z_{\gamma }$ for each trait, we carried out 100 randomizations for each trait of interest, using the same SNPs employed for calculating the PGS of that trait and randomly assigning a weight of +1 or −1 to each SNP for each simulation. The simulated PGS is not expected to show a signal of polygenic selection, as the weights are randomly flipped and should cancel for polygenic traits. To carry out a valid test for whether a signal of directional selection is truly polygenic—that is, it is not driven by one or a few SNPs under strong selection—for each trait, we define an inflation factor by calculating the ratio of the median $Z_{\gamma }^{2}$ for the simulation to the median of the chi-square distribution with 1 degree of freedom (0.455). This correction factor is constrained to be at least 1 and at most the across-trait median of 3.16.

### Analyzing correlation between trait effect sizes and selection coefficients

We used LD score regression (LDSC) version 1.0.1^40,49,70^ to estimate the genetic correlation between trait effect sizes and selection coefficients (s). We used the pre-calculated LD scores computed with individuals of European ancestry from the 1000 Genomes Project, which are provided with the LDSC software. To compute trans-ethnic genetic correlation, we used the S-LDXR software^71^. We used the pre-calculated reference files for European and East Asian populations that are provided with this software.

### Studying heritability enrichment and computing standardized effect size ($\tau^{*}$)

We used stratified LD score regression (S-LDSC)^49^ to estimate the contribution of each annotation to the heritability of polygenic traits. We combined the set of annotations of interest with the baseline-LD model (v2.2), which includes 97 annotations modeling minor allele frequency (MAF), linkage disequilibrium (LD), and functional architectures including coding regions, promoters, enhancers, and conserved elements^49,72,73^. We used heritability enrichment to quantify the effects of the annotation. It is defined as the proportion of heritability explained by SNPs in the annotation divided by the proportion of SNPs in the annotation. The standardized effect size ($\tau^{*}$) measures the effects unique to the focal annotation after conditioning on all the other annotations in the baseline-LD model^67^.

### Forward-in-time simulations of selection in the context of European history

We carried out forward-in-time simulations using SLiM v4.0.1^74^ to model European history, starting with the semi-realistic demographic model of Irving-Pease et al. 2024^4^. We divided the genome into coding, functional noncoding, and neutral regions. We simulated both purifying selection (selection against newly arising deleterious alleles), and stabilizing selection (selection against variation), as these are two major processes known to shape patterns of human genetic variation and are not forms of directional selection (defined for our purposes as sustained rises in frequency of alleles in a direction that increases the fitness in carriers). We were concerned that our test could artifactually be detecting signals at alleles affected by these non-directional selection processes, and to explore this we simulated a variety of scenarios. Each model included an experimental condition with both directional and non-directional selection processes, and a corresponding negative control that differed only by the absence of directional selection. Detailed information on the simulations and our analysis of them is provided in Supplementary Information section 2.

### GRM-matched simulations based on real data

For the purposes of Figure 2b and Extended Data Figure 1c where we wished simulate the genotypes of individuals for a variant with a selection coefficient *s_j_*, we used a random sample drawn from a Gaussian distribution with a covariance matrix of ${\sigma_{j}}^{2}K$. We estimated the genetic relationship matrix A using real data, and randomly selected ${\sigma_{j}}^{2}$ from an empirical distribution. We employed Equation 1 to simulate different selection coefficients and determined the initial allele frequency by drawing from an empirical distribution of allele frequencies in modern individuals. We used this value as a constraint to define the constant $\alpha_{j}$. To sample genotypes, we drew from a binomial distribution, with the probability of the alternative allele calculated using the standard logistic function applied to both sides of equation 1. All other simulations we report in this study are based on the SLiM framework.

### Sources of data for 15,836 ancient individuals

We restricted to 15,836 ancient individuals of genetically West Eurasian-related ancestry living between longitude 50W and 120E and latitude greater than 24N (Supplementary Table 1, Supplementary Information section 1). For 5,820 ancient individuals, the sequences we analyze are published^2,15,31,37,48,75–246^ and are reanalyzed here. For 296 ancient individuals, we publish shotgun sequencing data from samples for which in-solution enrichment data from the same ancient DNA samples, extracts, or libraries was previously published. The present study serves as the formal report of these new sequences, and reanalysis of the data presented here should cite both the present study and the study that originally reported data from these samples. Supplementary Table 2 lists these samples along with newly reported shotgun data for an additional 71 anonymized newly reported individuals (for a total of 367 newly reported shotgun genomes which have a median of 4.89-fold coverage and of which 43 have at least 20-fold coverage).

For 223 ancient individuals, we publish higher coverage in-solution enrichment data based on additional extracts, libraries and sometimes recaptures of libraries for which smaller amounts of in-solution enrichment data from the same samples were previous published, obtained by adding data from 517 newly reported ancient DNA libraries (Supplementary Table 3). The present study serves as the formal report of these merges of previously published data with the newly generated data. Reanalysis of the data presented here should cite both the present study and the study that originally reported data from these individuals.

For 9,426 never-before-reported ancient individuals obtained by in-solution enrichment of 17,880 newly reported ancient DNA libraries (Supplementary Table 3) and shotgun sequencing of an additional 71 newly reported ancient DNA libraries (Supplementary Table 2), we release raw ancient DNA data with permission of sample custodians. The individuals are anonymized, with the only information provided being that used in our analysis: the full genetic data, and point estimates of their dates and broad geographic categorization into five regions of West Eurasia.

### Sources of data for contemporary individuals

We analyzed data from 6,438 contemporary individuals, comprising 5,935 from the UK Biobank^247^, and 503 from the 1000 Genomes Project^248^.

For the UK Biobank data, we selected individuals genotyped on the UK Biobank Axiom array, excluding those genotyped on the UK BiLEVE array to minimize batch effects. To ensure broad representation across Western Eurasia, we subsampled the UK Biobank, limiting the selection to at most 300 people per “country of birth” within Western Eurasia, focusing on countries with ancient DNA in this study. This yielded 6,088 individuals.

We then performed an outlier removal step. Using their coordinates on the top 20 principal components derived from the full UK Biobank dataset, we standardized the scores and calculated Mahalanobis distances. Assuming the squared Mahalanobis distance follows a χ^2^ distribution with 20 degrees of freedom, we excluded individuals with P-values below the Bonferroni threshold of 8.2x10^−6^. This outlier removal step yielded a final set of 5,935 individuals from 58 countries, with a median of 55 and a mean of 102 per country.

### Ancient DNA data generation

The great majority of wet laboratory work was performed in the ancient DNA laboratory at Harvard Medical School in Boston, USA, following established protocols that evolved over time from mostly manual processing (sample preparation, DNA extraction with silica columns^249,250^ and partial UDG-treated double-stranded library preparation^251,252^; capture was automated using a Perkin Elmer EP3 or Agilent Bravo NGS Workstations^2,188,253^) to mostly automated processing (DNA extraction^254^, double- and single-stranded library preparation^255^, capture, pooling for sequencing). New libraries (if not deeply shotgun sequenced) were enriched with the Twist Ancient DNA panel^242^, whereas older libraries were enriched with the 1240k reagent (or its predecessor, 390k and 840k). We sequenced on an Illumina NextSeq500 instrument until 2019, when we switched to an Illumina HiSeq X10 instrument, and finally to an Illumina NovaSeq X instrument in 2022. Archaeologists or collaborators from other ancient DNA laboratories in some cases provided sample powder, DNA extracts, or libraries. Supplementary Table 3 provides summary statistics based on in-solution enrichment for 18397 ancient DNA libraries for which we report new capture ata. Supplementary Table 2 provides summary statistics for 369 ancient DNA libraries from 367 individuals for which we report new shotgun sequencing data.

### Ancient DNA bioinformatic processing

Most of the newly reported data come from sequencing the products of in-solution enrichment targeting a set of more than one million known polymorphisms^188,242^. In-solution enrichment extracts more information per invested sequence by enriching molecules to overlap sites that are polymorphic in humans (which also helps to greatly reduce the proportion of non-endogenous bacterial/microbial sources that colonized the samples post-mortem). The great majority of ancient DNA libraries we analyzed are marked with identification tags (barcodes and indices) before sequencing in pools. We merged paired-end sequences, requiring that there is no more than one mismatch in the overlap between paired sequences where the base quality is at least 20 or three mismatches if the base quality is <20. We did not analyze sequences we could not merge. We stripped adapters and identification tags to prepare molecules for alignment. We used a custom toolkit (ADNA-Tools v2.1.0) for all these steps. We aligned merged sequences to the hg19 version of the human reference genome with decoy sequences (hs37d5) using the single-ended aligner, BWA SAMSE v.0.7.15^256^ with typical ancient DNA alignment parameters −n 0.01 −o 2 and −l 16500 which disables pre-alignment seeding. We marked duplicate reads using Picard MarkDuplicates v.2.17.10^257^. In addition, we mapped merged sequences to the mitochondrial DNA Reconstructed Sapiens Reference Sequence (RSRS)^258^, which enables mitochondrial-specific metrics. Our bioinformatic processing produces data and key metrics, including estimates of authenticity based on elevated damage rates at the end of sequences (indicative of ancient DNA), contamination rates, and endogenous rates. For the shotgun sequencing of a subset of libraries with a high proportion of human DNA to generate coverage throughout the genome, we processed data in the same way as for the in-solution enrichment experiments.

### Imputation

To carry out imputation, we used as input either data from ancient individuals (mapped sequences) or modern individuals (SNP array genotypes), and then used allelic correlation patterns in a haplotype reference panel^248,259^ to predict genotypes at millions of sites.

In detail, for each sample we used bcftools mpileup (v1.13)^260^ to generate genotype likelihoods for all variants (SNPs and indels). We used the high coverage (30x) 1000 Genomes Project^248^ phase 3 sequences as the reference panel and converted the assembly version to GRCh37/hg19 using CrossMap (v0.5.2)^261^. We restricted to 2504 unrelated samples and biallelic variants that pass all the quality control filters reported by gnomAD (v2.1.1)^262^. We used GLIMPSE (v1.0.0)^16^ with the reference panel to impute and phase each sample individually. Due to higher reference bias for indels, we ignored their genotype likelihood, set them to missing, and passed this to GLIMPSE to impute all biallelic autosomal SNPs and indels based on the genotype likelihood of SNPs and haplotype information for both SNPs and indels in the reference panel. This means we only used reference panel data to impute indels even when we had sequences overlapping the indels. After imputation, we added the genotype caller information of all variants (SNPs and indels) to the final bcf file.

To minimize discrepancies between imputation of ancient DNA and UK Biobank data, we re-imputed the UK Biobank genotyping data. We used Affymetrix confidence files to simulate genotype likelihoods and processed these through the same imputation pipeline employed for ancient DNA.

### Sample quality control

For each imputed sample, we define imputation quality score as $IQS=mean\left(GP_{1}|GT=1\right),$ where GT is the most likely genotype based on the imputed genotype posterior $GP=\left(GP_{0},GP_{1},GP_{2}\right)$ and $\sum_{i=0}^{2}GP_{i}=1$. We only kept samples with high imputation quality score IQS>0.9. We used KING to detect duplicates and related samples up to the second degree. We prioritized samples by their IQS and dropped relatives up to the second degree until there were no two that were second-degree related or closer. We also fit a linear regression model to the top 100 PCs as explanatory variables, and used the reported date of samples as the response variable to remove outliers where reported and predicted date were very different. Full details of this procedure are presented in Supplementary Information section 1.

### Variant quality control

The data analyzed in this study come from multiple sources and sequencing technologies: imputed ancient DNA sequences (shotgun sequences and enrichment for more than one million SNPs), European ancestry individuals largely from the 1000 Genomes Project, and imputed individuals of Western Eurasian ancestry from the UK Biobank genotyped using the UK Biobank Axiom Array. We applied variant quality control in a three step procedure. Initially, we applied brute-force filtering to compute principal components, allowing for the identification of ancestry-matching samples across datasets with similar allele frequencies. We filtered out variants if their allele frequencies differed strongly between sample sets, with the goal of minimizing batch effects from combining samples from different sources. Finally, we excluded variants whose estimated selection coefficients were sensitive to data batches. This resulted in 9,739,624 variants, including 8,074,573 SNPs and 1,665,051 indels, passing the final variant QC out of 52,382,872 imputed variants. The step-by-step variant quality control process is detailed in Supplementary Information section 1.

### Allele frequency trajectories

We computed allele frequency trajectories using all individuals in the time series. We used a moving average sliding window, with a window size of 1000 years and a step size of 100. We used a binomial likelihood function to estimate the mean, confidence intervals, and standard error. We smoothed the mean and standard error using the GaussianProcessRegressor function from the Scikit-learn library in Python. We parameterized this function with alpha = 1e-4 and a 1*RationalQuadratic kernel, with length_scale_bounds set to (10, 1e6). We clipped the resulting values to remain within the range of 0 and 1.

### Assembly of GWAS data to which we correlated selection coefficients

We analyzed GWAS of 452 largely quantitative phenotypes from the UK Biobank^263^ that had the flag phenotype_qc_EUR set as PASS (the high-quality subset of 6951 UK Biobank GWAS). We added to this 107 independent GWAS studies^264,265^ mostly of complex diseases, alongside 2 brain volume GWAS from ^56^ and 2 GWAS from ^55^ for cognitive and noncognitive aspects of educational attainment. These 563=452+107+2+2 GWAS of largely unrelated people of European ancestry are the ones we refer to in the main text when discussing our primary scans for polygenic selection. For the trans-ethnic analysis, we analyzed an additional 31 GWAS in East Asians: 30 phenotypes from the Biobank of Japan (BBJ)^266^ and the GWAS summary statistics from the study of years of schooling GWAS by ^267^. For family-based analysis, we analyzed 102 GWAS for 34 traits compiled in ^54^; for each trait there were three GWAS: unrelated people, direct effect, and indirect effect. Summary statistics for all our polygenic tests of selection for all of these 696=563+30+1+102 GWAS are presented in Supplementary Table 5.

## Extended Data

**Extended Data Table 1.** Re-evaluation of signals from five scans for selection in Holocene West Eurasia. Significance of selection according to our analysis for loci identified in five previous scans for selection in Holocene West Eurasia (all but Field et al. are ancient DNA-based scans). The less stringent P-value thresholds are 10^−5^ for Field et al. 2016 and 0.05 for Kerner et al. 2023. The cumulative number of non-HLA signals identified as genome-wide significant and confirmed in our re-analysis with a posterior probability of selection *π*>99% is 18 (4% of the 410 non-HLA loci with *π*>99%). Of these, 8 were found in Mathieson et al., Field et al. added 0, Le et al. added 3, Kerner et al. added 0, and Irving-Pease et al. added 7.

	Genome wide significant loci				Less stringent threshold			
	Total	Pass QC	*π*>99%	*π*>50%	Total	Pass QC	*π*>99%	*π*>50%
Mathieson et al. 2015	12	11	10	10				
Field et al. 2016	3	3	1	3	37	36	3	11
Le et al. 2022	24	22	9	10				
Kerner et al. 2023	3	3	3	3	139	125	14	23
Irving-Pease et al. 2024	21	21	14	17				

## Supplementary Material

SIGuide

Supplementary-Information-24-2-26-final

## Data Availability

Aligned sequences for the newly reported data from 10016 ancient individuals are available through the European Nucleotide Archive under an accession number PRJEB106907. This includes complete genetic data for all newly reported individuals alongside point estimates of their dates and geographic categorization into five regions of West Eurasia, which is the full data used in the present study (Supplementary Tables 1–3). Our release of these data without restrictions to enable studies of natural selection has written approval from third-party sample custodians. Please contact corresponding author DR for any questions regarding metadata not used in this study—such as skeletal codes, latitudes and longitudes, site names, site descriptions, physical anthropology, and cultural context—which will be reported in future work that should be the references for studies of population history and archaeology (as a backup in case these studies are not published in reasonable time, we have deposited a version of the Supplementary Tables with the full archaeological metadata for all these individuals to Harvard Dataverse at https://doi.org/10.7910/DVN/7RVV9N with a 15 year embargo so it will go public in the year 2041). Imputed genomes for ancient individuals are also available at the Harvard Dataverse: https://doi.org/10.7910/DVN/7RVV9N. High-coverage (30x) Phase 3 sequences from the 1000 Genomes Project were downloaded from ftp.1000genomes.ebi.ac.uk/vol1/ftp/data_collections/1000G_2504_high_coverage/working/20201028_3202_phased. Genome coordinates were converted to GRCh37/hg19 using CrossMap v0.5.2. The processed 1000 Genomes data used as the imputation reference panel and for downstream analyses are publicly available at the Harvard Dataverse: https://doi.org/10.7910/DVN/7RVV9N. Individual-level data from the UK Biobank used in this study were obtained from the UK Biobank Resource and are available to eligible researchers upon application through the UK Biobank Access Management System, in accordance with UK Biobank policies.

## Software and code availability

An interactive web application for this study is available at http://reich-ages.rc.hms.harvard.edu. The PQLseqPy software is available from https://github.com/mokar2001/PQLseqPy. A permanently archived version of this package is available at Harvard Dataverse: https://doi.org/10.7910/DVN/7RVV9N. All code for the forward-in-time SLiM simulations is available at https://github.com/AnnabelPerry/slim-selection-simulations.
