# Supplementary material for: Ancient DNA reveals pervasive directional selection across West Eurasia
Source: Nature. Author manuscript; Available in PMC 2026 May 21. (PMC13189228; doi:10.1038/s41586-026-10358-1)
Supplement: SIGuide [file NIHMS2168269-supplement-SIGuide.docx]

**Supplementary Information Guide**

**Ancient DNA reveals pervasive directional selection across West Eurasia**

Ali Akbari, Annabel Perry, Alison R. Barton, Mohammadreza Kariminejad, Steven Gazal, Zheng Li, Yating Zeng, Alissa Mittnik, Nick Patterson, Matthew Mah, Xiang Zhou, Alkes L. Price, Eric S. Lander, Ron Pinhasi, Nadin Rohland, Swapan Mallick_,_ and David Reich

**Supplementary Information sections**

**Supplementary Information section 1: Quality Control (QC).** Quality control procedure for variant and sample filtering. Includes Tables S1.1–S1.4 and Figures S1.1–S1.7.

**Supplementary Information section 2: Simulation of European demographic history.** Forward-in-time simulations under a West Eurasian demographic model incorporating purifying, stabilizing, and directional selection. Includes Tables S2.1 and S2.2 and Figures S2.1–S2.13.

**Supplementary Information section 3: Statistical criterion for genome-wide significance.** Evaluation of significance thresholds under a non-normal null distribution and implementation of an FDR-based framework guided by GWAS enrichment. Includes Figures S3.1 and S3.2.

**Supplementary Information section 4: HAF score analysis provides evidence for directional selection.** Assessment of haplotype-based signals supporting directional selection and their expected behavior under positive and negative selection. Includes Figure S3.1.

**Supplementary Information section 5: Allele frequency trajectory and selection coefficient over time for 479 independent loci with >99% probability of selection.** Time-resolved allele frequency dynamics and inferred selection coefficients for high-confidence selected loci. Includes Table S5.1 and Figures S5.1-S5.80.

**Supplementary Information section 6: Re-evaluation of results from previous studies.** Systematic reanalysis under our analytical framework of reported selection signals from four ancient DNA selection scans and one modern-variation scan. Includes Tables S6.1 and S6.2 and Figures S6.1-S6.29.

**Supplementary Information section 7: A new picture of selection at the major risk factor for multiple sclerosis (MS).** Focused analysis of selection dynamics at the major MS locus, including ancestry modeling and allele frequency inference. Includes Figures S7.1-S7.3.

**Supplementary Information section 8: A fast GLMM implementation – PQLseqPy.** Description of a fast GLMM implementation used for large-scale selection scans. Includes Figure S8.1.

**Supplementary Data Sets**

**Supplementary Table 1: 22274 individuals analyzed in this study (6438 modern, 15836 ancient) of which 10016 are represented by new data.** Data Source 1 is 12258 modern (n=6438) and ancient (n=5820) individuals whose previously published sequences we reanalyze. Data Sources 2-4 are individuals represented by new data. Data Source 2 is 296 individuals with previously published in-solution enrichment data for which we report and analyze whole genome shotgun data. Data Source 3 is 223 individuals with previously published data for which we report and analyze additional in-solution enrichment data. Data Source 4 is 9497 never-before-reported ancient individuals for which we report and analyze data that is anonymized except for a point estimate of the data of origin and information about broad region in West Eurasia. Available as an Excel table.

**Supplementary Table 2: 367 newly reported shotgun ancient genomes.** Most are from individuals with previously reported in-solution enrichment data (n=296); the remainder are from individuals with data reported for the first time (n=71). Available as an Excel table.

**Supplementary Table 3: 18397 ancient DNA libraries with newly reported capture data.** The majority (n=17880) are from 9426 never-before-reported ancient individuals; the rest (n=517) are from 223 individuals for which we increase data quality. Available as an Excel table.

**Supplementary Table 4: Summary statistics for selection at 9.7 million variants.** The data are provided as a tab-delimited text file, compressed using gzip. Available at Harvard Dataverse: <https://doi.org/10.7910/DVN/7RVV9N>.

**Supplementary Table 5: Summary statistics for 696 tests of polygenic selection.** The data include: 452 European GWAS from UK Biobank^263^, 107 curated European GWAS used for S-LDSC meta-analysis^264,265^, 102 family GWAS from ^54^ (three GWAS for each trait), 2 GWAS from ^56^, 2 GWAS from ^55^, 30 East Asian GWAS from Biobank Japan^266^, and one from ^267^. Available as an Excel table.
