# Supplementary material for: Ancient DNA reveals pervasive directional selection across West Eurasia
Source: Nature. Author manuscript; Available in PMC 2026 May 21. (PMC13189228; doi:10.1038/s41586-026-10358-1)
Supplement: Supplementary-Information-24-2-26-final [file NIHMS2168269-supplement-Supplementary-Information-24-2-26-final.pdf]

Table of Contents

SUPPLEMENTARY INFORMATION SECTION 1 ... **3**

QUALITY CONTROL (QC) ... 3
Variant QC: Step 1 ... *3*
Variant QC: Step 2 ... *5*
Variant QC: Step 3 ... *7*
SAMPLE QUALITY CONTROL ... 9

SUPPLEMENTARY INFORMATION SECTION 2 ... **14**

SIMULATION OF EUROPEAN DEMOGRAPHIC HISTORY ... 14
Demography ... *14*
Burn-in period ... *15*
Sampling ... *16*
Genomic architecture ... *17*
Mutations ... *18*
MODELS OF BACKGROUND SELECTION ... 19
Model 1: With stabilizing selection mechanism ... *19*
Model 2: With purifying selection mechanism ... *19*
Experimental condition 2.1: Soft sweep ... *19*
Experimental condition 2.2: Hard sweep ... *19*
Model 3: With stabilizing and purifying selection mechanisms ... *19*
SELECTION MECHANISMS ... 21
Purifying selection mechanism ... *21*
Stabilizing selection mechanism ... *21*
Directional selection mechanism ... *22*
SIMULATING GWAS ... 23
EMPIRICAL CONSISTENCY CHECKS FOR SIMULATIONS ... 24
Background selection and neutral diversity ... *24*
Mutation age and trait effect size ... *25*
Allelic effect and frequency under stabilizing selection ... *26*
Principal component analysis (PCA) ... *27*
ANALYZING THE SIMULATIONS ... 28
Enrichment of GWAS hits for variants under directional selection ... *28*
Enrichment in GWAS as an approximation for False Discovery Rate ... *28*
Power analysis of GLMM and GLM and co-linearity ... *29*
Non-normality of the null distribution in GLMM test statistics ... *34*
Older sweeps in ancestral populations ... *37*
DETECTING DIRECTIONAL POLYGENIC SELECTION ... 37
Linear mixed model on polygenic scores ... *37*
Genetic correlation between trait and selection ... *37*

SUPPLEMENTARY INFORMATION SECTION 3 ... **39**

STATISTICAL CRITERION FOR GENOME-WIDE SIGNIFICANCE ... 39
Establishing the Optimal Significance Threshold ... *39*
Controlling for family wise error rate (FWER) ... *39*
Controlling for false discovery rate (FDR) by leveraging GWAS signals ... *41*

SUPPLEMENTARY INFORMATION SECTION 4 ... **45**

HAF SCORE ANALYSIS PROVIDES EVIDENCE FOR DIRECTIONAL SELECTION ... 45
HAF score dynamic for positive and negative selection ... *45*

SUPPLEMENTARY INFORMATION SECTION 5 ... **47**

Allele frequency trajectory and selection coefficient over time for 479 independent loci with >99% probability of selection 47
SUPPLEMENTARY INFORMATION SECTION 6 129
Re-evaluation of results from previous studies 129
Overview 129
Re-evaluation of results from Mathieson et al. 2015 133
Re-evaluation of results from Field et al. 2016 135
Re-evaluation of results from Le et al. 2022 141
Re-evaluation of results from Kerner et al. 2023 145
Re-evaluation of results from Irving-Pease et al. 2024 162
SUPPLEMENTARY INFORMATION SECTION 7 169
A new picture of selection at the major risk factor for multiple sclerosis (MS) 169
qpAdm modeling 170
Inference of allele frequency in ancestral populations 170
SUPPLEMENTARY INFORMATION SECTION 8 174
A fast GLMM implementation - PQLseqPy 174
REFERENCES 176

Supplementary Information section 1

Quality Control (QC)

The data analyzed in this study come from multiple sources: imputed ancient DNA sequences (shotgun sequences and in-solution enrichment reagent), sequences of people of European ancestry from the 1000 Genomes Project, and imputed SNP array data from the UK Biobank (genotyped using the UK Biobank Axiom Array). To generate datasets useful for filtering out variants that do not have reliable genotyping properties, we used principal components to identify groups of individuals with similar ancestry across different datasets. We then filtered out variants whose allele frequencies differed significantly between sample sets to minimize batch effects due to combining samples from different sources. Variant quality control involved a three-step procedure.

Variant QC: Step 1

We performed a first step QC that restricted to the 1240k sites for which we have particularly rich genotyping data. A variant passed provisional QC if it met all of the following criteria:

1. It belongs to the 1240k SNP set on the autosomes.
2. Chi2-test(aDNA_SG, aDNA_1240k) < 5
3. Chi2-test(UKBB_UK, GBR_CEU) < chi2_thr
4. Chi2-test(UKBB_EUR, GNOMAD_EUR) < chi2_thr
5. INFO_WEA_1240k > 0.6
6. INFO_WEA_SG > 0.6

We define sample sets in Table S1.1 and apply a chi-square test (chi2-test) to compare the allele counts of each variant across three pairs of sample sets. INFO_WEA_1240k and INFO_WEA_SG are IMPUTE2's INFO scores[1] for each variant, calculated for high-quality imputed individuals from western Eurasia with 1240k and shotgun sequences, respectively. We use the Bonferroni-corrected threshold (chi2_thr = 32.03) to filter variants. We select 450 pairs of high-quality imputed sequences from ancient individuals with both shotgun (SG) and 1240k enrichment reagent data, naming them aDNA_SG and aDNA_1240k. These two sets represent different types of sequences for the same individuals, ideally having identical allele frequencies. Allowing for a small error, we choose a chi-square test threshold of 5, resulting in 939,802 SNPs for the provisional variant QC step (Figure S1.1).

Table S1.1: Sample set used in the quality control of variants.

Sample set name	Number of samples	Description
aDNA_SG	450	Shotgun sequences of ancient individuals with both shotgun and 1240k enrichment reagent data
aDNA_1240k	450	1240k sequences of ancient individuals with both shotgun and 1240k enrichment reagent data
WEA1	2703	Western Eurasian individuals in our dataset with 0<Date<1k years BP
UKBB_EUR	458,937	Allele frequency of the European subset of UKBB is presented in the af_EUR column of the variant manifest of pan-ukbb (2022-04-11).
UKBB	5935	Samples from the UK Biobank used in this study.
UKBB_UK	283	Samples from the UK Biobank used in this study with the country of birth being the UK.
GNOMAD_EUR	4299	Allele frequencies in the European subset of GNOMAD are presented in the gnomad_genomes_an_EUR column of the variant manifest of pan-ukbb (2022-04-11).
GNOMAD_NFE	15414	Allele frequencies in individuals of non-Finnish European ancestry are represented by the AF_nfe field in the INFO columns of the GNOMAD v2.1.1 publicly available variant VCF files.
1KG_ALL	2504	All the individuals from 1000 GP
1KG	503	1000 GP individuals from EUR populations
GBR_CEU	190	1000 GP individuals from GBR and CEU populations
1KG_match_UKBB	404	1KG individuals that are ancestry matched with individuals from UKBB
UKBB_match_1kg	404	UKBB individuals that are ancestry matched with individuals from 1KG
1KG_match_WEA1	261	1KG individuals that are ancestry matched with individuals from WEA1
WEA1_match_1KG	261	WEA1 individuals that are ancestry matched with individuals from 1KG
UKBB_match_WEA1	1790	UKBB individuals that are ancestry matched with individuals from WEA1
WEA1_match_UKBB	1790	WEA1 individuals that are ancestry matched with individuals from UKBB

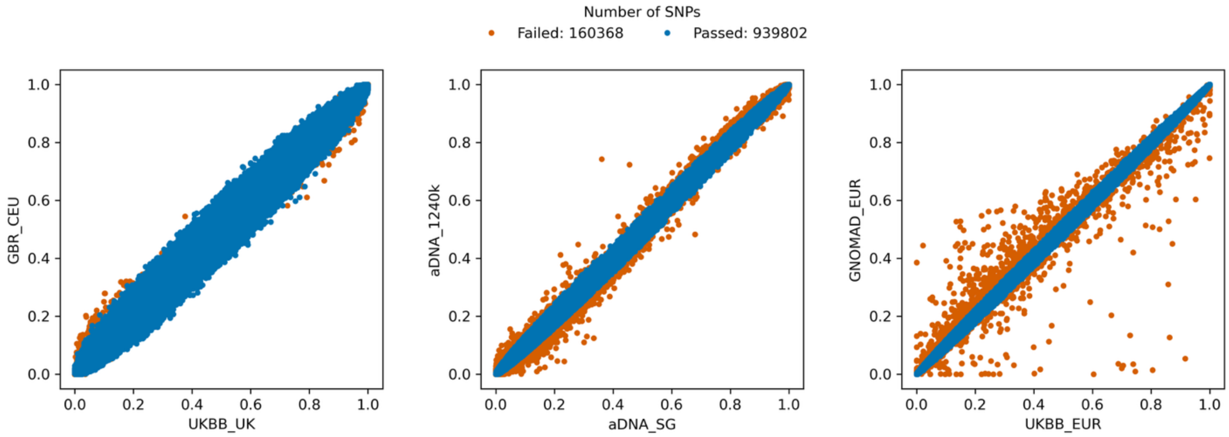

Figure S1.1: Allele frequency of 1240k SNPs in different sample sets. Each dot represents a SNP; blue dots are SNPs that passed the provisional QC, while orange ones failed. Both the x and y axes show allele frequency in the subset of individuals labeled on the corresponding axis.

Variant QC: Step 2

We next performed QC not just on the variants in the 1240k SNP set, but on all imputed variants in the 1000 Genomes Project variant set.

We used the provisional QCed SNP set and pruned them using plink2 with the --indep-pairwise 1000 1 0.2 option. Then, we used the 5,935 individuals from the UKBB in our imputed dataset restricting to the pruned SNP set, using plink2 to calculate eigenvectors and projecting all individuals to calculate principal components (PCs). We use 3 subsets (UKBB, 1KG, and WEA1) of samples to create 3 pairs of matching subsets with similar ancestry using the following procedure: For pop1 and pop2 non-overlapping subsets of individuals, we ran hierarchical clustering on their union set using the sklearn.cluster.AgglomerativeClustering package in Python on the top two PCs calculated above. The number of clusters (n_clusters) is the total number of individuals in these sets divided by 5, with a minimum of 10 clusters. Then, for each cluster, we pair individuals such that one is from pop1 and the other is from pop2 until no such pair is available. The set of all individuals that have been paired and are from pop1 is called pop1_match_pop2, and the other is called pop2_match_pop1. These two subsets consist of samples with similar ancestry based on the top two PCs. The 4 original subsets and their 5 matching pairs are detailed in Table S1.1. These matched subsets of individuals are visualized in Figure S1.2.

For individual *i* with both shotgun and 1240k sequences that are in the aDNA_SG and aDNA_1240k sets of sequences, we define Δ_i= (GT_i(Shotgun) – GT_i(1240k))/2 for each variant such that GT_i(Y) means the imputed genotype in data type Y (shotgun or 1240k) for individual *i* for the variant of interest. A variant passes final QC if:

1. Minor allele frequency in 1KG_ALL > 0.002
2. mean(|Δ|)<0.05
3. The p-value of the null hypothesis mean(Δ) = 0 is > 1e-10
4. P-chi2-normalized(GBR_CEU, GNOMAD_NFE) > 1e-10
5. P-chi2-normalized(UKBB_match_1KG, 1KG_match_UKBB) > 1e-10
6. P-chi2-normalized(WEA1_match_1KG, 1KG_match_WEA1) > 1e-10
7. P-chi2-normalized(WEA1_match_UKBB, UKBB_match_WEA1 > 1e-10
8. INFO_WEA_1240k > 0.6
9. INFO_WEA_SG > 0.6

Here, P-chi2-normalized(pop1, pop2) is the p-value of the chi-square statistic, divided by its mean over all variants, from the chi-square test between pop1 and pop2 given the allele counts. Allele frequency consistency between different pairs of sample sets is visualized in Figure S1.3.

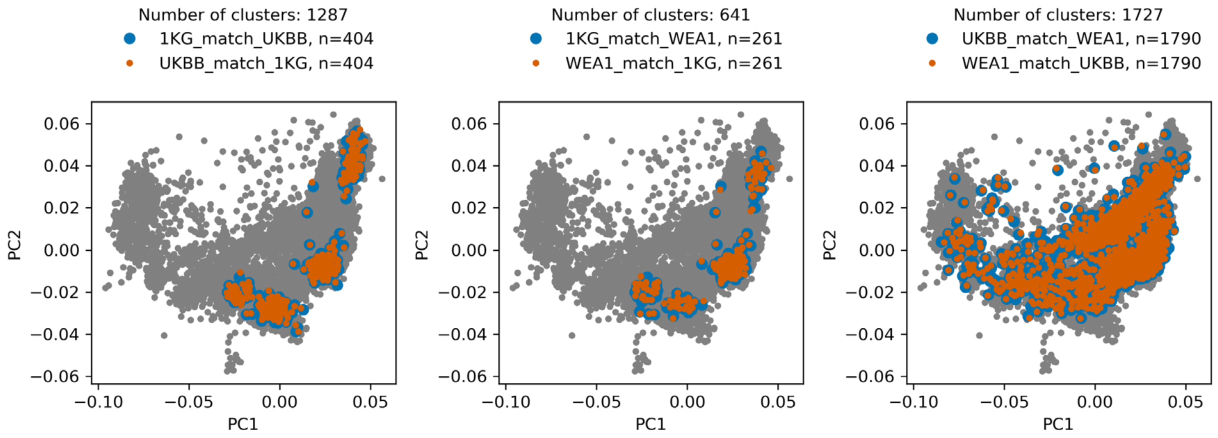

Figure S1.2: Ancestry-matched samples across different datasets. Each subplot displays ancestry-matched samples. The number of clusters is the n_clusters parameter used in the hierarchical clustering procedure, and n is the final number of ancestry-matched individuals in each sample set.

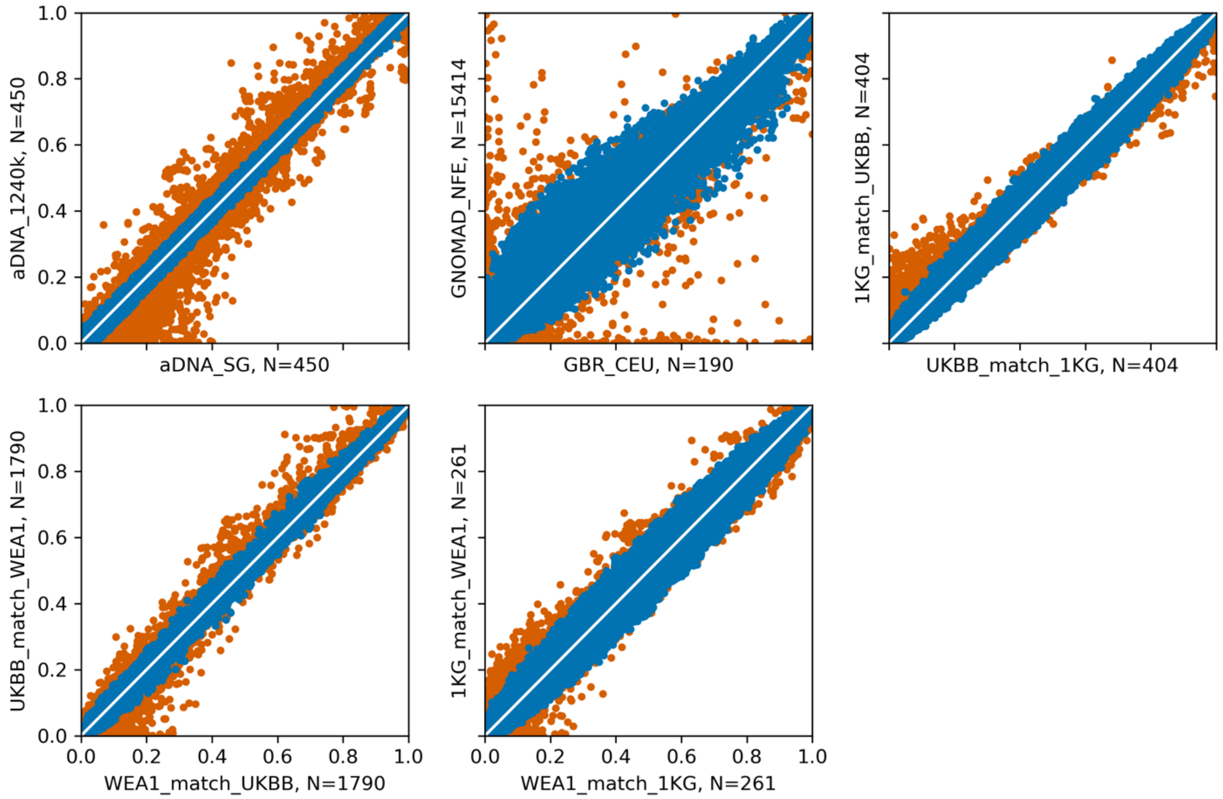

Figure S1.3: Allele frequency consistency across datasets. The figure displays 1,000,000 randomly selected variants that passed the filters (blue) and 1,000,000 that did not (orange). N represents the number of individuals in each subpopulation. Both the x and y axes show allele frequency in the subset of individuals labeled on the corresponding axis.

Variant QC: Step 3

To minimize discrepancies between the imputation of ancient DNA and UK Biobank data, we re-imputed the UK Biobank genotyping array from scratch. We utilized Affymetrix confidence files to simulate genotype likelihoods and processed these through the same imputation pipeline employed for ancient DNA. We removed variants that did not pass QC steps 1 and 2 (78.41% of all variants); the remaining 21.59% of variants passing these steps are expected to have less discrepancy across different datasets and sequencing technology (Figure S1.3).

To further reduce batch effects from imputing and merging different datasets in our selection statistics, we conducted analyses both with and without four covariates: IQS (imputation quality score), aDNA (1 for ancient individuals, 0 for modern), SG (1 for shotgun-sequenced ancient individuals, 0 otherwise), and 1kg (1 for 1000 GP samples, 0 otherwise), included as fixed effects in our GLMM. Let Z denote the Z-score of the estimated selection coefficient calculated without these covariates, and Z_0 the Z-score calculated with them. We applied weighted least squares (WLS) regression to the two Z-scores and excluded all variants with Pearson residuals greater than 2 (Figure S1.4).

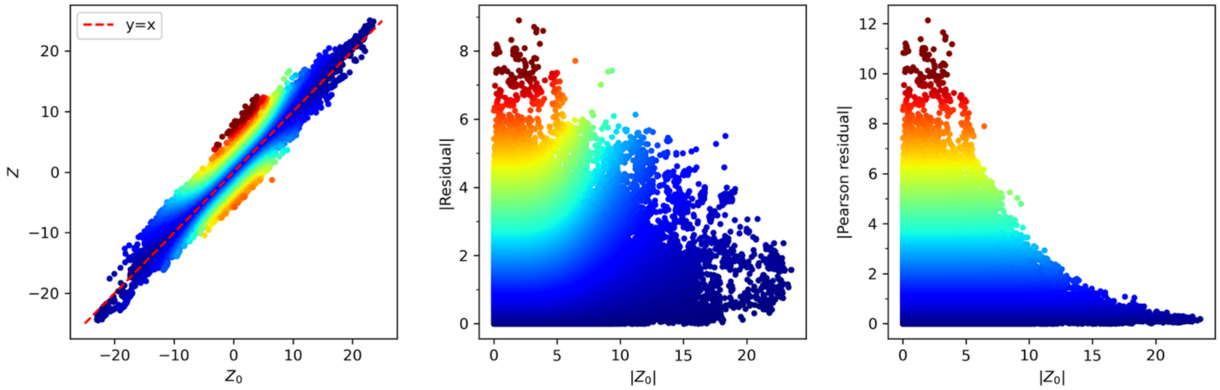

Figure S1.4: Comparison of Z-scores from GLMM, with (Z) and without (Z_0) modern samples. Outliers, which could be due to batch effects, are identified and removed using Pearson residuals from weighted least squares. Colormap is |Pearson residual|.

We fit a WLS for $Z = a + bZ_0$ with weights = $1/(c + dZ_0^4)$, such that c = 0.535, d = 2.44e-4. The weights of WLS are expected to be proportional to the variance of the response variable (Figure S1.5). We inferred weights empirically using the following approach. First, fit an Ordinary Least Square (OLS) for $Z = a + bZ_0$. $\hat{Z}$ is the OLS prediction and $Z - \hat{Z}$ is the residual. Define variable $x = |Z_0|$, $y = (Z - \hat{Z})^2$. Create 100 bins using percentiles of x and fit another OLS such that $\bar{y} = c + d\bar{x}^4$, where $\bar{x}$ and $\bar{y}$ represent mean values of x and y in each bin. We used the OLS and WLS functions of the statsmodels package in Python for this analysis.

A total of 9,739,624 variants, including 8,074,573 SNPs and 1,665,051 indels, passed all three steps of QC, representing 18.59% of the 52,382,872 imputed variants. The counts of variants

passing QC for different variant types (SNP or indel) and their presence in the PAN-UKBB, 1240K, or UK Biobank axiom array SNP sets, are summarized in Table S1.2.

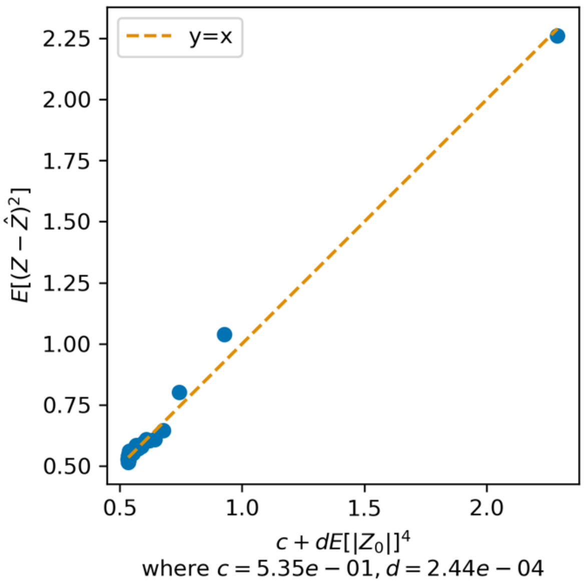

Figure S1.5: Empirical inference of weights for weighted least square (WLS).

Table S1.2: Summary of variants (SNP and indels) that passed final quality control, categorized by their presence in different variant sets.

Variant set	Category based on presence in different variant sets (1: present, 0: absent)								Variant set count
SNP	1	0	1	0	1	1	1	1	8,074,573
PAN-UKBB	1	1	1	0	1	0	1	0	8,816,746
1240k	0	0	1	0	0	0	1	1	891,918
UK Biobank Axiom	0	0	0	0	1	0	1	0	453,883
Category count	6,687,144	904,706	771,013	760,345	333,503	162,008	120,380	525	9,739,624

Sample Quality Control

To assemble a high-quality set of ancient genome-wide datasets for downstream analyses, we started from 42,291 imputed genome-wide datasets with IQS > 0.9.

We then restricted to Western Eurasian ancestry, and with no evidence of substantial contamination, as reflected in:

- Little heterogeneity of mitochondrial DNA sequences (upper bound of the 95% confidence interval (CI) for matching to the reference sequence was ≥90% based on the software contamMix[2]).
- Little heterogeneity of X chromosome sequences in genetic males (the upper bound of 95% CI for ANGSD[3] or HapConX[4] is ≤5%, or neither lower bound is ≥2%).
- The ratio of Y chromosome sequences to the sum of X and Y chromosomes sequences in capture data is <4% or ≥30%, or this is not satisfied but the upper bound of the 95% CI for ANGSD[3] or HapConX[4]<2%.
- Was not represented by damage-restricted molecules in our manually curated internal laboratory dataset (and thus tagged as likely to be contaminated).
- Had a cytosine-to-thymine error (damage) rate in the final nucleotide for individuals ≥1000 years old of ≥1% for partially UDG-treated libraries, or ≥5% for non-UDG-treated libraries (for datasets from younger individuals these numbers were 0.5% and 1% respectively).

We further restricted to datasets for which we had explicit permission for publication from archaeologist or anthropologist custodians. Specifically, we obtained 232 letters of permission from scholars who had authority to provide this and who specified with this study we could publish: anonymized raw data from never-before-reported ancient DNA samples including only data, point estimates of the dates of the individuals in years based either on archaeological or radiocarbon dates, and broad region within West Eurasia (N=North, W=West, E=East, SW=Southwest, SW=Southwest), and that they did not wish to be co-authors of the present study and would instead by co-authors of a separate publication that would be the appropriate reference for analyses of population history and full archaeological information and synthesis of archaeological and genetic data. For a number of individuals for which ancient DNA data had previously been published, we increased data amount or quality, and the present study serves as the formal report of these non-anonymized data.

We also restricted to unique individuals (we took the highest data quality representative individuals), with evidence of date uncertainty based on a large standard error on the point estimate of their date, having a very large number of detected relatives, or having genetically detected first degree relatives with very different dates.

Finally, we restricted to individuals with no evidence of cytogenetic abnormality based on analyses of coverage across the genome. Taken together, this yields 15,836 unique individuals which we used for analyses of polygenic adaptation. For analyses of single variants, we focused on a subset of 13,936 individuals not closely related to any other individuals (Table S1.3).

Table S1.3: Quality control filters and remaining sequences at each step.

Filter	Remaining sequences	Notes
Filter 1	42,291	Keep sequences with IQS > 0.9.
Filter 2	22,553	Restrict to Western Eurasian ancestry.
Filter 3	18,188	Remove sequences lacking permission, with contamination or damage, abnormal sex ratios, unreliable estimates, or poor dating.
Filter 4	18,180	Remove sequences with too many relatives (> 100).
Filter 5	18,164	Remove sequences if the dates of their first- or second-degree relatives vary widely, defined as a standard deviation > 1,000 years.
Filter 6	15,999	Remove identical sequences.
Filter 7	15,992	Remove sequences with abnormal chromosome coverages.
Filter 8	15,840	Remove date outliers by manual review and an outlier test.
Filter 9	15,836	Remove date outliers using first-degree relatives.
Filter 10	13,936	Restrict to unrelated samples.

Filter 1: Keep sequences with IQS > 0.9. For each imputed sample, we define an imputation quality score $IQS = mean(GP_1|GT = 1)$ where GT is the most likely genotype based on the imputed genotype posterior **GP** = (GP_0, GP_1, GP_2) and $GP_0 + GP_1 + GP_2=1$. We only kept samples with IQS>0.9.

Filter 2: Restrict to Western Eurasian ancestry. We calculated principal components (PCs) for individuals in the 1000 Genomes Project using 911,014 SNPs from the 1240k panel with a MAF > 0.5%. Sequences with an IQS > 0.9 were projected onto these PCs (42,291 sequences). The top 100 PCs were then clustered into 100 groups using the sklearn.cluster.AgglomerativeClustering package in Python. For each cluster, we calculated mean PC values and applied the following criteria to the cluster means to identify those with Western Eurasian ancestry: PC1 > 0, PC2 < −0.075, PC3 < 0.06, and PC4 > −0.01. Sequences belonging to clusters that did not meet these criteria were excluded. We further removed sequences located west of −50° longitude, east of 120° longitude, or south of 24° latitude, leaving 22,553 sequences (Figure S1.6).

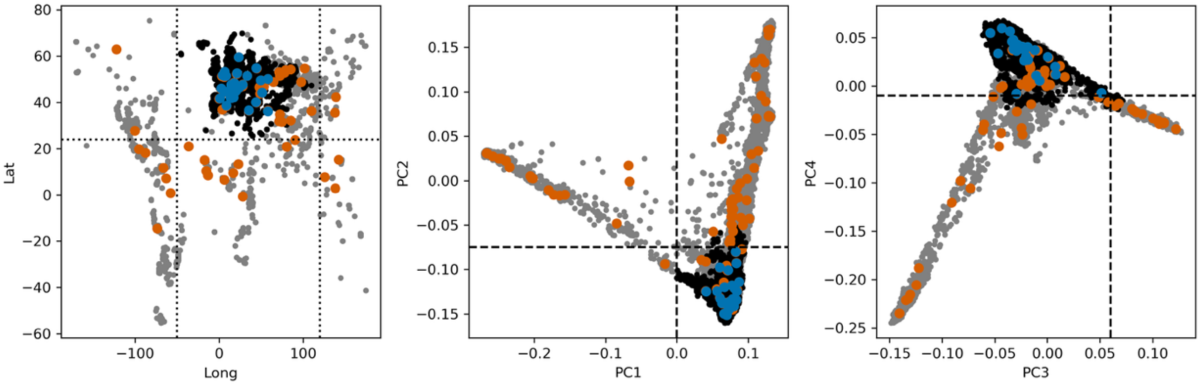

Figure S1.6: Black small circles represent included sequences, and gray small circles represent excluded sequences. Blue large circles show the mean PC values of included clusters, and orange large circles show the mean PC values of excluded clusters. Dashed lines indicate ancestry-based criteria applied to cluster means (PC1 > 0, PC2 < −0.075, PC3 < 0.06, PC4 > −0.01). Dotted lines in the left panel indicate geographic thresholds applied to sequences (longitude < −50°, longitude > 120°, latitude < 24°).

Filter 3: Remove sequences lacking permission, with contamination or damage, abnormal sex ratios, unreliable estimates, or poor dating. After applying the following seven criteria, 18,188 sequences remained.

I. Archaeologist permissions - 1663 sequences fail (1=confirmed, 0=exclude)

II. mtDNA contamMix results - 59 sequences fail (1: upper bound of 95% CI≥90% or no measurement recorded, or sample fails this metric but upper bound of ANGSD or hapConX<2%; 0: upper bound of 95CI<90% and sample not saved by upper bound of ANGSD or hapConX<2%)

III. low damage - 7 sequences fail (1: damage-rate for UDG-half≥1%, or non-UDG≥5%, or non-UDG≥1% and in last-millennium, or UDG-half>0.5% and in last-millennium, or includes or may include UDG fully-treated or pretrimmed data, or no damage measurement available; 0: otherwise)

IV. sex-ratio not expected for a male or female - 50 sequences fail (1: Y/(X+Y) ratio in capture is <4% or ≥30%, or this is not satisfied but saved by the upper bound of the 95% CI for ANGSD or hapConX<2%, or shotgun data; 0: these conditions not satisfied)

V. X chromosome contamination estimates from ANGSD and hapConX - 110 sequences fail (1: upper bound of 95% CI for either metric is ≤5%, or this is not satisfied but lower bound for neither metric is ≥2%; or no measurement is available; 0: otherwise)

VI. remove all damage-restricted sequences - 2597 sequences fail (1=non-damage-restricted; 0=damage-restricted)

VII. date standard deviation is >1250 years - 32 sequences fail (1: <1250 years or no measurement; 0: otherwise)

Filter 4: Drop sequences with more than 100 relatives up to the second degree.

Filter 5: Remove sequences with evidence of date uncertainty. For each sequence, count the number of relatives up to the second degree and compute the standard deviation of their dates; exclude outliers where N > 10 and the date standard deviation exceeds 1000.

Filter 6: Remove datasets from the same individuals. Sort sequences by suffix hierarchy (ascending: 1=.DG, 2=.SG, 3=AG.BY.AA, 4=AG.TW.BY, 5=AG.TW, 6=.TW, 7=AG.BY, 8=.AG), then by IQS (descending). Remove duplicate datasets identified by KING[5].

Filter 7: Check for chromosomal abnormalities for each sequence. We have 22 IQS estimates for each autosome. Compare min(IQS) and mean(IQS), and drop outliers.

Filter 8: Remove date outliers by manual review and an outlier test. We use the 1240k SNP set to calculate the top 100 principal components (PCs) of individuals in the remaining sequences. These 100 PCs are used as explanatory variables to fit a linear regression with the date of the samples as the response variable. We use the Pearson residuals (Z-score) from this model to detect outliers. This residual measures how far a sample's reported date is from the date predicted by the PCs, relative to the model's residual standard error. Remove any sample with a residual magnitude greater than 4 (Figure S1.7). We do not apply this test to samples older than 10,000 years BP, as predictive power is very poor. Additionally, we removed 37 individuals where the archaeological information appeared to be systematically discrepant with the genetic clustering pattern.

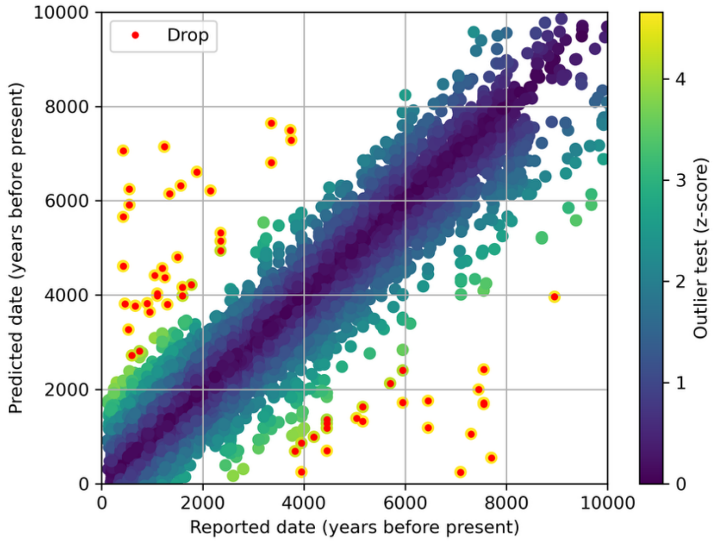

Figure S1.7: Outlier screen for sample dates. Predicted date (years before present) from a linear model on the top 100 PCs plotted against the reported date for sequences up to 10,000 years BP. Point color shows the Pearson residual (z-score). Samples with |z| > 4 (red) are removed.

Filter 9: Remove date outliers using first-degree relatives. If two sequences are first-degree related and their date estimates are 1000 years apart, drop the one if the outlier test z-score from the previous step is larger than 2.

Filter 10: Restrict to unrelated samples. Create a maximal set where no two pairs of sequences are related (up to the second degree).

A summary of all the individuals analyzed in this paper is presented in Table S1.4.

Table S1.4: Individuals analyzed in this study

Individuals	Description
12224	**1. Previously published individual (no newly reported data)**<br>6438 present-day (5935 SNP array genotyping, 503 shotgun)<br>5800 ancient (3232 capture, 2568 shotgun)
287	**2. Previously published individual with new shotgun sequences**
181	**3. Previously published individual with new capture data**
9568	**4. Never-before reported individual**<br>9487 capture, 81 shotgun

Supplementary Information section 2
Simulation of European demographic history

We conducted forward-in-time simulations to model human evolution according to European demographic history. The genome was divided into three compartments: coding, functional noncoding, and neutral. We tested a variety of models, including purifying selection (selection against newly arising deleterious alleles), and stabilizing selection (selection against variation), as these are two major processes known to shape patterns of human genetic variation. Each simulation pair includes an experimental condition with directional selection, and a corresponding negative control that can include purifying selection or stabilizing selection or both. The pair differ only by the absence of directional selection.

Demography

We conduct forward-in-time simulations using SLiM v4.0.1[6] to model human evolutionary history based on the semi-realistic demographic model outlined in Irving-Pease et al. 2024[7]. This model explicitly specifies four highly divergent ancestral populations that mixed to form three highly divergent populations that then mixed in turn in various proportions to form late groups in the region, with the degree of population differentiation and timing of population splits and mergers roughly matching what has been reconstructed over the course of dozens of studies of European demographic history since 2015 (Figure S2.1). Our simulations begin with a burn-in period in which the population is homogeneous, and then create population structure according to the Irving-Pease et al. 2024 model:

1) At 1500 Generations Before Present (GBP), we simulate migration of an out-of-Africa population (OOA) into two populations of an effective size of 15,000 individuals each: Northern Europeans (NE) and West Asians (WA) (Figure S2.1).
2) 700 generations after burn-in (800 GBP), we simulate bifurcation of individuals in WA to two form new populations: Caucasus hunter-gatherers (CHG) and Anatolian farmers (ANA). The population sizes are 3,000 and 5,000, respectively.
3) 900 generations after burn-in (600 GBP), we simulate bifurcation of NE to generate two new populations: Western hunter-gatherers (WHG) and Eastern hunter-gathers (EHG) with population sizes 2,000 and 3,000, respectively.
4) 1241 generations after burn-in (259 GBP), we generate Neolithic farmers (Neo) via admixture, in a 25:75 ratio, of WHG and ANA. Neo is initialized with population size 50,000 such that massive population growth occurs in a single generation.
5) 1323 generations after burn-in (177 GBP), we simulate admixture of CHG and EHG in a 50:50 ratio to generate 20,000 Yamnaya (Yam).
6) 1334 generations after burn-in (166 GBP), we simulate admixture of Neo and Yam in a 50:50 ratio to generate a Bronze Age (BA) population with size 50,000.

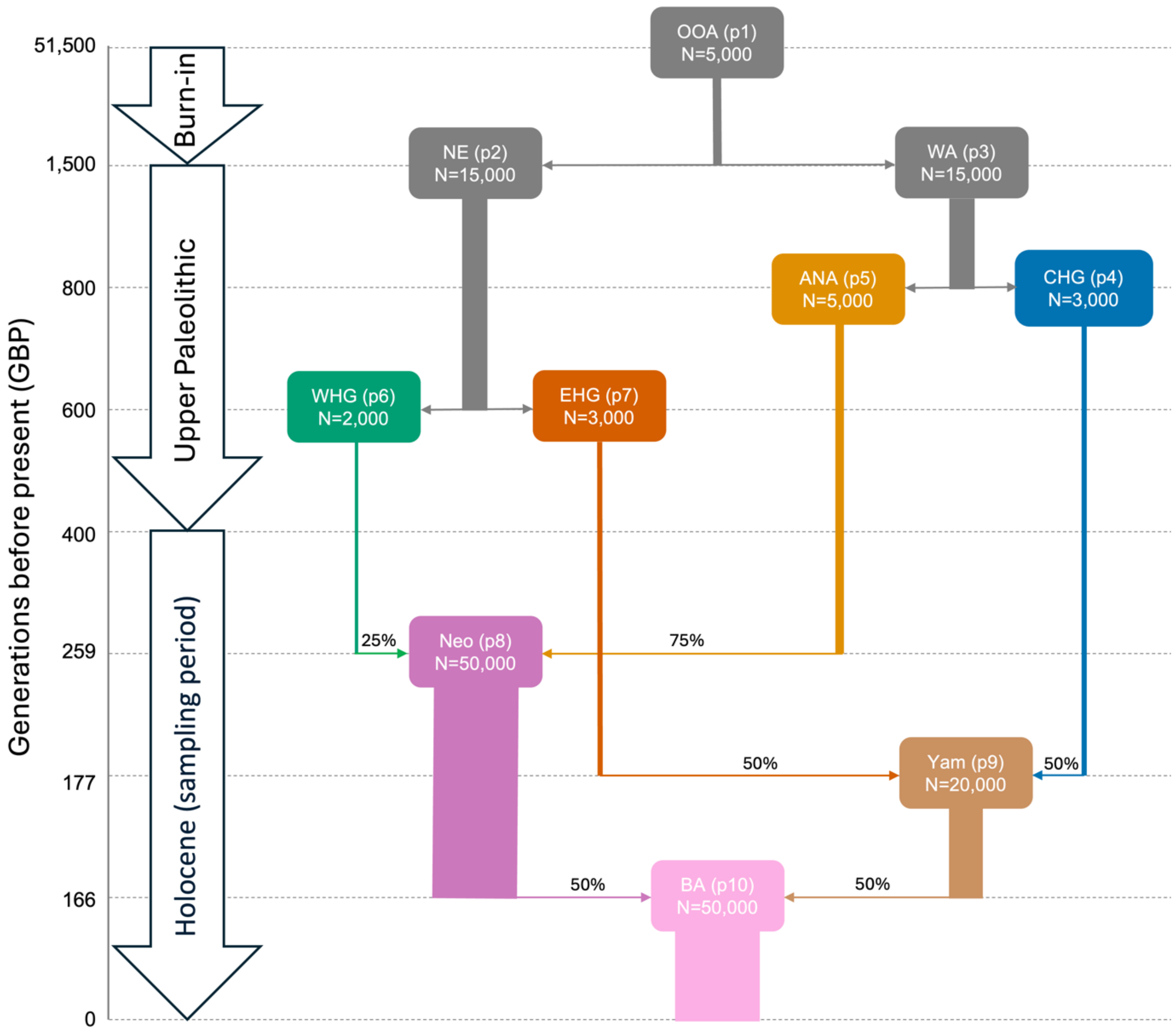

Figure S2.1: Schematic representation of our simulated population demography. This is a redrawn version of the model of Irving-Pease et al. 2024. Each population is designated by an acronym for its name, followed by the SLiM population identifier (in parentheses) and its population size N. Each bar represents the population's evolutionary time span, with width proportional to its population size N. Admixture proportions for ancestral populations are indicated where applicable. Abbreviations: OOA, Out-of-Africa; NE, Northern Europeans; WA, Western Asians; ANA, Anatolian farmers; CHG, Caucasus hunter-gatherers; EHG, Eastern hunter-gatherers; WHG, Western hunter-gatherers; Neo, Neolithic farmers; Yam, Yamnaya; BA, Bronze Age.

Burn-in period

To introduce standing genomic variation and permit equilibration to the optimal trait value of stabilizing selection, we simulate a period during which all evolutionary and phenotype-generating mechanics are permitted but there is neither demography nor sampling. The generations and population size for this "burn-in" period are 5,000 individuals in a single population for 50,000 generations. These parameters are chosen by previous literature on simulations indicating burn-in

should proceed for at least 10N generations, where N is population size[8,9]. We can think of the population resulting from this burn-in as the ancestral out-of-Africa population (OOA).

Sampling

To simulate our ancient DNA "time transect", we sample genomes every 10 generations starting at 400 generations before present (Figure S2.2). Sampling thus occurs at 41 unique time points. Ancestral compositions and sample sizes are selected to approximate real data (Figure S2.2).

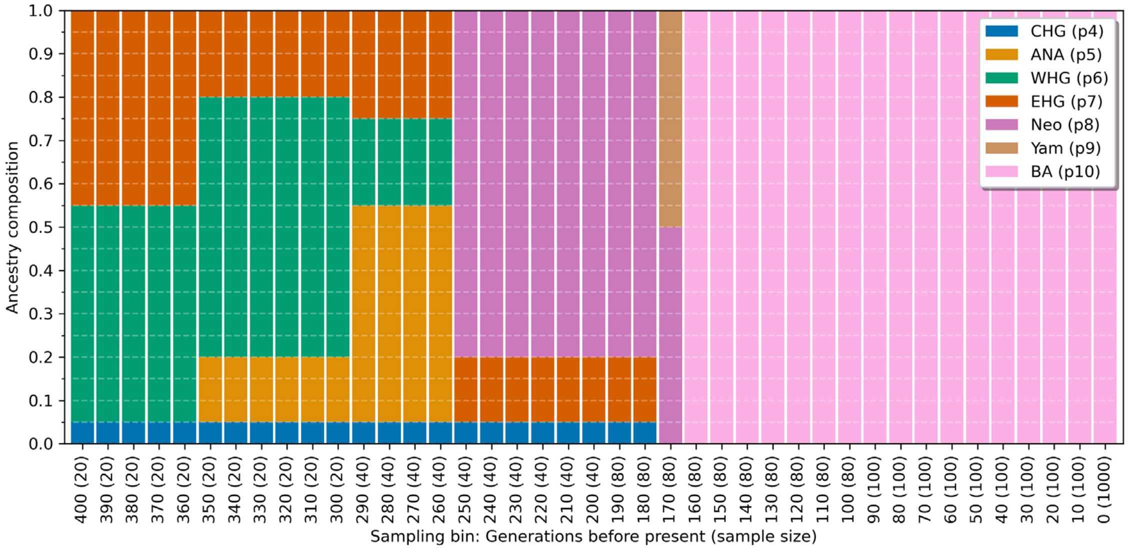

Figure S2.2: Sampling from simulated ancient DNA of Western Eurasia. Each bin represents a sampling time point, with the x-axis indicating the generation before present (sample counts in parentheses) and the y-axis displaying ancestral composition. Each color represents a population, and sampling in each bin is proportional to its ancestral composition at that time point. Ancestral compositions and sample sizes are selected to approximate the observed distribution in our actual dataset.

The goal of these simulations is to capture some of the real complexity of the dataset we are analyzing, both with regard to the nature of West Eurasian population structure, and with regard to the inhomogeneous sampling of real individuals in the dataset. We acknowledge that even this simulation is far from capturing the full complexity of the real history and data. For example:

(i) The true history certainly involved exchanges of genes among populations in between the main gene flow events that we model.

(ii) Our model does not capture the fact that real populations across the region had variable proportions of contribution from the ancestral sources. For example, real Neo had different proportions of the ANA and WHG sources, and real BA populations had different proportions of the Yam and Neo sources

(iii) Related to (ii), our model does not capture isolation-by-distance of groups from each other, for example in the Neo period and in the BA period.

(iv) The Irving-Pease et al. 2024[7] model we use as the basis for our simulations is tuned to European history, which we think is useful because the great majority of the samples we analyze are from Europe. However, this model does not capture population substructure in the Near East, which is also the source of a substantial number of the individuals in the dataset. It also does not adequately capture population structure in southern Italy and the Aegean, where in truth there was additional significant admixture of CHG-derived ancestry. And it does not capture the non-trivial proportions of North African and East Asian admixture present in some real individuals in the dataset.

Despite these limitations, the model we simulate captures a great deal of realistic complexity, and therefore allows us to carry out meaningful tests of whether our analytical framework is able to robustly correct for substructure, and calibrate thresholds of genome-wide statistical significance in the presence of such substructure.

Genomic architecture

Genomes in our simulation are diploid with recombination defined by the GRCh37/hg19 LD-based recombination rate map[10] (genetic_map_hg19_withX.txt.gz downloaded from https://alkesgroup.broadinstitute.org/Eagle/downloads/tables/) and genetic annotations based on GENCODE V47lift37[11] from the UCSC Table Browser (Figure S2.3). Details on the implementation of the gene annotation map are included in the section on Mutations. We simulate only a subset of the whole genome in each replicate for computational tractability. For each replicate, we randomly sample a genome chunk of a specified size, with a default of 10 Mbp unless stated otherwise. We then ensure that the proportion of coding regions in the sampled chunk is within 50% of the chromosomal average. Furthermore, the maximum length allowed for a region without recombination—defined as a recombination rate of 0 in the GRCh37/hg19 recombination rate map—is set at 20 Kbp. For brevity, we refer to the simulated genome subset in each replicate as the "genome."

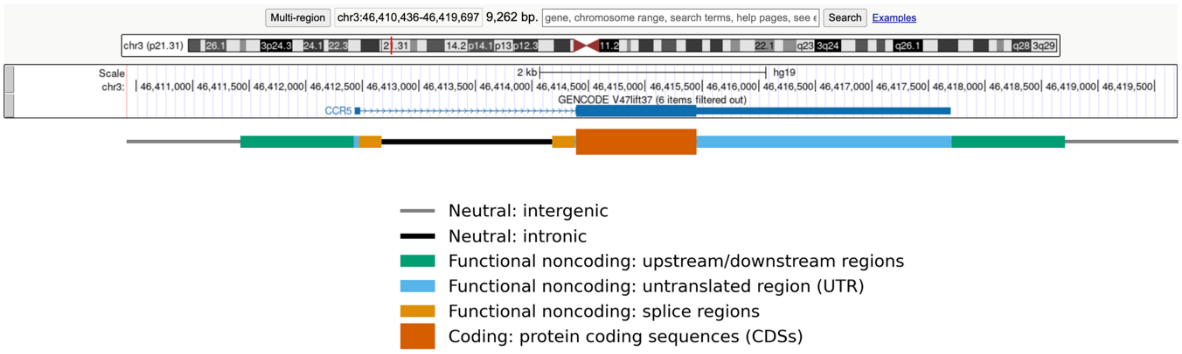

Figure S2.3: Partitioning of the human genome around the CCR5 locus into three categories based on GENCODE_V47lift37 annotations from the UCSC Genome Browser: neutral (intergenic, intronic), functional noncoding (upstream/downstream, UTRs, splice sites), and coding (protein-coding sequences).

Mutations

In empirical data, regions with many variants subject to purifying selection also tend to harbor a high number of trait-influencing variants[12–14]. We simulate three categories of genomic segments—“coding,” “functional noncoding”, and “neutral” (Figure S2.3)—each with a distinct distribution of mutation effects on traits and fitness.

Coding regions simulate areas strongly enriched for trait and fitness effects, functional noncoding regions simulate areas modestly enriched, and neutral regions introduce the neutral variation observed across most of the genome. Coding regions are defined as any segment annotated as a protein-coding sequence in the UCSC Genome Browser (GENCODE_V47lift37 annotations[11]). Regions annotated as upstream or downstream of a protein-coding sequence (defined as 1 kb upstream of a transcript start and 1 kb downstream of a transcript end), untranslated regions (both 5’ and 3’), or splice regions (defined as 200 bp from the start and end of each intron) are categorized as “functional noncoding”[14]. Regions not classified as coding or functional noncoding are simulated as neutral (Figure S2.3).

We define four types of mutations: m1, m2, m3, and m4. The functions of these mutation types vary depending on whether the simulation involves background or stabilizing selection. m1 mutations are always neutral, while m3 mutations, on average, have a stronger impact than m2 mutations. In simulations with background selection (model 2), m2 and m3 mutations are assigned selection coefficients. In simulations with stabilizing selection (model 1), m2 and m3 mutations influence the trait. In simulations incorporating both (model 3), m2 and m3 mutations possess both selection coefficients and trait impacts. Under experimental conditions in Model 2, m4 mutations are introduced with a predefined positive selection coefficient.

The distribution of mutation types across genomic regions, derived from empirical estimates[14], is constant across all experiments. Coding regions contain 40% m1 and 60% m3 mutations, functional noncoding regions contain 60% m1 and 40% m2 mutations, and neutral regions contain only m1 mutations. We use an empirically estimate of the mutation rate of $\mu_{total} = 1.4\times10^{-8}$ base pairs per gamete per generation[14].

Models of background selection

Model 1: With stabilizing selection mechanism

In Model 1, we simulate a phenotype under stabilizing selection with an optimal trait value, where all functional (non-neutral) mutations affect the trait and are assigned a trait effect size upon introduction. Background selection is induced through the stabilizing selection mechanism, which purges large-effect variants to maintain the optimal trait value. In the experimental condition, polygenic directional selection is introduced by shifting the optimal trait value at a predefined generation[8], while the negative control remains unchanged.

Model 2: With purifying selection mechanism

Model 2 does not incorporate stabilizing selection; background selection is induced solely through purifying selection. Here, each functional variant is assigned a negative selection coefficient and is continuously purged from the population. Without a simulated trait, directional selection in the experimental condition targets a single favored mutation. This model includes two experimental conditions:

Experimental condition 2.1: Soft sweep

A random standing variant in a functional region (coding or noncoding) is assigned a predefined positive selection coefficient at a specified generation to simulate a soft sweep[15].

Experimental condition 2.2: Hard sweep

A *de novo* mutation is introduced at a specific generation and assigned a positive selection coefficient to simulate a hard sweep.

For both experimental conditions, the negative control is identical and without directional selection. Subsequently, the Eyre-Walker model is used to generate effect sizes for functional variants (i.e., variants with a nonzero selection coefficient).

Model 3: With stabilizing and purifying selection mechanisms

Model 3: Although Models 1 and 2 are more widely used in the literature[8,9,14,16], we implemented a more complex scenario (Model 3) to further probe the behavior of the testing framework. Model 3 combines stabilizing and purifying selection mechanisms to induce background selection. In this model, each functional mutation is assigned a negative selection coefficient and an effect size (based on the Eyre-Walker model[17]) at the time of introduction. Thus, the effective selection pressure on each variant is determined by its effect size (through the stabilizing selection mechanism) and its selection coefficient (through the purifying selection mechanism). In the experimental condition, polygenic directional selection is introduced by shifting the optimal trait value at a predefined generation, whereas the negative control remains unchanged.

We differentiate between directional selection on *phenotypes*, on *de novo variants* (mutations introduced during the sampling time period), and on *standing variants* (alleles which existed prior to the sampling time period). Directional selection on a phenotype is defined as a shift in the most evolutionarily fit value for that phenotype[8]. Because a shift in phenotypic optimum can only occur in the presence of stabilizing selection, we implement directional selection on phenotypes only in models which also implement stabilizing selection. We simulate the two other types of directional selection in models without stabilizing selection. Directional selection on a standing variant occurs when the selection coefficient for a preexisting allele shifts at some discrete timepoint[15], whereas directional selection on *de novo* variation occurs when a new mutation with a beneficial selection coefficient is introduced to the genome[18,19].

Selection mechanisms

Purifying selection mechanism

Once mutations are assigned to their categories, simulations with a purifying selection mechanism (models 2 and 3) are needed to determine the extent to which each mutation impacts individual fitness. The following section thus applies only to simulations including purifying selection and not to model 1, which only includes stabilizing selection.

We draw selection coefficients for type m2 mutations from an exponential distribution with an expected value of -5×10^{-4}, concordant with the mean value of deleterious mutations in non-coding, functional regions estimated in [14]. Following empirical descriptions of deleterious mutations in protein-coding regions of humans, we draw selection coefficients for type m3 mutations from a gamma distribution with an expected value of -0.03, shape parameter 0.206 (ref.[20]). Both categories of deleterious mutations are mapped to individuals' fitness values via the following equation, which is the default fitness function in SLiM:

$$\hat{\mathcal{F}}_i = \prod_j (1 + ds_j D_{ij})$$

Here, d=0.5 is the dominance coefficient, s_j is the selection coefficient for SNP j, and D_{ij} is the genotype of individual i at SNP j. In models including both purifying and stabilizing selection (model 3), individual fitness values, $\hat{\mathcal{F}}_i$ are scaled based on their phenotype values to account for the impact of stabilizing selection (discussed in the *stabilizing selection mechanism* section).

Stabilizing selection mechanism

Phenotype

To simulate stabilizing selection, we draw selection coefficients from the same distributions as described in the *purifying selection mechanism* section and use the Eyre-Walker model[17] to define trait effect size β_j for SNP j. For model 1, we set ($s_j = 0$), while for model 3, a functional variant has both non-zero effect size and selection coefficient. The Eyre-Walker model[17] takes the form $\beta_j = \delta\left(4N_e|s_j|\right)^{\tau}(1+\varepsilon_j)$, where δ is a random sign uniformly selected from +1 or -1, and ε_j is randomly generated noise from a normal distribution with mean 0 and variance $\sigma^2 = 0.1$ (ref.[21]). We used the parameters $\tau = 0.5$ (ref.[22,23]) and $N_e = 5000$.

The genetic contribution to the phenotype of individual i, or "genetic score" (G_i), is defined as the effect-size-weighted sum of the individual's genotypes: $G_i = \sum_j \beta_j D_{ij}$. The phenotype of individual i is then $\phi_i = G_i + E_i$, where $E_i \sim N(0, V_E)$, $V_E = V_G(1 - h^2_{SNP})/h^2_{SNP}$, $h^2_{SNP} = 0.5$, and V_G and V_E represent the genetic and environmental components of the phenotype's variance, respectively.

Stabilizing selection

Stabilizing selection is a type of natural selection that favors phenotypes near the population's optimum, reducing variation by selecting against extreme trait values and maintaining a stable

average phenotype over time. We simulate stabilizing selection in models 1 and 3 using a Gaussian fitness function. For each individual i, the fitness value $\mathcal{F}_i$ is defined as:

$$\mathcal{F}_i = \hat{\mathcal{F}}_i \times e^{\frac{-(\phi_i - \Phi)^2}{2\sigma_s^2}}$$

where $\hat{\mathcal{F}}_i$ is the multiplicative impact of selection coefficients for all functional variants, introduced in the *purifying selection mechanism* section. In model 1, we set $\hat{\mathcal{F}}_i = 1$. The Gaussian scaling factor adjusts the fitness function based on the deviation of each individual's phenotype from a specified optimum Φ[8,9,24]. We set $\Phi = 100$ and use a standard deviation $\sigma_s = 10$ (ref.[8]).

Directional selection mechanism

In line with prior literature, we simulate directional selection for experiments 1 and 3 as an abrupt shift in the phenotypic optimum for stabilizing selection[8,9]. At 200 GBP, our simulation shifts the phenotypic optimum from some initial value, $\Phi_1 = 100$, to a new value, $\Phi_2 = 105$. All subsequent fitness calculations are performed exactly as described in Background Selection (for model 2) and Stabilizing Selection (for models 1 and 3).

For model 2, there are no phenotypes. We therefore simulate positive directional selection on the variant level. In experiment 2.1, we randomly select an *a priori* neutral genetic variant in either a coding or non-coding region at 200 GBP as a parametrized value and assign it a positive selection coefficient, randomly drawn from 0.01, 0.02, or 0.03.

In model 2.2, we randomly introduce a new mutation 300 GBP to be either coding or noncoding; the new mutation has a positive selection coefficient (s = 0.05). Because selection in model 2.2 acts on new mutations, using the same parameters as in experiment 2.1 would take much longer for the mutation to be established at more than minimal frequency and thus escape the stochastic phase of a hard selective sweep[25]. Therefore, we use an earlier onset of selection with a stronger selection coefficient.

Simulating GWAS

In Models 1 and 3, the Eyre-Walker model is embedded in the SLiM simulations as we simulate a phenotype in those models. However, for Models 2.1 and 2.2, we do not simulate a trait explicitly, and at the end of the simulation, we calculate trait effect sizes for each variant according to the Eyre-Walker model, as explained in the Phenotypes section. Finally, we define the constant c such that $\hat{\beta}_j = c\beta_j$ (ref.[23]) and

$$h^2_{SNP} = \sum_i 2f_i(1-f_i)\hat{\beta}_j^2 = 0.5.$$

Here, f_i is the allele frequency of SNP i at the final simulation stage (time zero), and $\hat{\beta}_j$ represents the true effect sizes of alleles for a normalized trait ($V_\phi = 1$), which are zero for non-functional SNPs. However, to simulate a GWAS, we estimate the marginal effect size given the true effect size. This means that a variant in LD with a causal variant may have a nonzero marginal effect size even if its true effect size is zero. We use the LD matrix of all variants at time zero with a minor allele frequency greater than 1% and effect size $\hat{\beta}_j$ to obtain the marginal effect size using Equation 1 from ref.[26]. Later, we use Equation 2 from the same paper to estimate the variance of the marginal effect sizes, assuming a trait variance of 1. Finally, we set the sample size to approximate the genomic control (λ_{GC}) of 2.5 observed for height in the Pan-UKBB.

Empirical consistency checks for simulations

In this section, we describe various analyses we performed to test if our simulation outputs matched empirical data qualitatively with respect to features relevant to our subsequent analyses.

Background selection and neutral diversity

The genomic architecture of the simulated genomes includes three elements: coding, functional noncoding, and neutral regions (Figure S2.3). Background selection is expected to be stronger in regions under higher selective pressure (these pressures are stabilizing selection in Model 1, purifying selection in Model 2, and both in Model 3). We use heterozygosity[27] as a measure of neutral diversity and observe that coding regions have the lowest diversity, neutral regions the highest, and functional noncoding regions in between, consistent with our expectations and with the approximately correct magnitude of effects[14] (Figure S2.4).

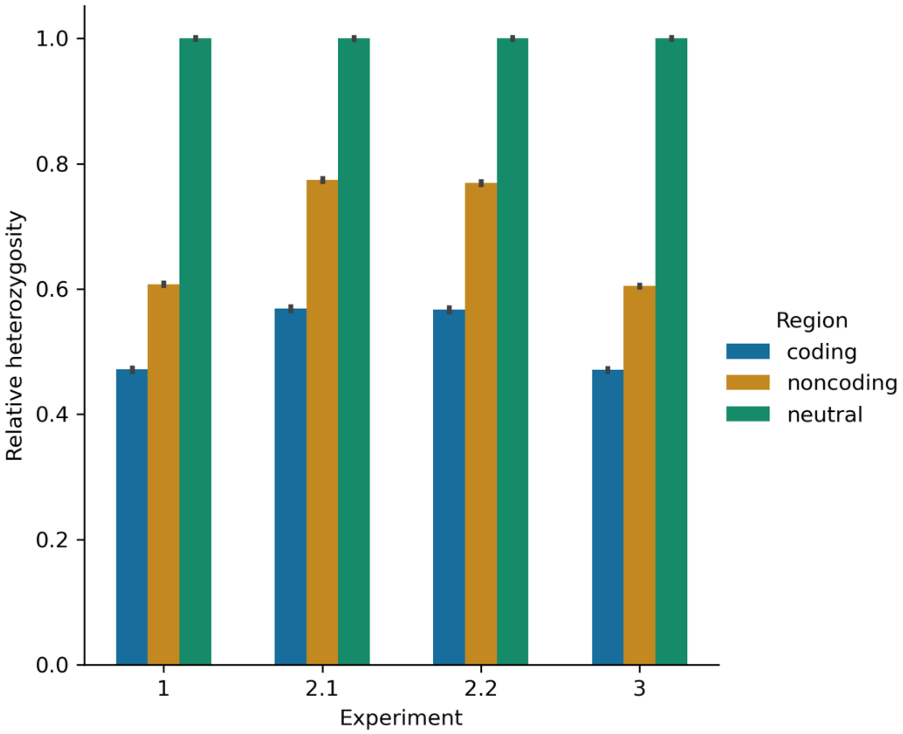

Figure S2.4. Relative heterozygosity in coding and noncoding regions normalized to neutral regions across experiments. Coding regions exhibit the lowest heterozygosity, noncoding regions are intermediate, and neutral regions show the highest levels, as expected. Error bars represent the standard error of the mean. The absolute heterozygosity of neutral regions is approximately $3x10^{-4}$. In empirical data, heterozygosity relative to neutral regions is approximately 0.5 in coding regions and 0.9 in noncoding regions, with an absolute heterozygosity in neutral regions of approximately $8x10^{-4}$.

Mutation age and trait effect size

Purifying and stabilizing selection mechanisms rapidly remove mutations with large effects, creating an inverse relationship between mutation age and trait effect size[13]. In all simulated models, the expected allele age as a function of the quantile of the absolute effect size decreases monotonically, consistent with this expectation (Figure S2.5).

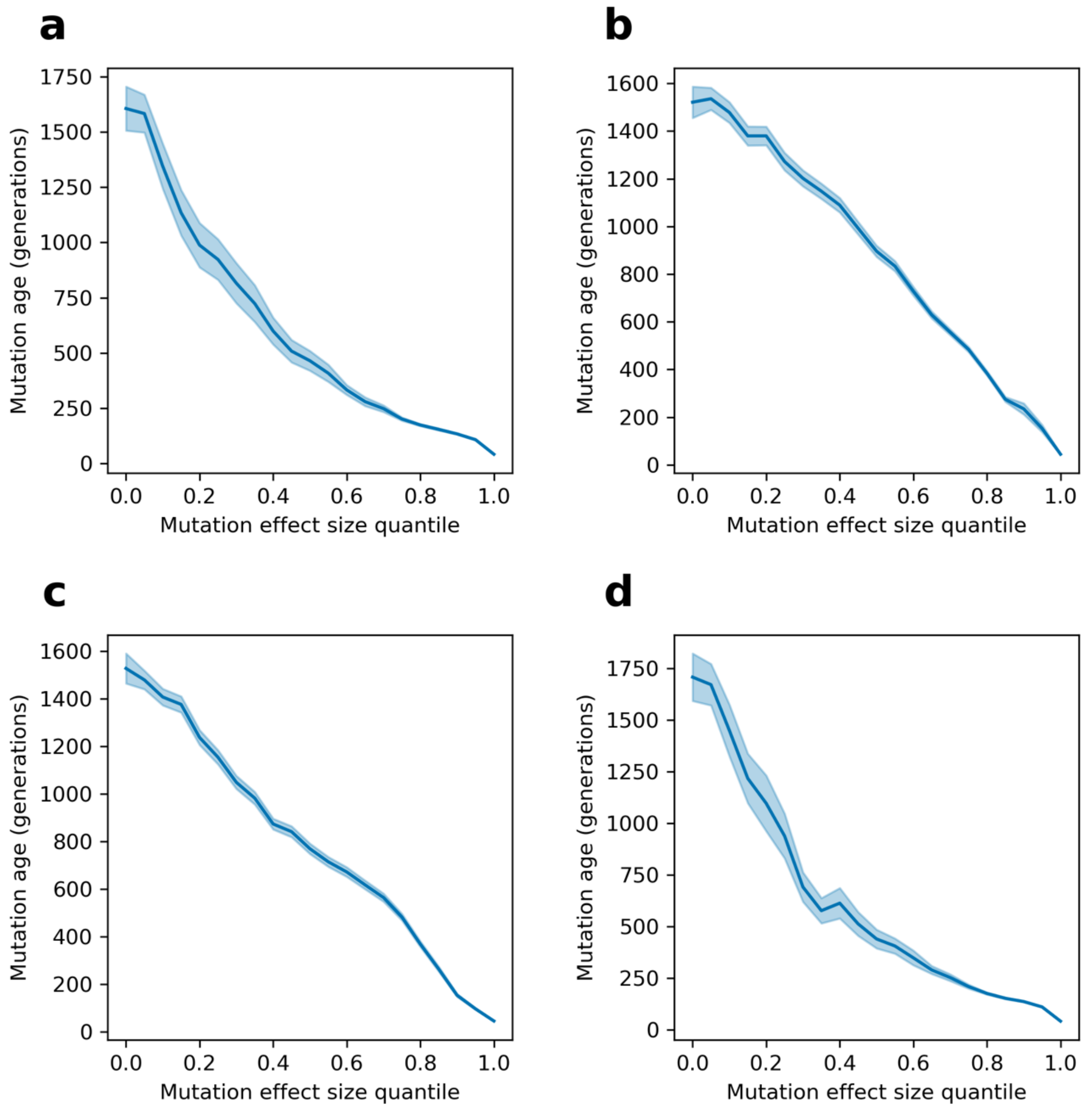

Figure S2.5: Relationship between effect size and the mutation age of functional mutations for (**a**) Model 1, (**b**) Model 2.1, (**c**) Model 2.2, and (**d**) Model 3. The shaded area represents the 95% confidence interval of the estimated mean.

Allelic effect and frequency under stabilizing selection

Stabilizing selection maintains an optimal trait value by reducing phenotypic variance, primarily by eliminating alleles that cause extreme deviations from the optimum. Alleles with large effects tend to drive phenotypes far from the optimum and are therefore removed more rapidly from the population. In contrast, alleles with smaller effects are less deleterious and persist at intermediate frequencies, resulting in a U-shaped distribution when plotting effect size against allele frequency[16]. Our simulations reproduce these dynamics: the plot of trait effect size versus allele frequency for trait-increasing alleles shows a U-shaped relationship that is qualitatively consistent with empirical observations (Figure S2.6).

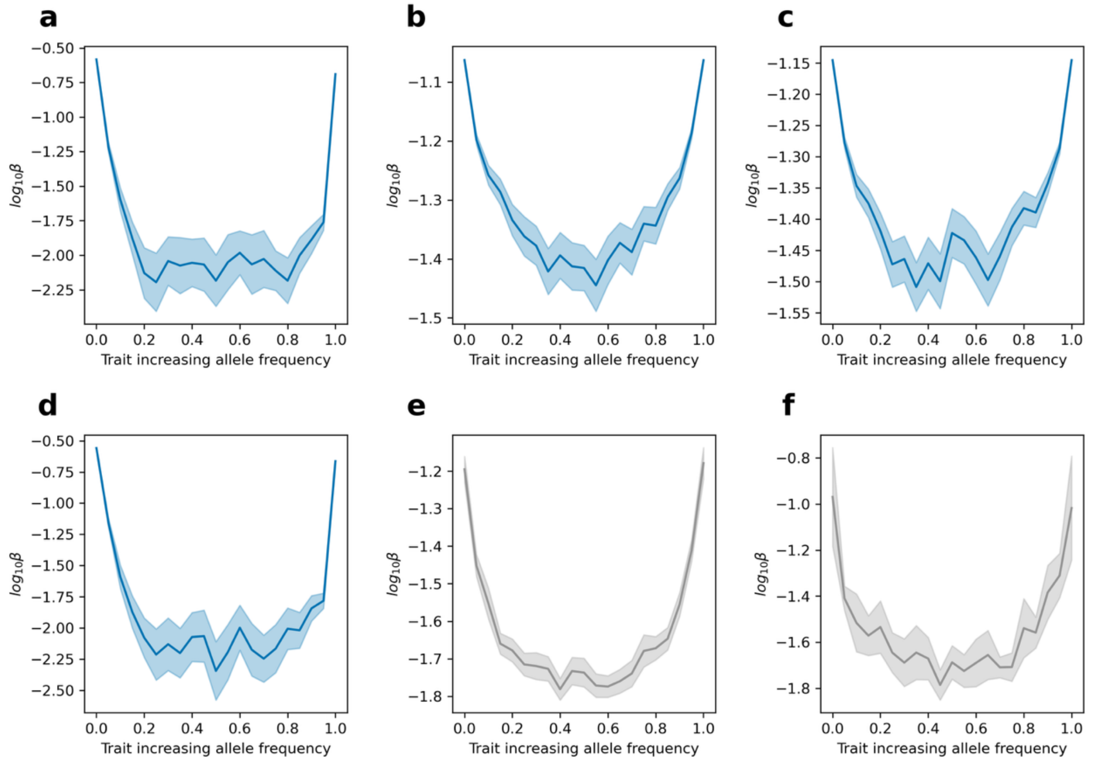

Figure S2.6: Relationship between trait effect size and the frequency of the trait-increasing allele for (**a**) Model 1, (**b**) Model 2.1, (**c**) Model 2.2, and (**d**) Model 3. Panels (**e**) and (**f**) show, in gray color, the corresponding empirical patterns for height (UK Biobank Data-Field 50) and cholesterol (UK Biobank Data-Field 30690), respectively. The UK Biobank analyses include variants with P-values < $5x10^{-8}$, LD-clumped with $r^2 < 0.05$ and $D' < 0.2$ using a 500 kbp window. The shaded area represents the 95% confidence interval of the estimated mean.

Principal component analysis (PCA)

We simulated the demography of the European population[7] and validated our simulation using the top two principal components. The overall structure of the simulated populations resembles that of real data, confirming that the simulation functions as intended. However, the evolutionary history represented in the real data is considerably more complex. For example, our simulation of the Bronze Age (BA) assumed a single panmictic population, a simplification that does not capture the true complexity. This discrepancy is evident in Figure S2.7, where Bronze Age samples in the real data are more dispersed than the tightly clustered samples in the simulation. In the section *Power analysis of GLMM and GLM and co-linearity*, we discuss how this complexity necessitates the use of a GLMM, rather than a GLM with principal components as covariates, to address issues related to population structure and collinearity simultaneously.

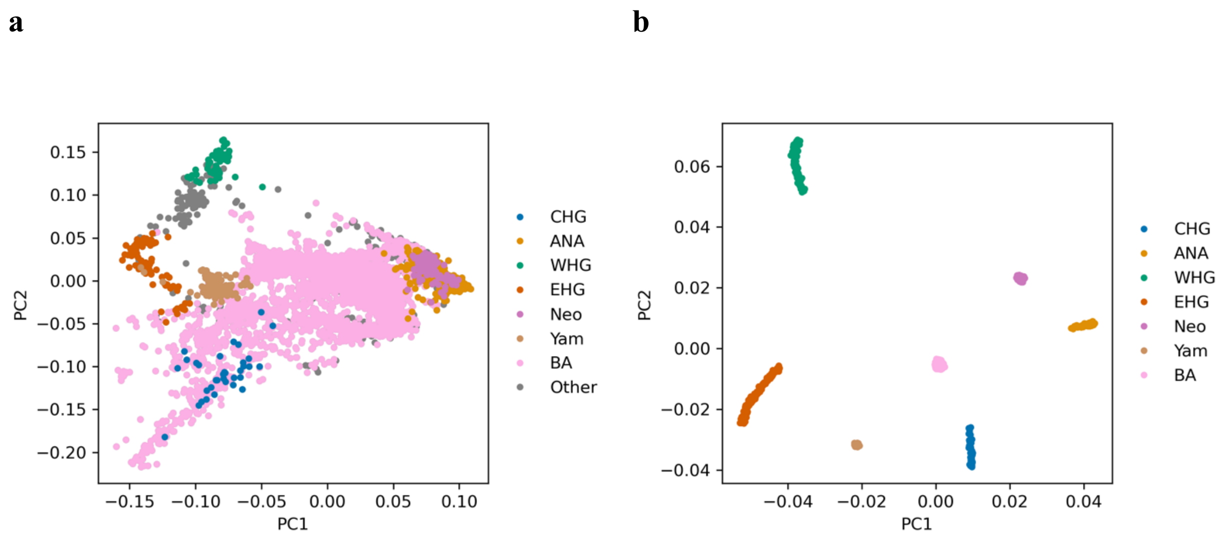

Figure S2.7: Principal components analysis on (a) real data from Western Eurasia and (b) simulated data based on the approximated demography of Western Eurasia. The simulated data capture broad patterns, but the real data exhibit much more complex substructure.

Analyzing the simulations

Enrichment of GWAS hits for variants under directional selection

We ran SLiM simulations for Experiments 1, 2.1, 2.2, and 3 over a 10-Mbp region randomly chosen from the genome. Each experiment includes 800 simulations (400 with directional selection and 400 without). The GRM matrix was calculated separately for each simulation using all other simulations in the same experiment to avoid proximal contamination, similar to the leave-one-chromosome-out (LOCO) approach[28].

For each experiment, we computed enrichment for a negative control (simulations without directional selection) and a positive control (all simulations, with and without directional selection). Enrichment is defined as the fraction of variants beyond the *P* value threshold of the selection statistics on the x-axis that are GWAS hits after adjusting for minor allele frequency (Supplementary Information section 3). In every case, enrichment was observed only when directional selection was included (Extended Data Figure 4).

Enrichment in GWAS as an approximation for False Discovery Rate

We used simulations from models 2.1 and 2.2. For simulations with directional selection, we retained only SNPs within 25 kbp of the favored mutation, considering these variants as positive cases of directional selection; all variants in simulations without directional selection served as controls. We then estimated the False Discovery Rate (FDR) as a function of the p-value threshold of the GLMM method. The enrichment plot for these simulations, as shown in Figure 1b, closely tracks the 1-FDR curve up to the plateau. Therefore, this enrichment plot can be used to approximate the FDR and control for inflation in the summary statistics.

In models 1 and 3, directional selection is polygenic and simulated by shifting the optimal value. Because each simulation covers only a 10 Mbp region, some trait-affecting variants are in close proximity, making it difficult to label variants cleanly as positive or negative controls as in models 2.1 and 2.2.

The magnitude of the enrichment depends on the fraction of variants identified as GWAS hits. For example, if 1% of variants are GWAS hits (ignoring adjustments for minor allele frequency), the maximum absolute enrichment value cannot exceed 100. In these simulations, only a small fraction of variants are GWAS hits due to computational constraints. In real GWAS, a much larger portion of the genome (10–20%) is identified as GWAS hits, making the absolute enrichment magnitude appear smaller. Nevertheless, our study relies on the plateau effect of the enrichment pattern rather than the absolute magnitude to approximate FDR.

Power analysis of GLMM and GLM and co-linearity

We adapt the simulations from Model 2.1 and run a SLiM simulation on a random 100-kbp region of the genome. We use a list of selection coefficients starting at 0.0 and increasing by 0.001 up to 0.03. We run approximately 4000 simulations per selection coefficient, except for s = 0, for which we run 20,000 simulations. For non-zero selection, we set the onset of selection at 200 generations ago for half of the simulations and 300 generations ago for the other half. We use the favored allele in the simulations with directional selection as the positive case (discarding all other SNPs), and use all the SNPs in simulations without directional selection as control. We set the False Positive Rate (FPR) to 1e-5 because we expect only a very small fraction of variants to be under selection. A higher FPR, such as 0.05, would lead to a False Discovery Rate (FDR) near 1, meaning nearly all identified variants would be false positives.

In these simulations, the GLMM outperforms all GLM models regardless of the number of PCs used as covariates. In GLM models, power increases as the number of PCs rises from 0 to 3, but adding a fourth PC leads to a drastic and continued decline in power. This is expected because our simulation employs a three-way admixture model, so three PCs are sufficient to capture the structure and remove false positives due to population stratification (Figures S2.1 and S2.7). Adding more PCs introduces collinearity and inflates the variance of the time component (selection coefficient), resulting in a loss of power (Figures S2.8 and S2.9). This suggests that for the simple model in our simulation, three (or even two) PCs are sufficient to resolve population structure, whereas using either too few (0 or 1) or too many (4 or more) PCs leads to severe loss of power—either from unresolved stratification or from collinearity. In contrast, real data exhibit complex substructure even after the Bronze Age, necessitating additional PCs that can further reduce power through collinearity (Figures S2.8, S2.9). To effectively control for population structure while minimizing power loss, we employ the GLMM model throughout this study.

To directly evaluate our approach, which uses a GLMM with a significance threshold of $|X| > 5.45$, and to compare its performance with GLMs incorporating varying numbers of principal components as covariates, we conducted a power analysis across these regression models. The results are summarized in Table S2.1. Consistent with our previous observations (Figures S2.8–S2.10), GLMs including two or three principal components perform relatively better than other GLM configurations, whereas models with too few or too many principal components show substantially reduced performance. Across all simulation settings, the GLMM consistently outperforms all GLM variants by a large margin.

We further evaluated the bias of the estimated selection coefficient as a function of the true simulated selection coefficient across all simulations used in Figure S2.8, summarized in Figure S2.11. Overall, the estimated selection coefficients track the true simulated values reasonably well. However, bias is more pronounced for variants with lower minor allele frequency. We examine the origin of this effect in detail in the following section, titled “Non-normality of the null distribution in GLMM test statistics.”

a

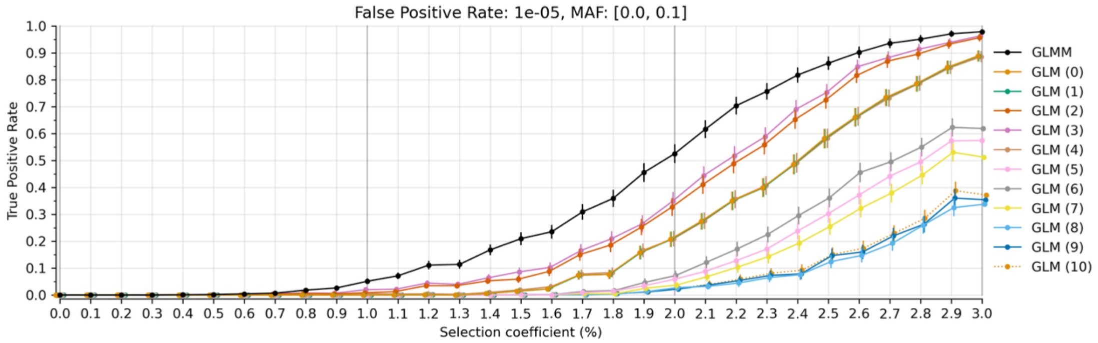

b

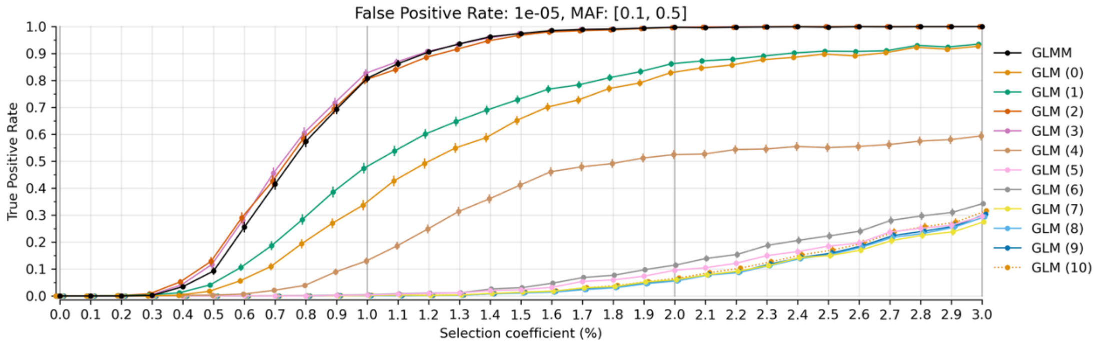

Figure S2.8: True positive rate at a false positive rate of 1e-5 versus selection coefficient for MAF ranges (a) [0, 0.1] and (b) [0.1, 0.5]. The black line shows the GLMM model, and the colored lines show the GLM model (with the number of PCs in parentheses). Selection coefficients range from 0.0 to 0.03 in 0.001 increments.

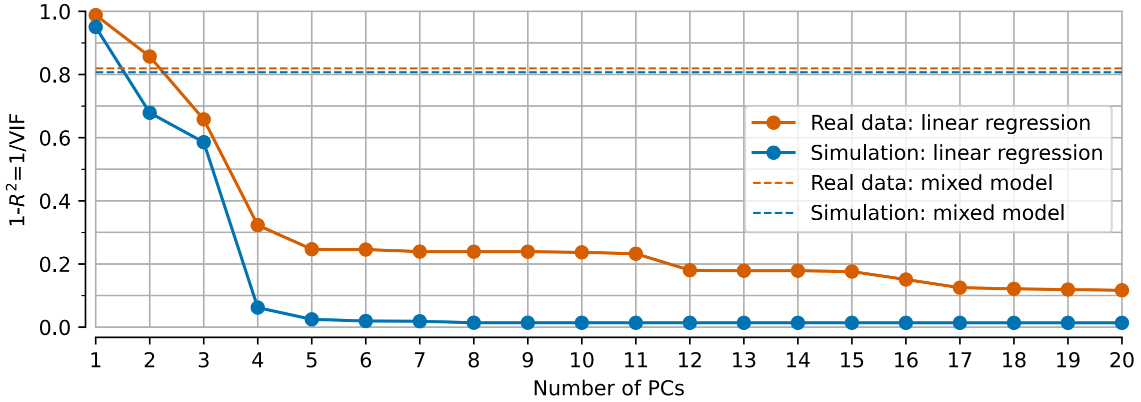

Figure S2.9: Loss of power due to collinearity between time and principal components. The y-axis shows $1 - R^2$ (equal to 1/VIF), a proxy for statistical power; lower values indicate greater variance inflation and reduced power. Each dot represents a linear regression with time as the response and the number of PCs (x-axis) as covariates. Both simulated and real data reveal that including more PCs increases collinearity with time, diminishing power. In simulations, power drops sharply after three PCs, while in real data the decline is more gradual due to a complex structure not captured in the simulation (so continued correction adds value). R^2 is the coefficient of determination, indicating the proportion of variance in time explained by the PCs. VIF (variance inflation factor) quantifies the inflation of the variance of the estimated coefficient due to collinearity. The dashed lines indicate $1 - R_c^2$, where R_c^2 is the conditional R^2 (ref.[29]). R_c^2 is the proportion of variance in time explained by the fixed effects and the genetic random effect in a linear mixed model, where time is the response variable. The model includes an intercept as the fixed effect and a random effect with a GRM as its covariance structure. In this model, since the only fixed effect is the intercept, which contributes no variance, R_c^2 effectively reflects the variance explained by the genetic random effect. It measures the model's goodness of fit and is analogous to R^2 in ordinary linear regression. We used 20,000 simulations under Model 2 without directional selection on a randomly chosen 100-kb genomic region.

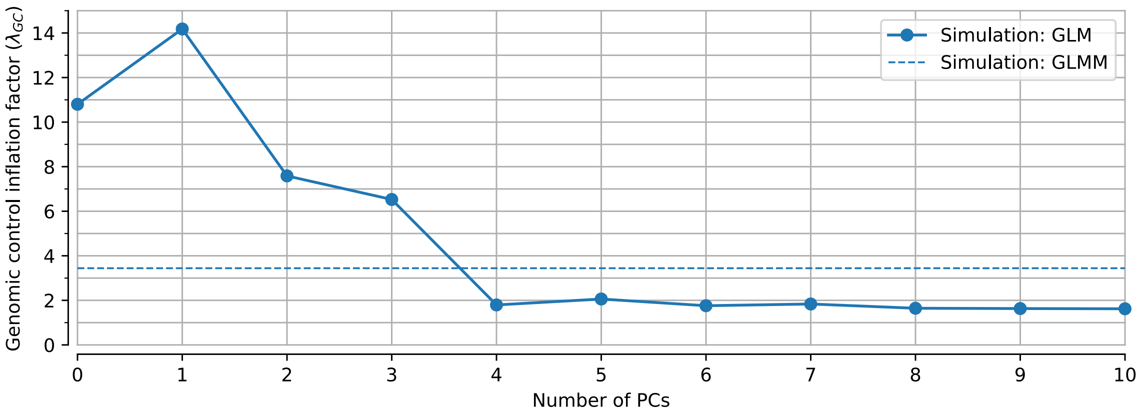

Figure S2.10: Genomic control inflation factor (λ_{GC}) for the GLM and GLMM in simulation. λ_{GC} is defined as median(z^2)/0.455, which quantifies the median inflation of the z^2 statistics relative to a chi-square distribution with one degree of freedom. Various factors, including polygenic signal, confounding due to population structure, and SNP assessment using a modern reference panel, may contribute to this inflation (see section "SNP ascertainment using a modern reference panel"). We used 20,000 simulations under Model 2 without directional selection on a randomly chosen 100-kb genomic region.

Table S2.1: True positive (TP) and False positive (FP) of different regression models using the significance threshold of |X|>5.45. Each experiment includes 800 simulations (400 with directional selection and 400 without).

	Experiment 1		Experiment 2.1		Experiment 2.2		Experiment 3	
	TP (%)	FP (%)	TP (%)	FP (%)	TP (%)	FP (%)	TP (%)	FP (%)
GLMM	25.50	0.00	59.25	0.00	89.00	0.00	22.25	0.00
GLM (0)	0.25	0.00	31.25	0.00	72.50	0.00	10.75	1.50
GLM (1)	2.25	0.00	35.75	0.00	75.25	0.00	3.75	0.25
GLM (2)	20.50	0.00	50.50	0.00	88.75	0.00	6.00	0.00
GLM (3)	18.00	0.00	46.75	0.00	84.00	0.00	9.25	0.25
GLM (4)	0.25	0.00	24.75	0.00	17.50	0.00	0.00	0.00
GLM (5)	0.00	0.00	9.00	0.00	45.00	1.25	0.00	0.00
GLM (6)	0.00	0.00	12.50	0.00	51.50	3.00	0.00	0.00
GLM (7)	0.00	0.00	15.25	0.25	45.50	0.50	0.00	0.00
GLM (8)	0.00	0.00	13.50	0.00	42.00	0.00	0.00	0.00
GLM (9)	0.00	0.00	13.50	0.25	39.50	0.00	0.00	0.00
GLM (10)	0.00	0.00	13.00	0.00	41.25	0.25	0.00	0.00

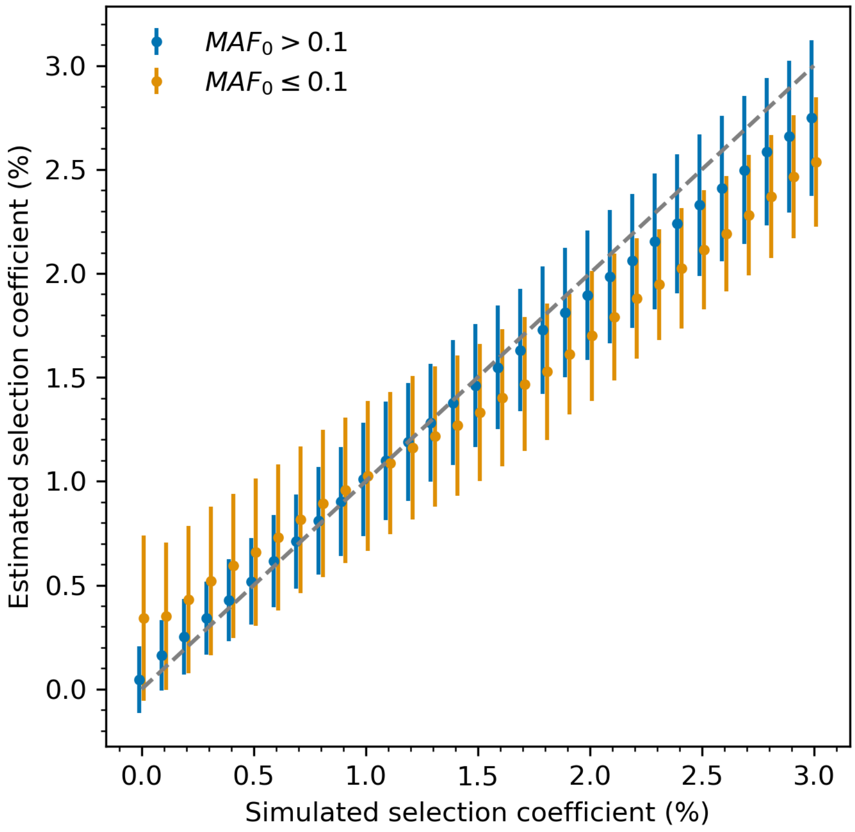

Figure S2.11: Accuracy and bias of selection coefficient estimation. Estimated selection coefficients from the simulations shown in Figure S2.8, plotted against the true simulated selection coefficients. Points represent mean estimates, and error bars indicate one standard deviation of the empirical distribution of estimated selection coefficients across simulations. Results are shown separately for variants with initial minor allele frequency $MAF_0 > 0.1$ and $MAF_0 \leq 0.1$. The dashed line denotes $y = x$.

Non-normality of the null distribution in GLMM test statistics

Under the null hypothesis, test statistics are often assumed to follow a normal distribution, and inflation is typically corrected using genomic control (λ_{GC}) when only a small fraction of the genome is affected. To evaluate this assumption, we adapted simulations from Model 2.1 and ran them in SLiM on random 100-kbp genomic regions, generating ~3.6 million SNPs from 20,000 replicates without directional selection. After applying λ_{GC} correction, the QQ plot shows that observed P-values are substantially overestimated, demonstrating a clear mis-specification of the null (Figure S2.12). This results in severe loss of power because the normal null assumption is invalid in this GLMM setting.

In addition, GLMM summary statistics exhibit frequency-dependent bias and inflation in Z-scores. A major contributor is SNP ascertainment due to imputation using modern reference panels, which preferentially retain variants with higher minor allele frequency in the reference panel at time zero (MAF_0). Because imputation quality improves with higher MAF_0 [30], SNPs that drifted upward in frequency are more likely to pass quality control, creating an apparent bias and inflation in Z-scores. For two neutral SNPs starting at the same historical frequency, the one with higher MAF_0 is preferentially ascertained, producing apparent bias and inflation in the test statistics. Given that the allele frequency spectrum is strongly skewed toward rare alleles[31,32], this ascertainment mechanism alone can produce a genomic inflation factor even in the absence of population structure or true selection (Figure S2.13).

The non-linear logit link function in the GLMM further amplifies this distortion. To assess its contribution, we compared GLMM to GLM (with logit link) and ordinary least squares (OLS), modeling genotype as the response with time and principal components as predictors. The GLM shows a similar or stronger frequency-dependent bias compared with GLMM, whereas OLS largely eliminates the distortion (Figure S2.13). This highlights that both ascertainment and the non-linear transformation inherent to logistic models contribute to the observed bias and inflation.

These results imply that while our false discovery rate (FDR) calibration and genome-wide significance thresholds remain valid, standard approaches such as genomic control or LDSC intercepts[33] are inappropriate for adjusting inflation in this context. The observed inflation reflects not only unresolved population structure but also biases introduced by SNP ascertainment and the non-linear transformation in the GLMM framework (Figure S2.13).

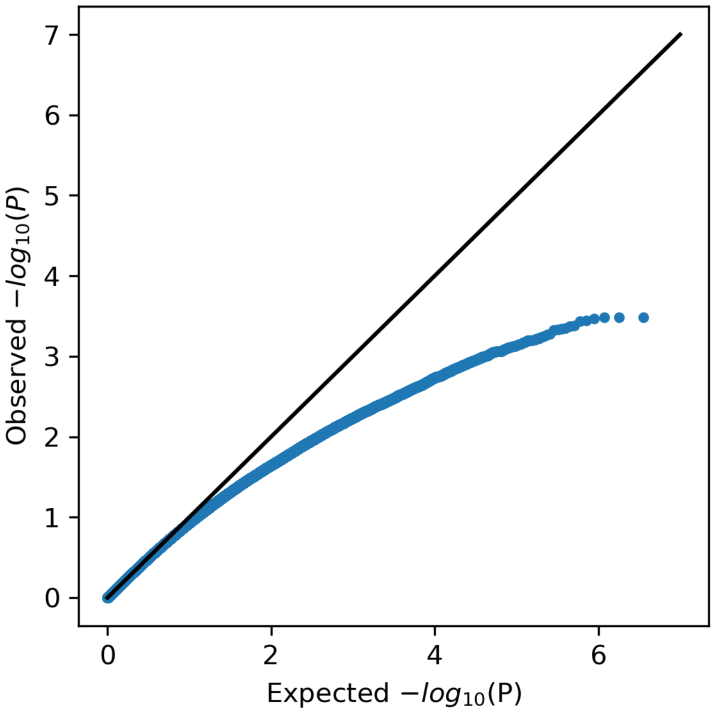

Figure S2.12: Quantile–quantile (QQ) plot of observed P-values against expected P-values from GLMM test statistics after genomic control (λ_{GC}) correction, assuming the test statistics follow a normal distribution under the null. Null simulations are adapted from Model 2.1 and run in SLiM on a random 100-kbp region of the genome, generating around 3.6 million SNPs from 20,000 replicates without directional selection.

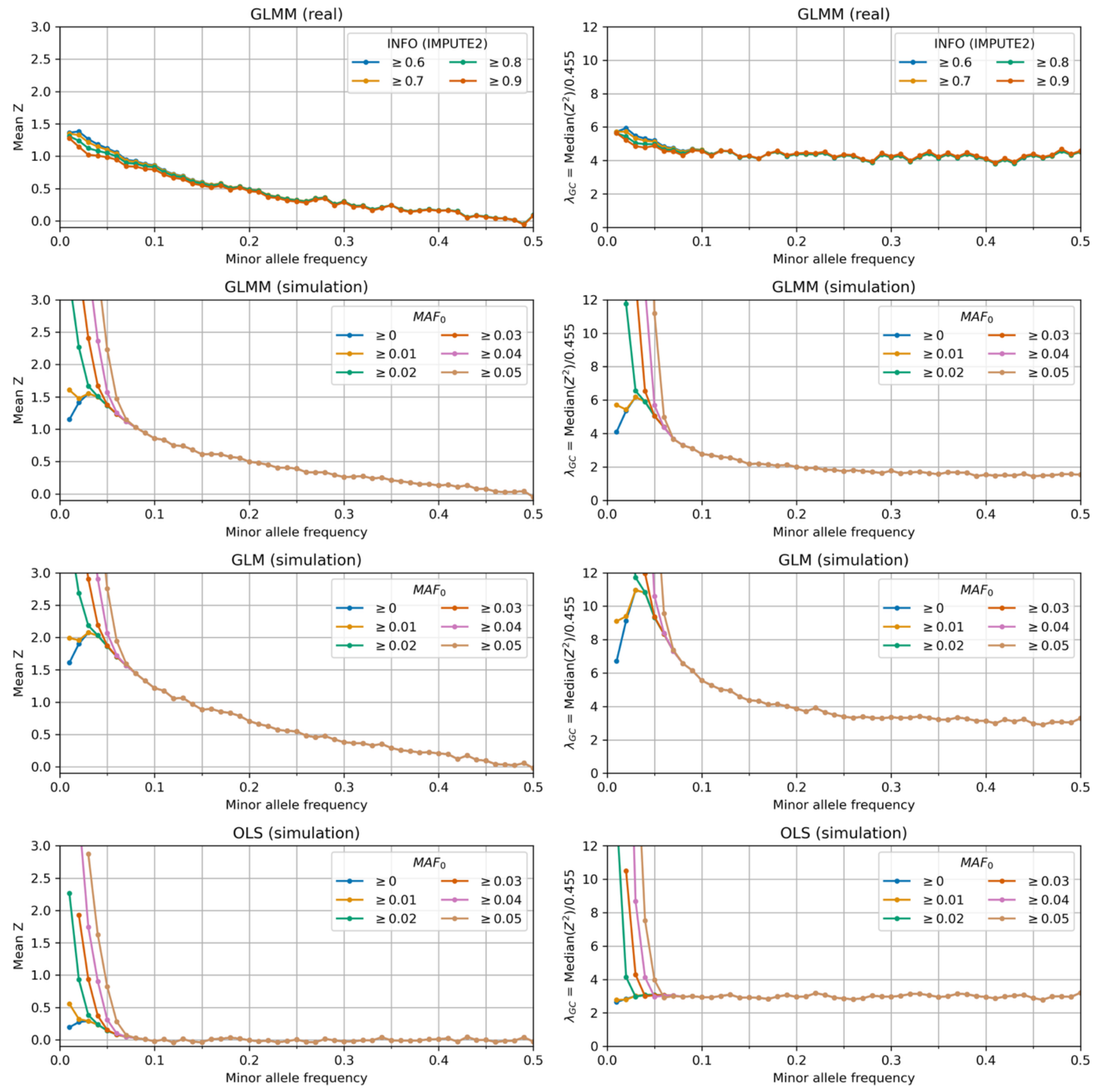

Figure S2.13: Frequency-dependent bias and inflation in z-scores. The left panels show mean Z-scores and the right panels show genomic control inflation factors (λ_{GC}) as a function of minor allele frequency in the whole time transect, stratified by imputation quality score (IMPUTE2's INFO score) for real data (top row) and minor allele frequency at time 0 (MAF_0) for simulation (second-to-fourth rows). GLMM and GLM, with a logit transformation, display stronger frequency-dependent bias and inflation at low frequencies compared to a linear regression (OLS), indicating that part of the distortion arises from the nonlinear logit transformation. Stratification by MAF_0 further shows that ascertainment based on higher MAF_0 in a modern reference panel, driven by imputation quality dependence on MAF_0, adds additional apparent inflation and bias. For the GLM and OLS we use the top 3 PC's as covariates. Simulations are adapted from Model 2.1 and run in SLiM on a random 100-kbp region of the genome, generating ~3.6 million SNPs from 20,000 replicates without directional selection.

Older sweeps in ancestral populations

To assess the effect of older selective sweeps in ancestral populations that are no longer under selection during the time transect, we leverage Experiment 1. Specifically, we generate four extreme scenarios by introducing a SNP fixed (p=1) in one of the four ancestral populations (CHG, ANA, EHG, WHG) and absent (p=0) in the other three. For each individual i, the expected allele frequency (p_i) is calculated based on ancestry proportions shown in Figure S2.1. Genotypes are then simulated by sampling from a binomial distribution, $G_i \sim \text{Binomial}(n=2, p=p_i)$. Each scenario is repeated 100 times; none yielded a p-value below the Bonferroni-corrected threshold of 1.25×10^{-4}. These results confirm that the GLMM does not capture signals from ancient sweeps that are no longer subject to selection in the studied time transect. For the significance threshold $|X| > 5.45$, the false-positive rate in this experiment is zero.

Detecting directional polygenic selection

For a trait under directional selective pressure, trait-increasing alleles are expected to shift in frequency to move the trait closer to its optimal value. We use three statistics to detect directional polygenic selection: two tests based on linear mixed models applied to two variations of polygenic scores, and one test based on the genetic correlation between selection and GWAS summary statistics (see Methods). We conduct 400 Model 1 simulations, with 200 under directional selection and 200 negative controls without directional selection. The performance of these methods is summarized in Table S2.2.

Linear mixed model on polygenic scores

We use two tests based on fitting a linear mixed model on polygenic scores, for two variations of polygenic score, to capture signals of directional polygenic selection referred to as Z_γ and $Z_{\gamma_{sign}}$ (see Methods). To adjust for residual inflation in the estimated Z_γ and $Z_{\gamma_{sign}}$, we correct the Z-scores using an inflation factor (λ_{IF}). Simulations suggest that this adjustment is not necessary, as the LMM model is already conservative and shows no sign of increased false positives due to inflation, with false positive rates of 1% for Z_γ and 0% for $Z_{\gamma_{sign}}$ (Table S2.2). However, we adopt the more conservative approach with the adjustment, as our simulation does not capture all the complexities of the real-world data.

To calculate the polygenic scores, we use the clumping procedure in PLINK 1.9[34] and clump variants using parameters clump-r2 = 0.05 and clump-kb = 500, prioritizing variants with smaller GWAS P-values. We use GEMMA (v0.98.5)[35] to fit the linear mixed model (see Methods).

Genetic correlation between trait and selection

The third test is based on the genetic correlation between selection summary statistics estimated from the GLMM model and GWAS estimates. The genetic correlation-based test (Z_{r_s}) is calculated using LDSC[33] using 10 blocks for jackknife estimates. In cases where heritability estimates of the

selection summary statistics are not significantly larger than zero, the methods reports a null value which is interpreted as a negative case in this experiment.

Table S2.2: Power analysis of tests for directional polygenic selection based on 400 Model 1 simulations, including 200 under an experimental condition with directional selection and 200 negative controls without directional selection.

Polygenic test	Bonferroni corrected (P < 1.25e-4)		Nominal (P < 0.05)	
	True positive (%)	False positive (%)	True positive (%)	False positive (%)
Z_{γ}	99	0	99.5	1
$Z_{\gamma_{sign}}$	89.5	0	99.5	0
$Z_{\gamma}/\sqrt{\lambda_{IF}}$	73.5	0	99.5	0.5
$Z_{\gamma_{sign}}/\sqrt{\lambda_{IF}}$	51	0	98.5	0
Z_{r_s}	53	0	66.5	0.5

Supplementary Information section 3
Statistical criterion for genome-wide significance

Establishing the Optimal Significance Threshold

When the null distribution is normal, significance thresholds are typically defined by controlling the family-wise error rate (FWER). In this study, however, the null distribution is not normal (Supplementary Information section 2), making FWER inappropriate for defining significance. For completeness, we evaluated several FWER-based approaches, and confirmed that, as expected from the non-normality, they were inefficient or not robust. Instead, we adopt an alternative strategy of controlling the false discovery rate (FDR), leveraging high-quality GWAS studies to guide significance thresholds.

Controlling for family wise error rate (FWER)

We explored various approaches to identify a control factor (CF) to adjust the nominal χ^2 of the selection signal that is both reliable and maximizes the utility of the data. We tried three different CF: genomic inflation factor (λ_{GC}), simulation based, and finally LD score regression intercept.

Genomic inflation factor (λ_{GC}). To adjust for residual confounding of our selection statistics, we tried adjusting with genomic inflation (λ_{GC}), defined as the median of the nominal χ^2 of the selection coefficient divided by the median of a chi-square distribution with 1 degree of freedom (0.455). This empirical correction factor is 4.75 for our dataset, excluding the HLA region. We tried using the λ_{GC} as the control factor for the nominal χ^2.The nominal genome-wide significance threshold, corresponding to an adjusted P-value threshold of 5×10^{-8} with CF=4.75, is $P=1.5\times10^{-32}$, which yields 49 independent loci, excluding the HLA region. By using orthogonal information (GWAS data) to estimate the fraction of the genome affected by directional selection, however, we infer that 45% of the genome is in at least weak linkage disequilibrium (LD) ($r^2 > 0.05$) with high-confidence signals of selection (<5% False Discovery Rate - FDR) (Extended Data Figure 2b). This is a much larger fraction of the genome shaped by directional selection signals than can be explained by 49 loci, and thus, relying on λ_{GC} is too conservative.

LD score and pseudo-intercept. "LD score regression" (LDSC) analyzes the relationship between the LD among variants and their associated test statistics to disentangle the effects of polygenicity from confounding. If it is working properly, it is expected to show a linear relationship between "LD score bin" and tests statistics whose y-intercept can be used to estimate inflation and is often in fact used in this way in GWAS studies. We explore this approach to correct for inflation in our selection scan.

A potential challenge in using LDSC to correct for inflation in a selection scan is that our null is not normally distributed (Supplementary Information section 2) and therefore we see a non-linear behavior in the LD score (Figure S3.1a). This cause the LDSC intercept to be heavily dependent on the SNPs with lower LD score which are usually at low frequency and also impacted by the

inflation due the non-normality of the null. Because of these issues, we cannot simply use the LDSC framework to determine an appropriate inflation factor correction.

However, we are mainly interested in the tail of the distribution, and because inflation depends on allele frequency (Figure S2.13) and thus LD score, we explore computing a pseudo-intercept using S-LDSC with baseline v2.2, where only SNPs with LD-scores above a threshold min(LD-score) are considered in the calculation of the intercept. The pseudo-intercept as a function of min(LD-score) decreases monotonically until min(LD-score) = 89, which gives a pseudo-intercept of 2.33, and then after that point, it fluctuates. The nominal genome-wide significance threshold, corresponding to an adjusted P-value threshold of 5e-8 with CF = 2.33, is 8.7×10^{-17}, yields 377 independent loci excluding the HLA region. The FDR for this threshold is estimated to be ~0%.

This CF from the pseudo-intercept is the most reasonable compared to previous approaches, assessed based on the independent FDR criterion, and in fact it is similar to the threshold we used in practice and which we obtained by calibration from orthogonal data (enrichment in signals of association to phenotypes from GWAS). However, because of the non-linearities, we refrain from using this correction to control formally for FWER.

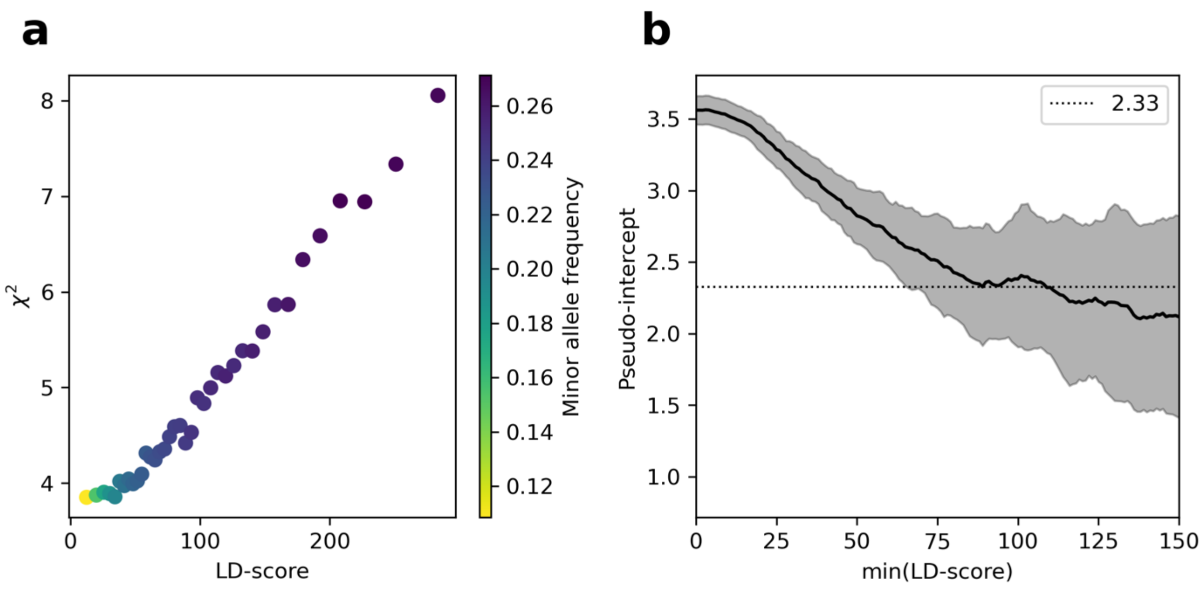

Figure S3.1: (**a**) LD score plot for nominal χ^2 statistics, with each point representing an LD score quintile. Values are averaged within each bin for visualization purposes only. (**b**) The pseudo-intercept is the intercept of the LD score regression after dropping all SNPs with LD scores below the minimum threshold.

Controlling for false discovery rate (FDR) by leveraging GWAS signals

In this study, for each variant, we compute a selection statistic for which the null distribution is not well approximated by a standard normal distribution due to frequency-dependent biases introduced by imputation and the non-linear link function of generalized linear mixed models, as explained in detail in Supplementary Information Section 2 (subsection: *Non-normality of the null distribution in GLMM test statistics*). In addition, a substantial fraction of the genome appears to be affected by real directional selection (Extended Data Figure 2), violating the assumption that most variants are null. For these reasons, conventional approaches such as genomic control or fixed theoretical significance thresholds are not appropriate in this setting. Therefore, we abandon controlling for the family-wise error rate (FWER), which is the probability of obtaining one or more false positives in a multiple testing scenario, and instead take advantage of information-rich, high-quality GWAS data to estimate the false discovery rate (FDR) and posterior probability, and thereby to calibrate our summary statistics.

If a variant is under selection, it must be biologically relevant and therefore functional. Suppose we have a binary functional annotation that labels SNPs as functional or nonfunctional. Variants truly under selection are expected to be functional, whereas variants not under selection are not necessarily functional. As a result, variants showing evidence of selection are expected to be enriched for functional annotation, with stronger and more reliable selection signals exhibiting higher enrichment due to fewer false positives. As confidence in the selection signal increases and the false-positive rate approaches zero, the enrichment is expected to plateau.

While such an annotation is not available, large-scale GWAS provide a practical proxy. Genome-wide significant GWAS associations are highly enriched for functional variants, as well as variants in linkage disequilibrium with them. We therefore leverage an external source of information that is independent of our selection study. We then examine how the proportion of variants overlapping this GWAS-derived binary annotation changes as a function of the absolute value of the selection statistic. At low statistic values, this proportion is close to the genome-wide baseline. As the statistic increases, the probability that a variant reflects a true signal of selection is expected to increase monotonically. This ordering assumption is reasonable and is supported both by realistic forward-in-time simulations (Figure 1, Extended Data Figure 4) and by empirical patterns observed in the data (Figure 1 and Extended Data Figure 5). Because true targets of selection are expected to be functionally relevant, increasing evidence for selection should correspond to increasing enrichment for functional annotations.

A key empirical observation is that this enrichment does not increase indefinitely. Instead, it rises and then reaches a clear plateau. This plateau indicates that beyond a certain statistic threshold, the additional variants included have a stable functional composition. If a non-negligible fraction of false positives were still present at higher statistic values, enrichment would be expected to continue increasing rather than stabilize. Forward-in-time simulations confirm that under directional selection, the false discovery rate becomes negligible in the extreme tail of the statistic, whereas null models do not produce this behavior (Figure 1, Extended Data Figure 4).

We therefore identify the onset of this plateau as an empirical threshold for genome-wide significance. This approach does not require specifying a parametric model for the null distribution and relies on the assumption, supported by simulations and empirical data, that the false discovery rate is close to zero in the extreme tail of the statistic (Figure 1, Extended Data Figure 4). We rescale the raw test statistic so that this empirically determined threshold aligns with the conventional genome-wide significance level used in GWAS, yielding a calibrated statistic whose threshold corresponds to a regime dominated by true signals of directional selection. We then estimate the false discovery rate by interpreting the stabilized enrichment as reflecting the asymptotic proportion of true positives in this regime.

In this section, our goal is not to assess colocalization between selection signals and specific GWAS hits, nor to perform fine-mapping or assign biological interpretation to individual overlaps. Instead, we use GWAS associations across many traits to define a broad external genomic annotation enriched for functional variation, and we leverage enrichment of this annotation to empirically calibrate genome-wide statistical significance for our selection statistic in a setting where standard null assumptions do not hold. GWAS results are used solely as an external functional annotation, independent of any locus-level interpretation. This annotation is constructed from genome-wide significant associations across 454 high-quality GWAS from the Pan-UK Biobank and encompasses 12.5% of the variants analyzed here. Ignoring adjustment for minor allele frequency effects, the maximum attainable enrichment is bounded by 8, highlighting the strength of the enrichment observed in Figure 1a.

In Figure S3.2, the x-axis is the nominal p-value of the selection coefficient, and the left y-axis is the enrichment in pan-UKBB GWAS studies. To estimate the enrichment values, ultimately determining FDR and the posterior probability of being a true selection signal, we follow the procedure outlined below.

First, for all SNPs that pass the quality control, we apply a pruning procedure using PLINK (version 1.9) with an r^2 threshold of 0.99 and a window size of 1 Mbp. This results in 4,033,881 SNPs, of which 12.5% are genome-wide significant in at least one of 454 GWAS from the Pan-UKBB that pass QC.

To adjust for minor allele frequency variation, we initially apply a logistic regression model to this set of SNPs:

$$log\left(\frac{p_i}{1-p_i}\right) = a + bf_i$$

where p_i is the expected probability that SNP i is a GWAS hit given its minor allele frequency f_i.

For each threshold for significance t, we define $R(t)$ as:

$$R(t) = \frac{1}{N(t)}\sum_{i\in S_t}\frac{y_i}{p_i}$$

Where $S_t=\{i: P_i \leq 10^{-t}\}$ and $N(t)$ is number of SNPs in set S_t. Subsequently, we define $\alpha_0=R(0)$, and $E(t)=R(t)/R(0)$. We rewrite E(t) in the following form:

$$E(t) = \frac{\int_{x=t}^{\infty}\left[n(x)p(\text{real}|x)\alpha_1 + \left(1 - p(\text{real}|x)\right)n(x)\alpha_0\right]dx}{(\alpha_1 - \alpha_0)\int_{x=t}^{\infty} n(x)dx}$$

where $n(t) = \frac{-\partial N(t)}{\partial t}$ and $p(\text{real}|t)$ is the posterior probability of being a true selection signal.

Thus,

$$p(\text{real}|t) = \frac{\partial\left(E(t)N(t)\right)}{\partial\left(N(t)\right)}$$

The false discovery rate *FDR(t)* is defined as:

$$FDR(t) = 1 - \frac{E(t) - 1}{\lim_{t\to\infty} E(t) - 1}$$

To estimate *E(t)* and *N(t)* smoothly, we utilize the polyfit function from the numpy package in Python. Our estimations for values approaching 0 or 1 tend to be less reliable. Consequently, we refrain from reporting values below 1% or above 99%.

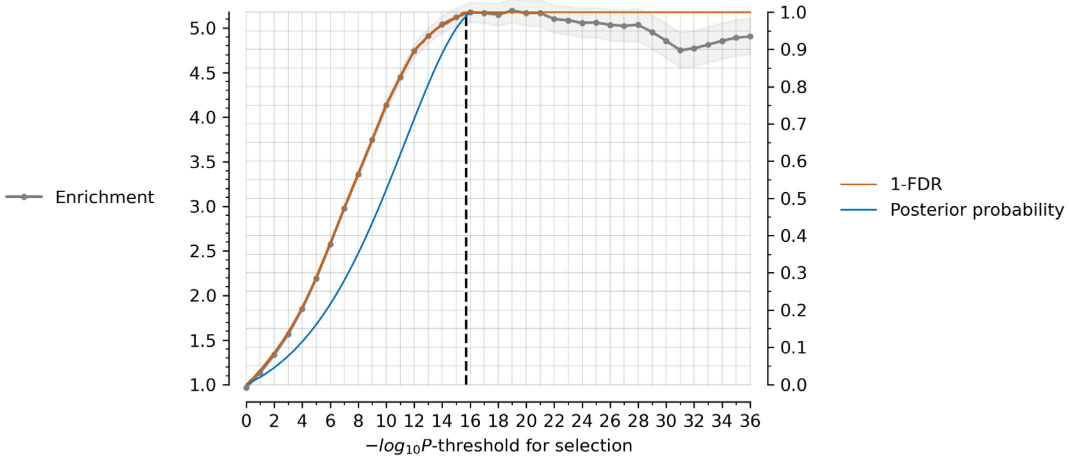

Figure S3.2: Estimated FDR and posterior probability of being a true selection signal as a function of the nominal p-value threshold. The black dashed line shows the P-threshold (1.95e-16) with a posterior probability of 99%.

This analysis suggests that at a nominal p-value threshold of 1.95e-16, the posterior probability is ~99%. The appropriate threshold for controlling FWER should be somewhere between 8.7e-17 and 8.2e-33 based on the discussion above. However, the 8.2e-33 threshold seems extremely

conservative, while the enrichment in GWAS studies gives us a good handle on FDR and makes the most out of the data, and hence we use the FDR approach.

We implement the FDR approach in practice using a newly defined statistic $X = Z/\sqrt{CF}$, where Z is the nominal test statistic for the selection signal without full correction for inflation, and CF is an empirically calibrated correction factor. We set CF=2.28 ($\sqrt{CF} = 1.51$) based on this being the point at which we estimate that the posterior probability is 99%.

With our parameterization of the X-statistic, the threshold |X|=5.45 which if it was a normally distributed variable would imply a classically genome-wide significance threshold of $5x10^{-8}$, is the threshold of genome-wide significance for our study as well. Thus, p-values obtained from interpreting our X-score as a normally distributed variable provide reasonable guidelines for whether particular SNPs are genome-wide significant.

Supplementary Information section 4

HAF score analysis provides evidence for directional selection

HAF score dynamic for positive and negative selection

The haplotype allele frequency (HAF) score for a given haplotype is calculated by summing the derived allele counts of the polymorphic sites on that haplotype[36,37]. It distinguishes carrier haplotypes from non-carriers of the favored allele in an ongoing selective sweep, without prior knowledge of the favored allele. The HAF-score is defined for a haploid population, requiring phased haplotype information and ancestral and derived allelic states to distinguish carriers from non-carriers of the favored mutation in an ongoing selective sweep. For a diploid population, however, calculating the mean HAF-score does not require phased information, and only the derived allele frequency (DAF) is needed. The mean HAF-score is given by:

$$\overline{HAF} = n \sum_{i=1}^{m} DAF_i^2$$

where n is the number of haplotypes (twice the number of diploid individuals), m the number of polymorphic sites in the sample, and DAF_i the derived allele frequency for the i-th polymorphic site.

In a neutrally evolving population with a constant population size N, the expected HAF score under the coalescent model[38] is:

$$E[HAF] = \frac{\theta(n-1)}{2}$$

where $\theta = 2N\mu L$ represents the scaled mutation rate, μ is the mutation rate per base pair per generation, L is the haplotype length, and n is the number of sampled haplotypes.

For strong selection ($Ns \gg 1$) without recombination, the expected HAF scores for carrier and non-carrier haplotypes of a favored allele with frequency f during a hard selective sweep are given by[36]:

$$E[HAF^{car}] \approx \theta n \left(\frac{f+1}{2} - \frac{1}{(1-f)n+1}\right)$$

$$E[HAF^{non}] \approx \theta n \left(\frac{1}{2} + \frac{1}{2n} - \frac{1}{(1-f)n+1}\right)$$

Thus, the expected HAF score for n haplotypes undergoing a hard selective sweep is:

$$E[HAF] = f \times E[HAF^{car}] + (1-f) \times E[HAF^{non}] \approx \theta n \left(\frac{f^2+1}{2} + \frac{1-f}{2n} - \frac{1}{(1-f)n+1} \right)$$

When $n(1-f)f > (2-f)$, E[HAF] is greater than $\theta(n-1)/2$, indicating that the expected HAF score is larger than the neutral case if the sweep is not near fixation (Figure S4.1). Thus, a positive deviation from expectation provides evidence of a partial sweep.

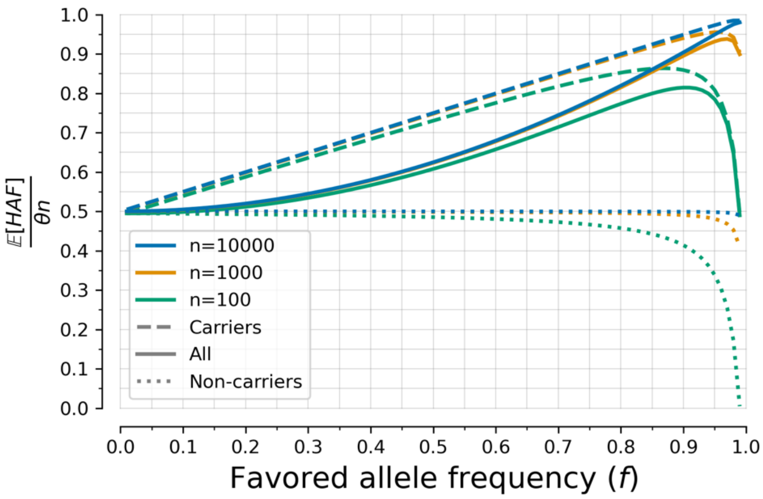

Figure S4.1: Haplotype allele frequency (HAF) score as a function of favored allele frequency during an ongoing hard selective sweep without recombination.

For background selection, the effective population size (N) is reduced due to purifying selection against linked variation. This leads to a decrease in θ and the expected HAF score, making it lower than in the neutral case[14]. Thus, in principle, observation of a rise in the HAF score can provide unambiguous evidence of positive directional selection associated with our X-statistic.

A challenge in testing for evidence of direction selection based on a rise in the HAF score is that alleles in the genome that are subject to positive selection are also expected to be regions rich in functional effects, and thus are expected to be more subject to purifying selection. When we compute how HAF score changes as a function of our test statistic for natural selection, we observe a nominal decrease in HAF score—not the increase expected for positive selection—which could be due to this phenomenon (Extended Data Figures 3b,c). To distinguish between scenarios in which the correlation of our X-statistic to functionally more important regions is due to the signal we are interested in (that is, directional selection), and cannot be trivially explained as an artifact of background selection, we controlled for the empirically estimated effects of background selection (McVicker-B, Murphy-phastCons, Murphy-CADD, number of SNPs, and total heterozygosity in a 200 kb window) genome-wide. The resulting residual HAF-score increases as a function of X-statistic, beginning to rise just before |X|=5.45, replicating the behavior observed in simulations (Figures 1c, 1d, Extended Data Figure 3c)

Supplementary Information section 5

Allele frequency trajectory and selection coefficient over time for 479 independent loci with >99% probability of selection

In this section, we visualize allele frequency trajectories (Figure S5.1-S5.40) and selection coefficient over time (Figure S5.41-S5.80) for 479 independent loci (410 outside the HLA region and an additional 69 in HLA) with $|X|>5.45$ corresponding to a $\pi>99\%$ probability of selection. To produce this list, we identified the strongest signal in the genome and considered all SNPs in LD with it in modern Europeans from the 1000 Genomes Project ($r^2>0.05$) to potentially reflect the same signal. We then found the second-strongest signal excluding these positions, and so on, until no more SNPs pass this threshold (Extended Data Figure 2b). The SNPs are listed in genomic order.

We applied an additional pruning step to the 479 candidate SNPs identified after the initial clumping based on r^2. In this step, we calculated D′ among the candidate SNPs within a specified window size, prioritized variants by their GLMM p-values, and recursively designated tag SNPs. Any variant with D′ above a given threshold relative to a tag SNP was pruned. This process was repeated until all remaining variants were retained as tag SNPs or pruned. Thresholds for D′ were set at 1, 0.9, 0.5, 0.2, and 0.05, and window sizes were set at 100 kbp, 200 kbp, 500 kbp, 1 Mbp, 2 Mbp, 5 Mbp, and 10 Mbp. Table S5.1 reports the number of SNPs retained and pruned at each D′ threshold, separately for the HLA region and outside the HLA region and for each window size.

**Table S5.1:** Number of candidate SNPs after D′ based pruning.							
**Window size**	**Region**	**Status**	**Number of candidate SNPs**				
			**D′ ≤ 1**	**D′ ≤ 0.9**	**D′ ≤ 0.5**	**D′ ≤ 0.2**	**D′ ≤ 0.05**
**100 kbp**	Outside HLA	Retained	410	392	383	374	372
		Pruned	0	18	27	36	38
	Inside HLA	Retained	69	41	35	31	29
		Pruned	0	28	34	38	40
**200 kbp**	Outside HLA	Retained	410	386	373	358	351
		Pruned	0	24	37	52	59
	Inside HLA	Retained	69	35	26	23	20
		Pruned	0	34	43	46	49
**500 kbp**	Outside HLA	Retained	410	378	345	324	313
		Pruned	0	32	65	86	97
	Inside HLA	Retained	69	31	16	11	10
		Pruned	0	38	53	58	59
**1 Mbp**	Outside HLA	Retained	410	373	329	301	285
		Pruned	0	37	81	109	125
	Inside HLA	Retained	69	25	12	4	4
		Pruned	0	44	57	65	65
**2 Mbp**	Outside HLA	Retained	410	371	324	280	252
		Pruned	0	39	86	130	158
	Inside HLA	Retained	69	21	8	3	2
		Pruned	0	48	61	66	67
**5 Mbp**	Outside HLA	Retained	410	370	315	259	207
		Pruned	0	40	95	151	203
	Inside HLA	Retained	69	20	4	1	1
		Pruned	0	49	65	68	68
**10 Mbp**	Outside HLA	Retained	410	369	312	245	161
		Pruned	0	41	98	165	249
	Inside HLA	Retained	69	15	3	1	1
		Pruned	0	54	66	68	68

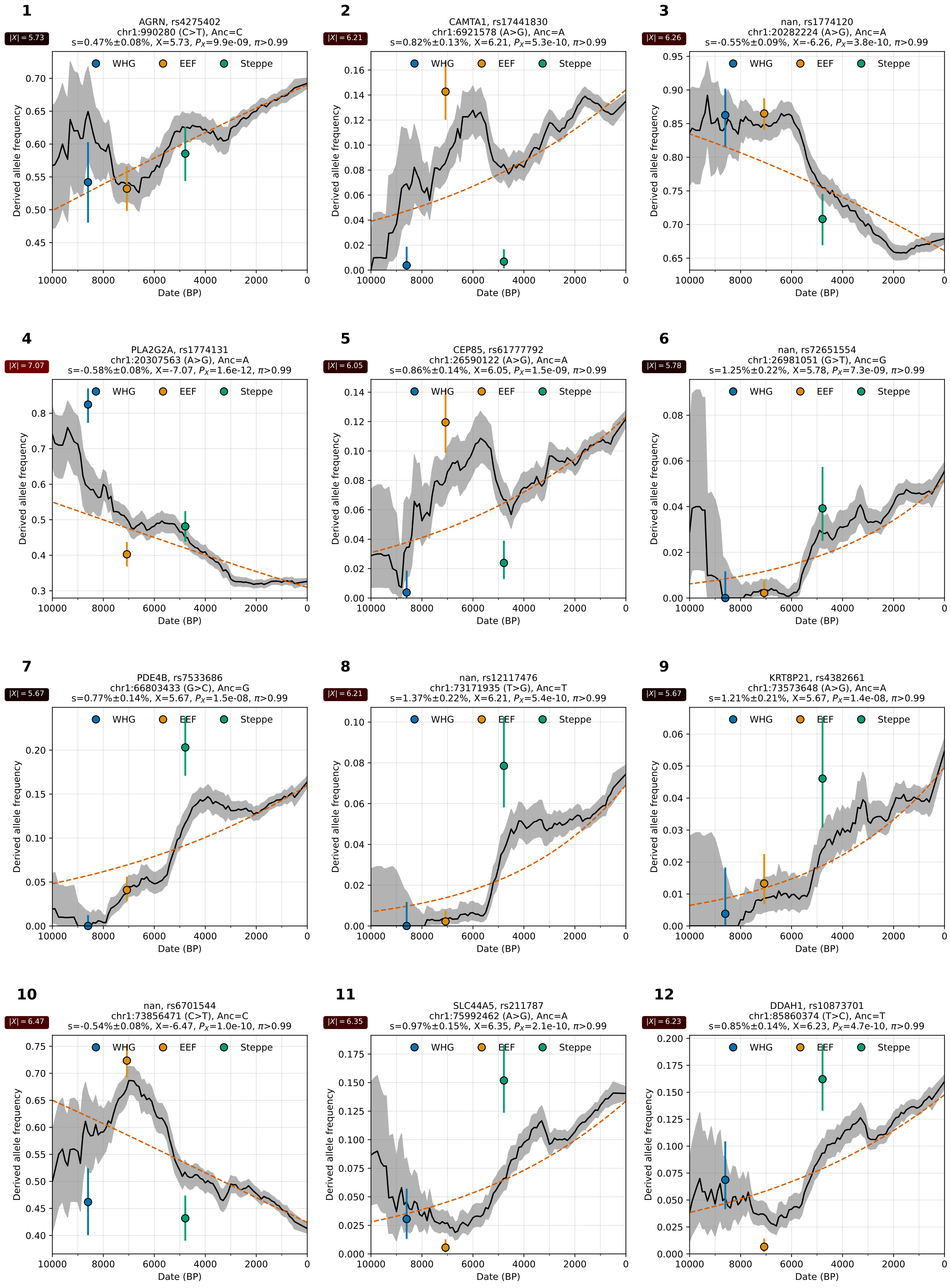

Supplementary Figure S5.1: Allele frequency over time.

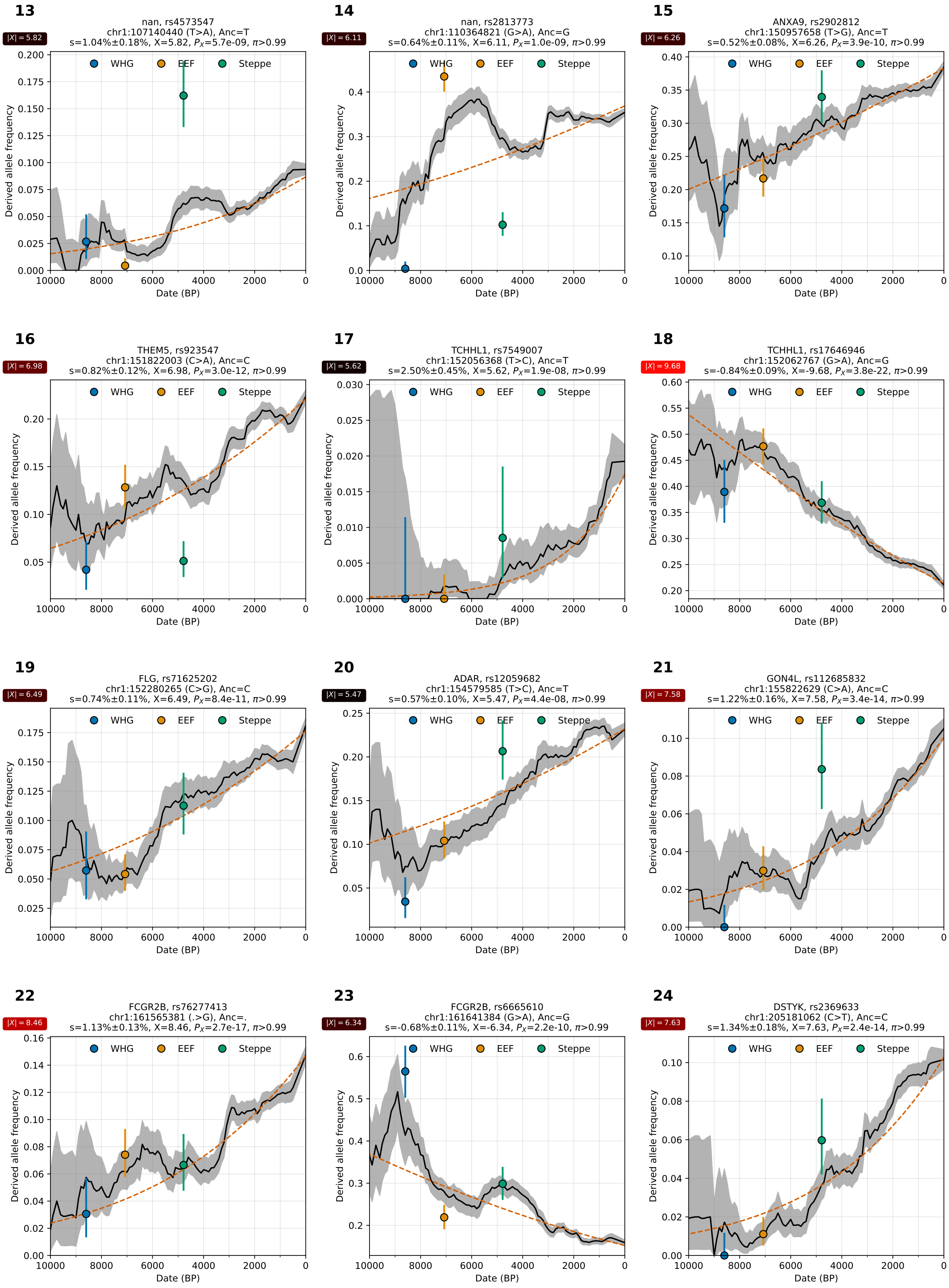

Supplementary Figure S5.2: Allele frequency over time.

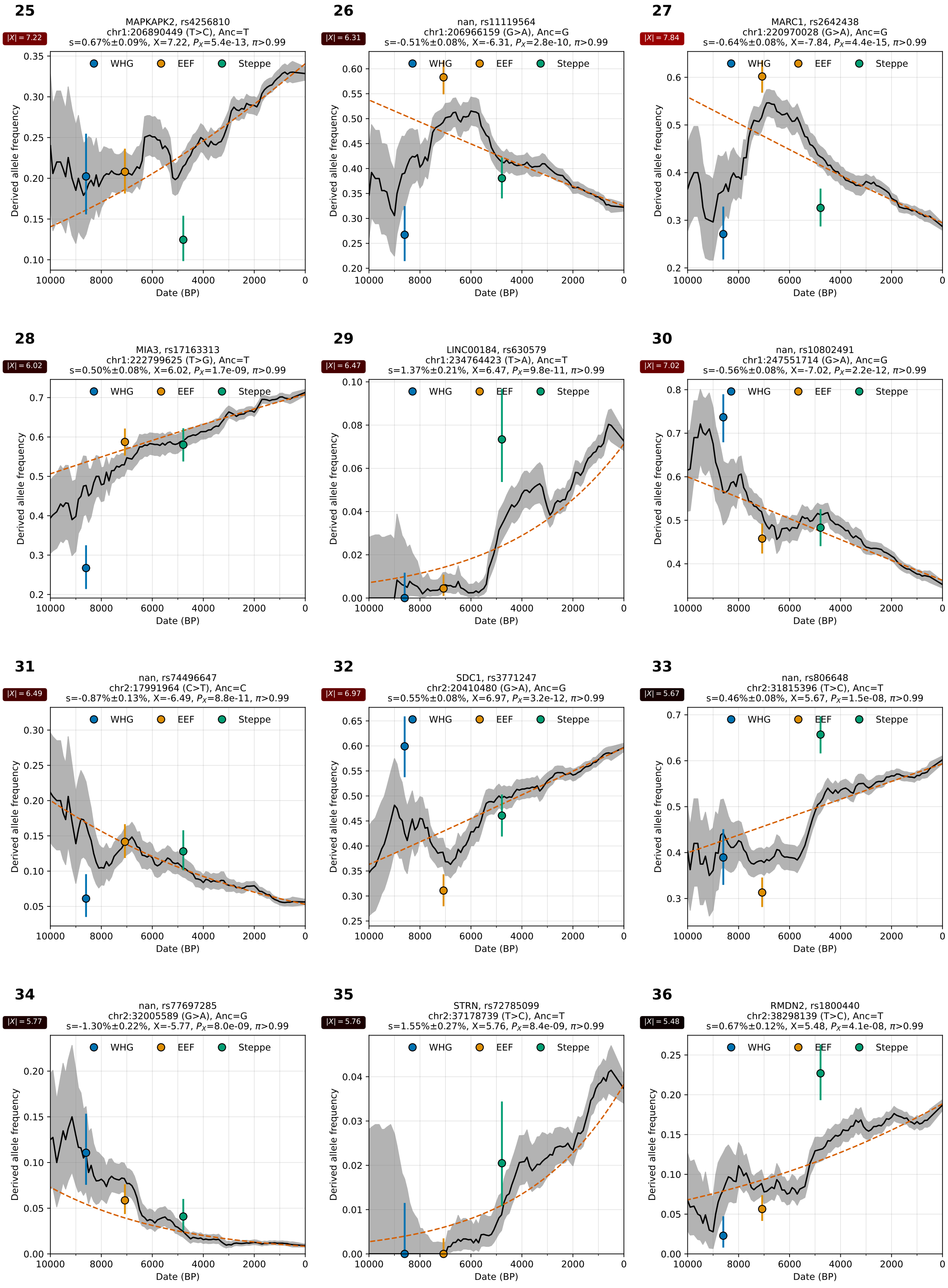

Supplementary Figure S5.3: Allele frequency over time.

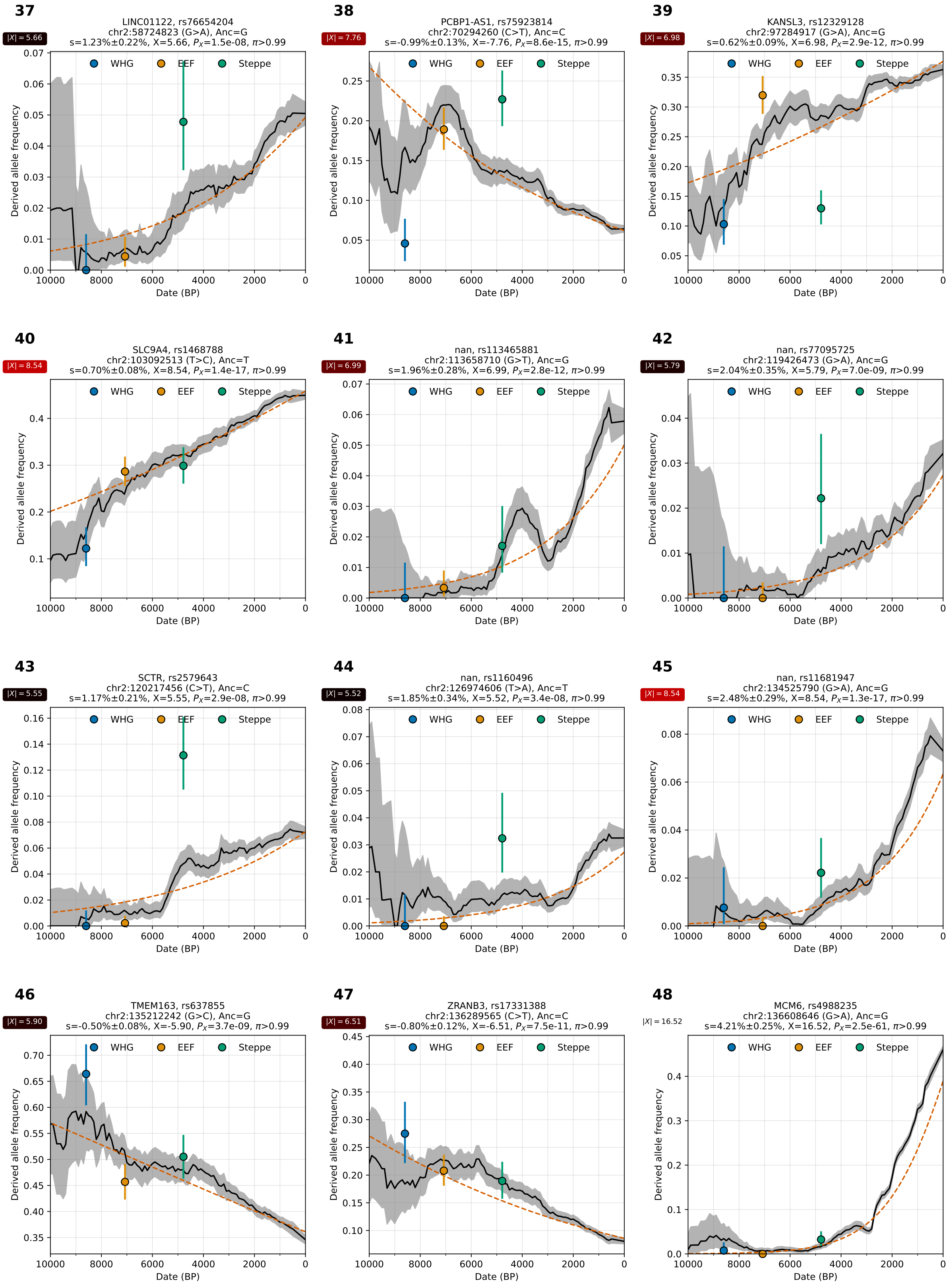

Supplementary Figure S5.4: Allele frequency over time.

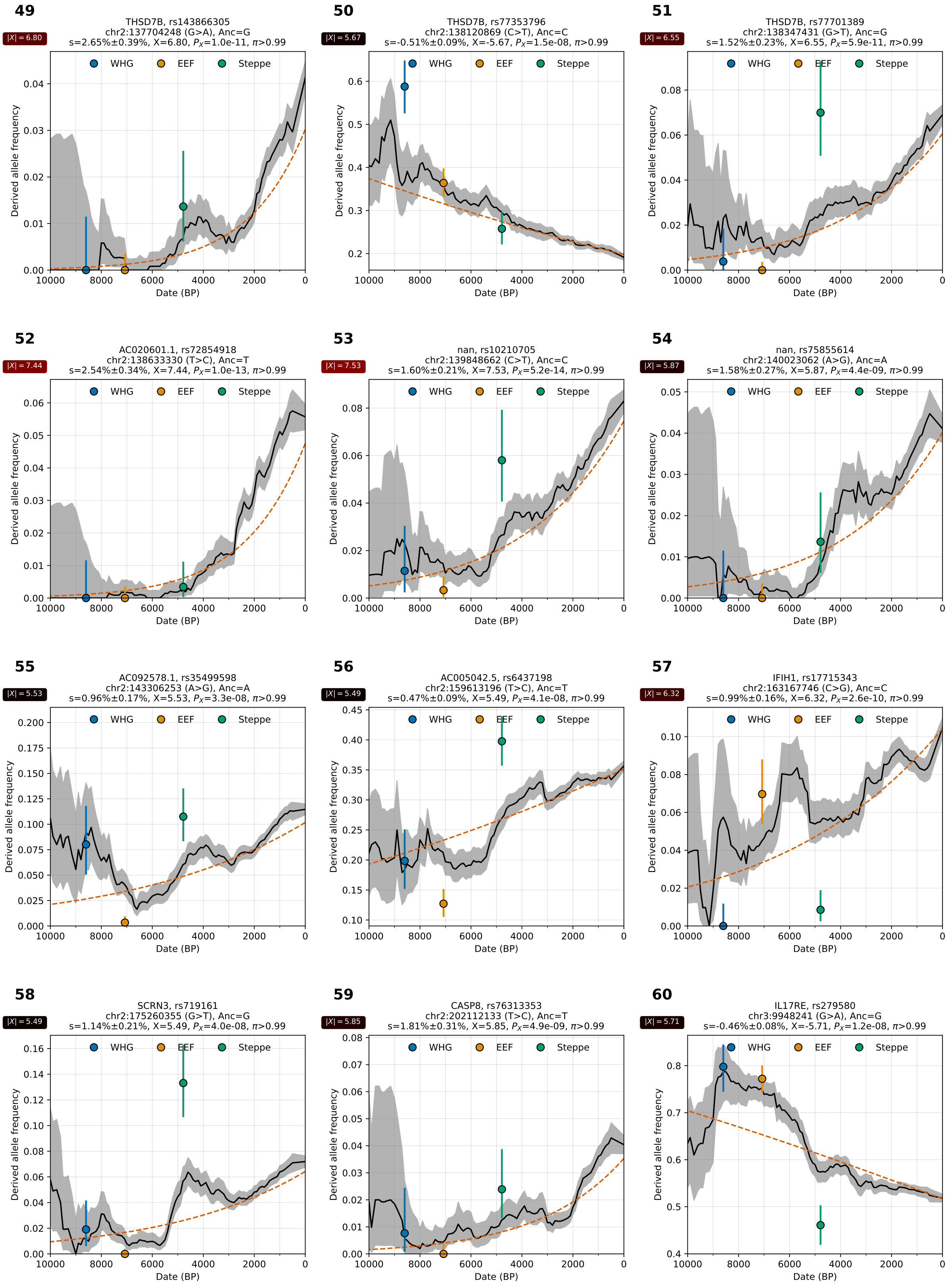

Supplementary Figure S5.5: Allele frequency over time.

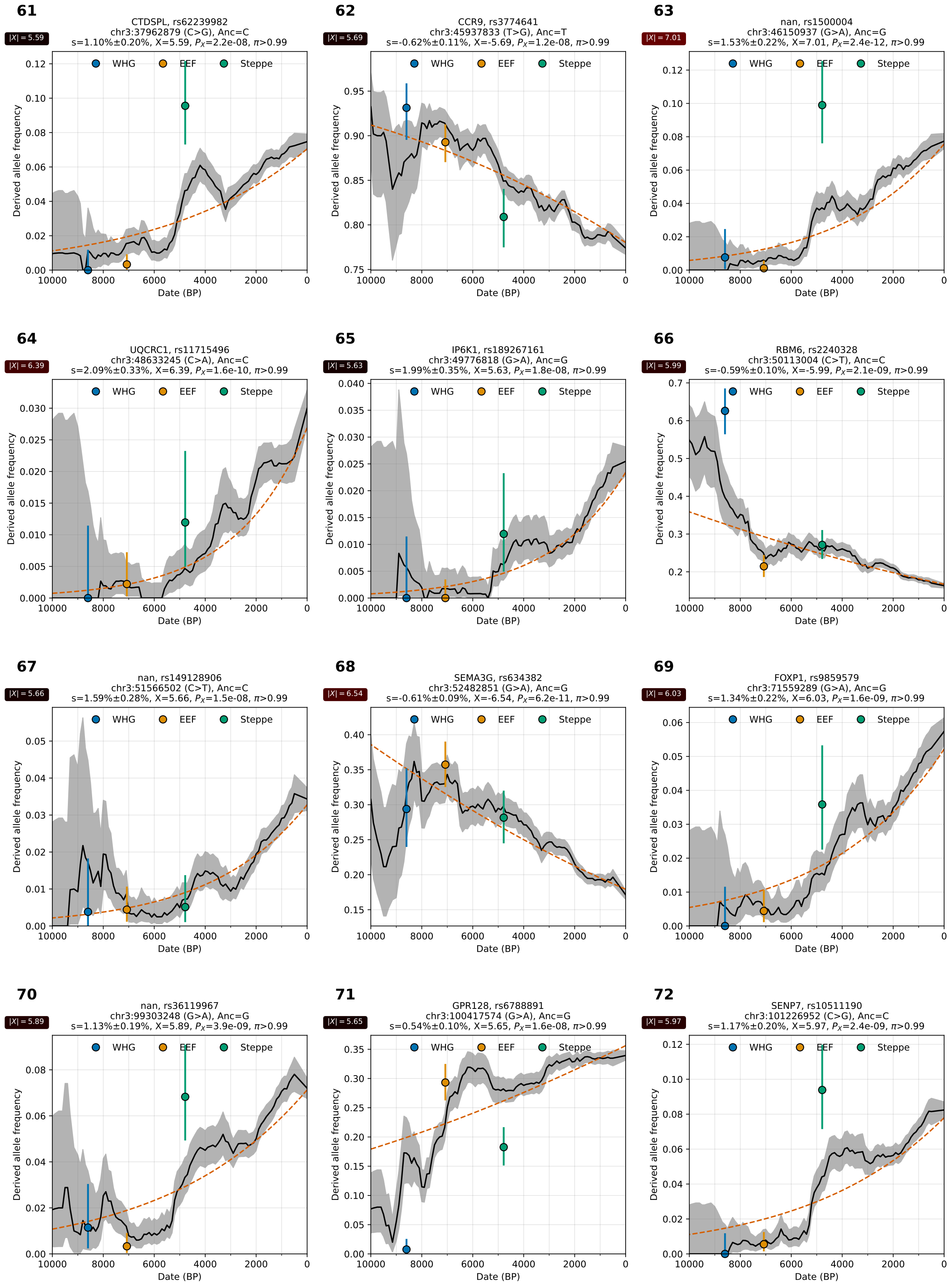

Supplementary Figure S5.6: Allele frequency over time.

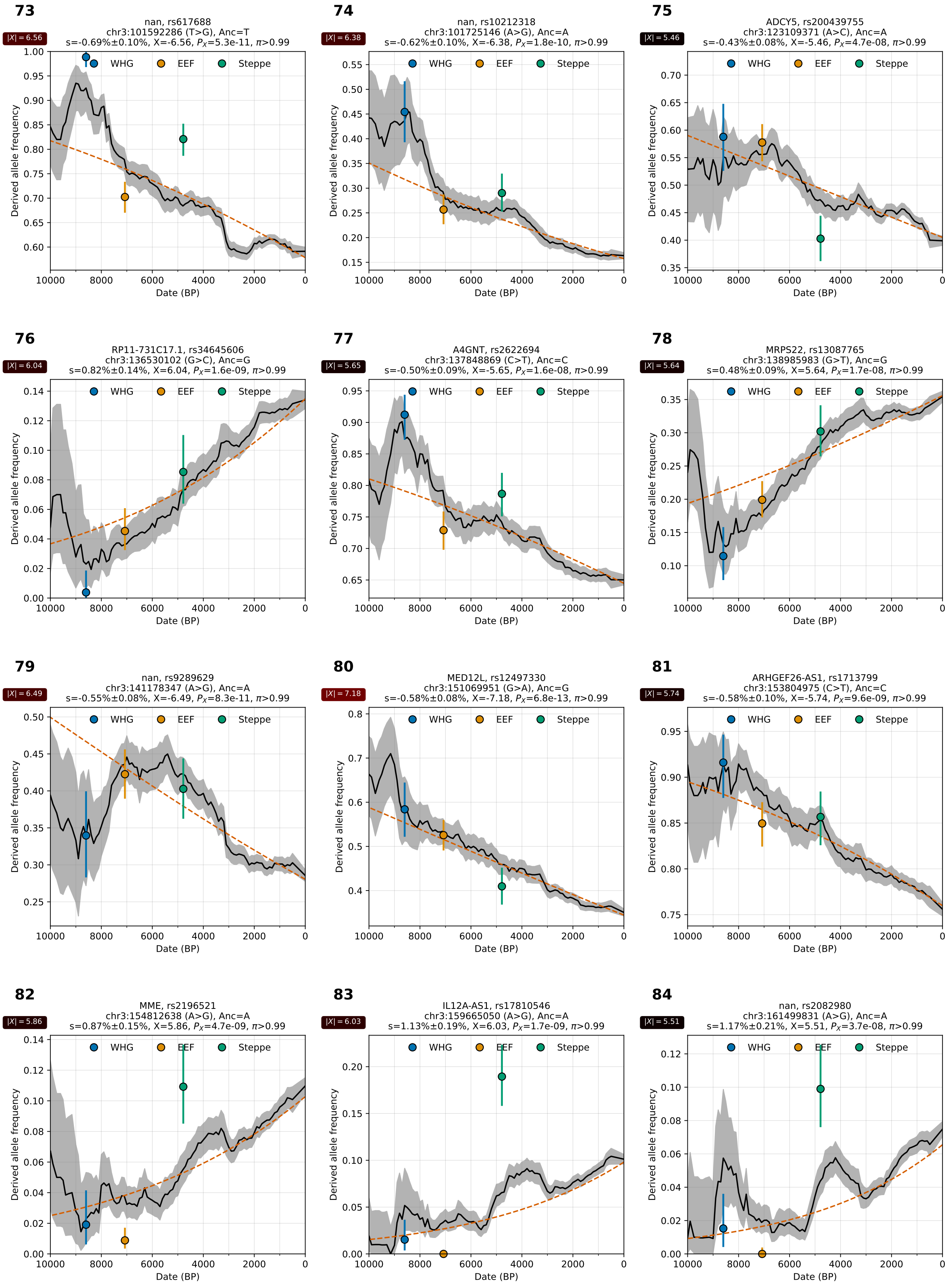

Supplementary Figure S5.7: Allele frequency over time.

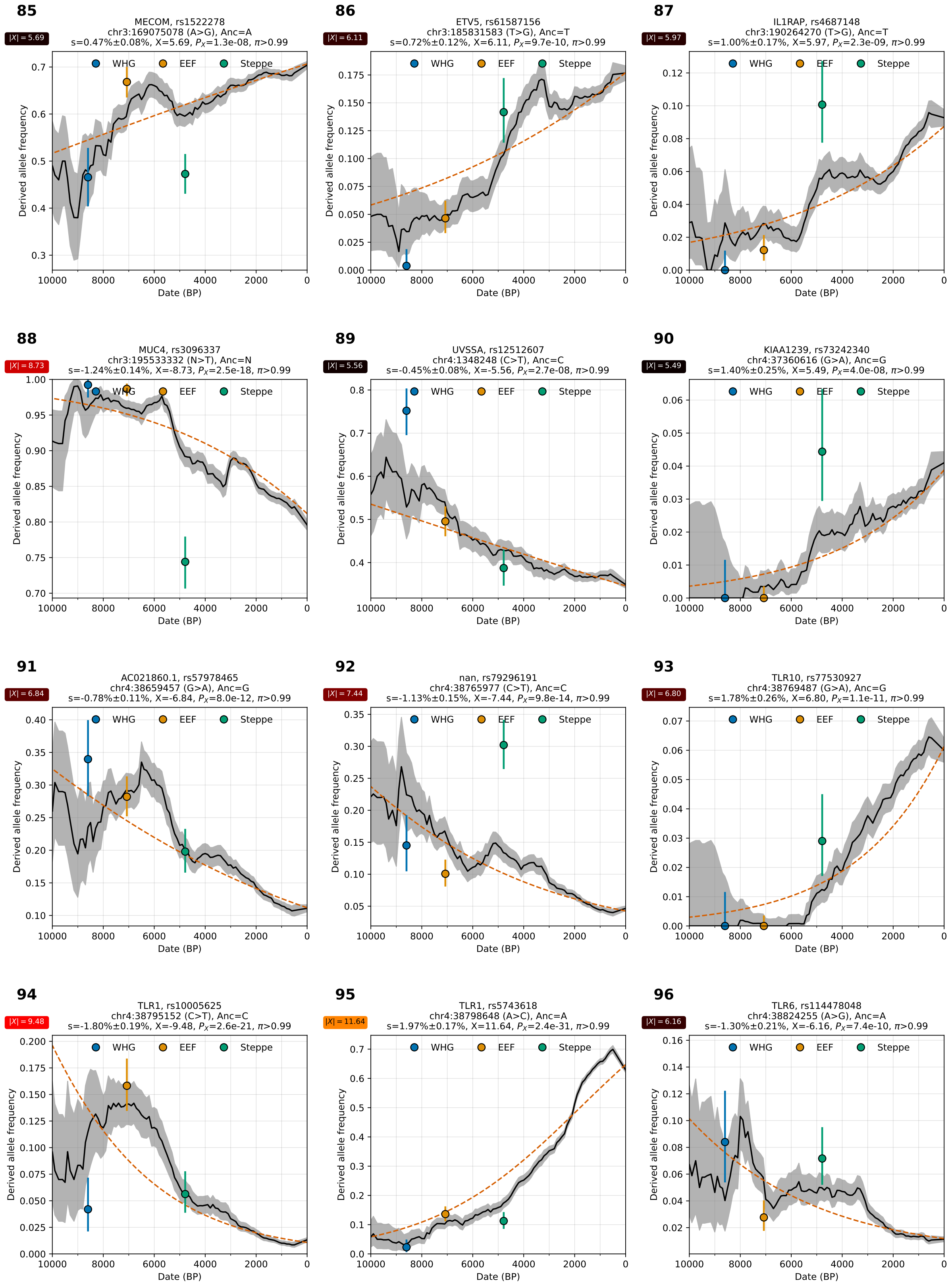

Supplementary Figure S5.8: Allele frequency over time.

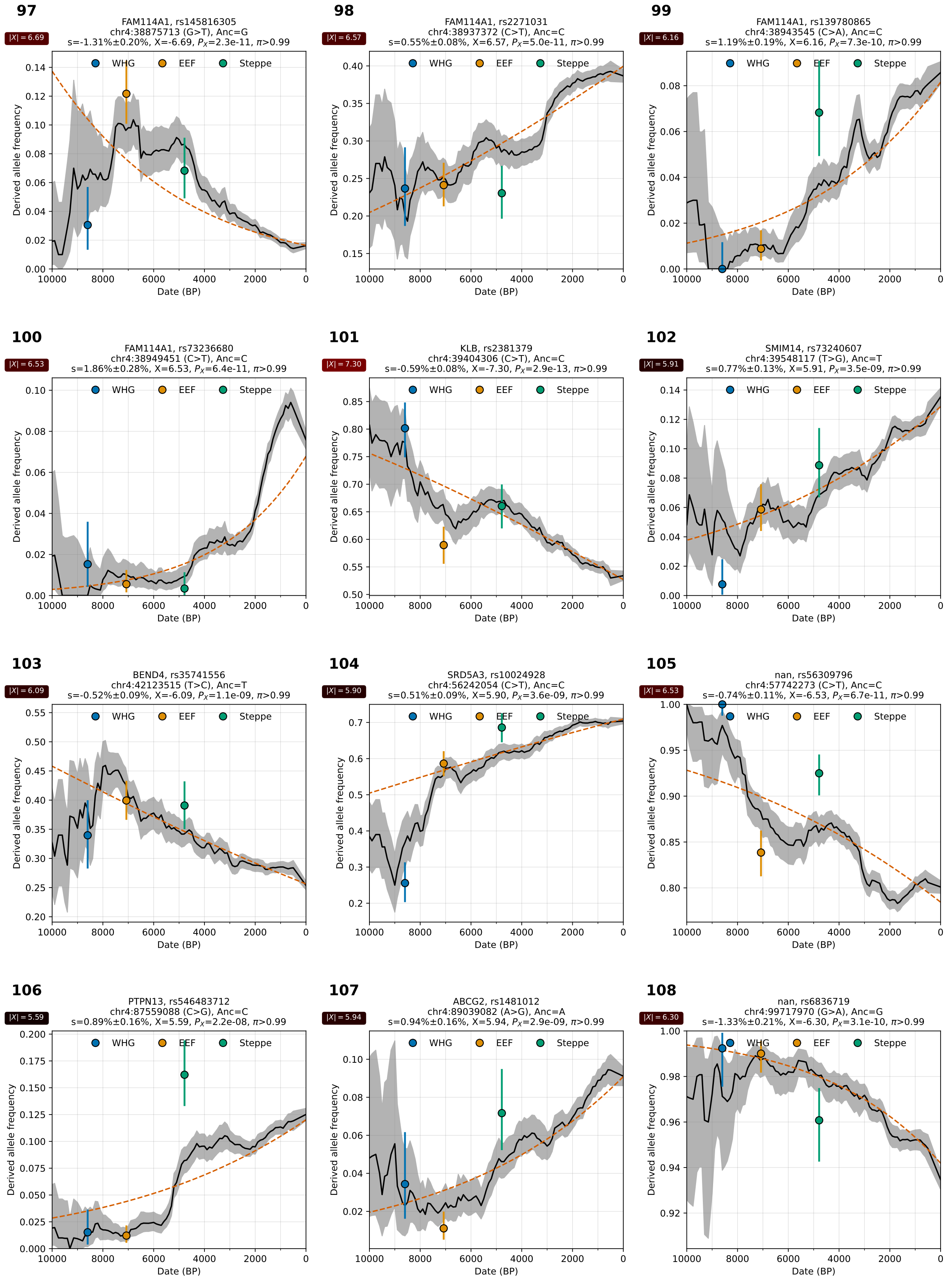

Supplementary Figure S5.9: Allele frequency over time.

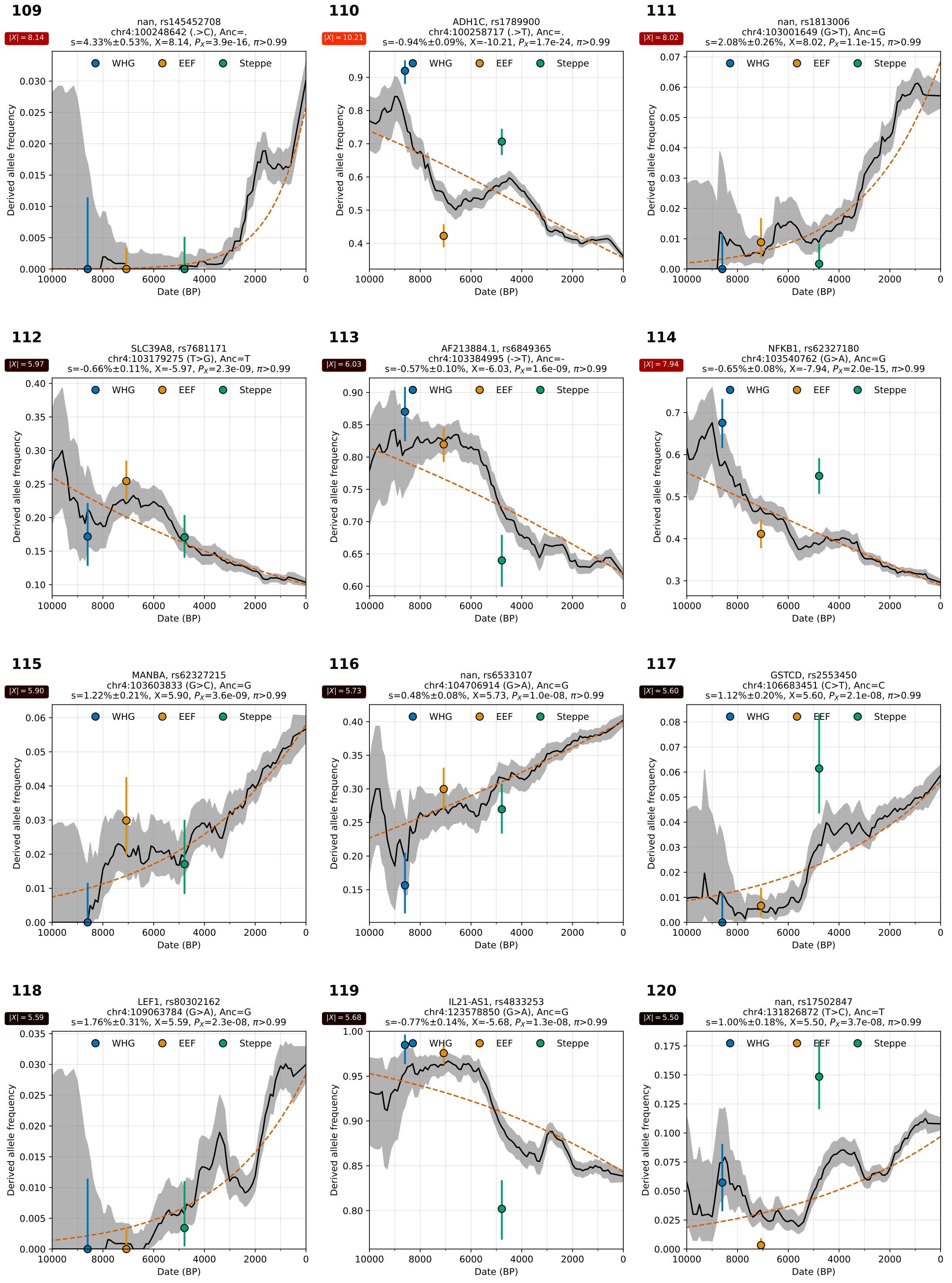

Supplementary Figure S5.10: Allele frequency over time.

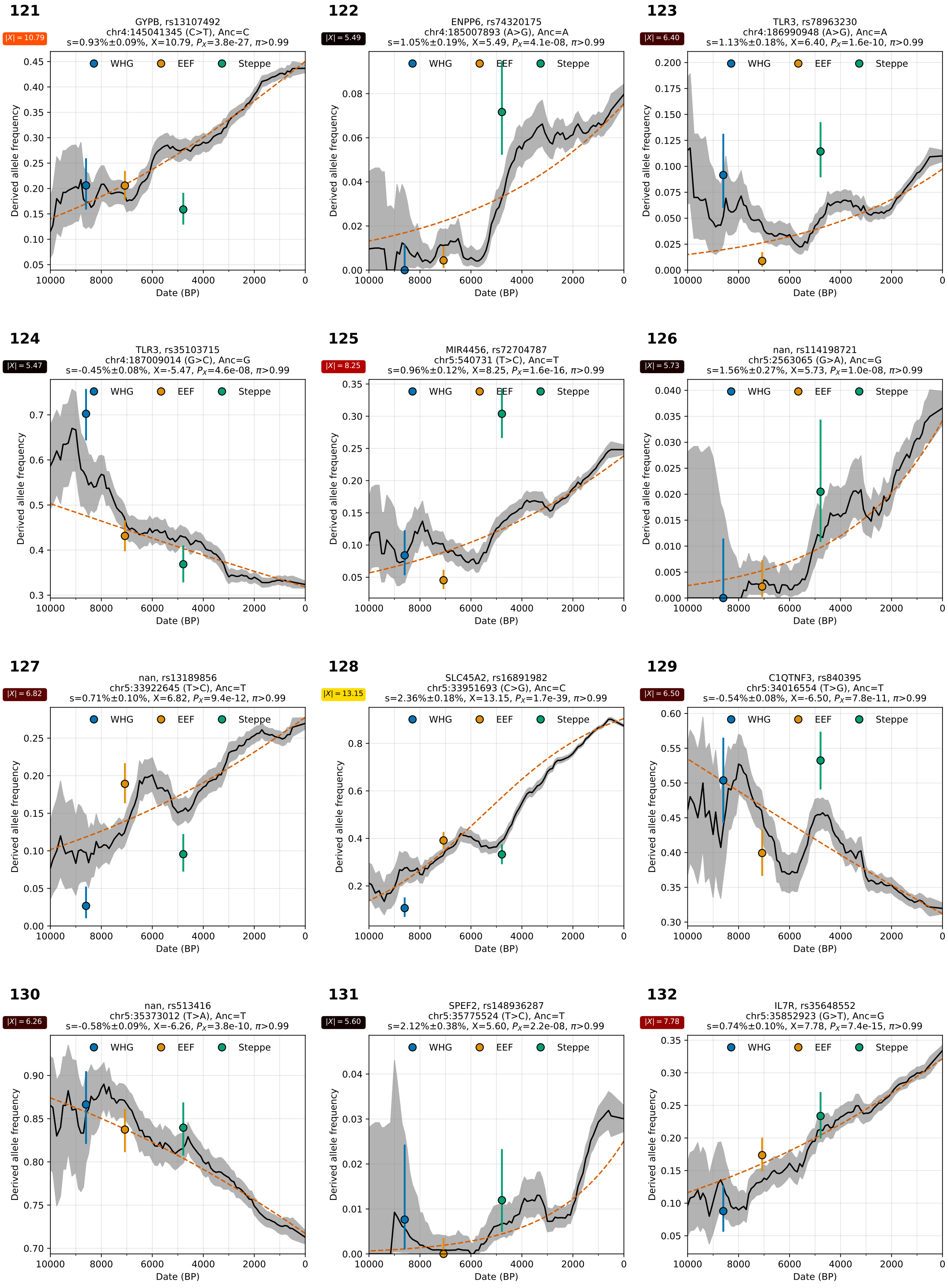

Supplementary Figure S5.11: Allele frequency over time.

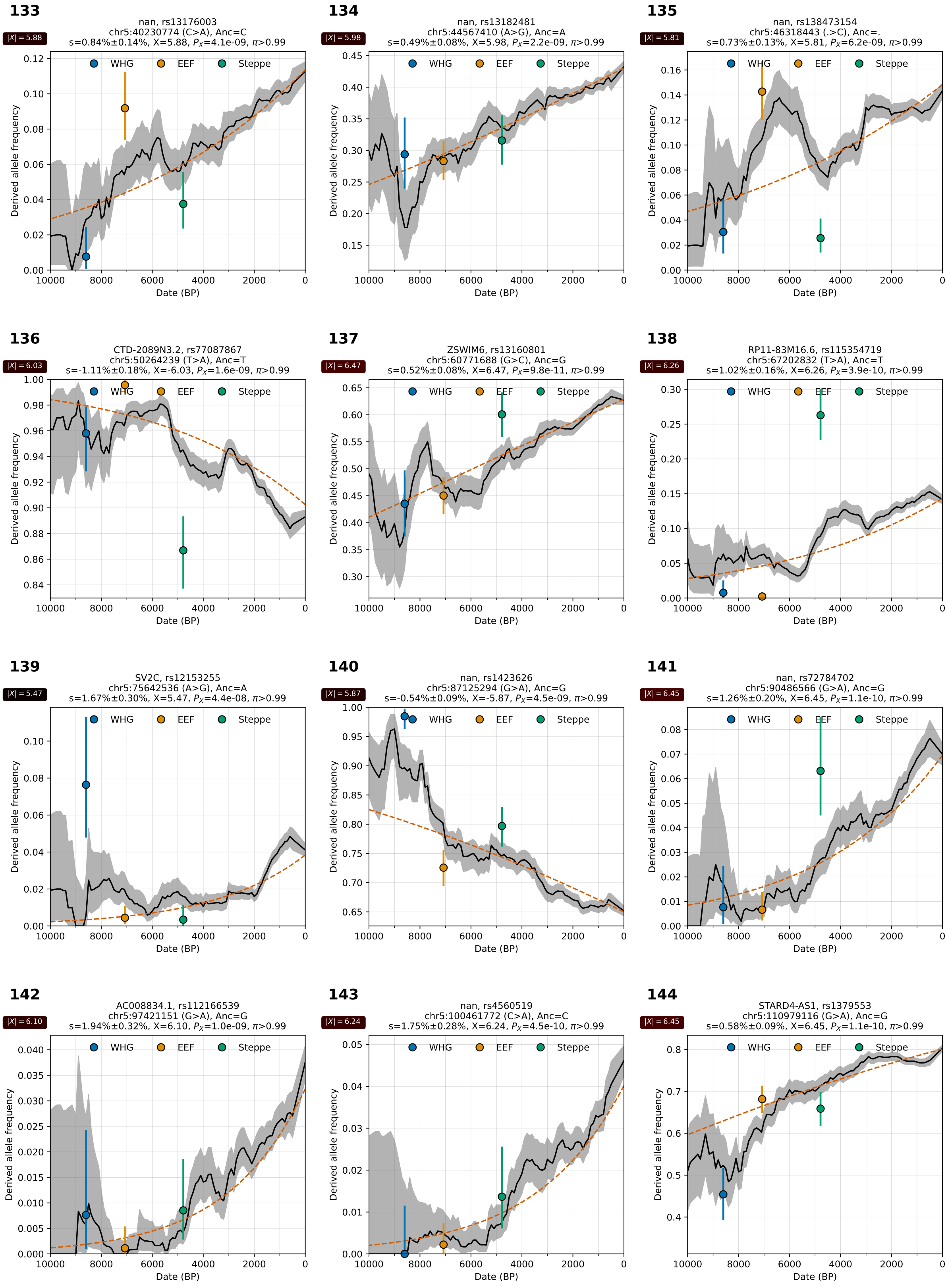

Supplementary Figure S5.12: Allele frequency over time.

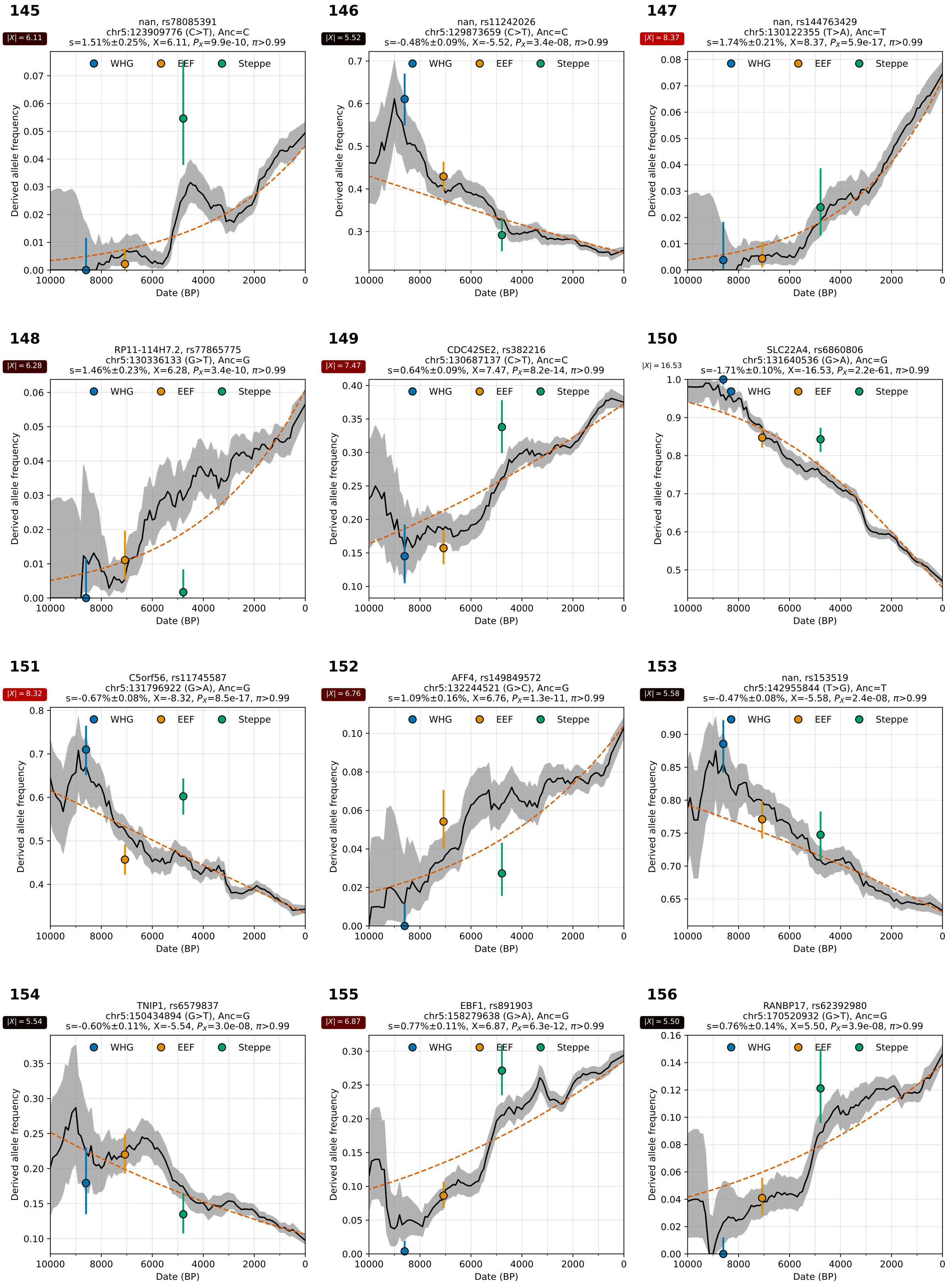

Supplementary Figure S5.13: Allele frequency over time.

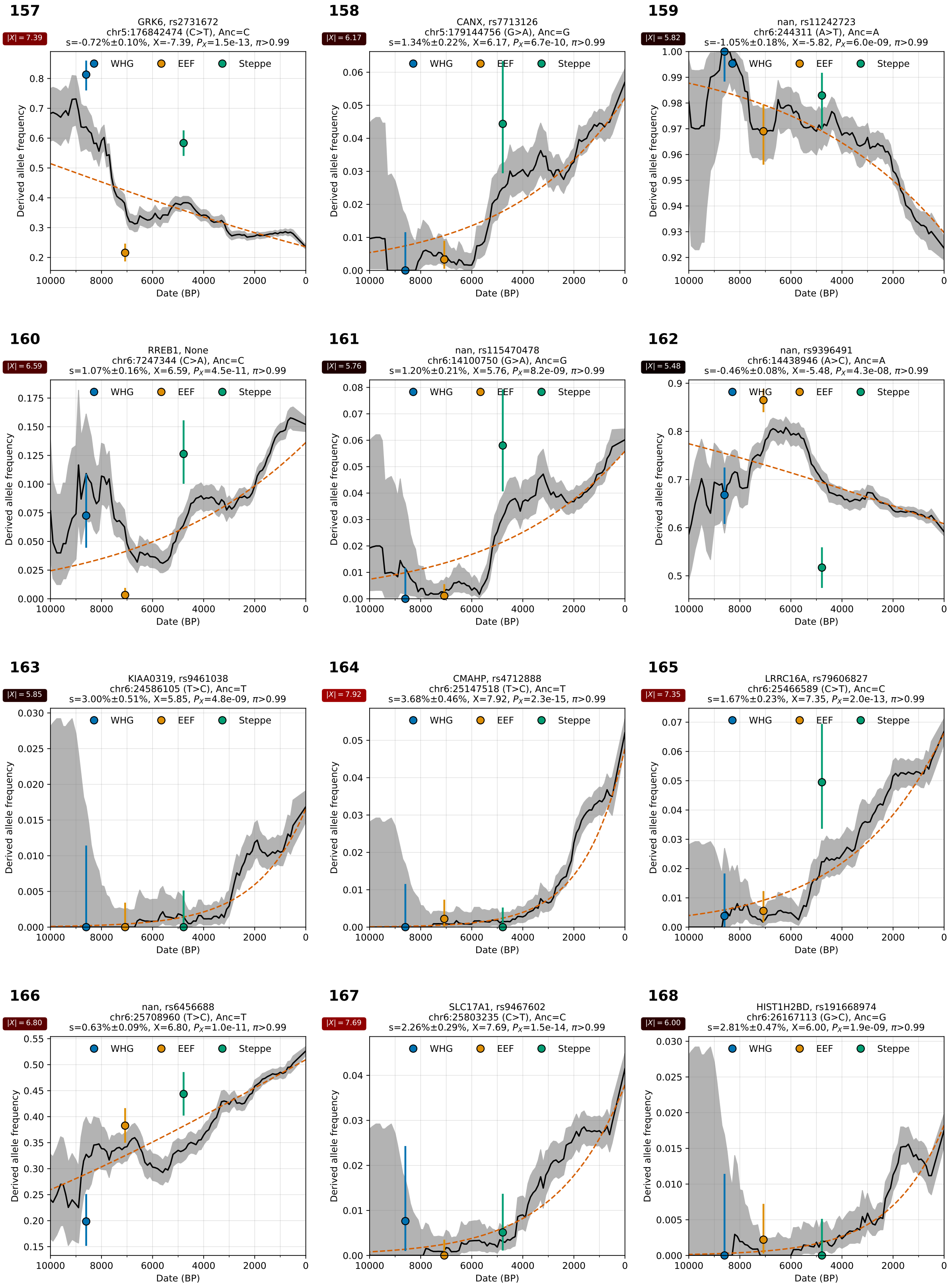

Supplementary Figure S5.14: Allele frequency over time.

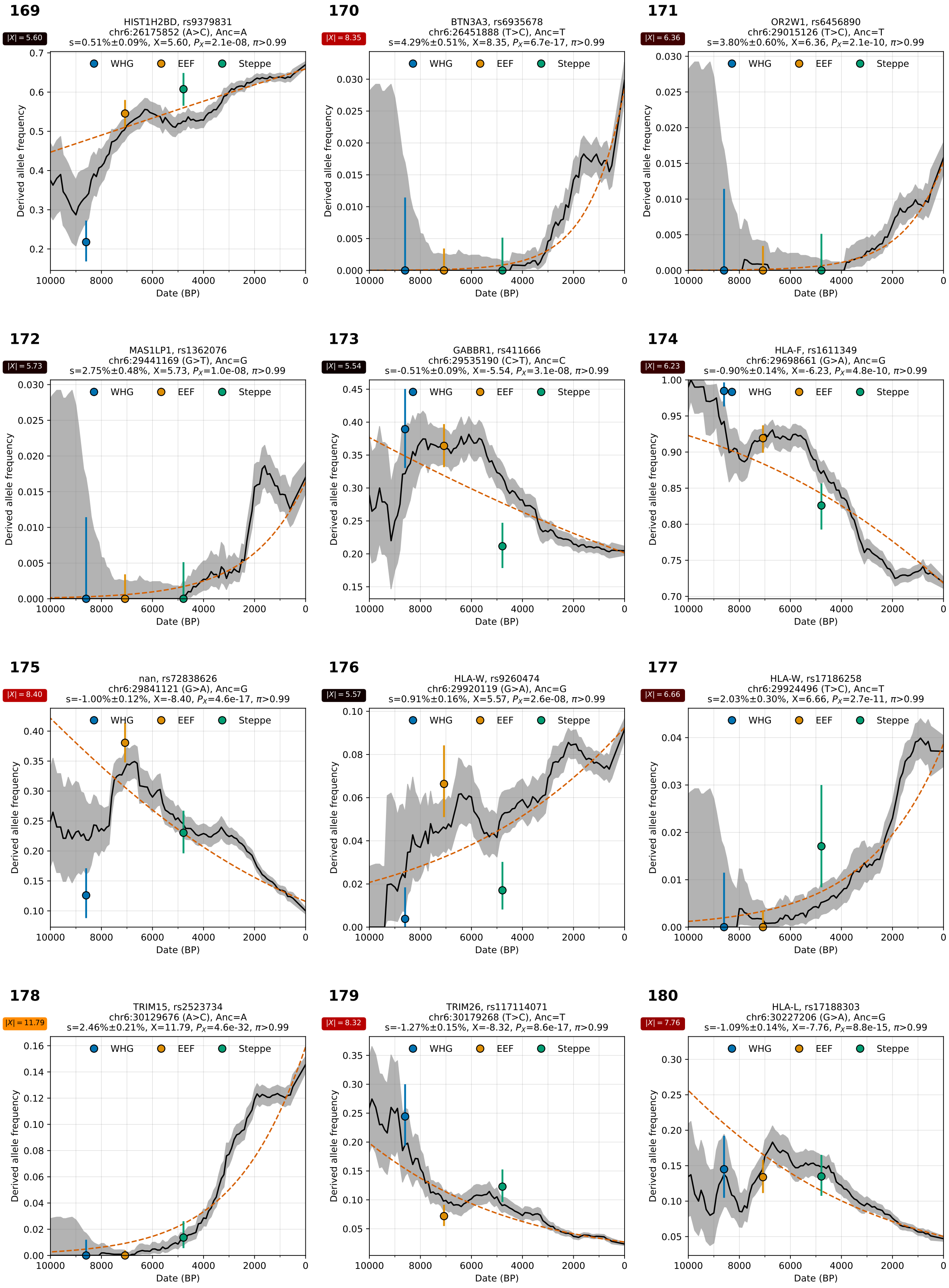

Supplementary Figure S5.15: Allele frequency over time.

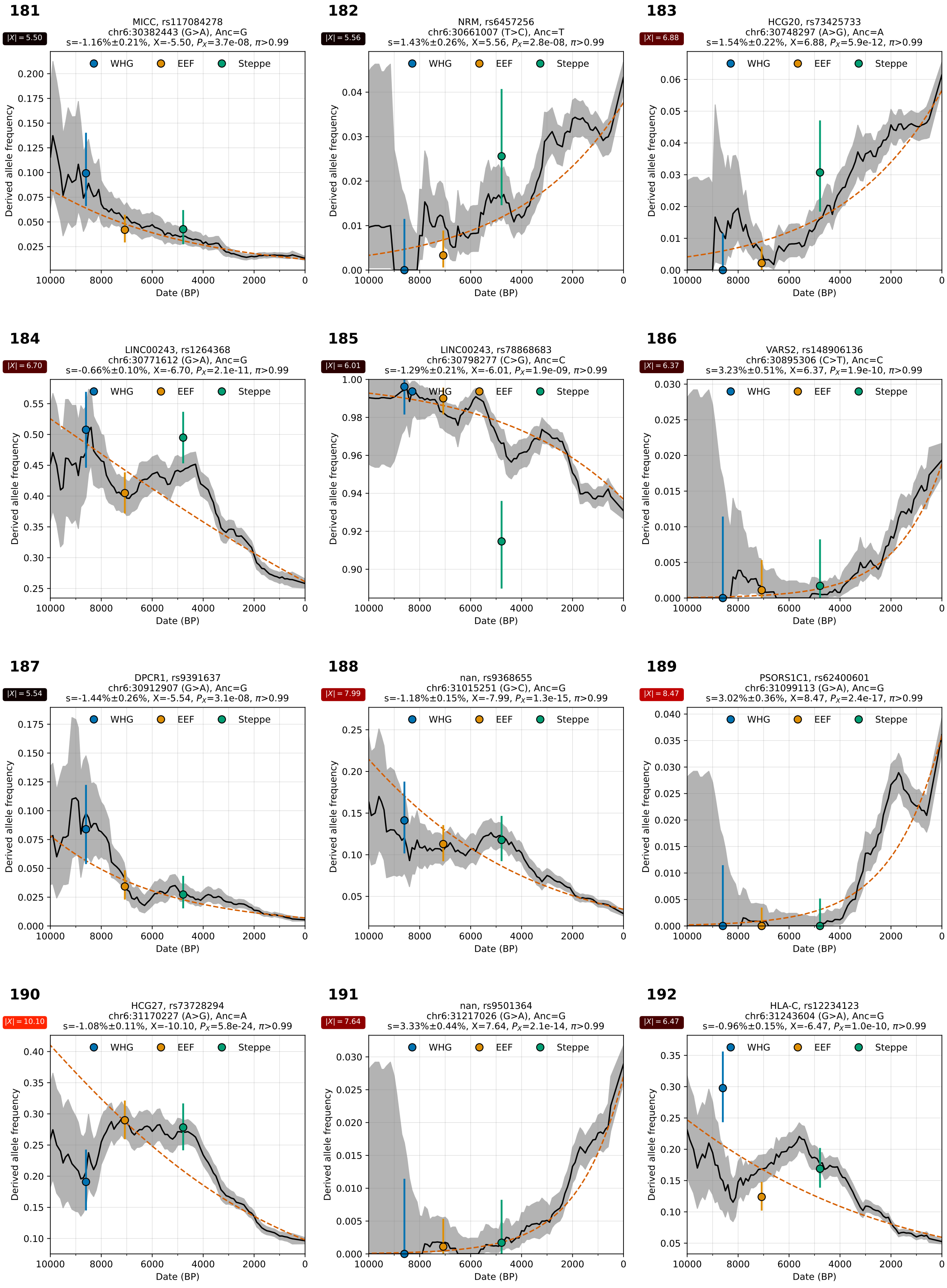

Supplementary Figure S5.16: Allele frequency over time.

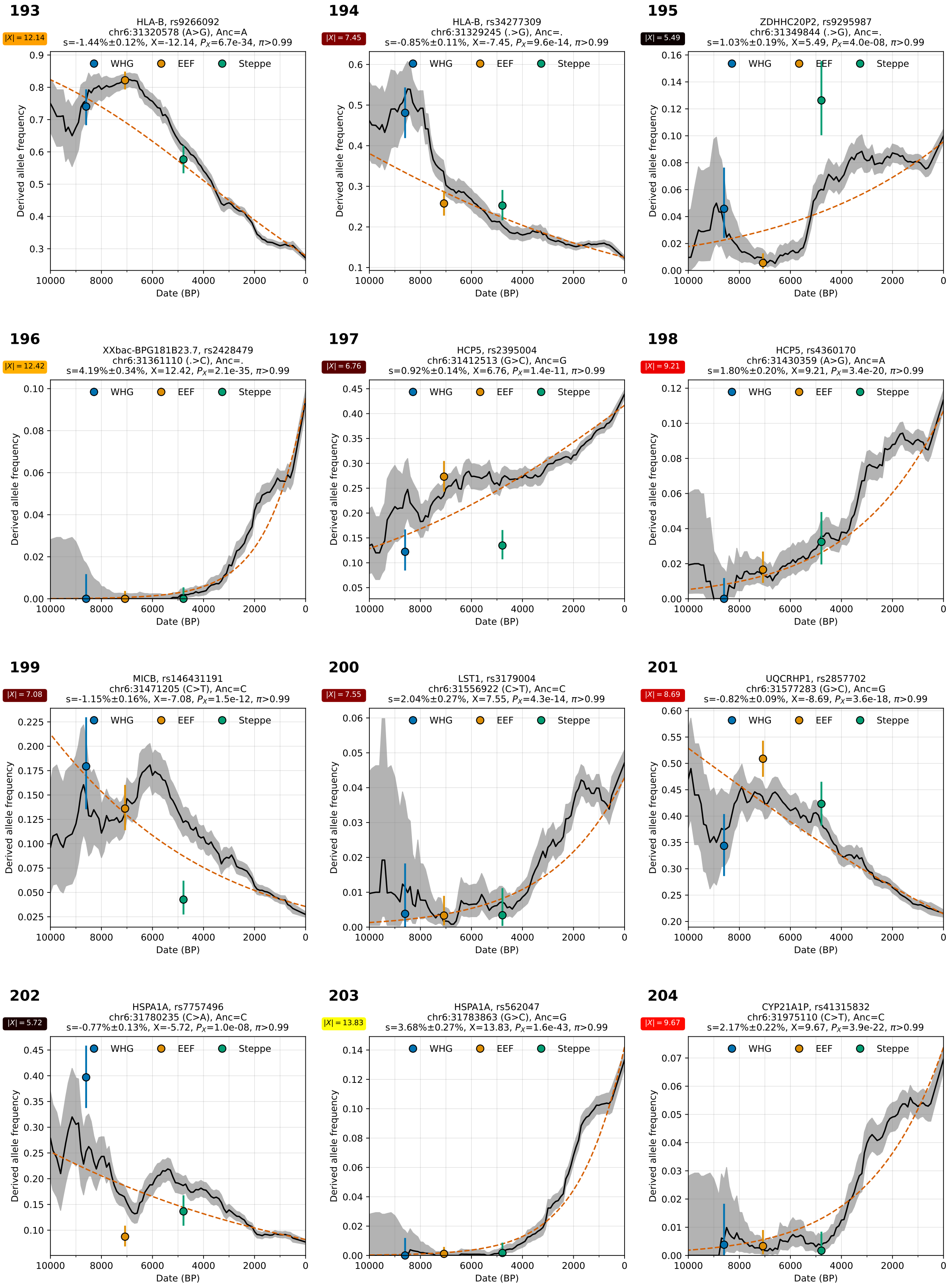

Supplementary Figure S5.17: Allele frequency over time.

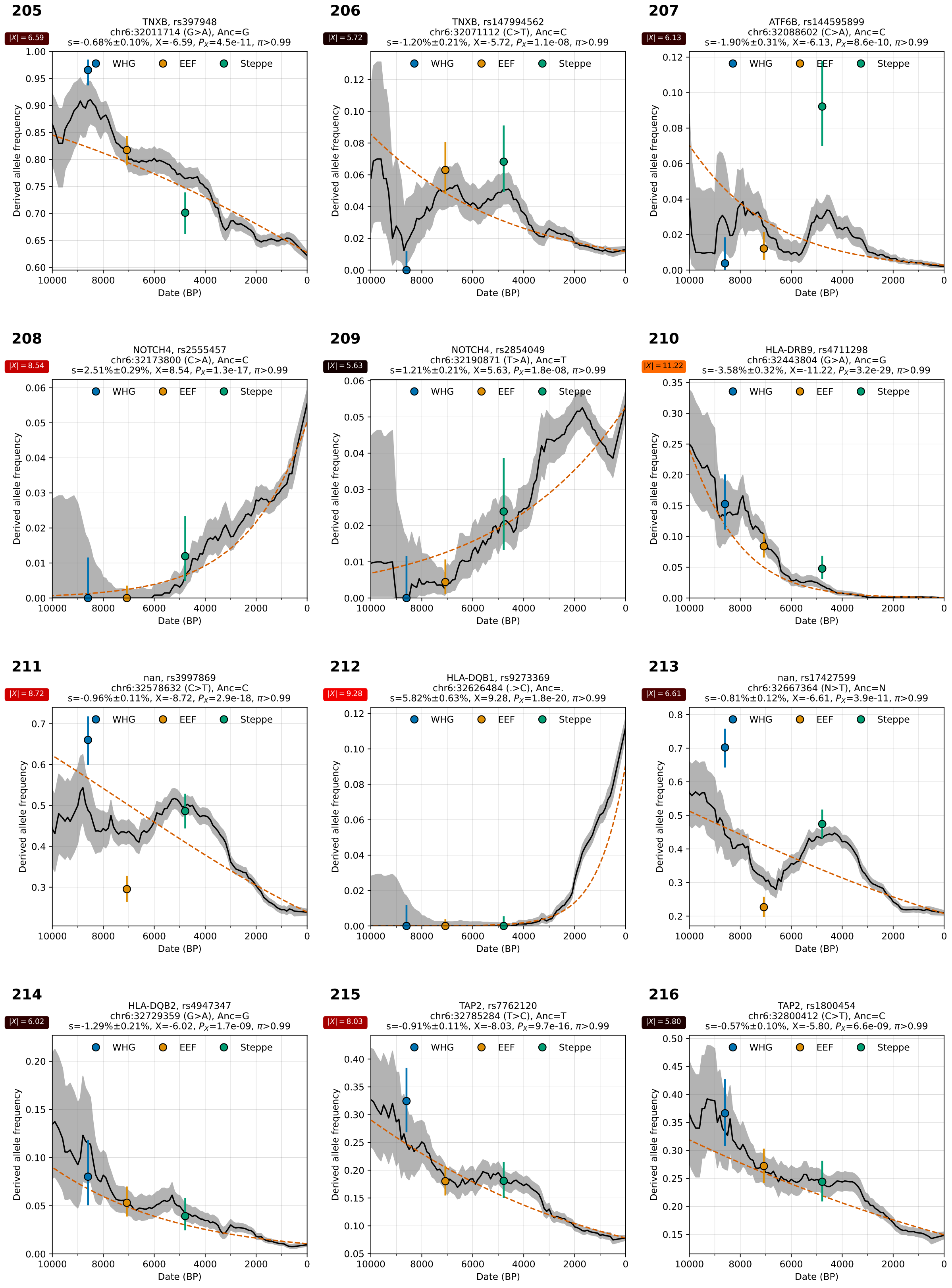

Supplementary Figure S5.18: Allele frequency over time.

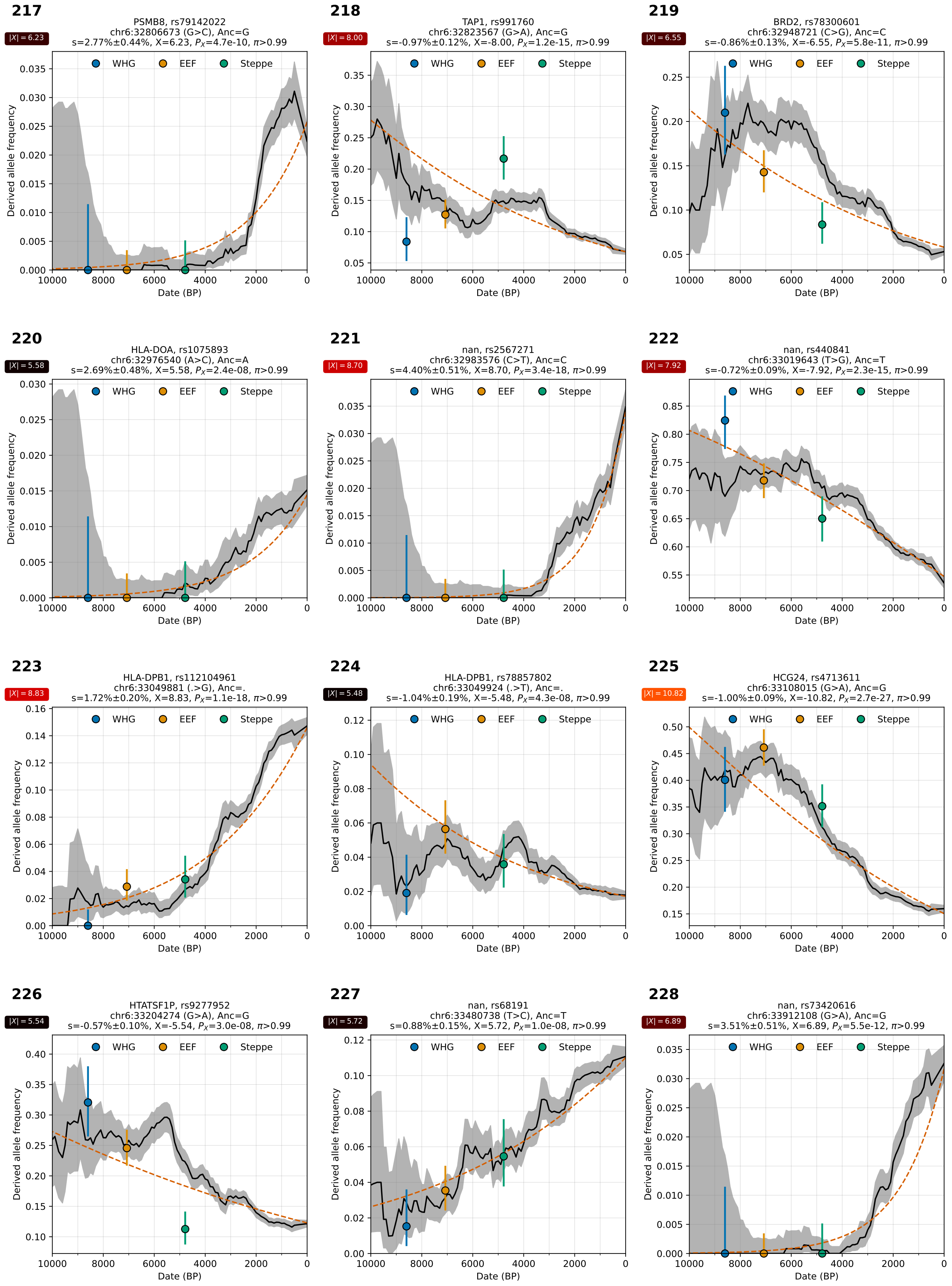

Supplementary Figure S5.19: Allele frequency over time.

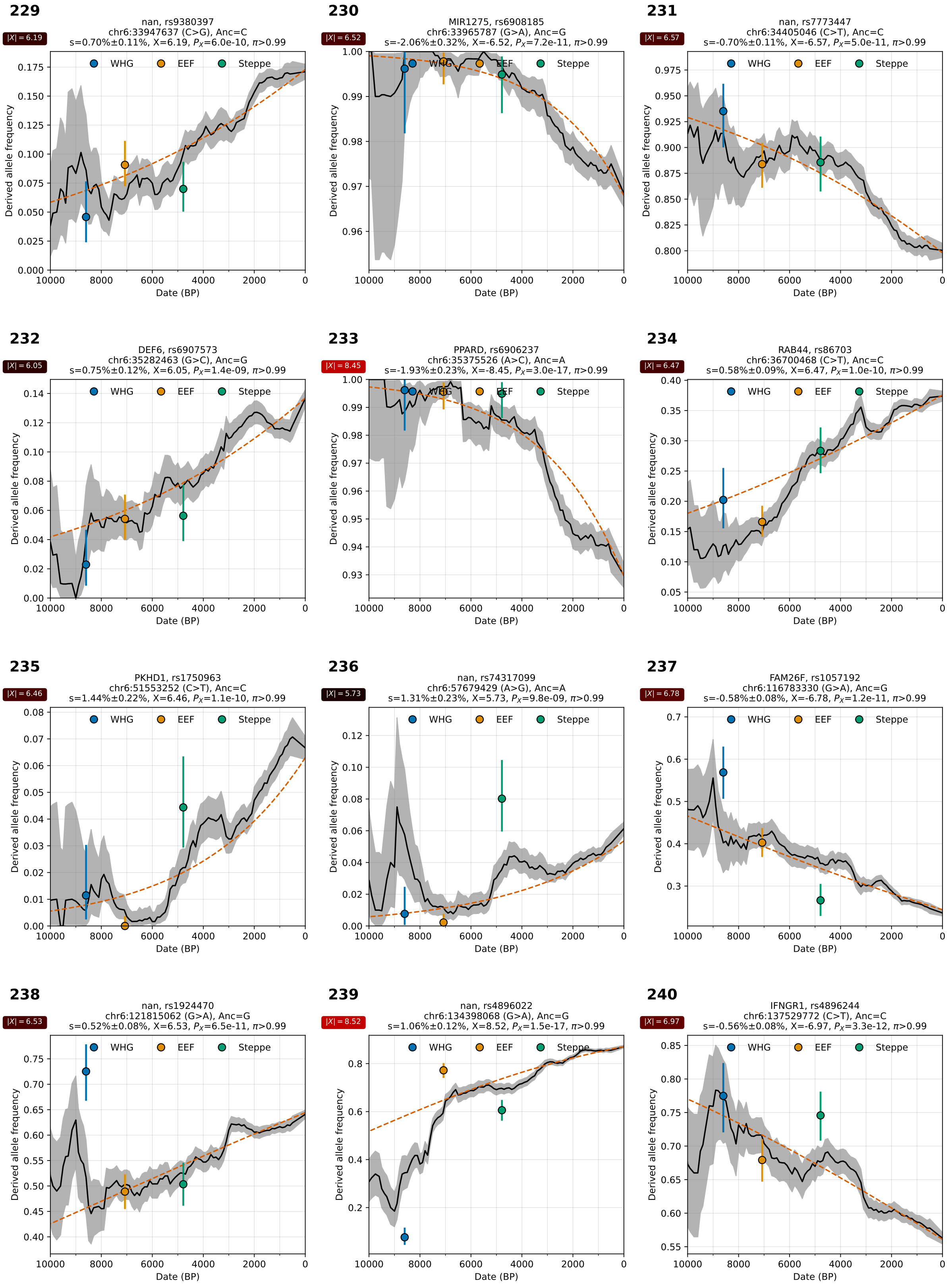

Supplementary Figure S5.20: Allele frequency over time.

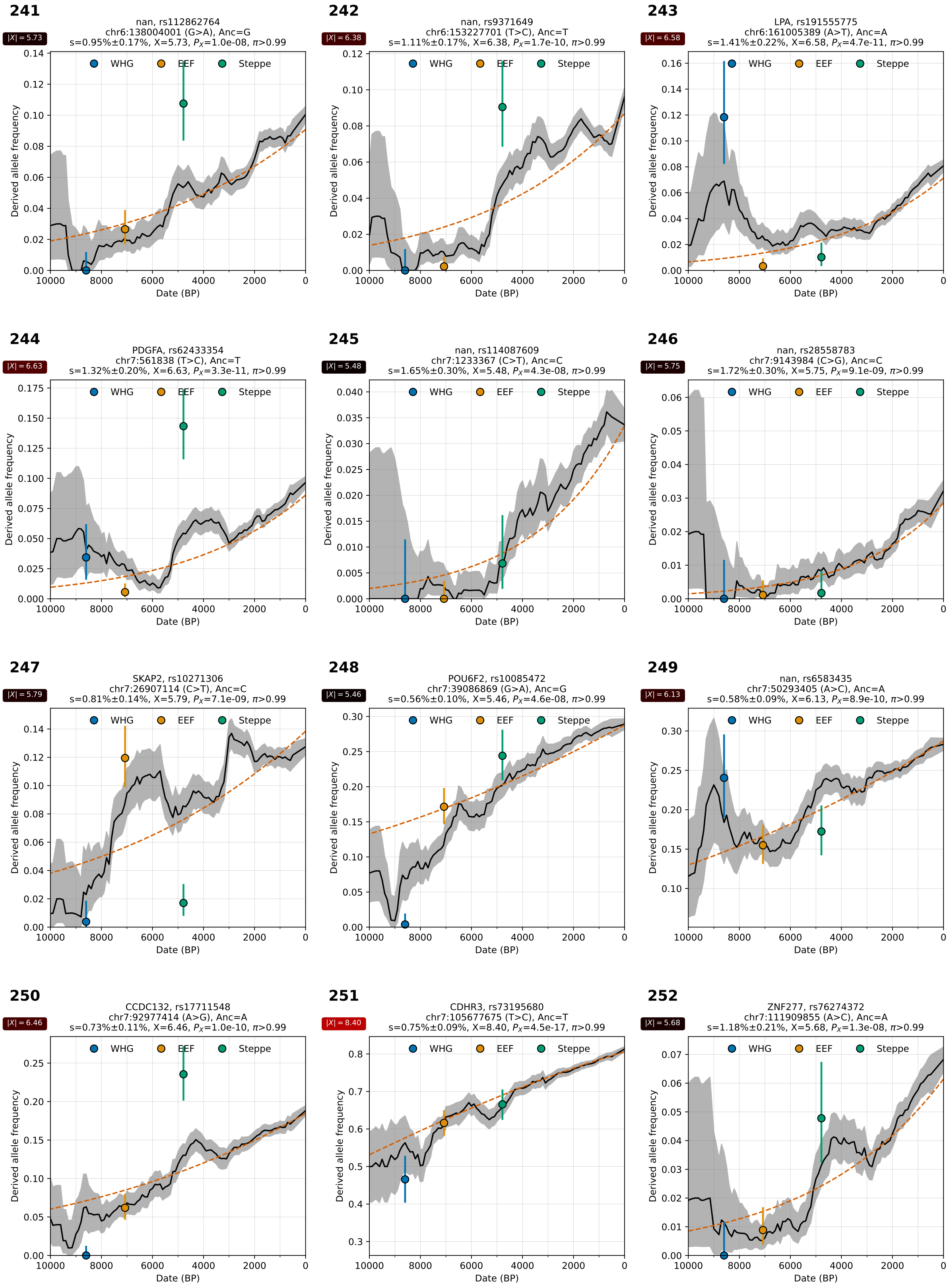

Supplementary Figure S5.21: Allele frequency over time.

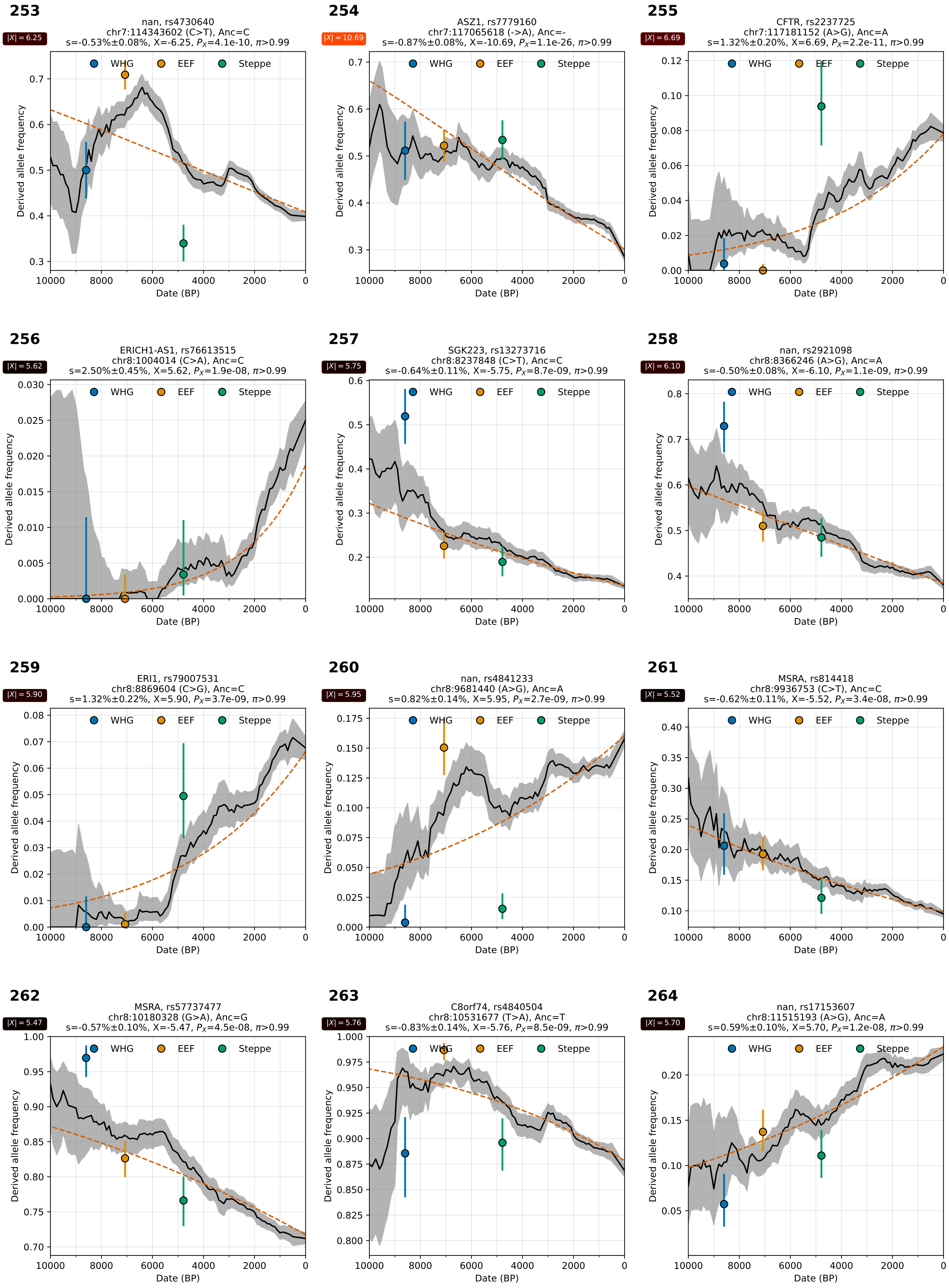

Supplementary Figure S5.22: Allele frequency over time.

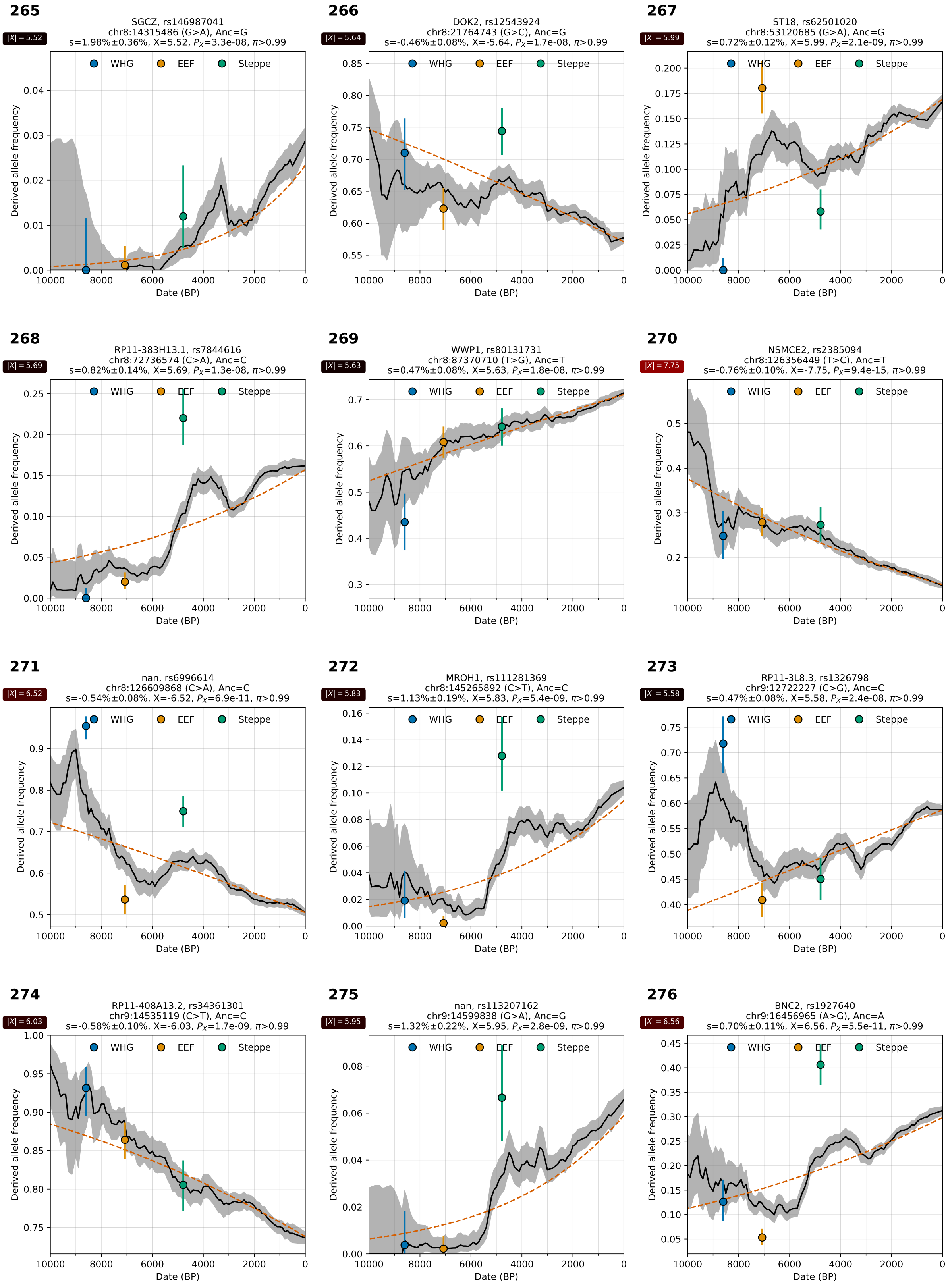

Supplementary Figure S5.23: Allele frequency over time.

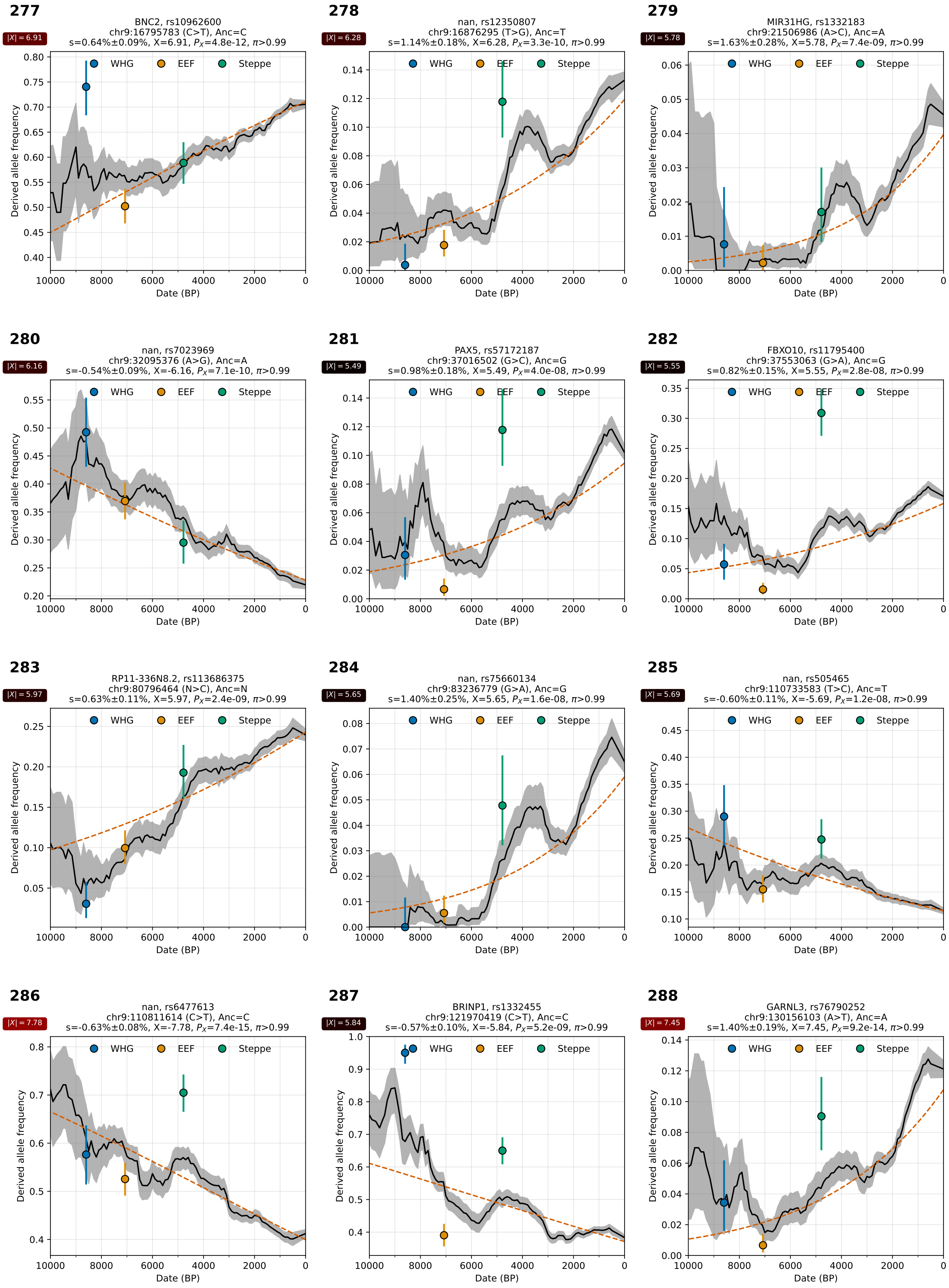

Supplementary Figure S5.24: Allele frequency over time.

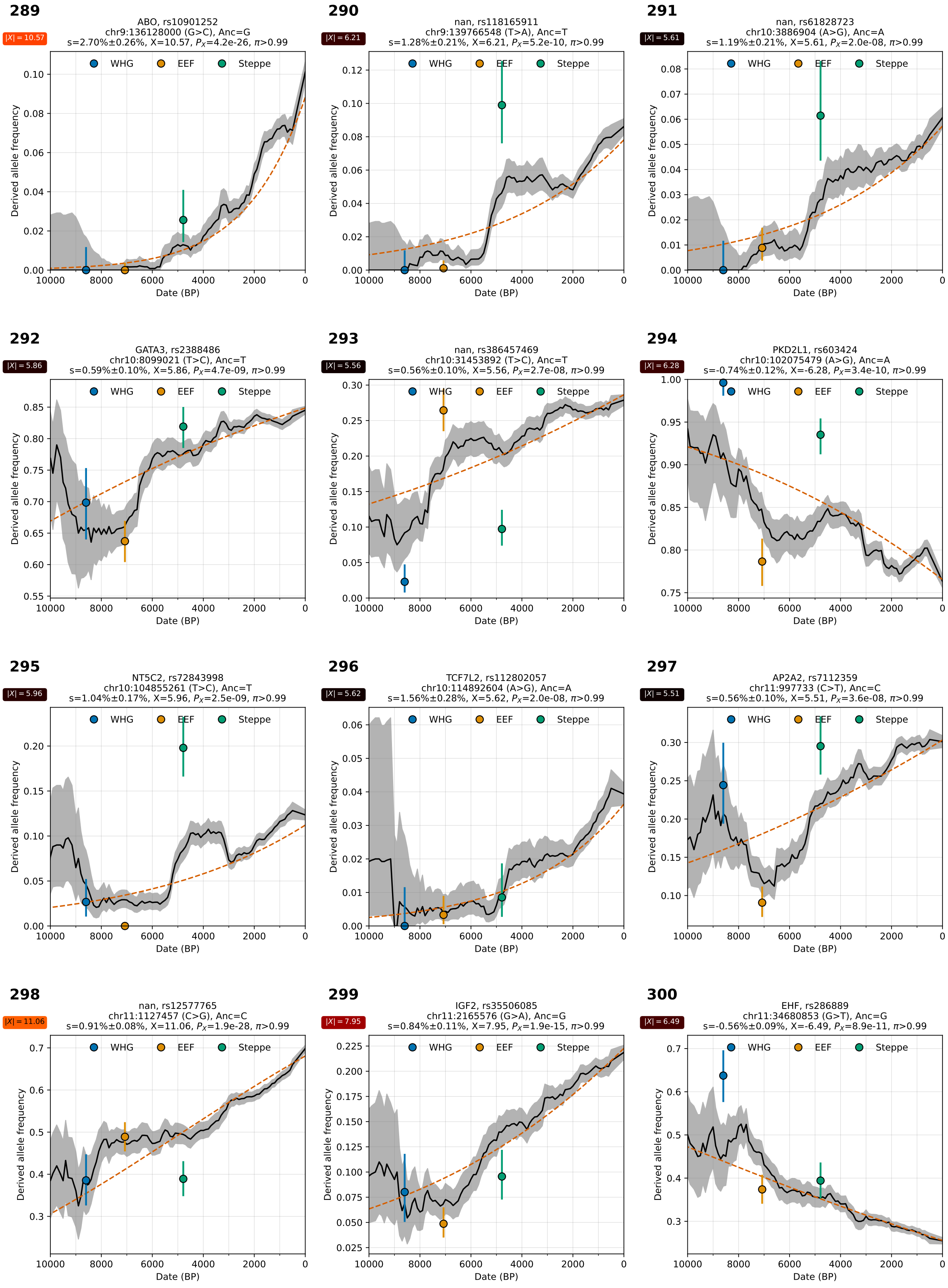

Supplementary Figure S5.25: Allele frequency over time.

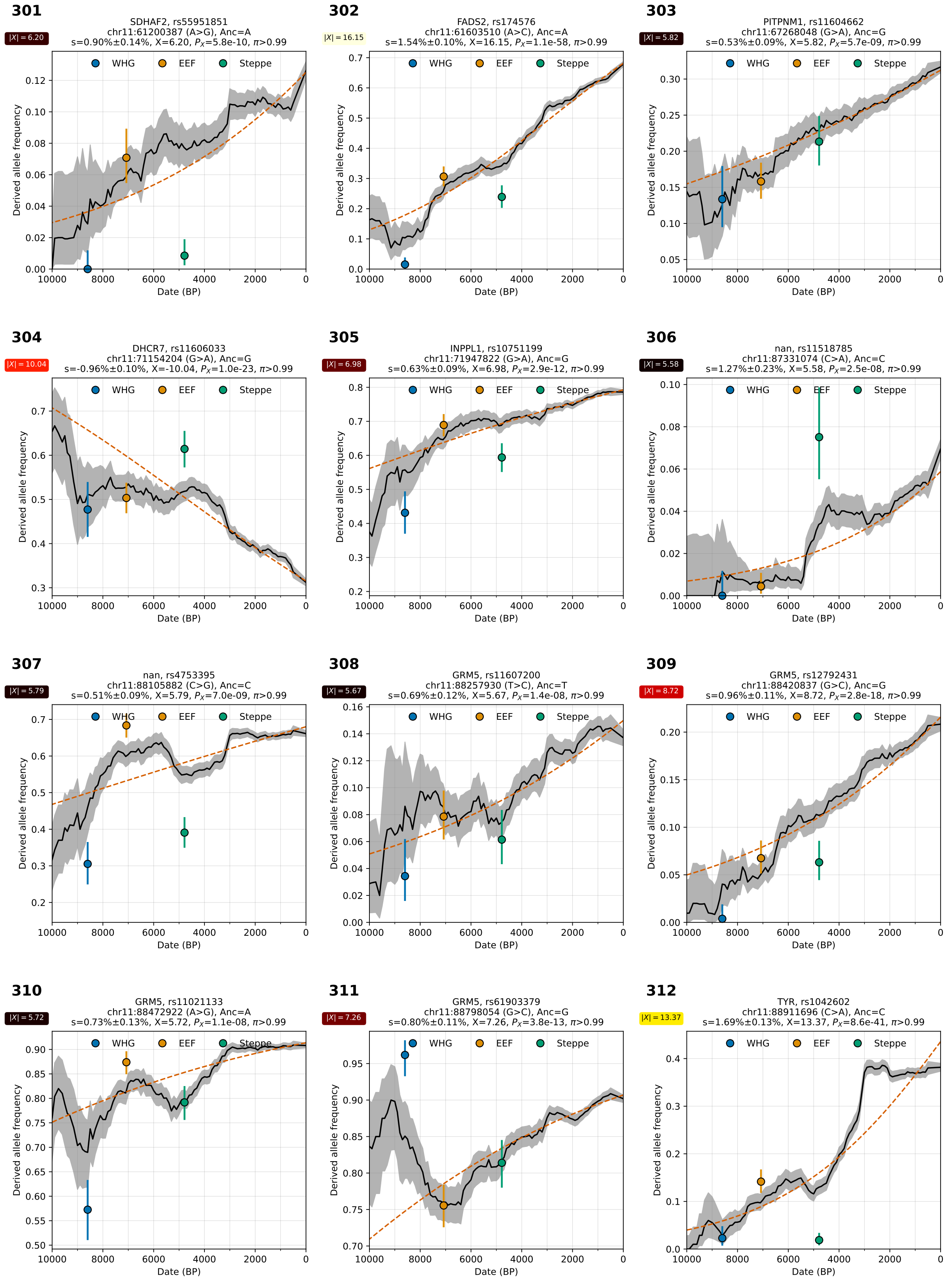

Supplementary Figure S5.26: Allele frequency over time.

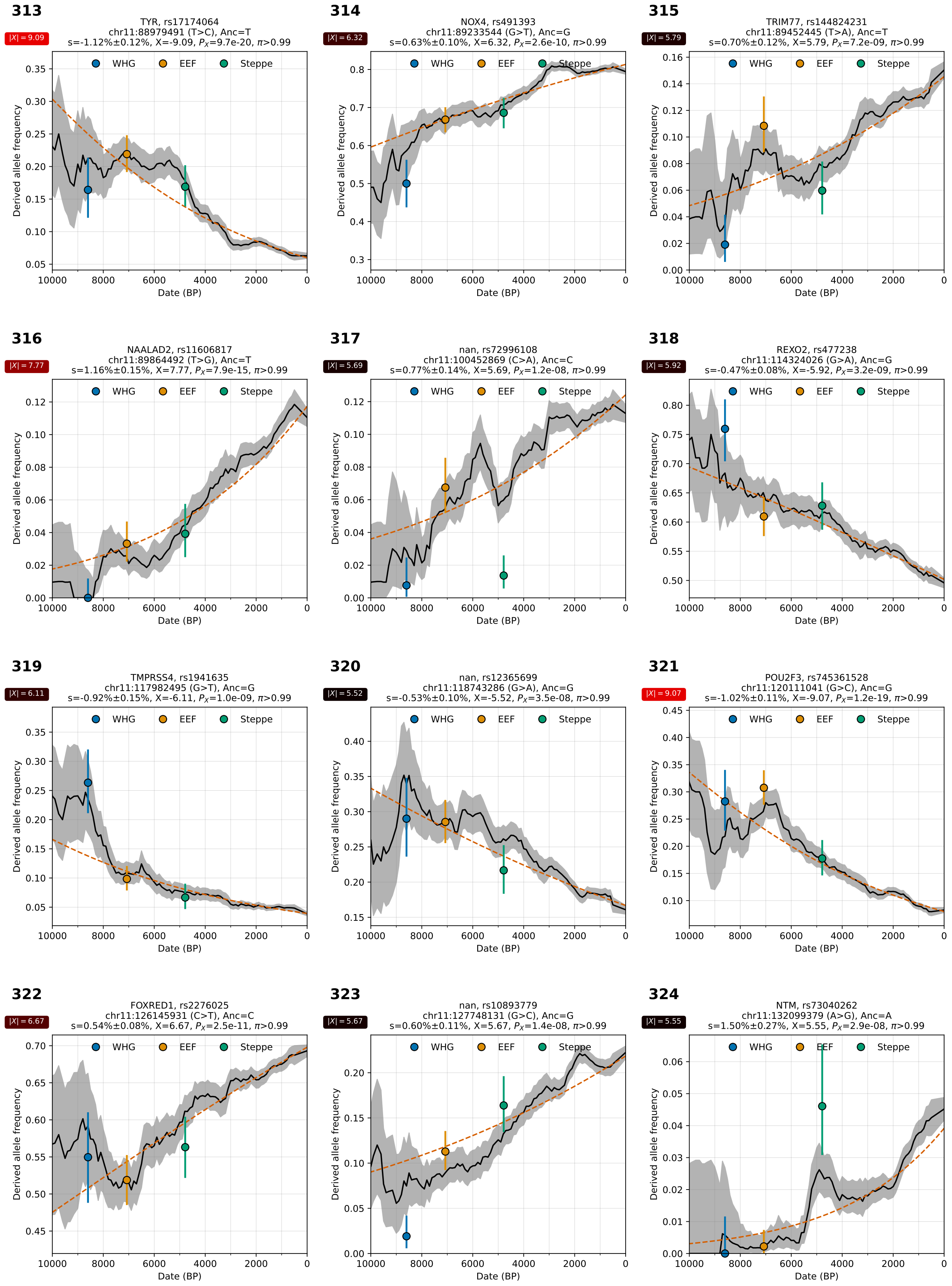

Supplementary Figure S5.27: Allele frequency over time.

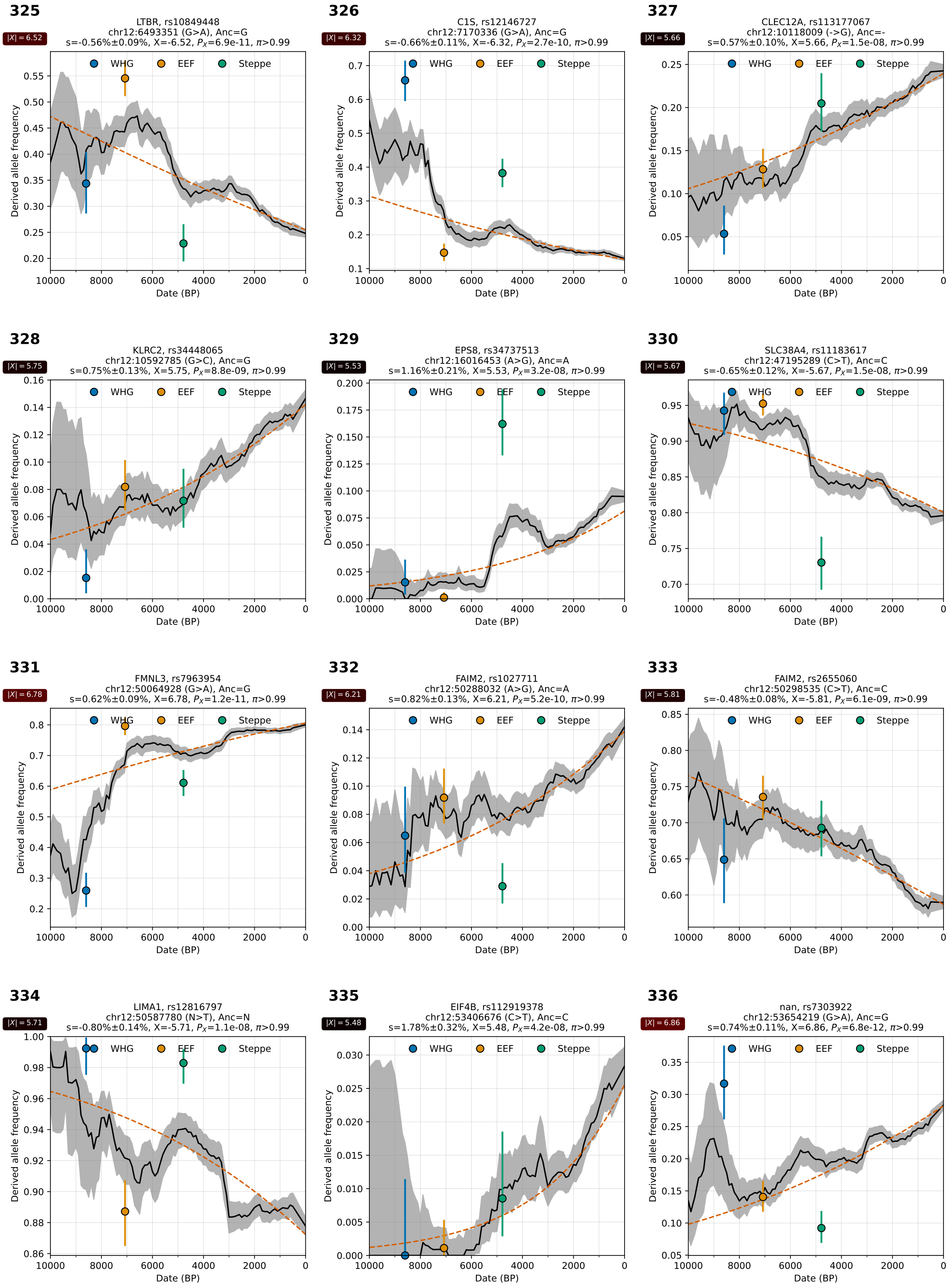

Supplementary Figure S5.28: Allele frequency over time.

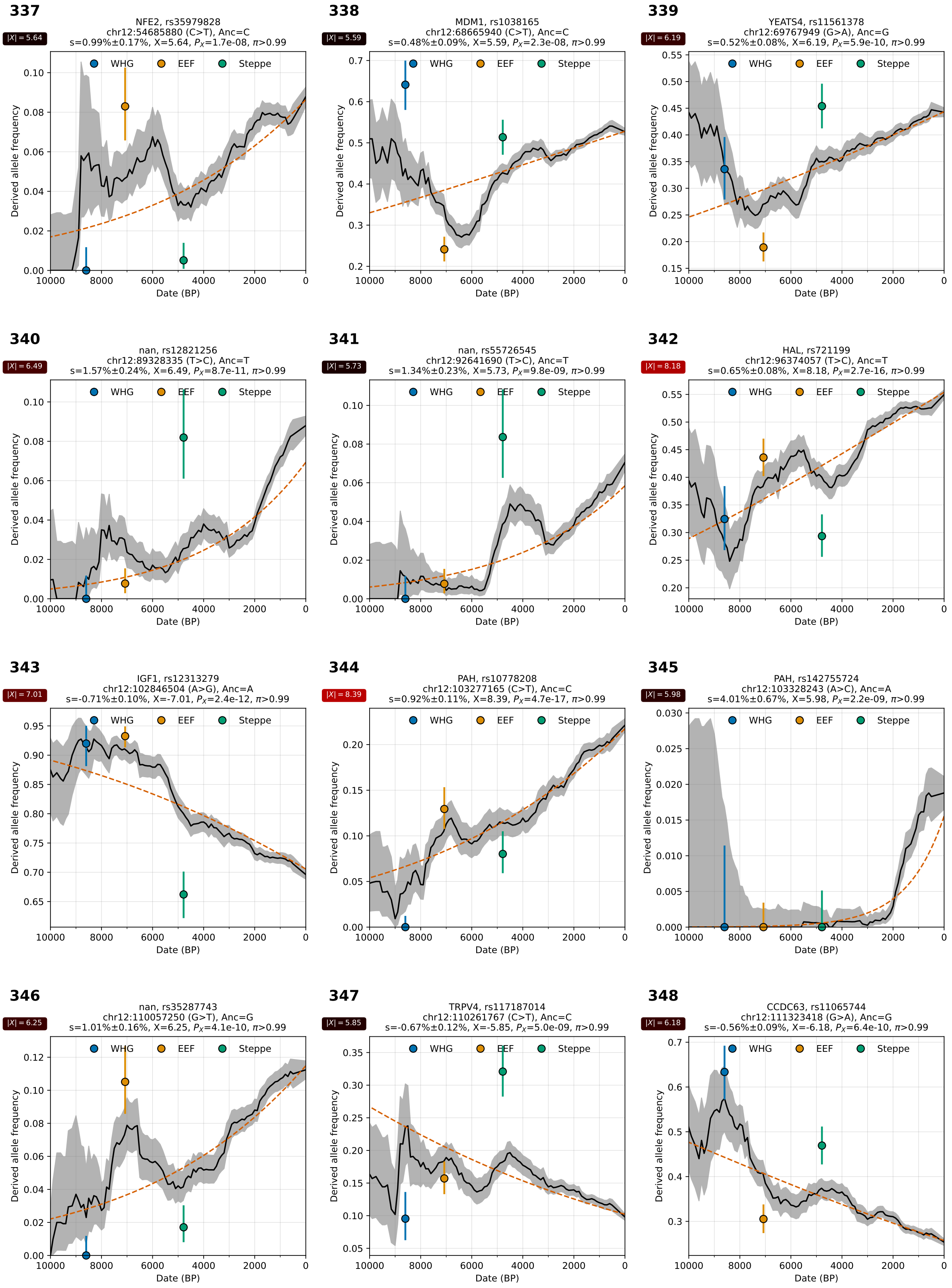

Supplementary Figure S5.29: Allele frequency over time.

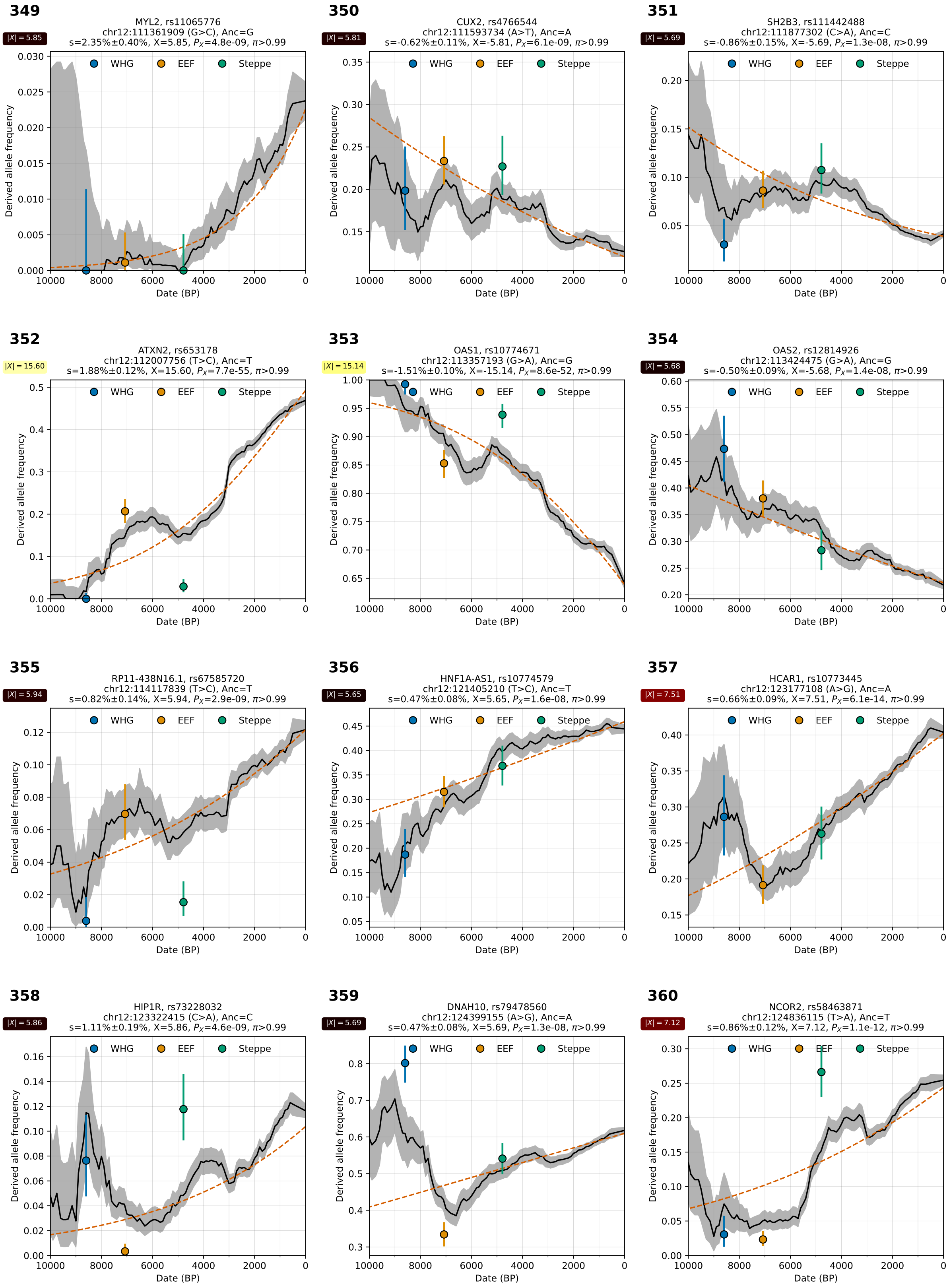

Supplementary Figure S5.30: Allele frequency over time.

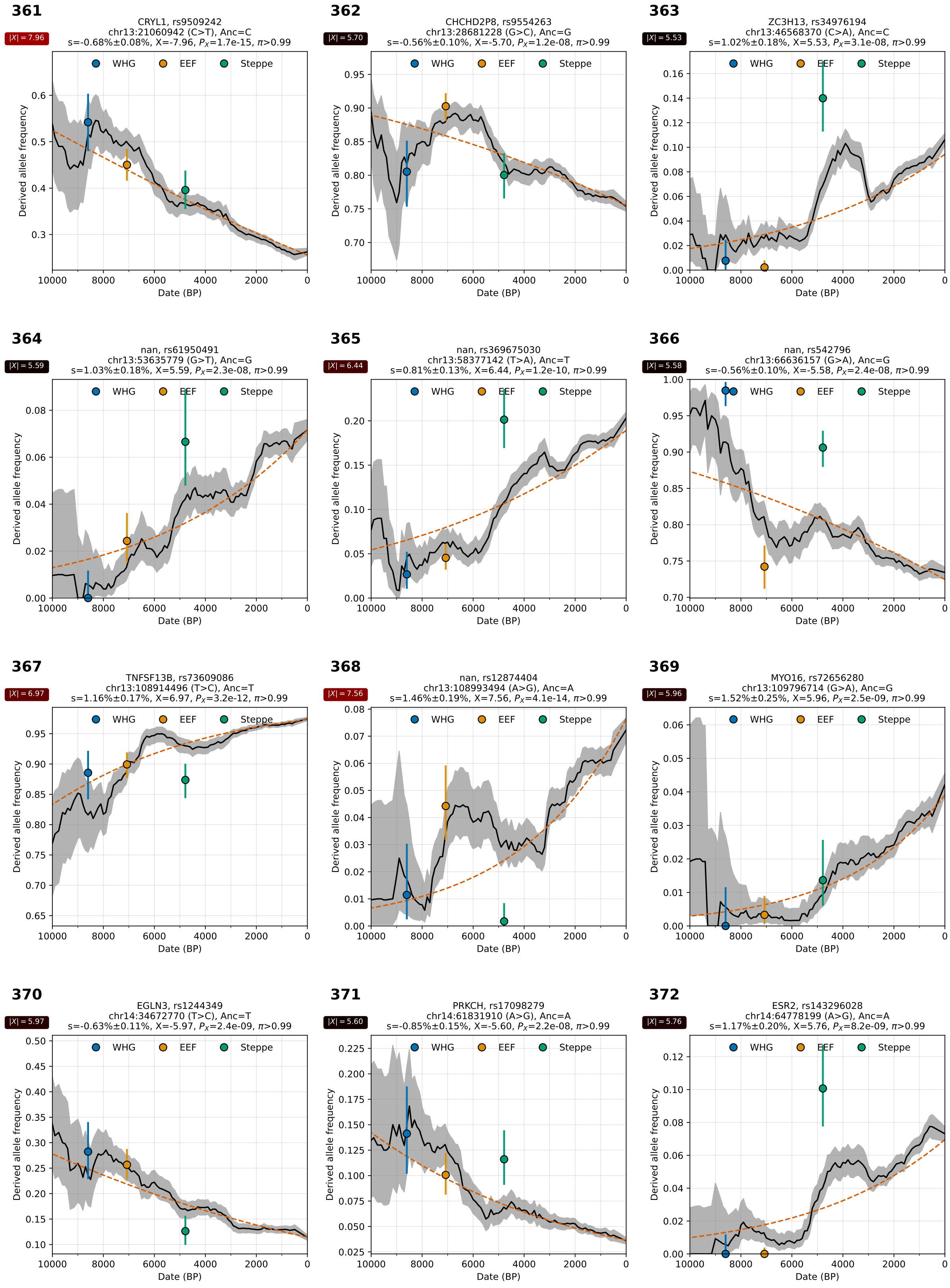

Supplementary Figure S5.31: Allele frequency over time.

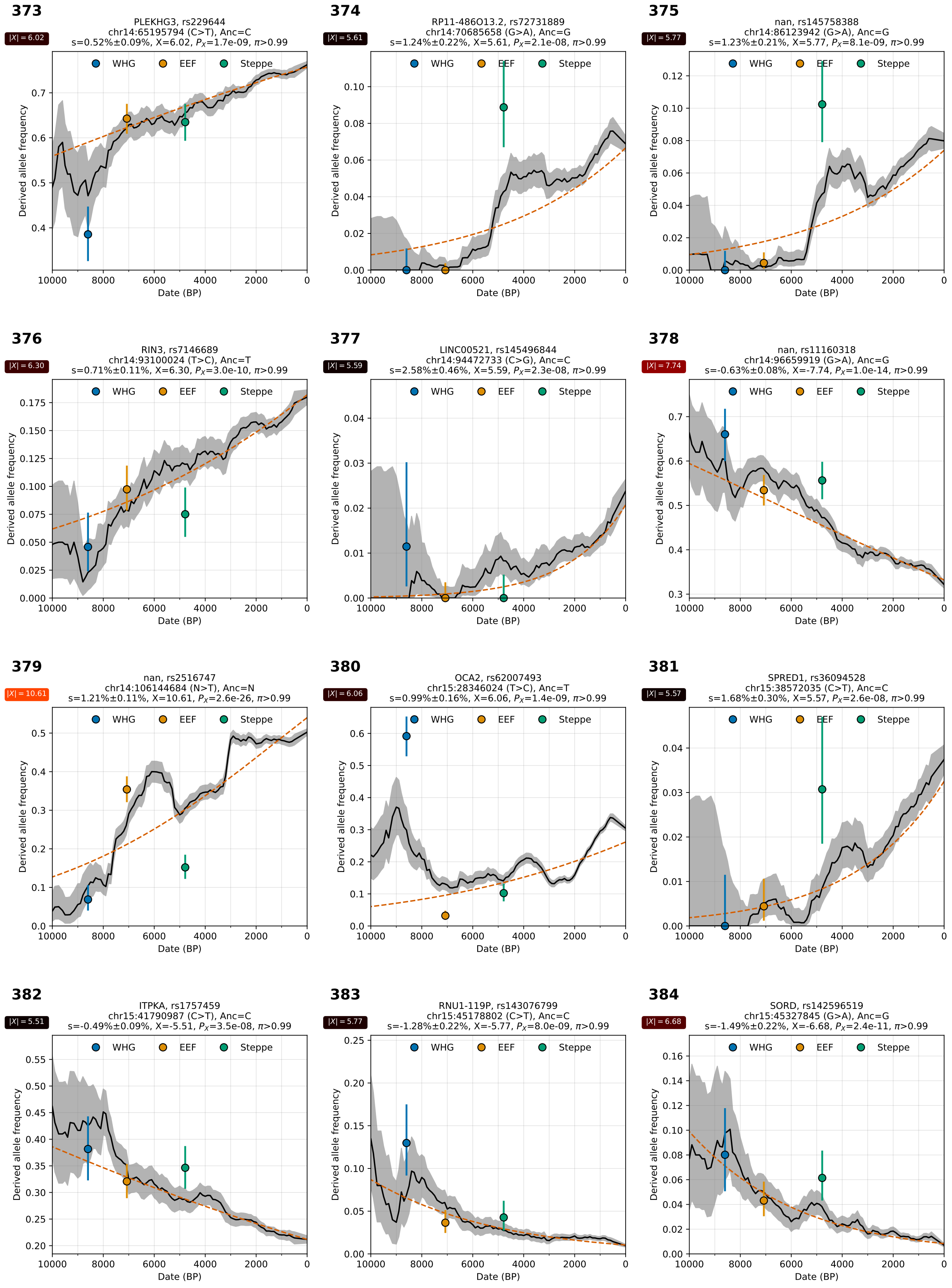

Supplementary Figure S5.32: Allele frequency over time.

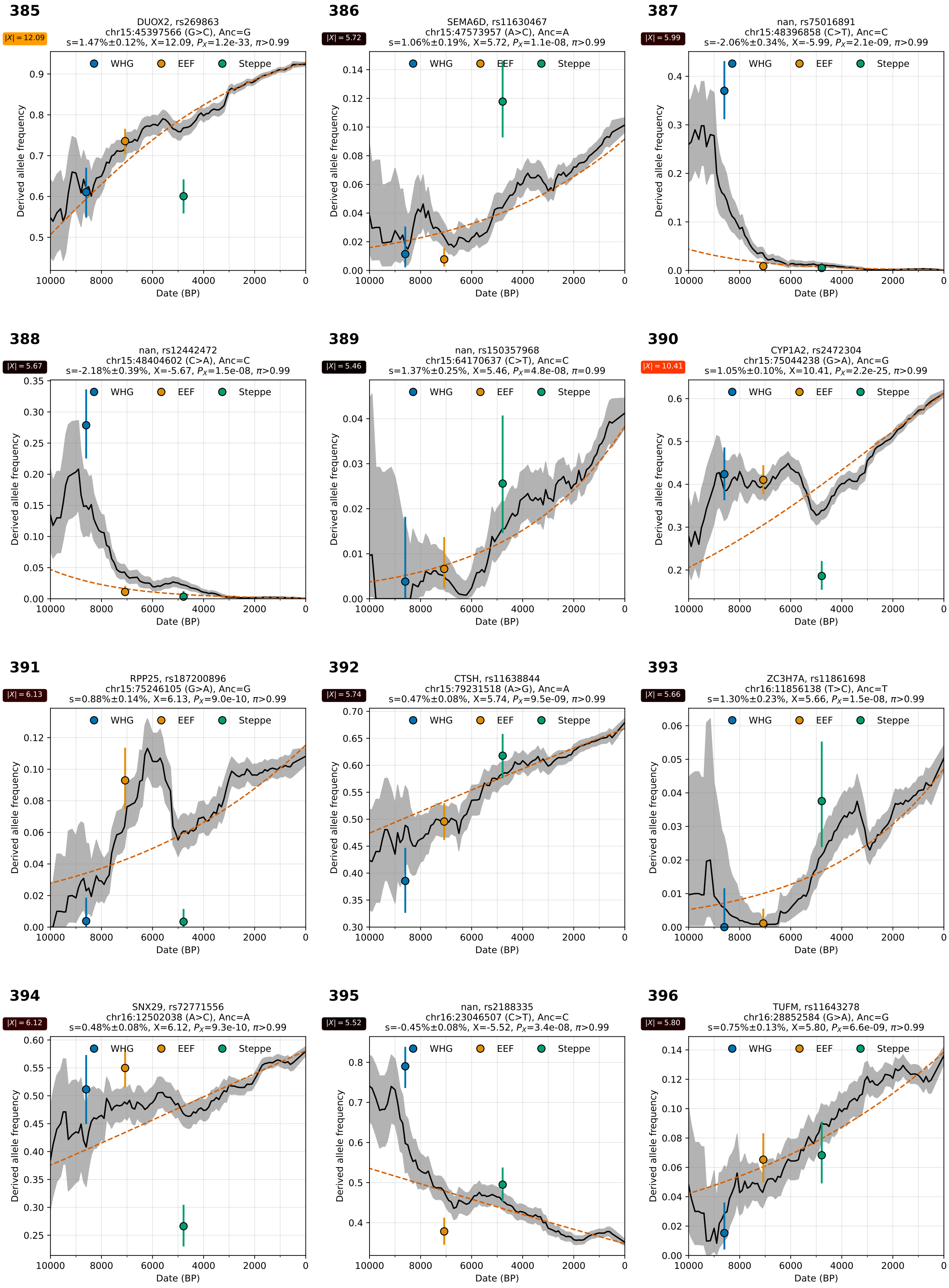

Supplementary Figure S5.33: Allele frequency over time.

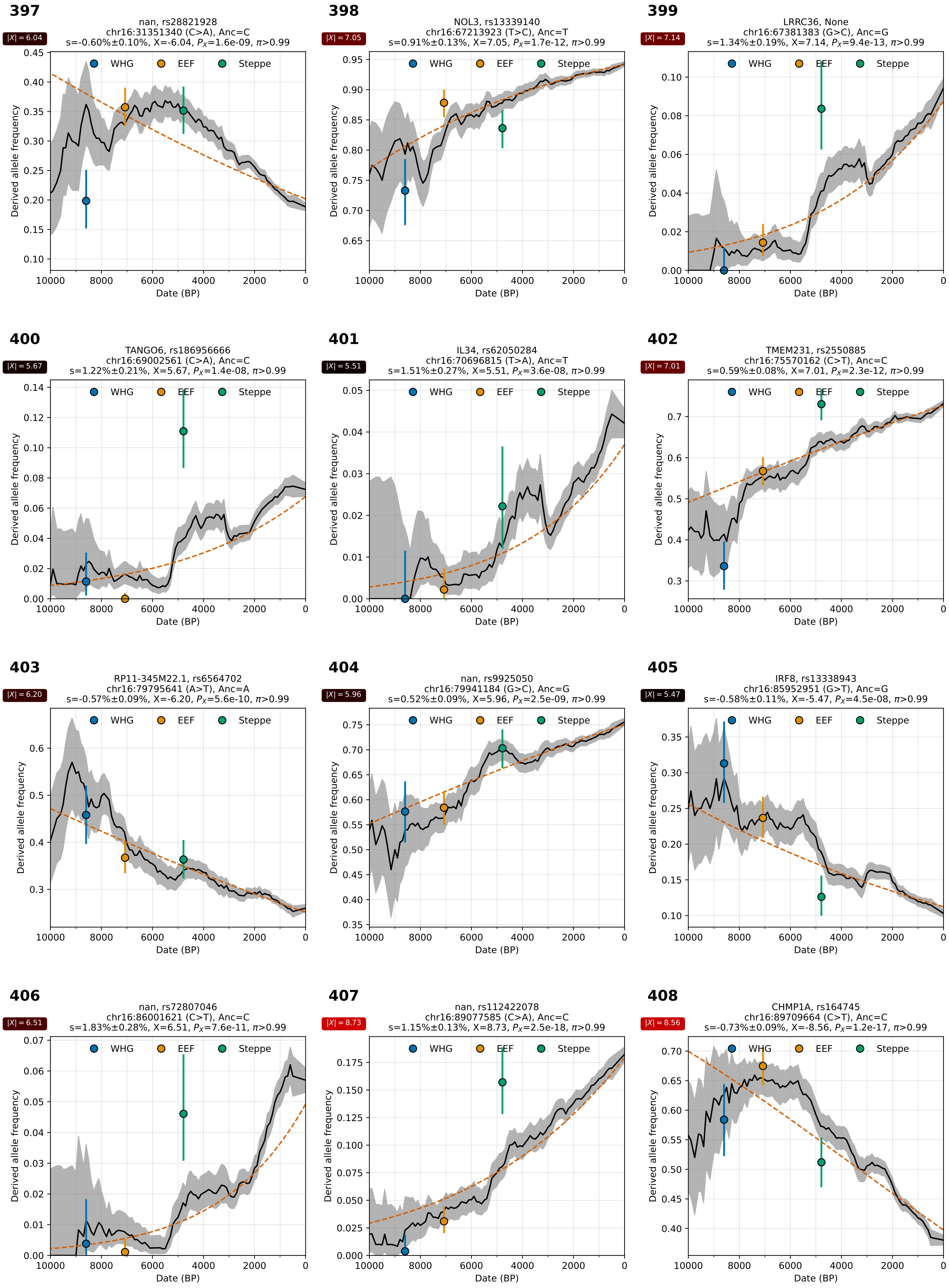

Supplementary Figure S5.34: Allele frequency over time.

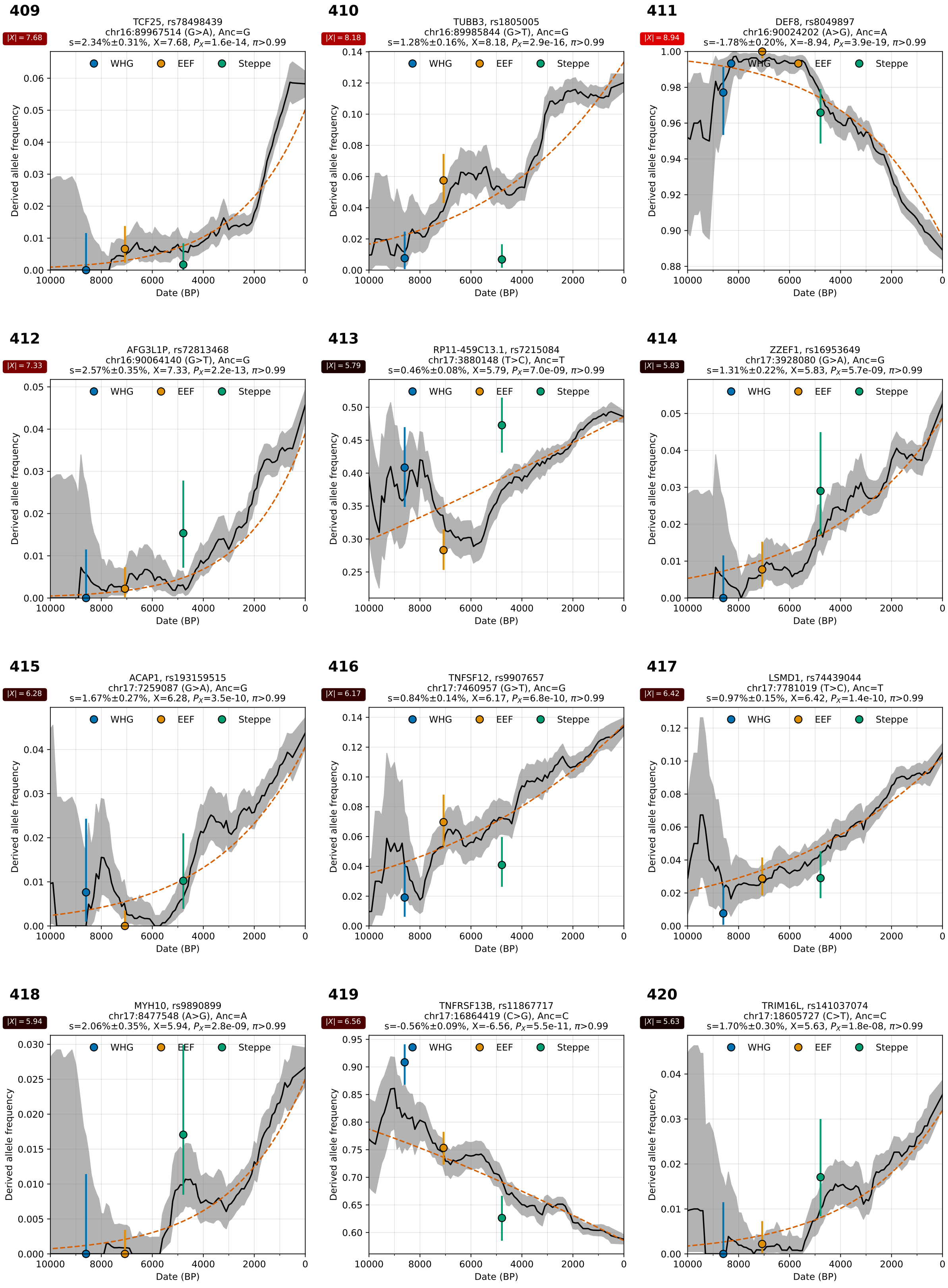

Supplementary Figure S5.35: Allele frequency over time.

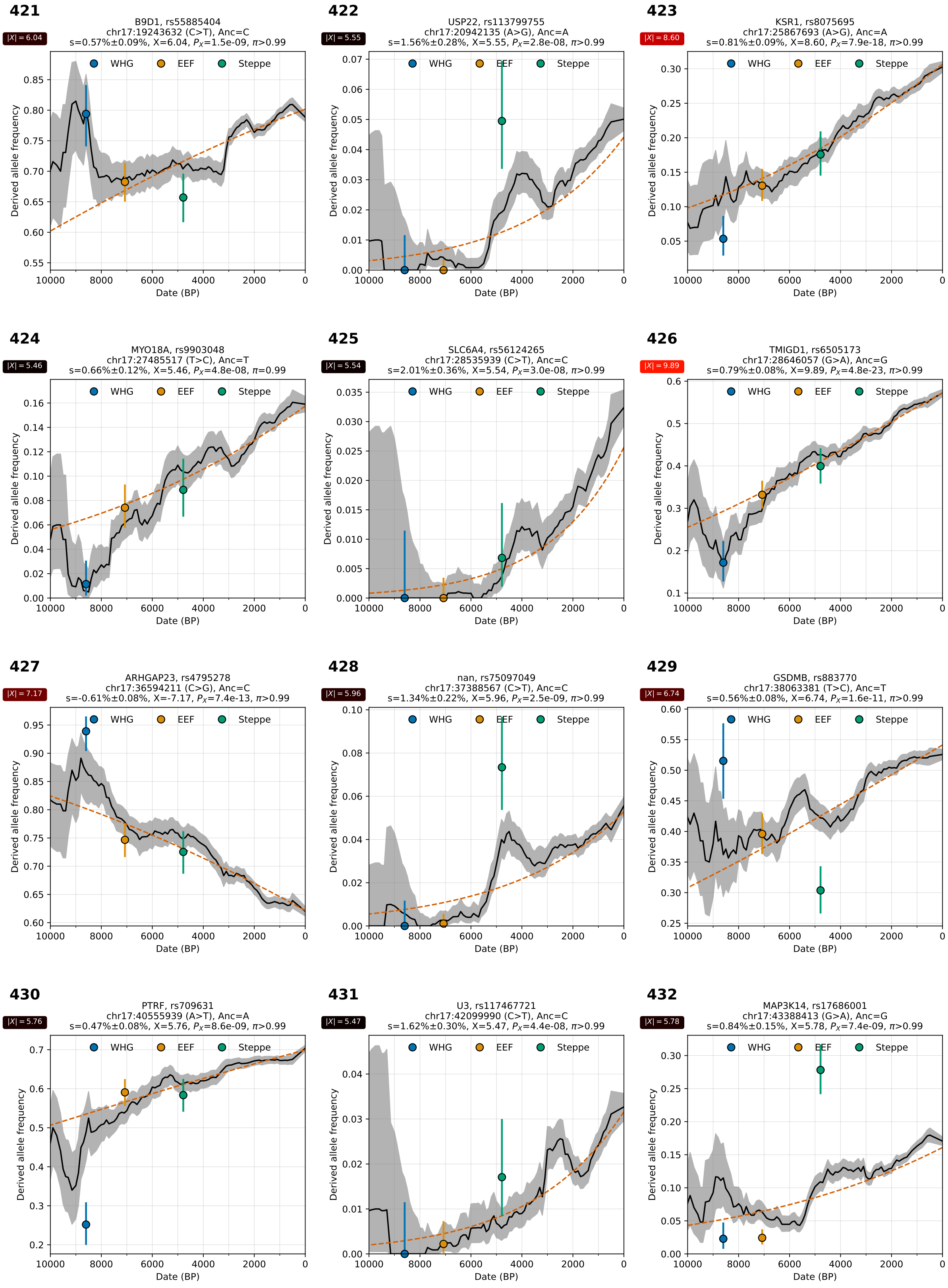

Supplementary Figure S5.36: Allele frequency over time.

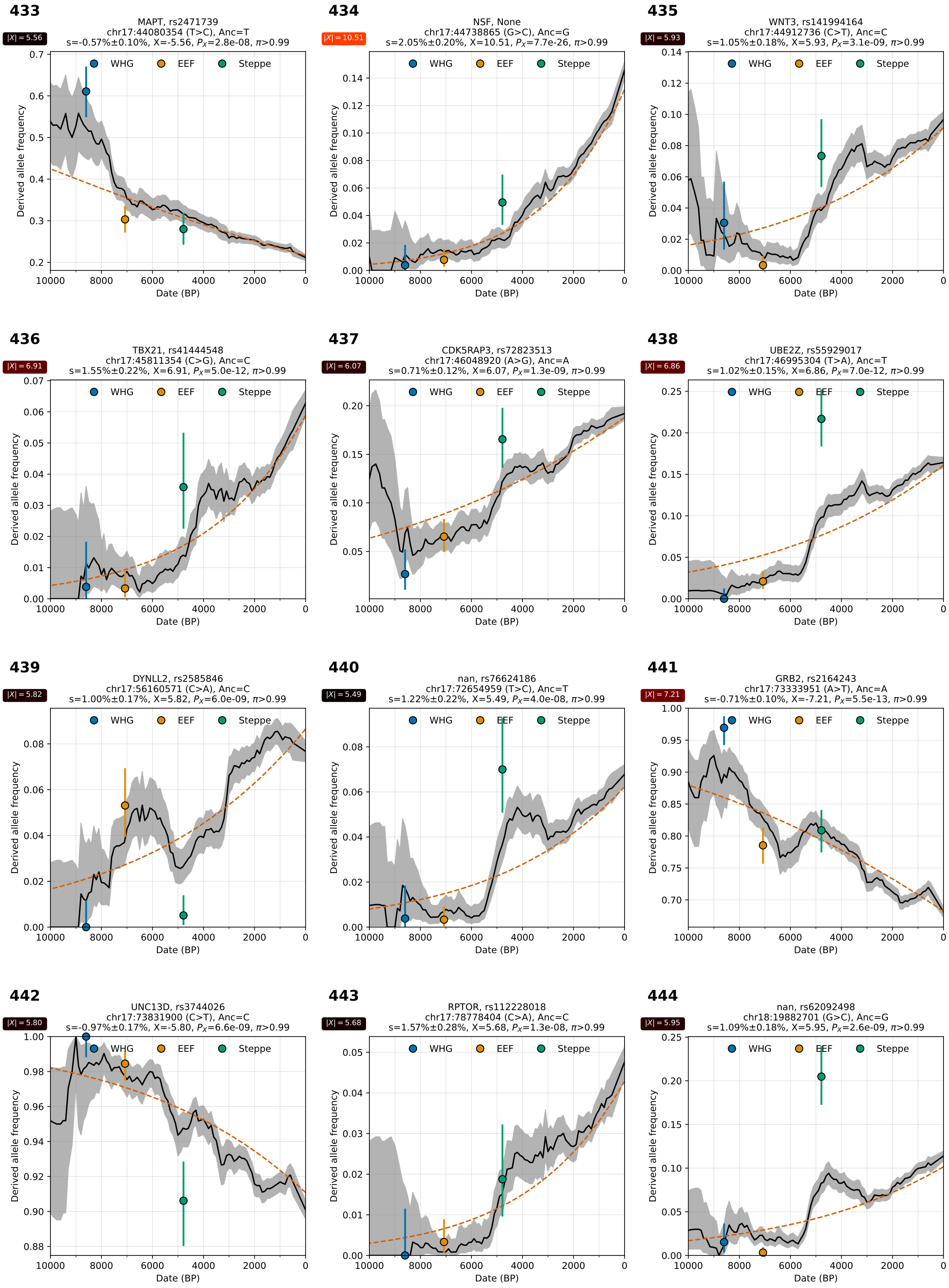

Supplementary Figure S5.37: Allele frequency over time.

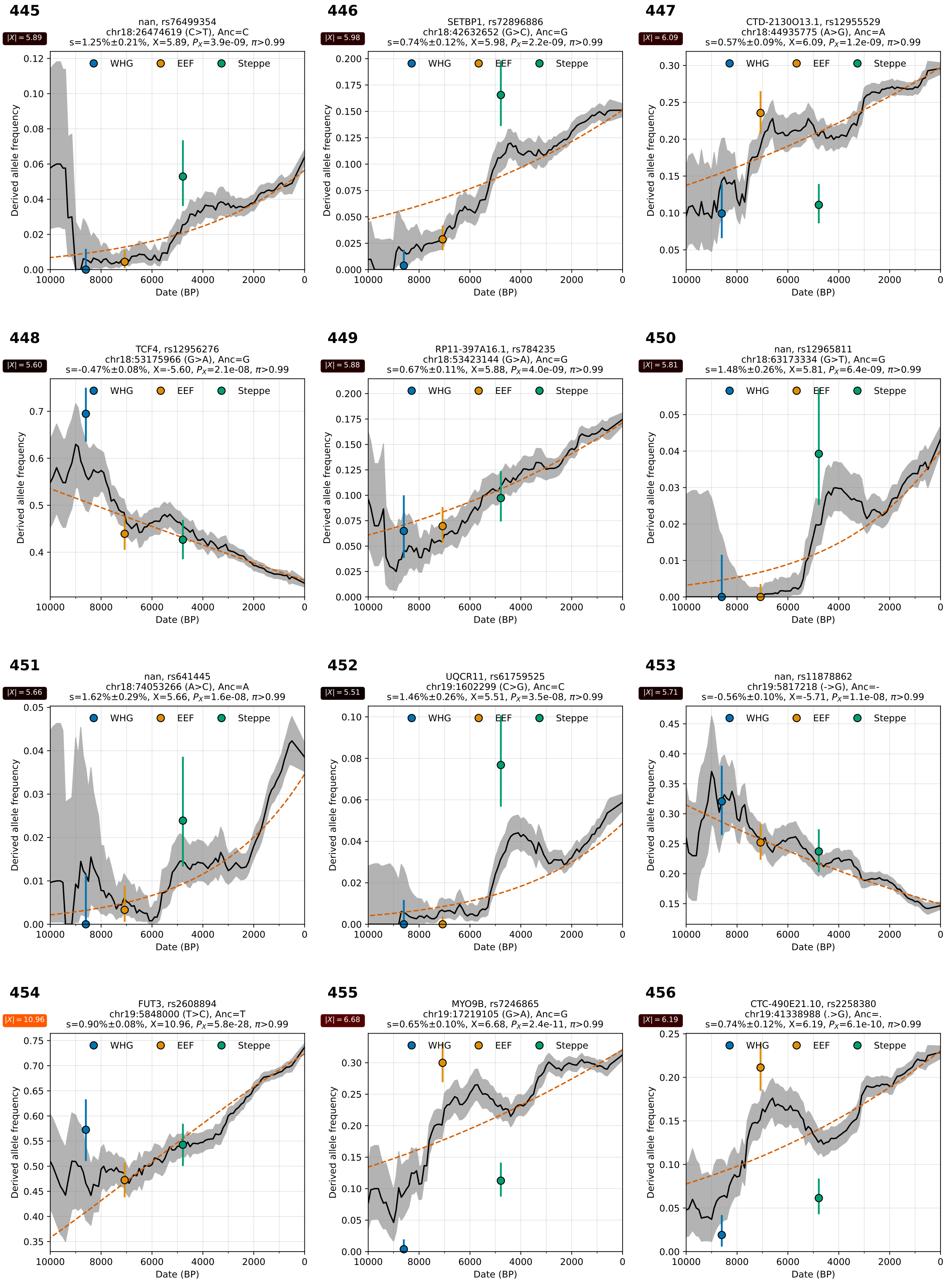

Supplementary Figure S5.38: Allele frequency over time.

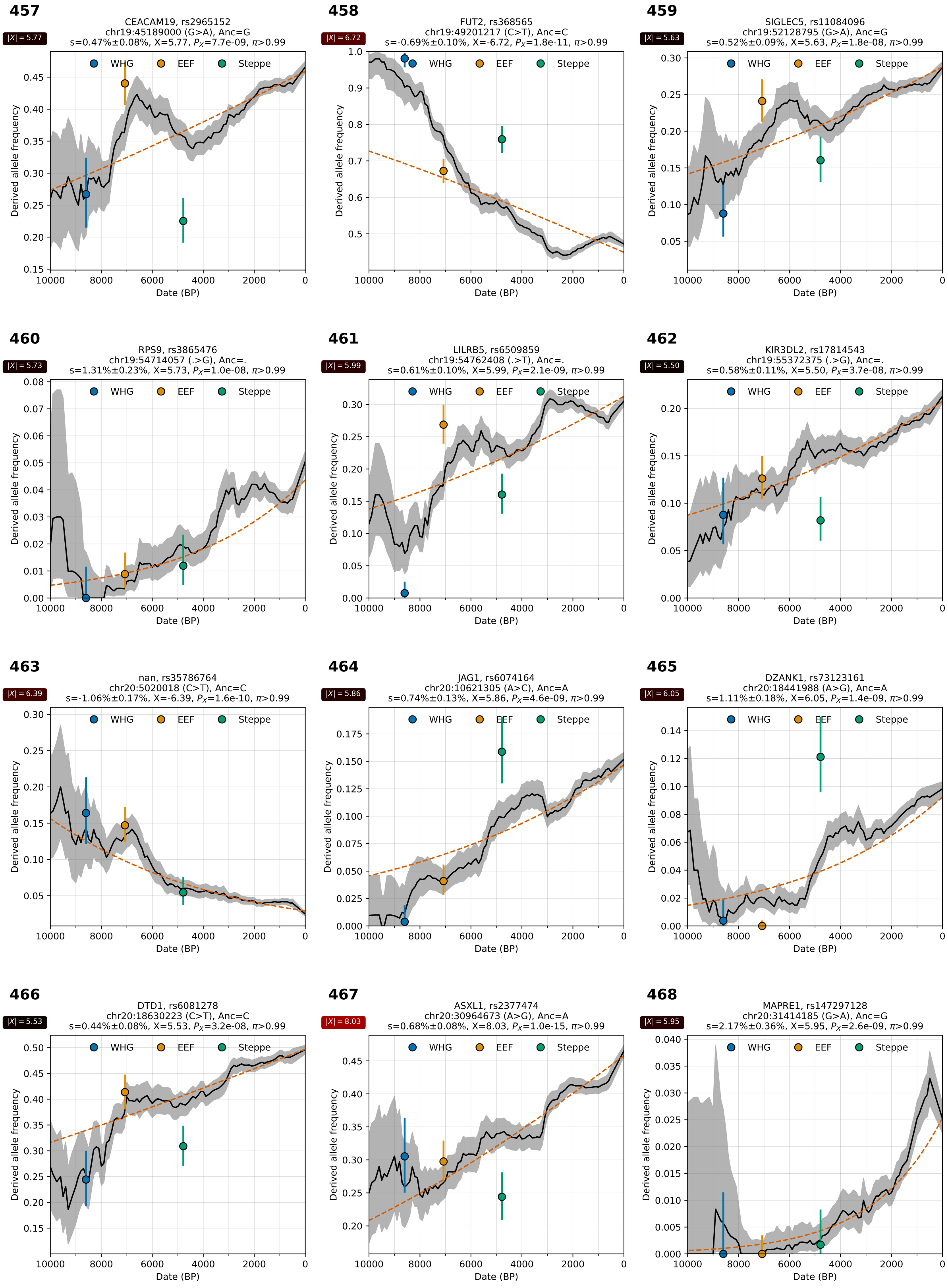

Supplementary Figure S5.39: Allele frequency over time.

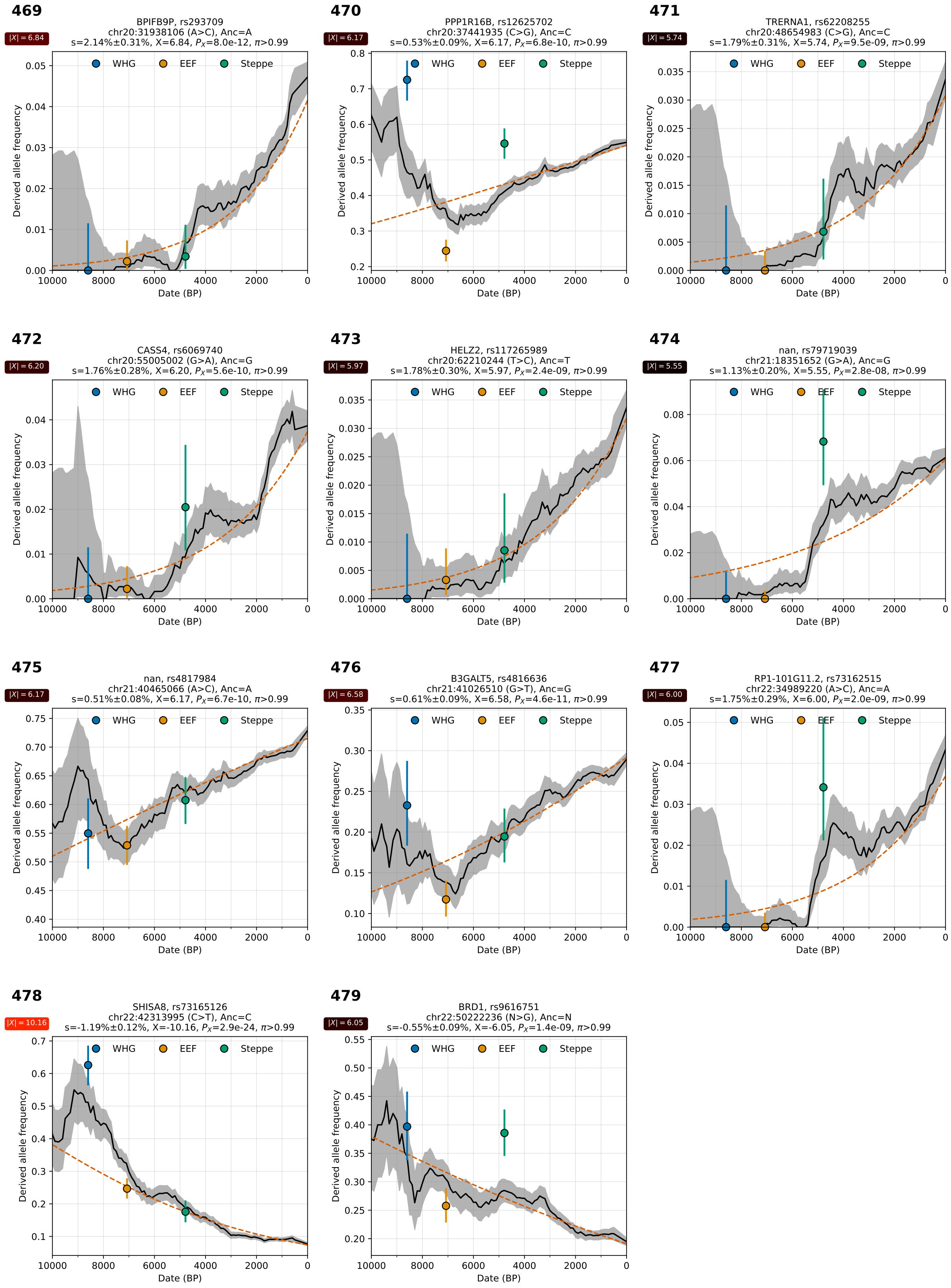

Supplementary Figure S5.40: Allele frequency over time.

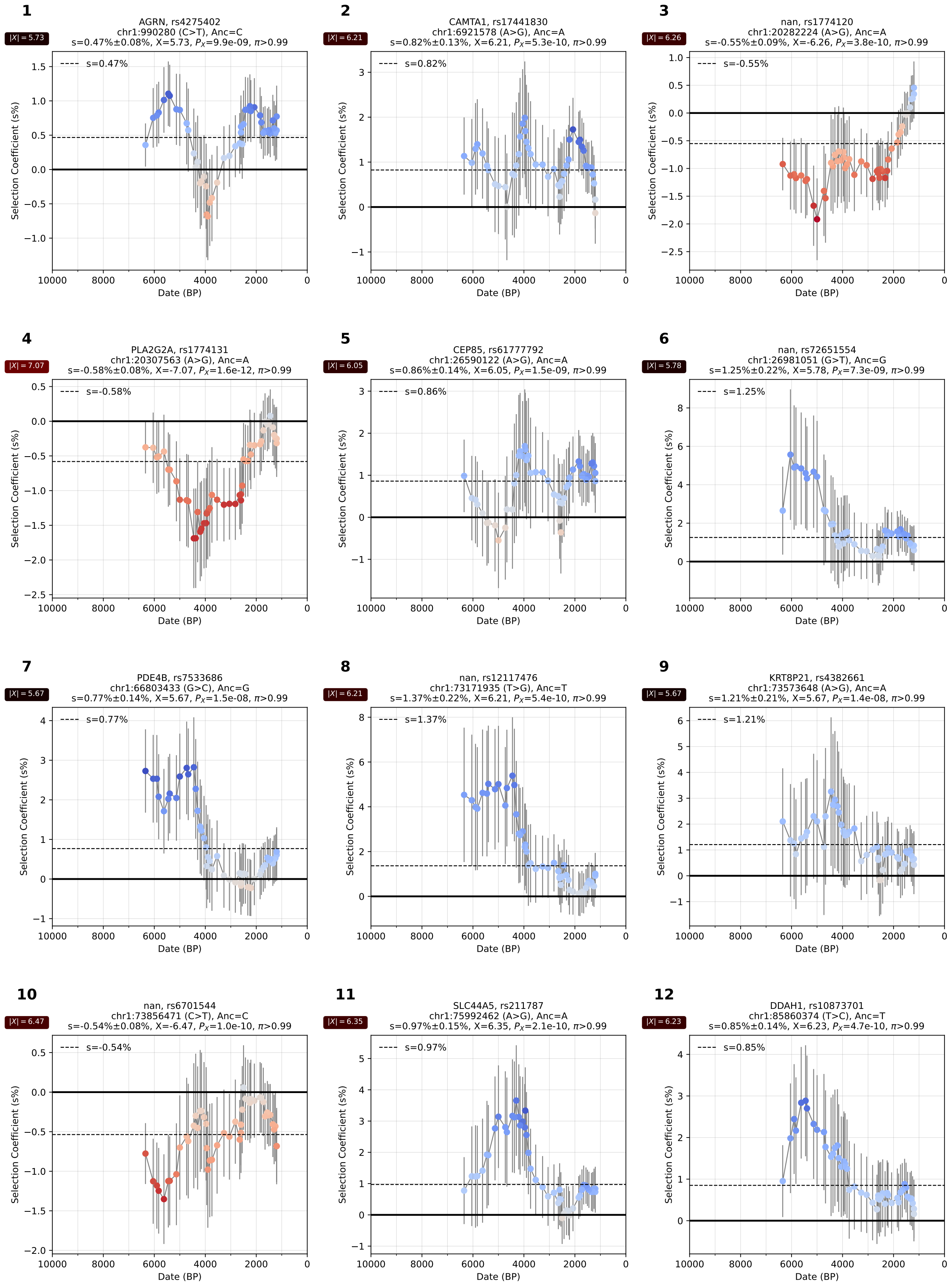

Supplementary Figure S5.41: Selection coefficient over time.

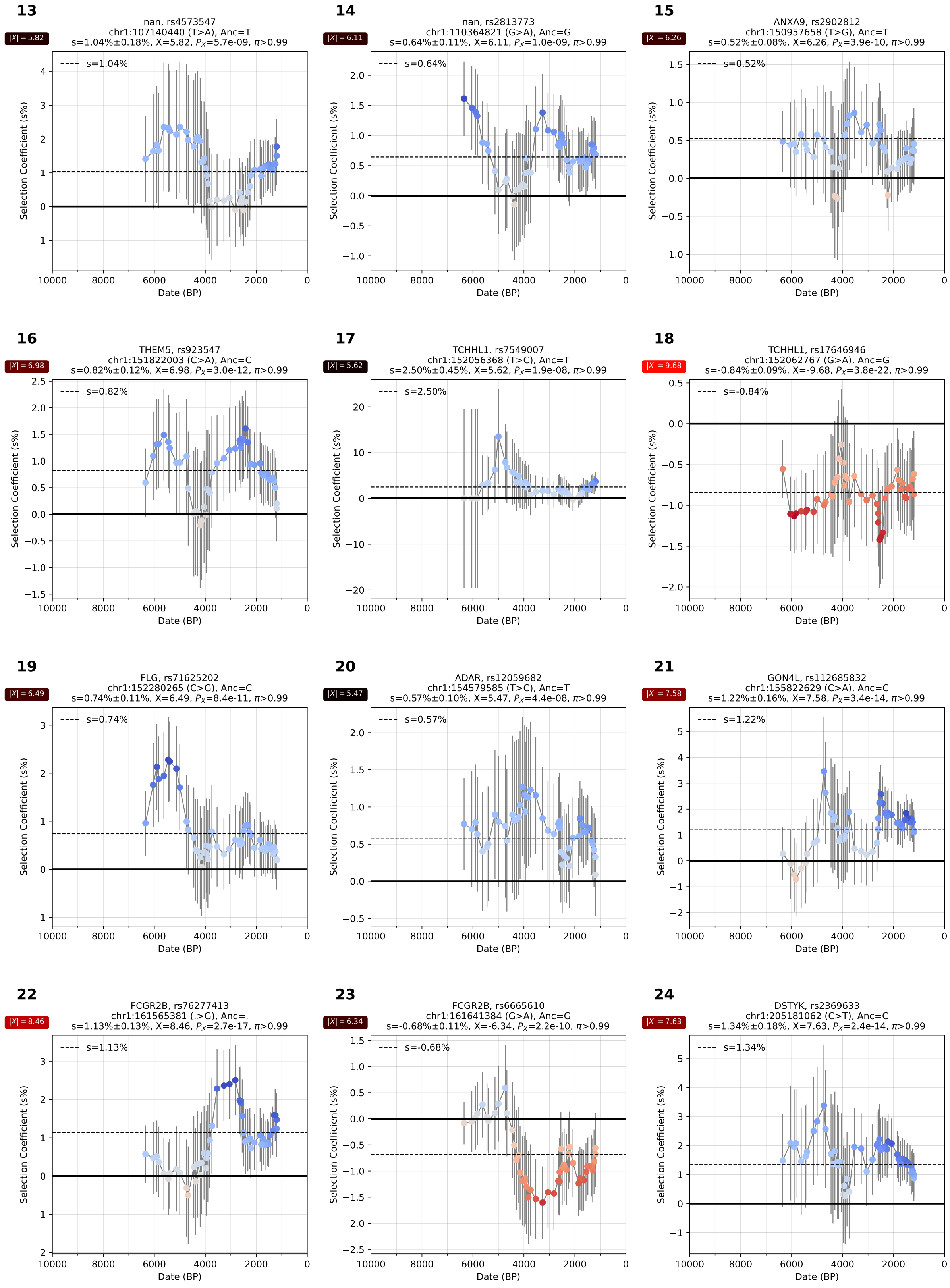

Supplementary Figure S5.42: Selection coefficient over time.

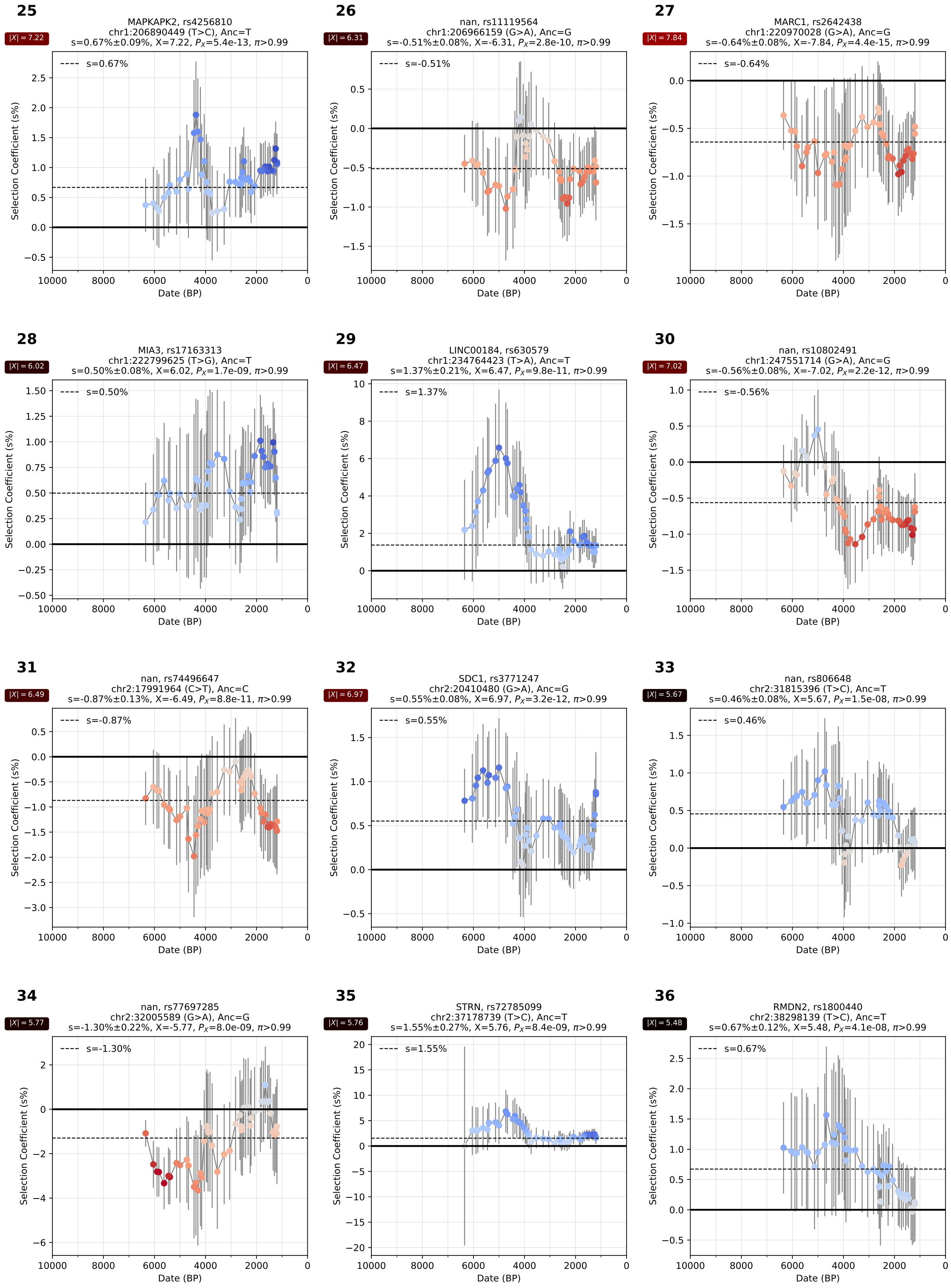

Supplementary Figure S5.43: Selection coefficient over time.

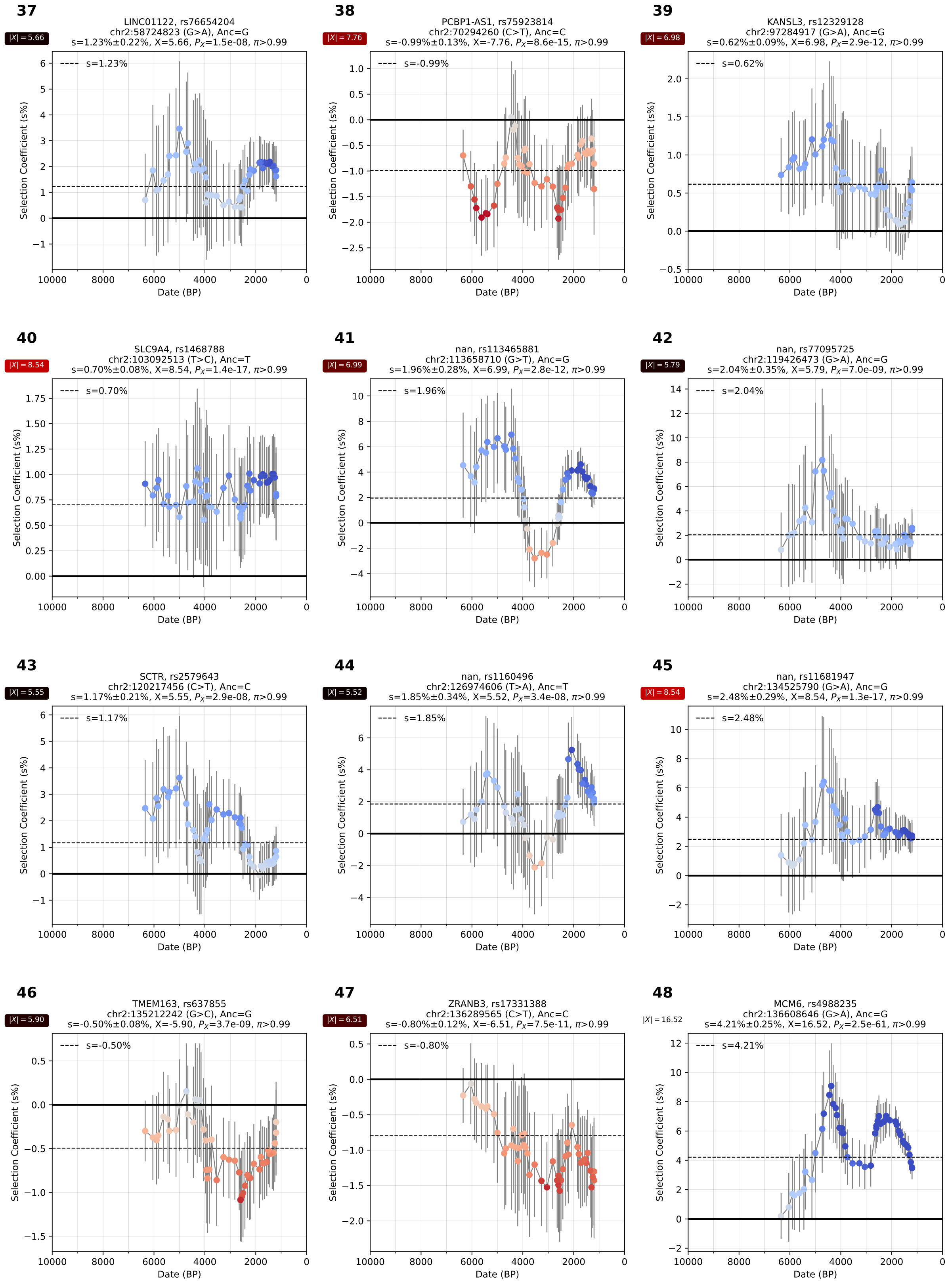

Supplementary Figure S5.44: Selection coefficient over time.

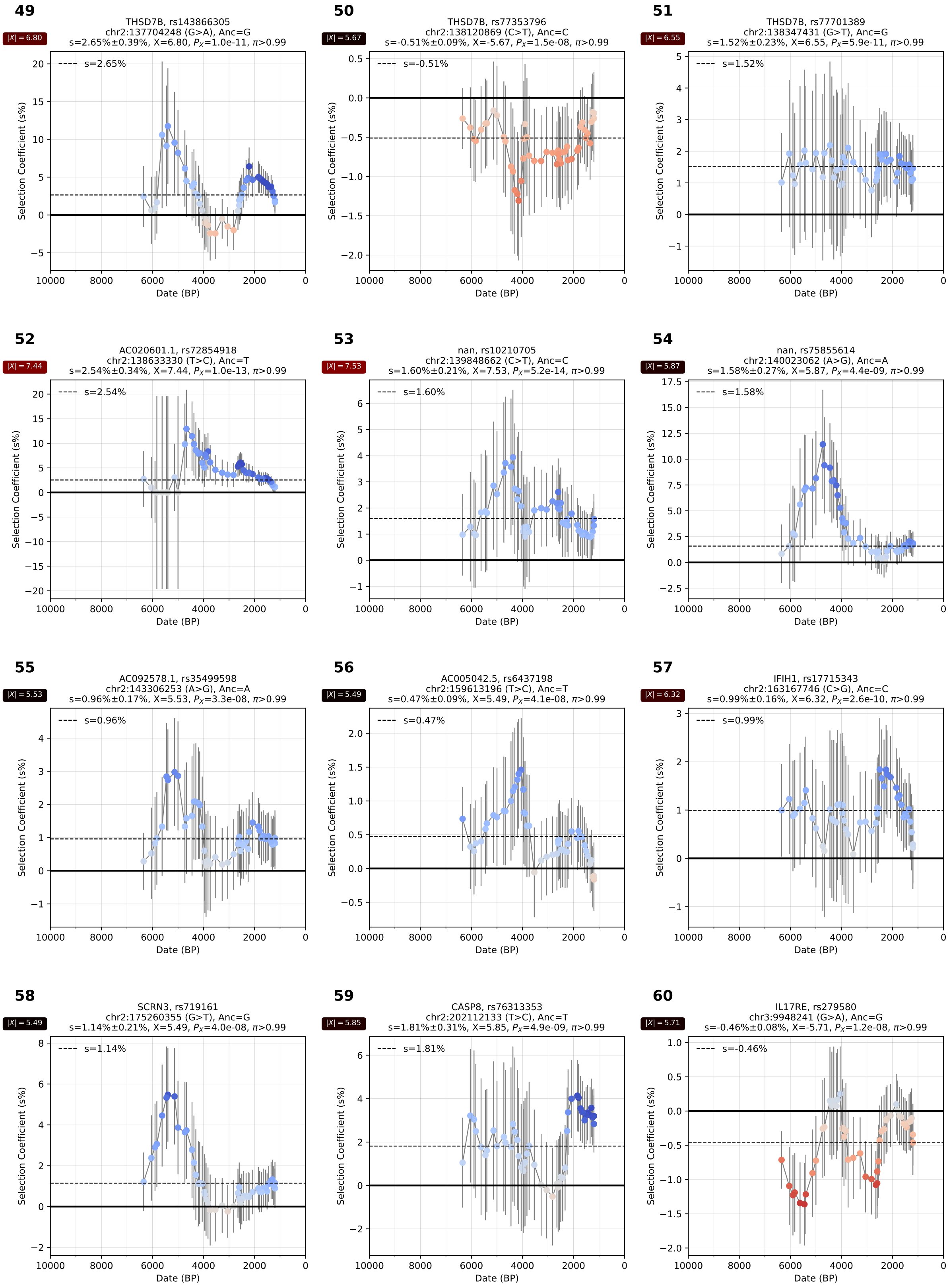

Supplementary Figure S5.45: Selection coefficient over time.

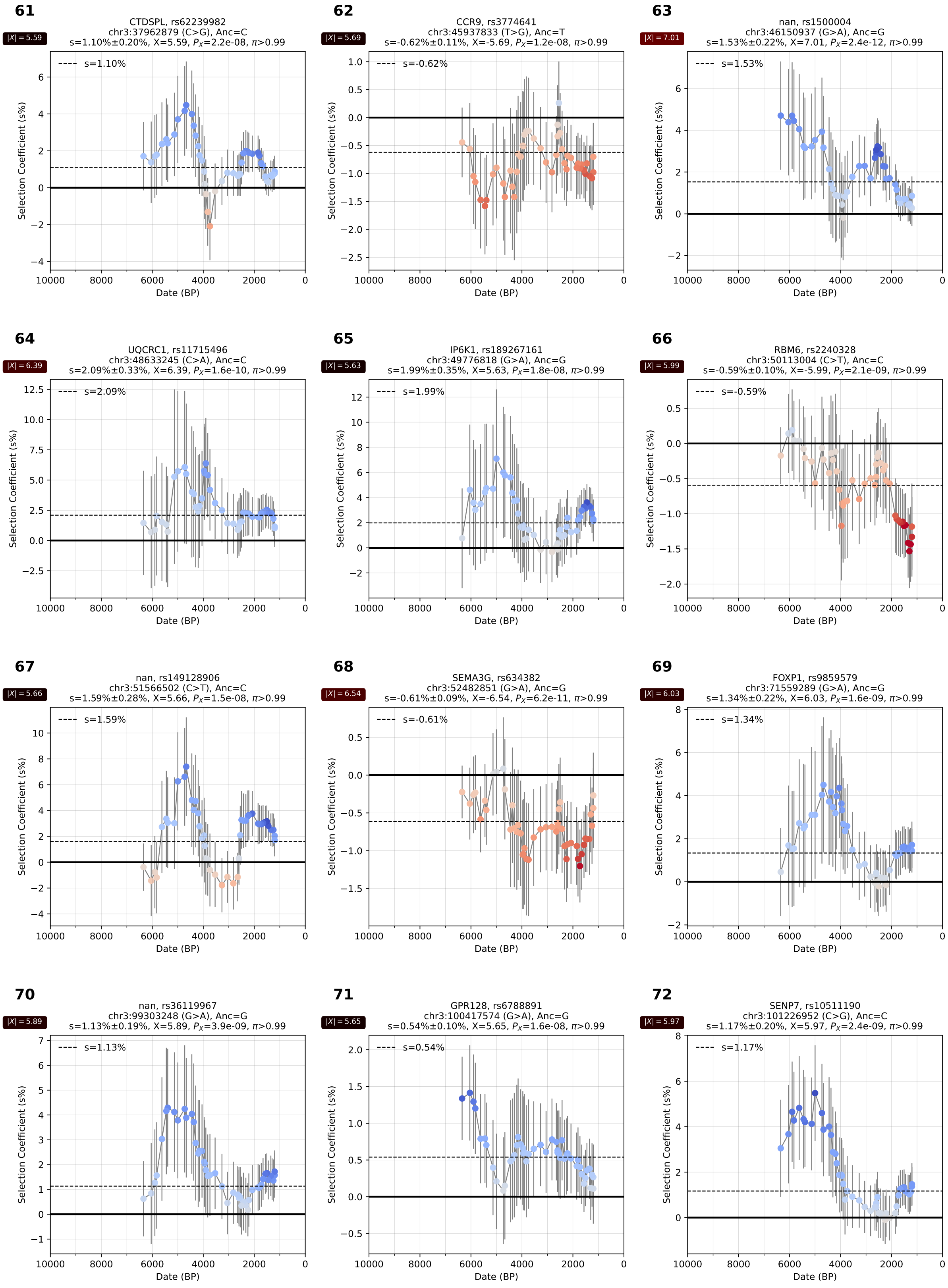

Supplementary Figure S5.46: Selection coefficient over time.

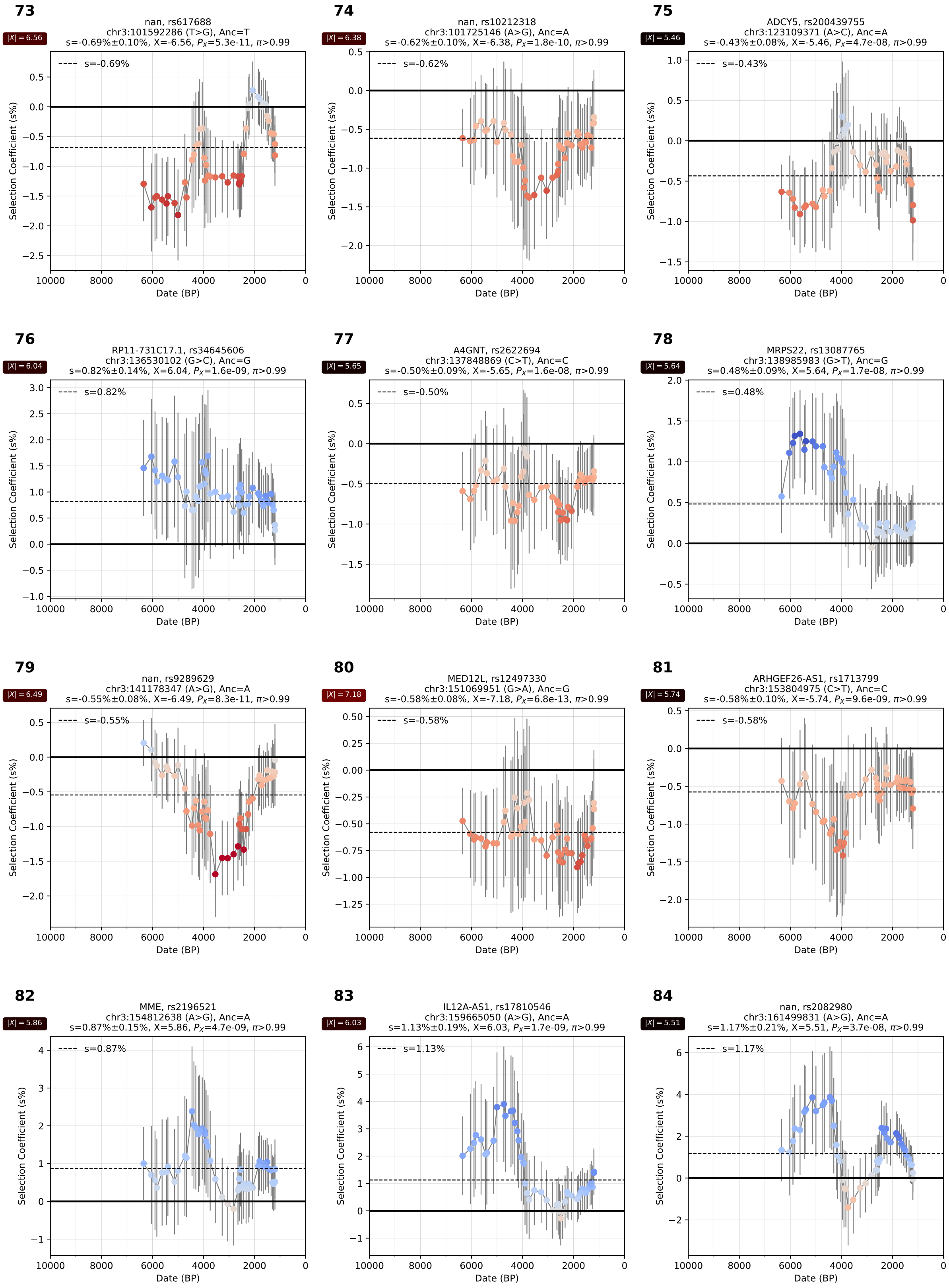

Supplementary Figure S5.47: Selection coefficient over time.

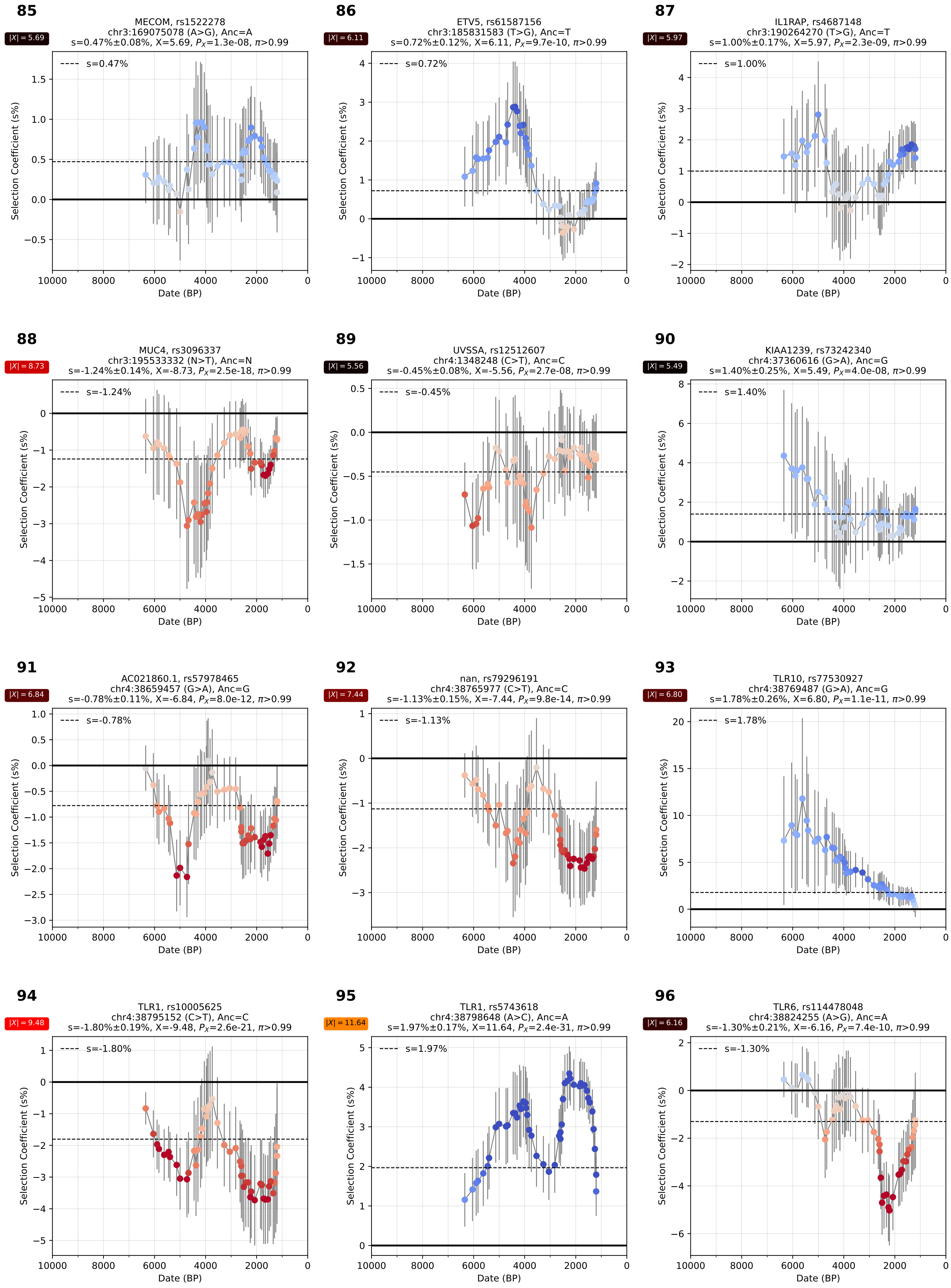

Supplementary Figure S5.48: Selection coefficient over time.

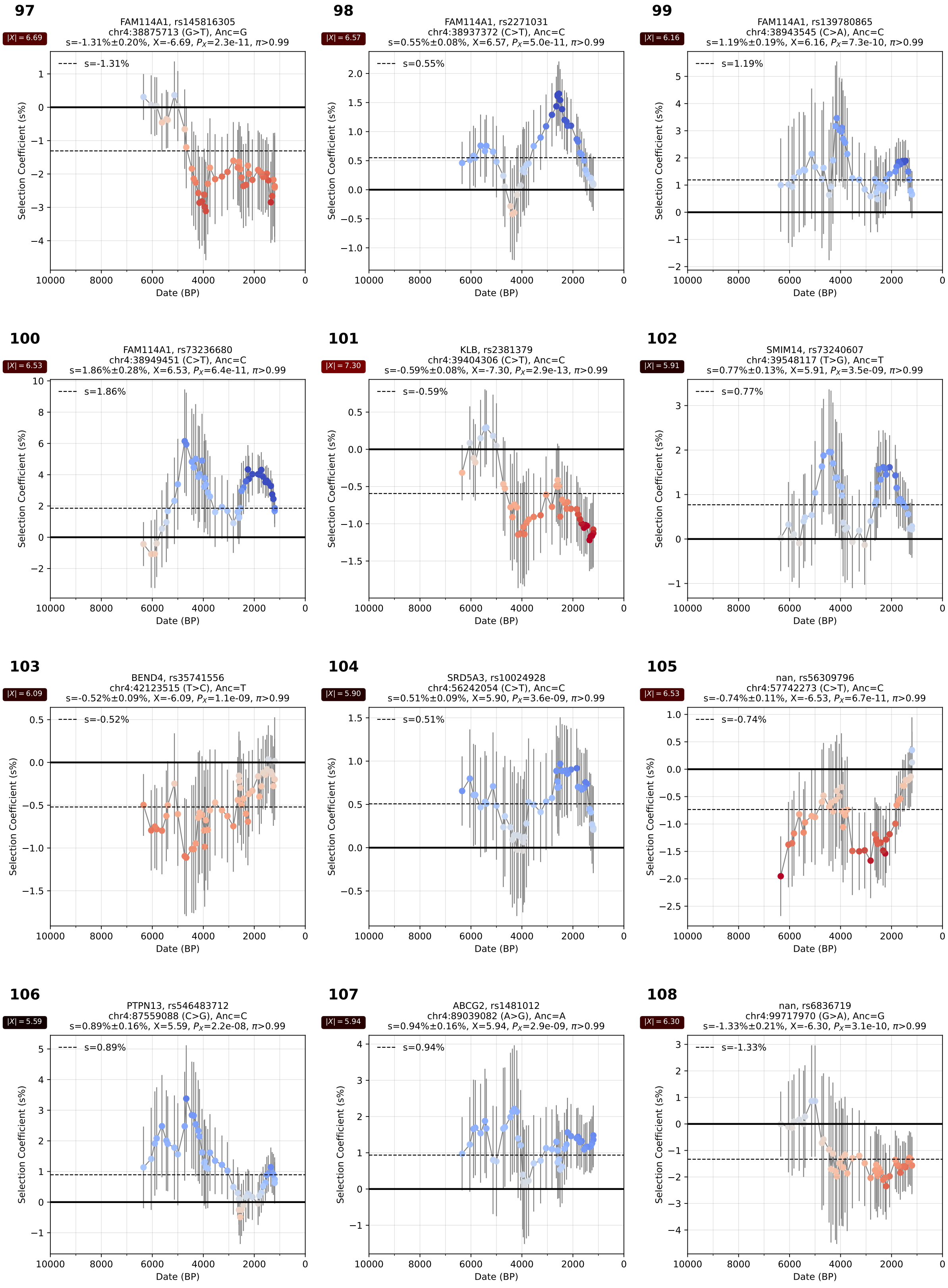

Supplementary Figure S5.49: Selection coefficient over time.

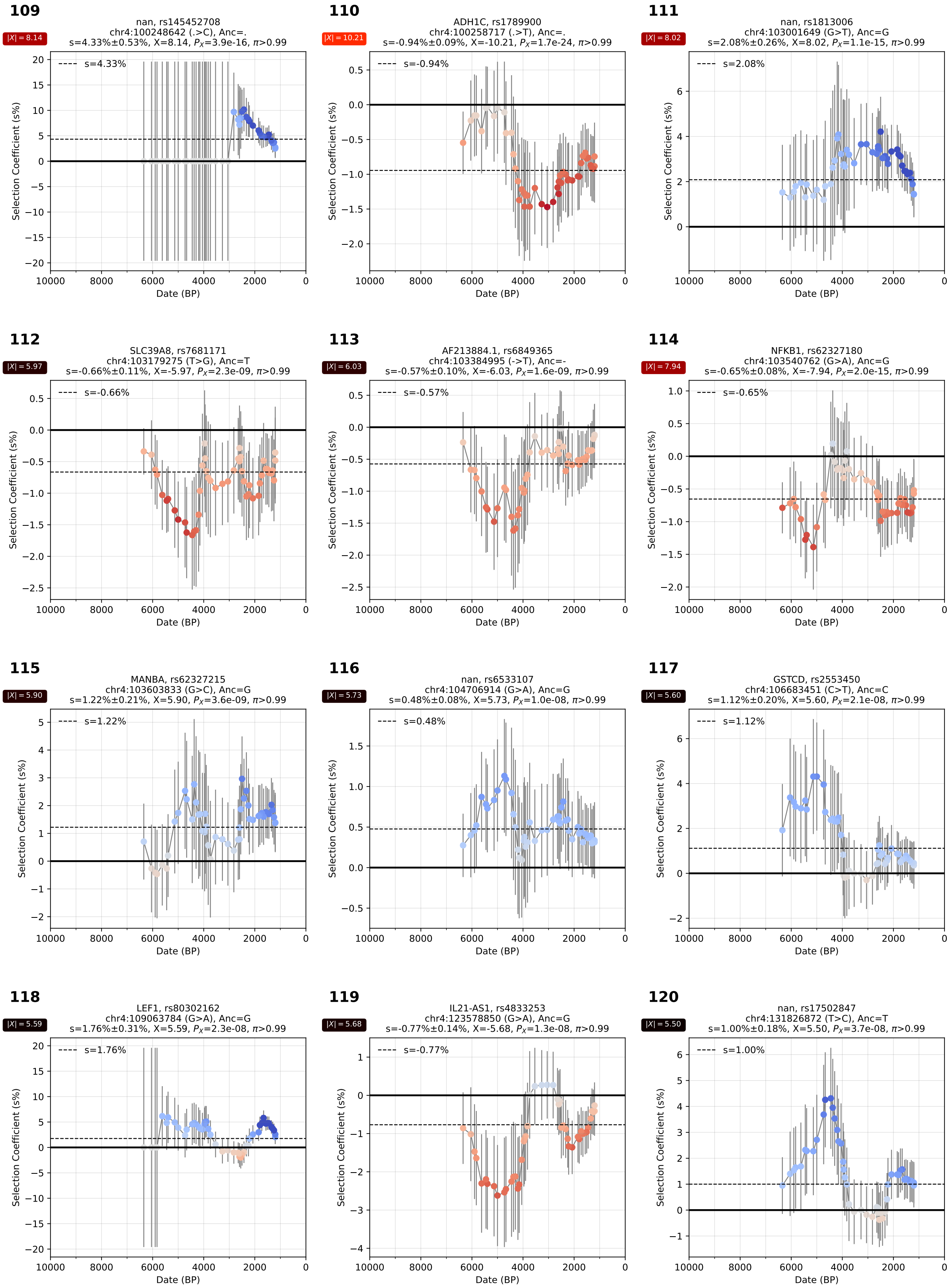

Supplementary Figure S5.50: Selection coefficient over time.

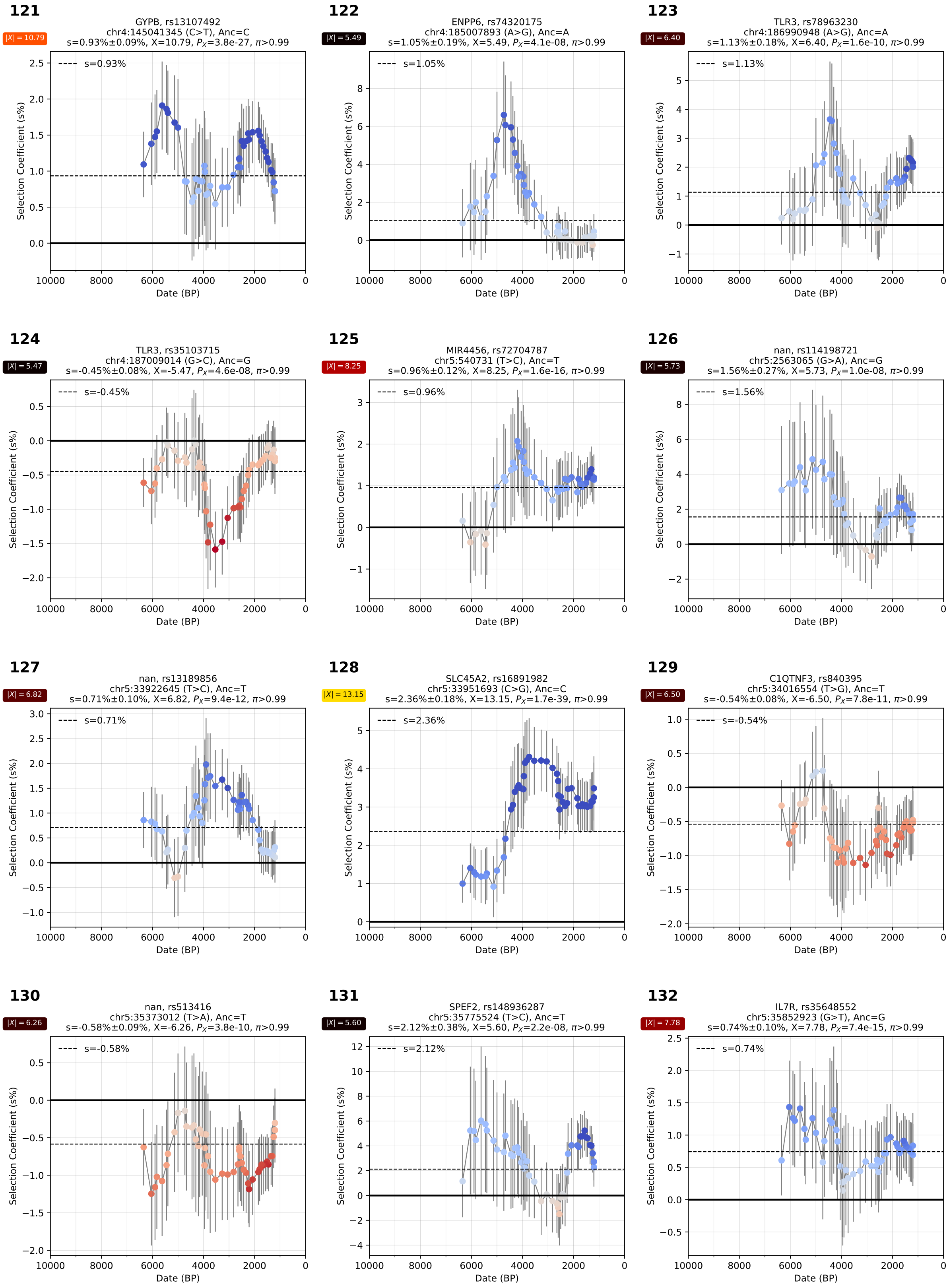

Supplementary Figure S5.51: Selection coefficient over time.

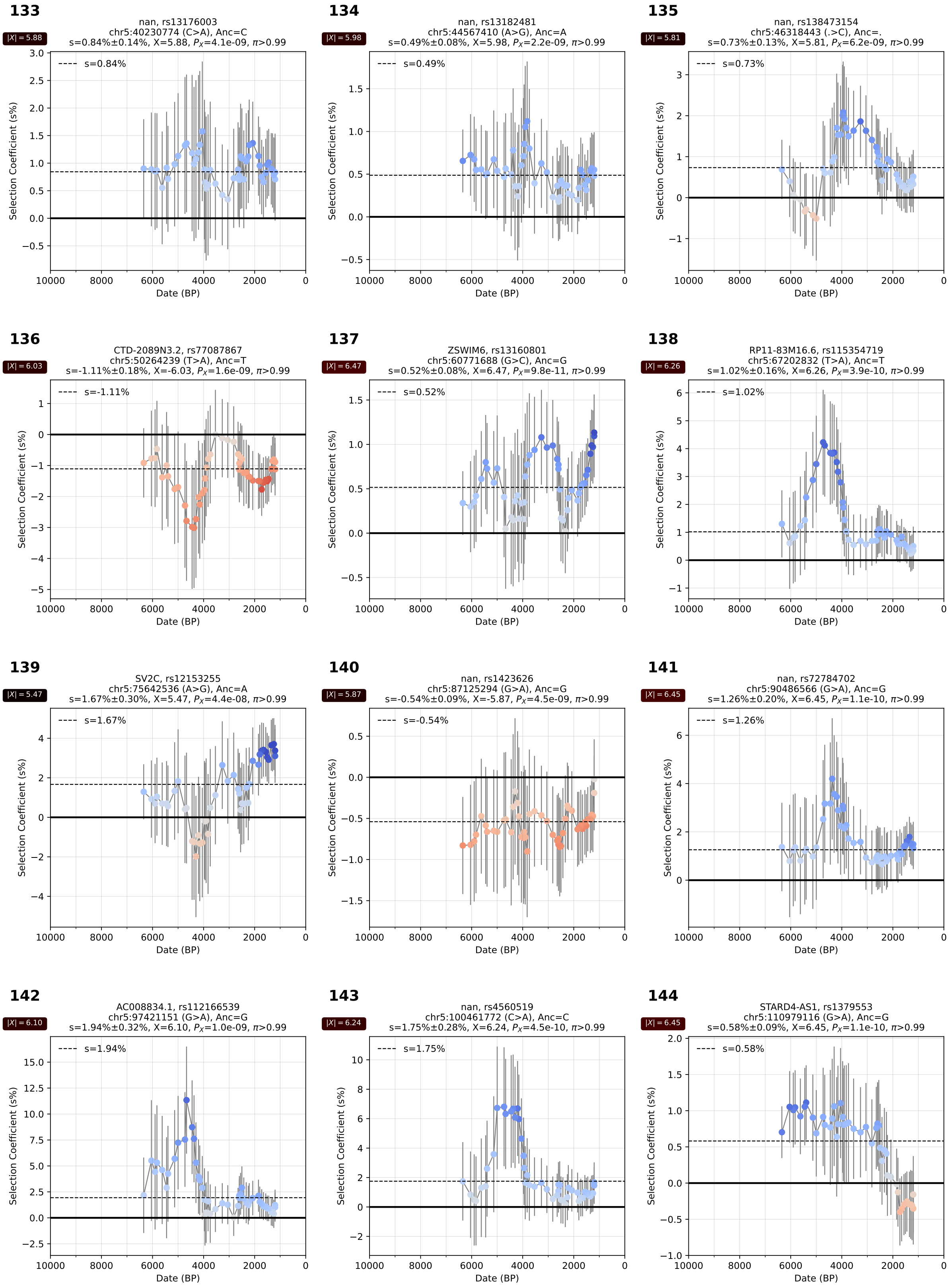

Supplementary Figure S5.52: Selection coefficient over time.

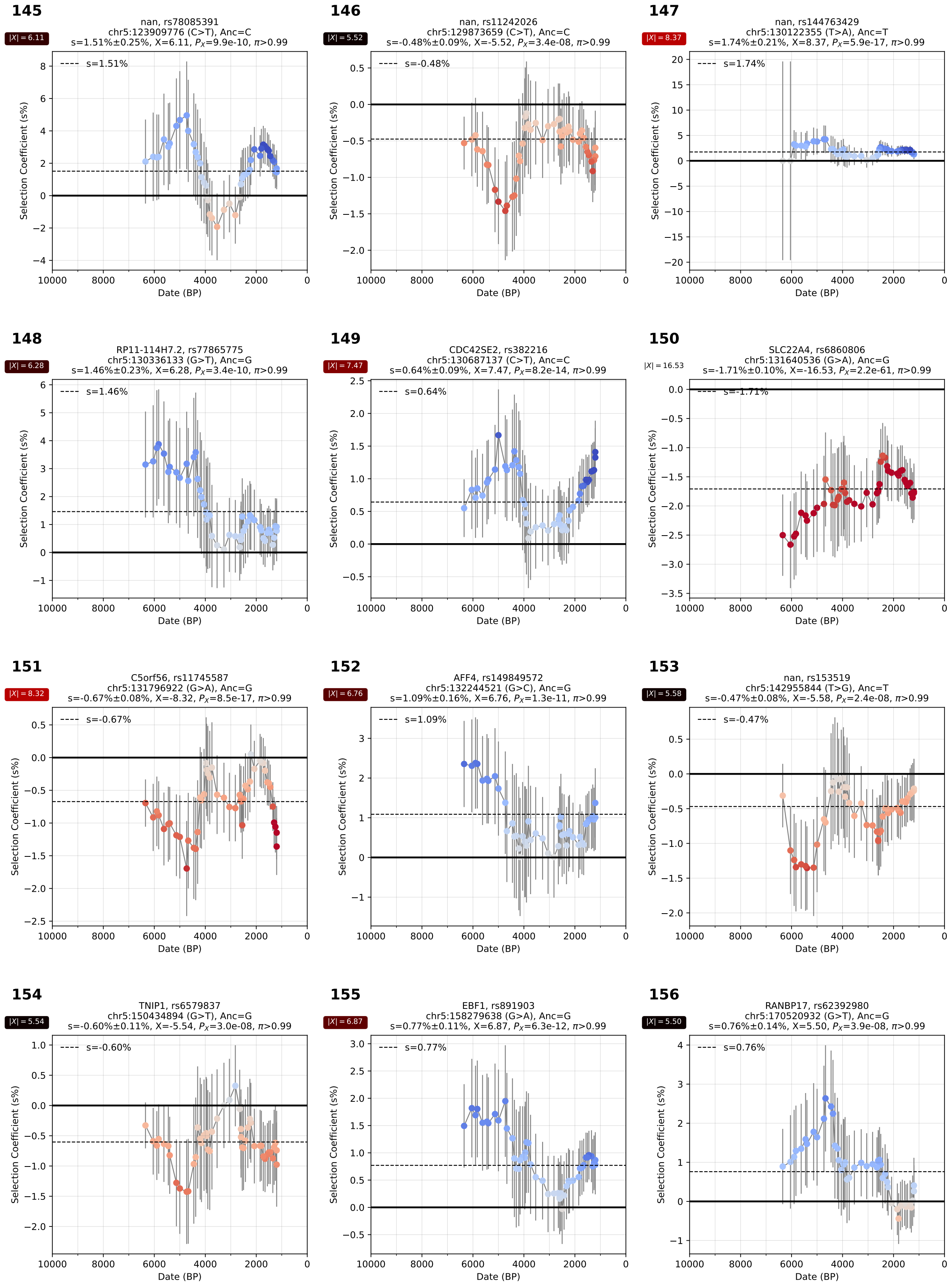

Supplementary Figure S5.53: Selection coefficient over time.

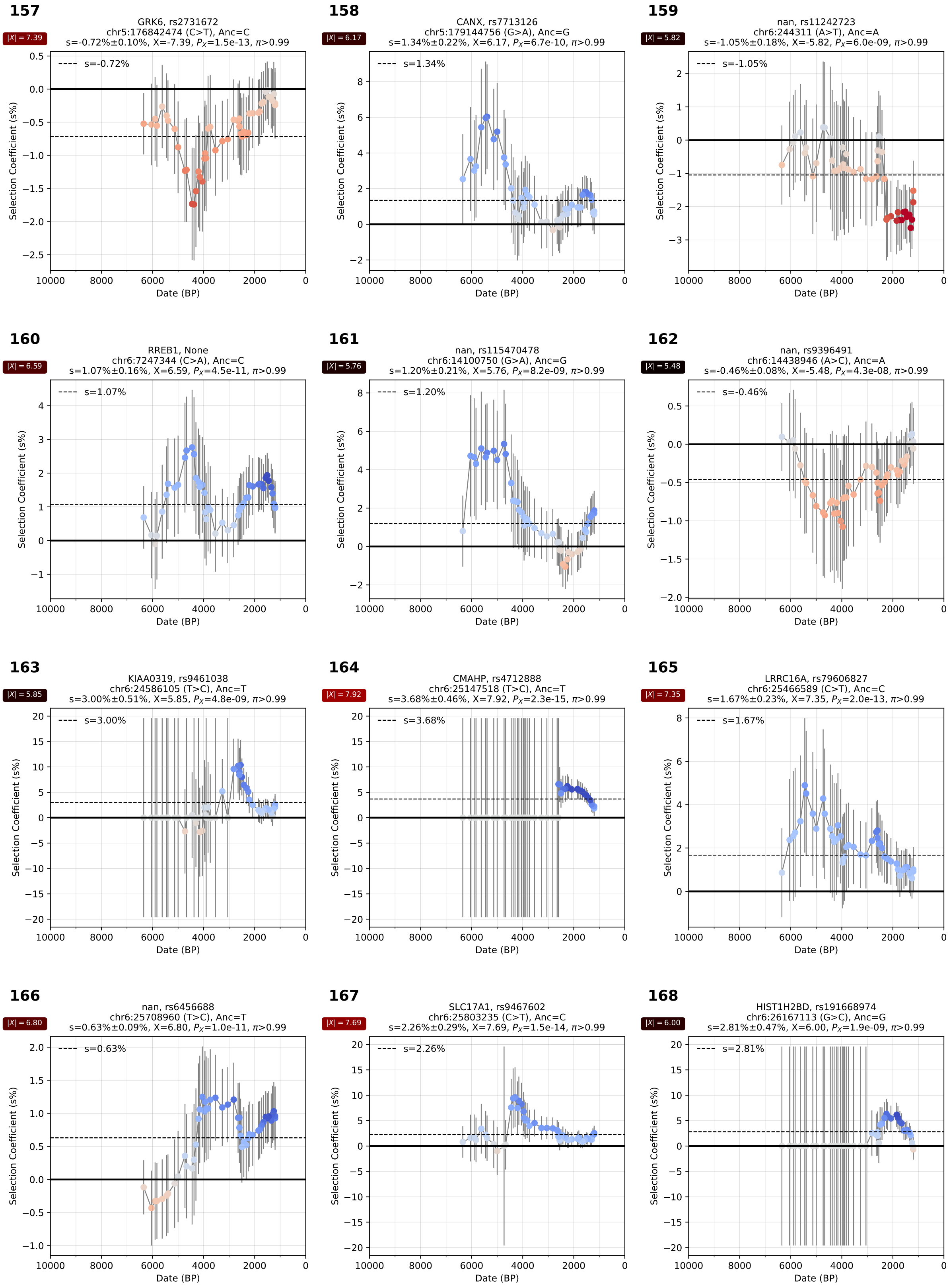

Supplementary Figure S5.54: Selection coefficient over time.

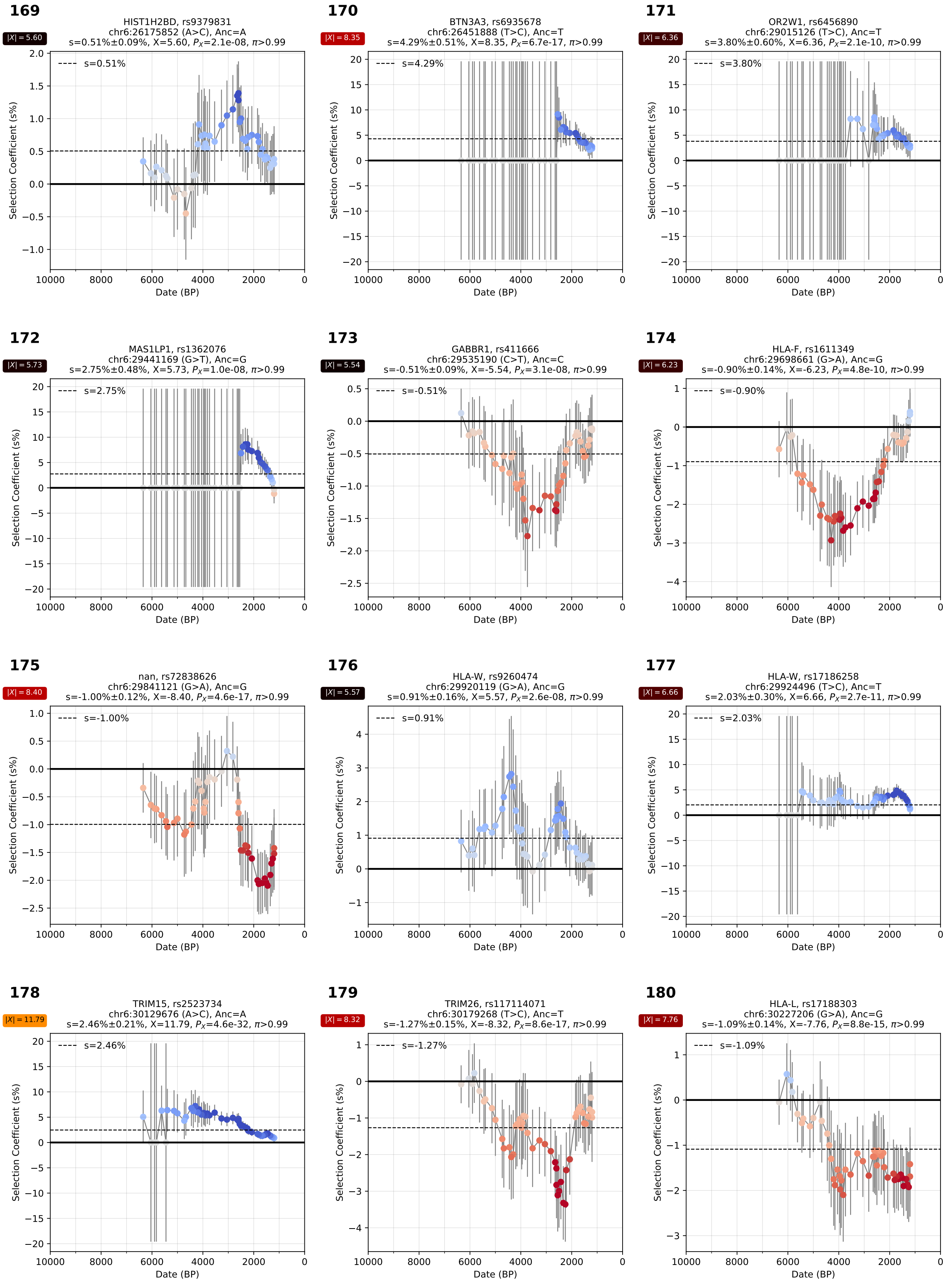

Supplementary Figure S5.55: Selection coefficient over time.

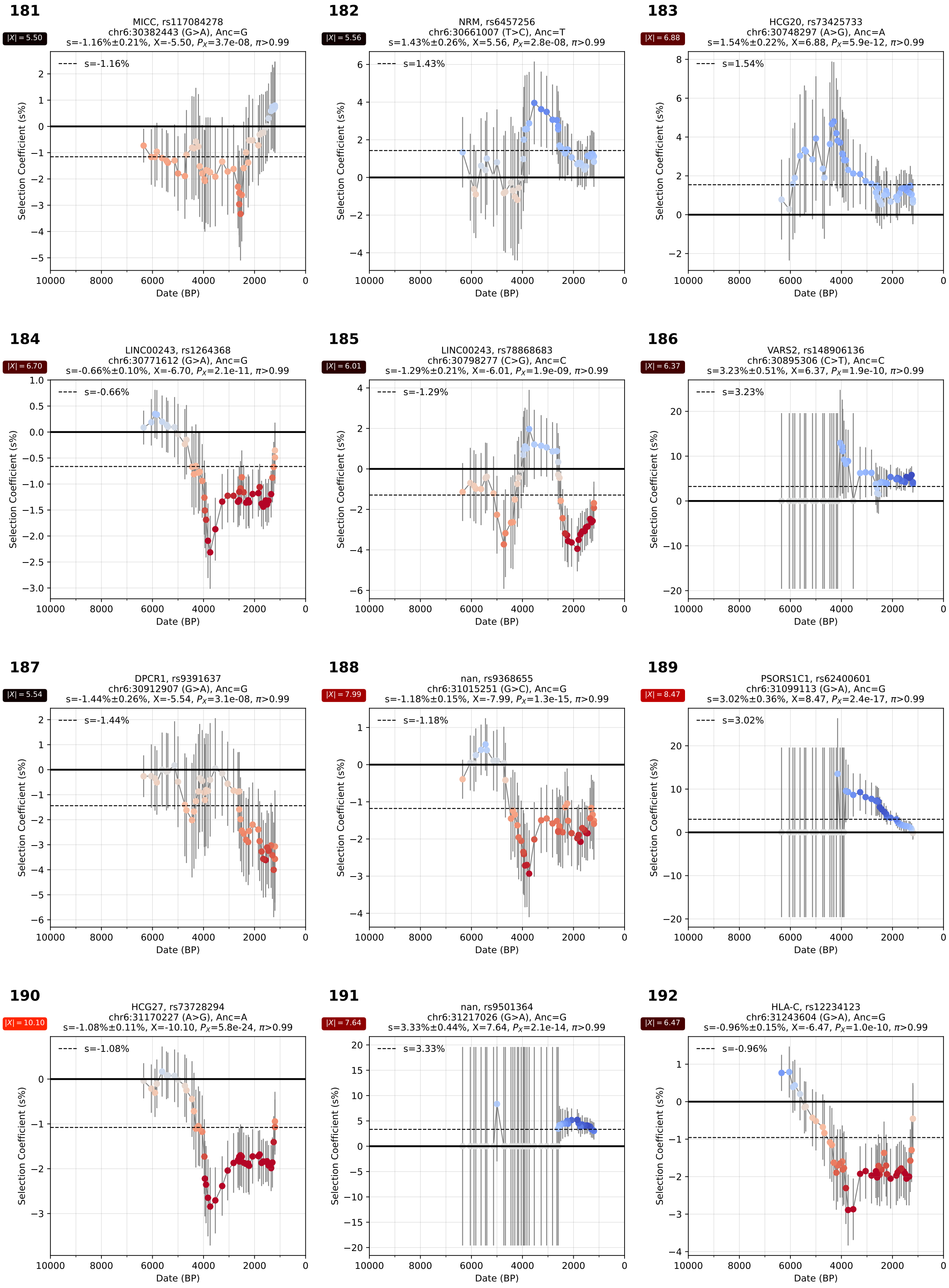

Supplementary Figure S5.56: Selection coefficient over time.

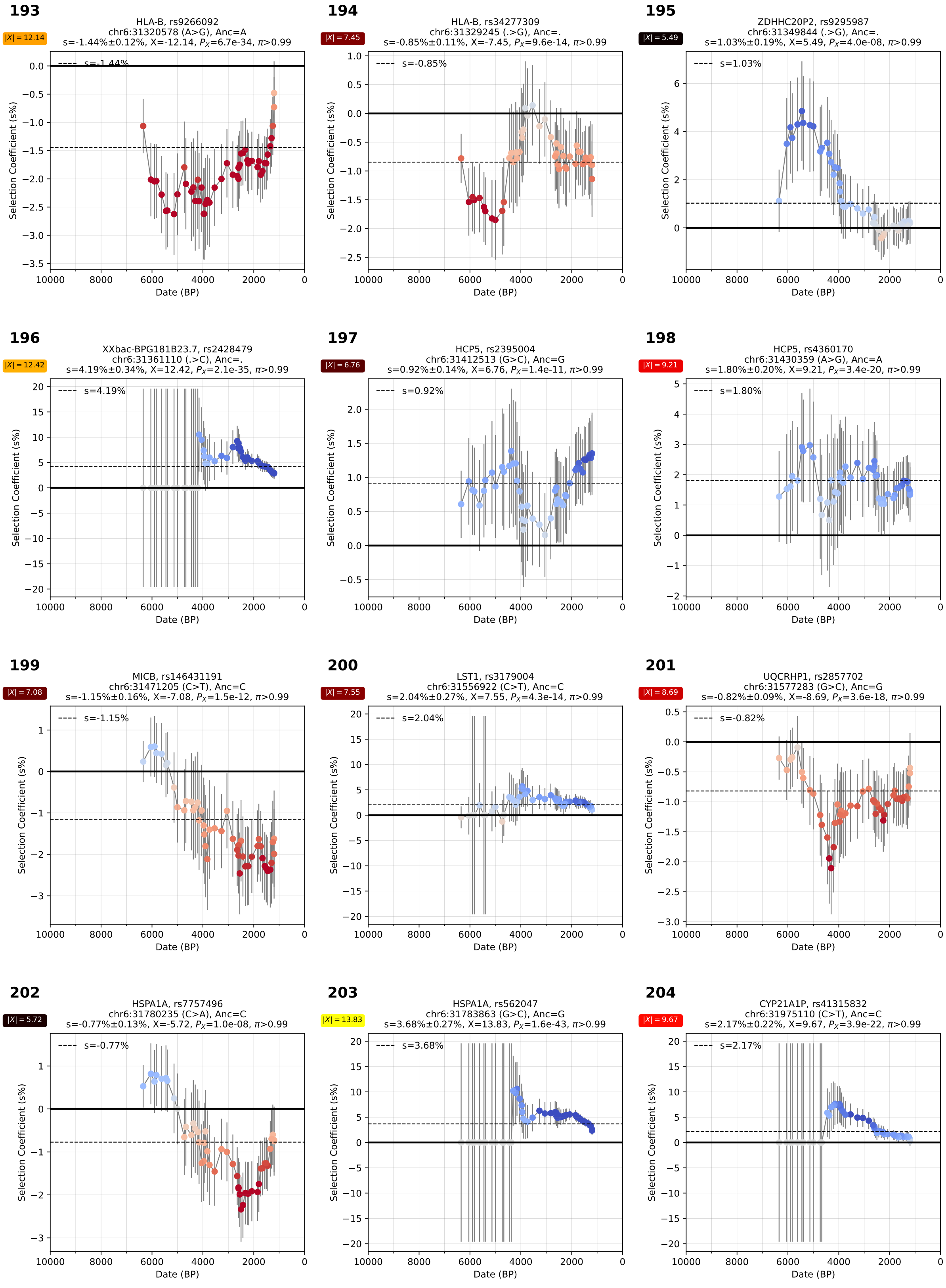

Supplementary Figure S5.57: Selection coefficient over time.

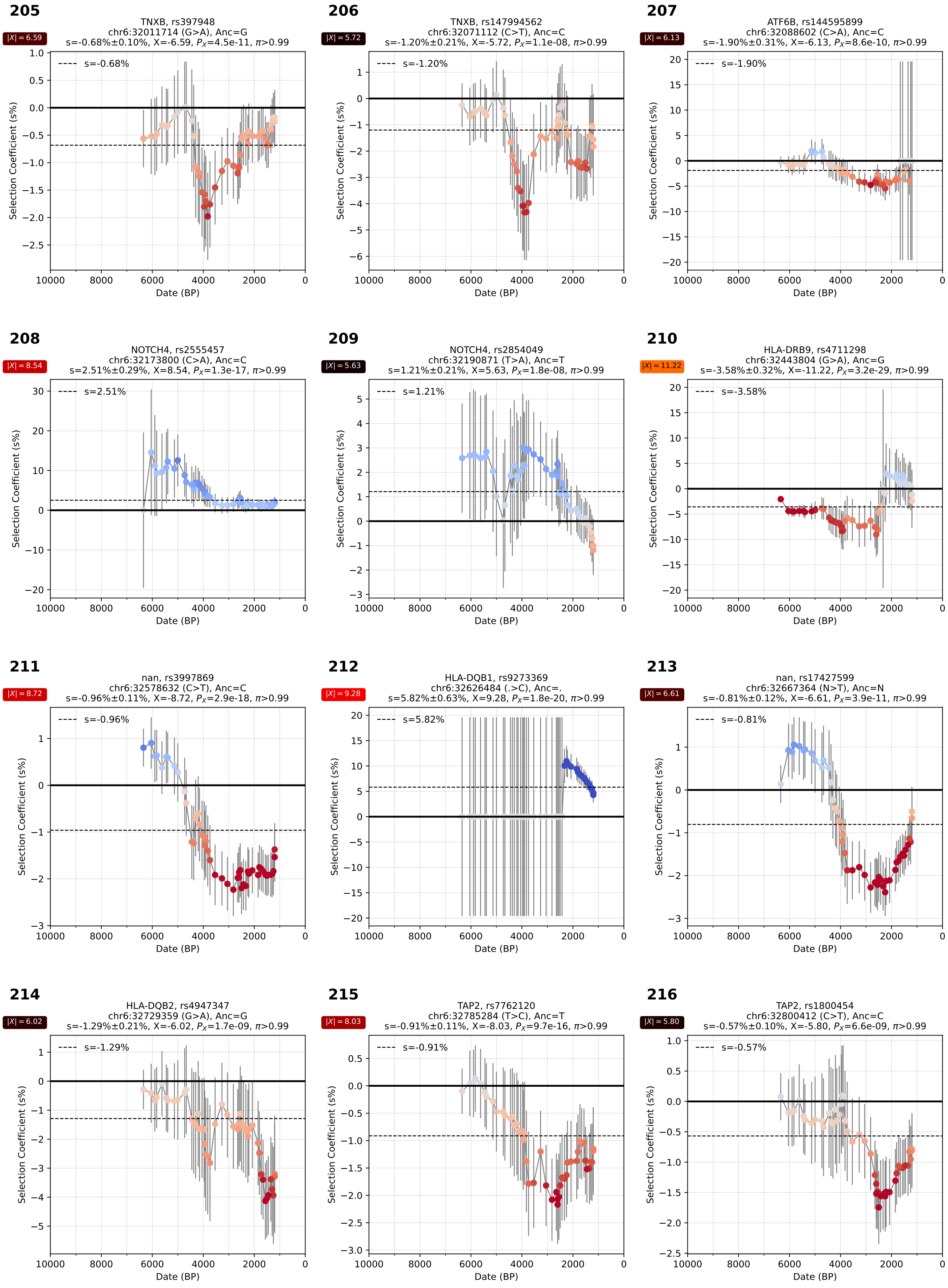

Supplementary Figure S5.58: Selection coefficient over time.

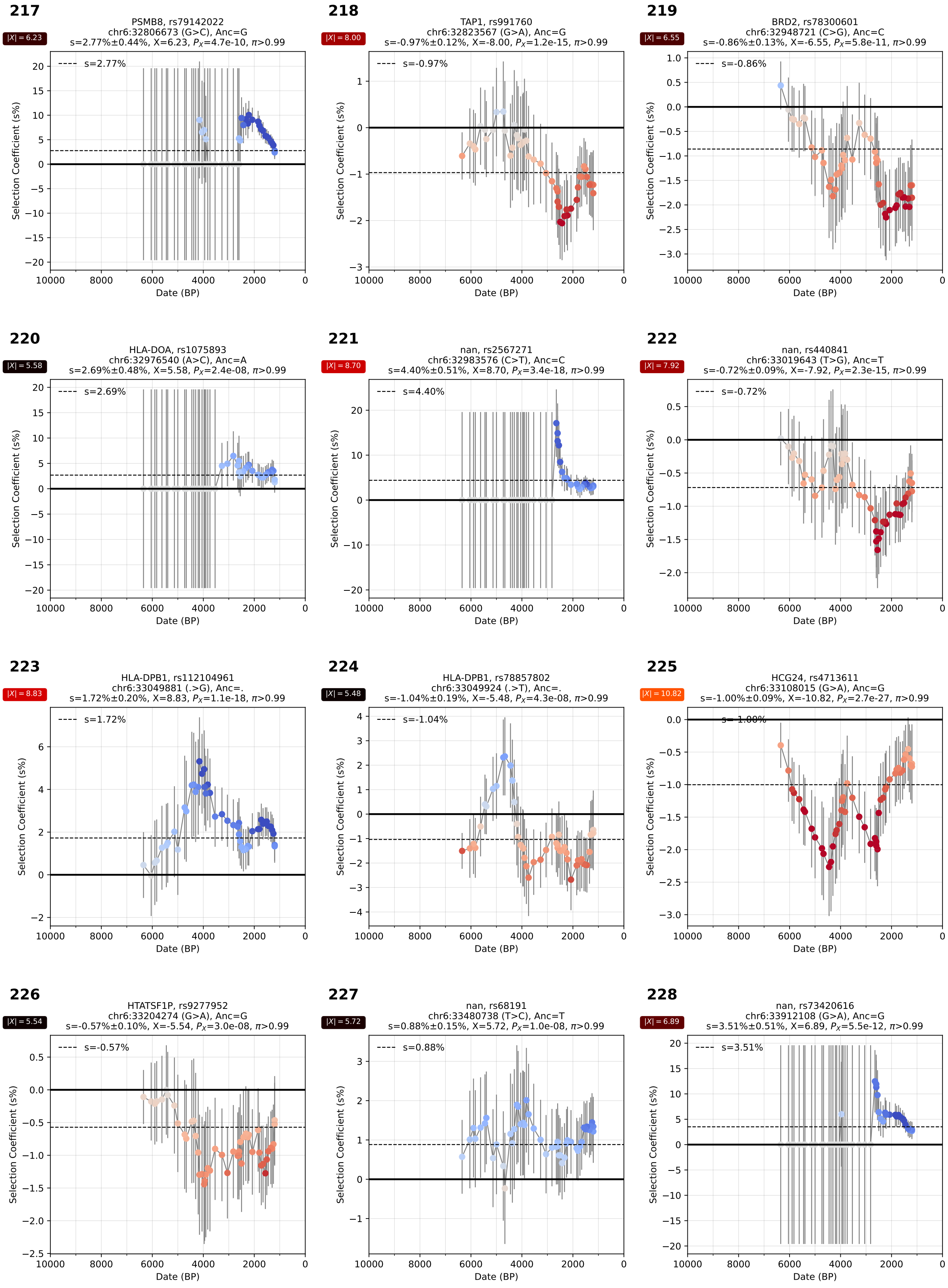

Supplementary Figure S5.59: Selection coefficient over time.

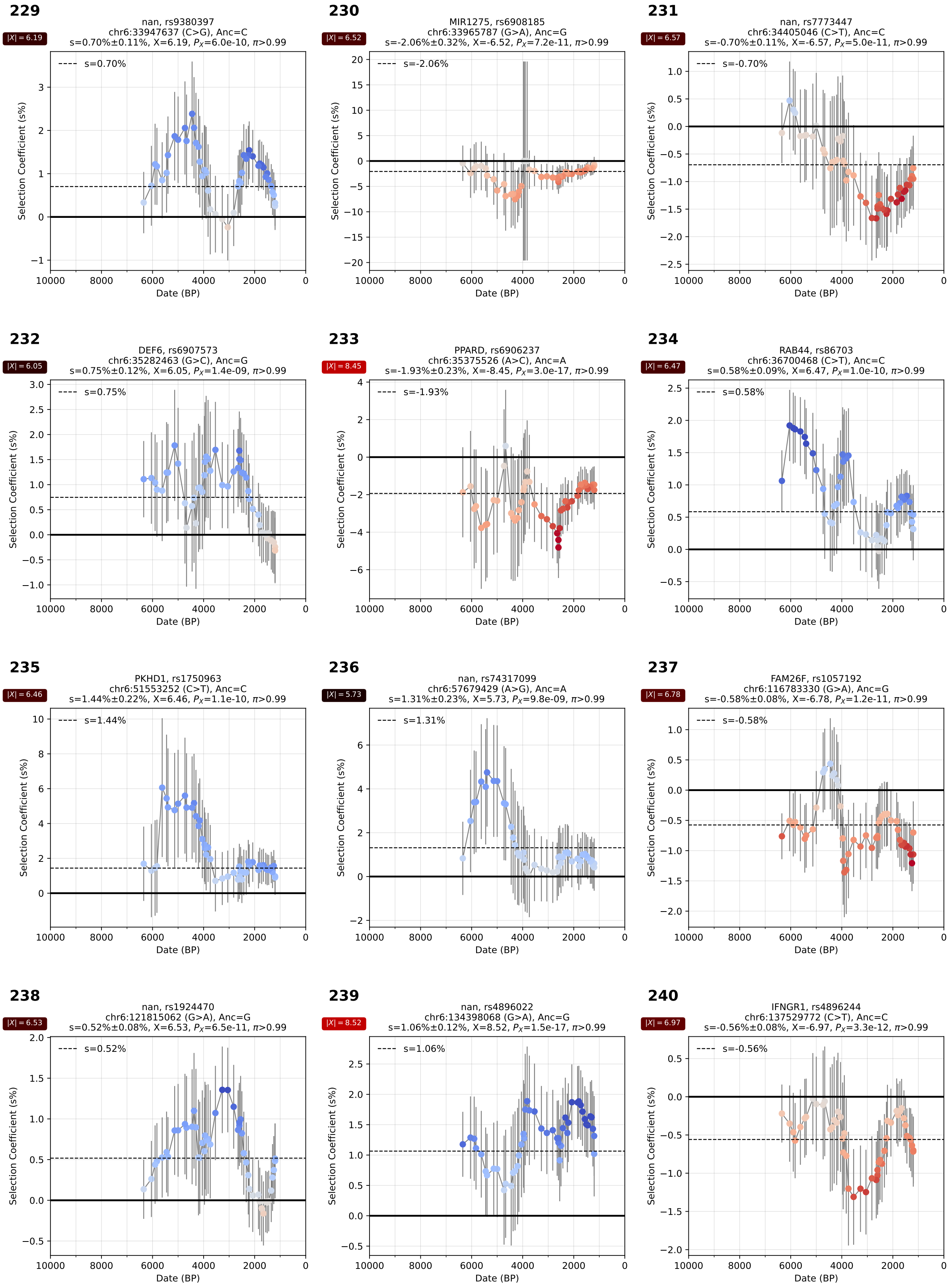

Supplementary Figure S5.60: Selection coefficient over time.

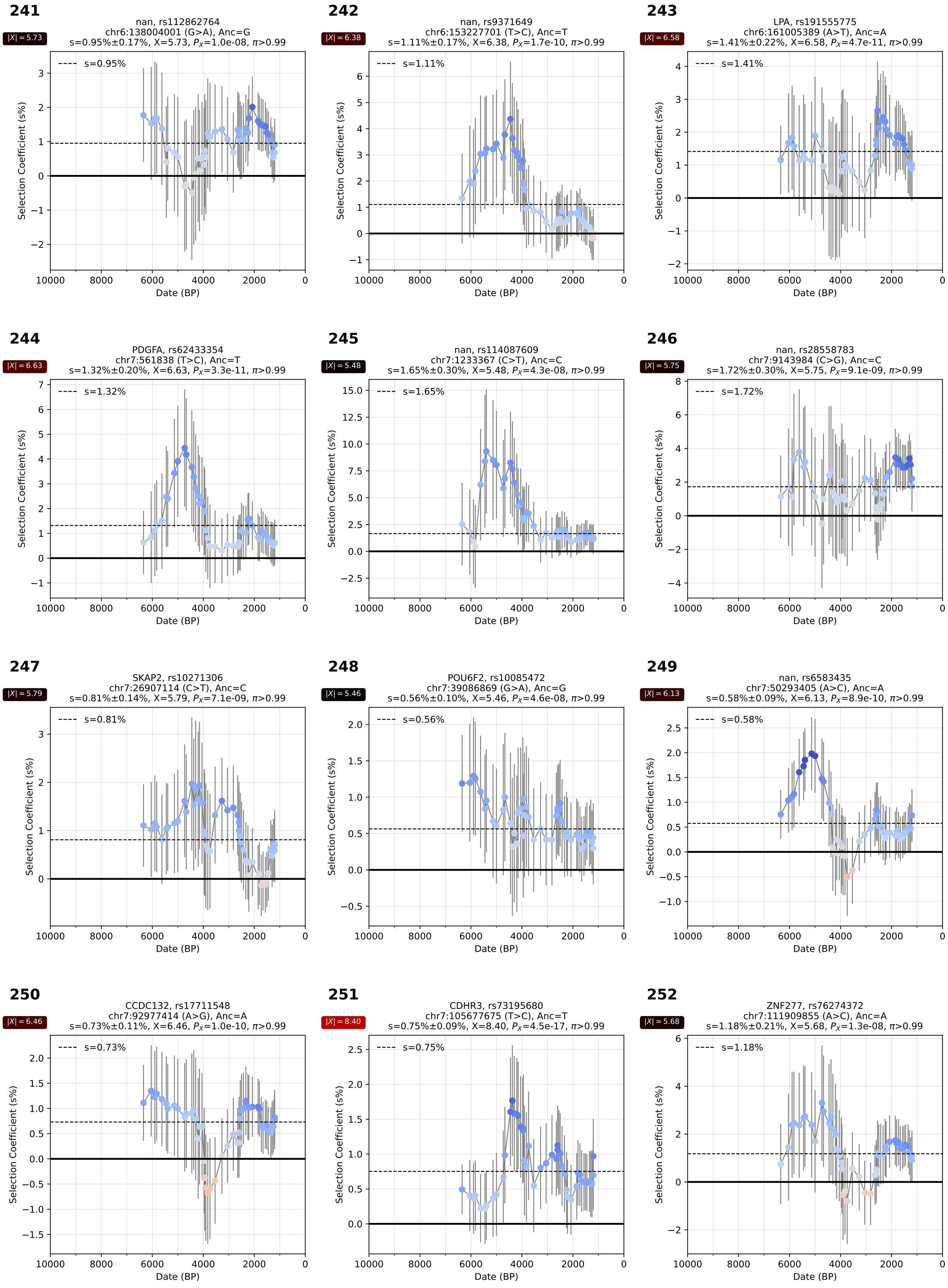

Supplementary Figure S5.61: Selection coefficient over time.

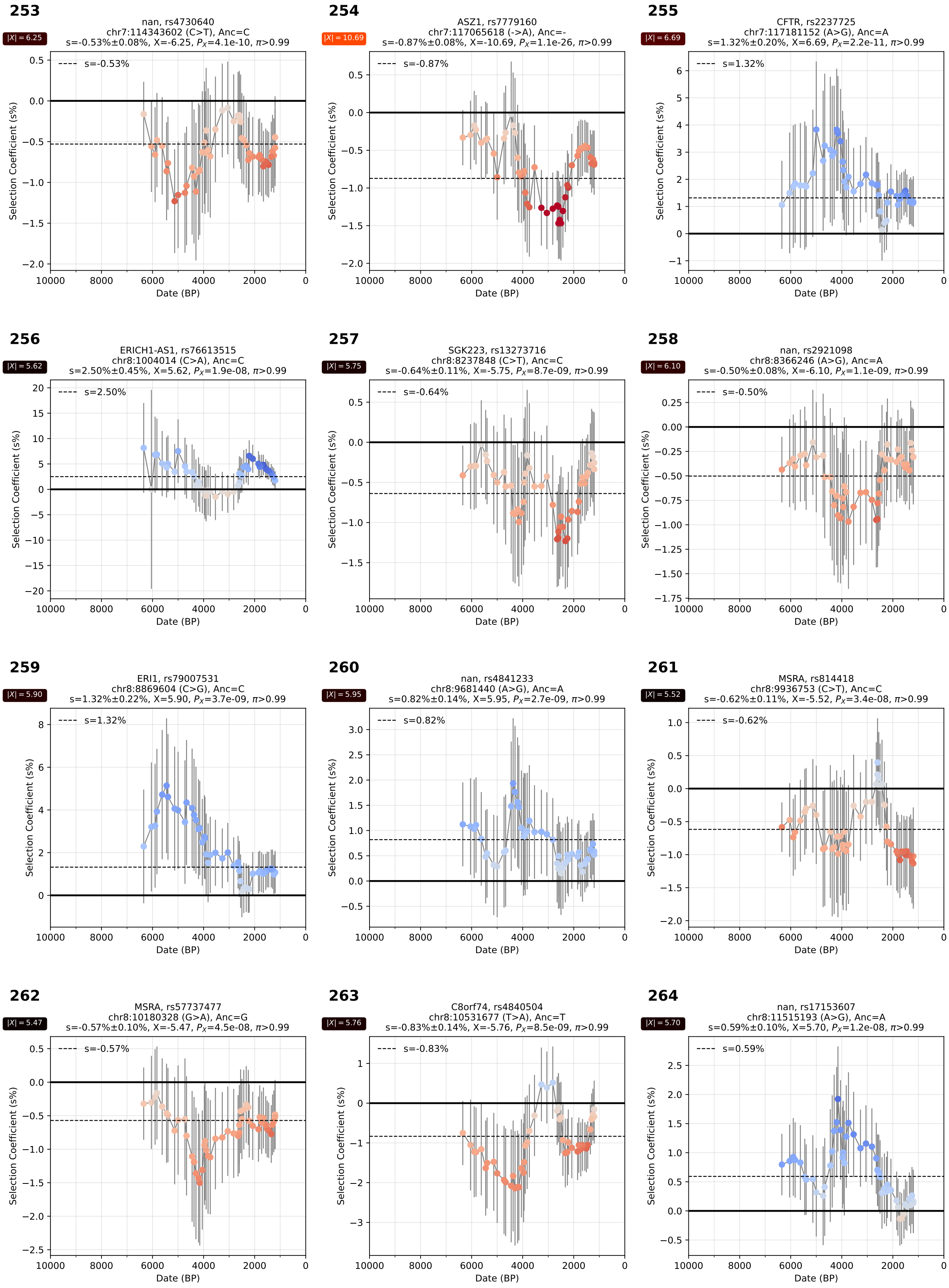

Supplementary Figure S5.62: Selection coefficient over time.

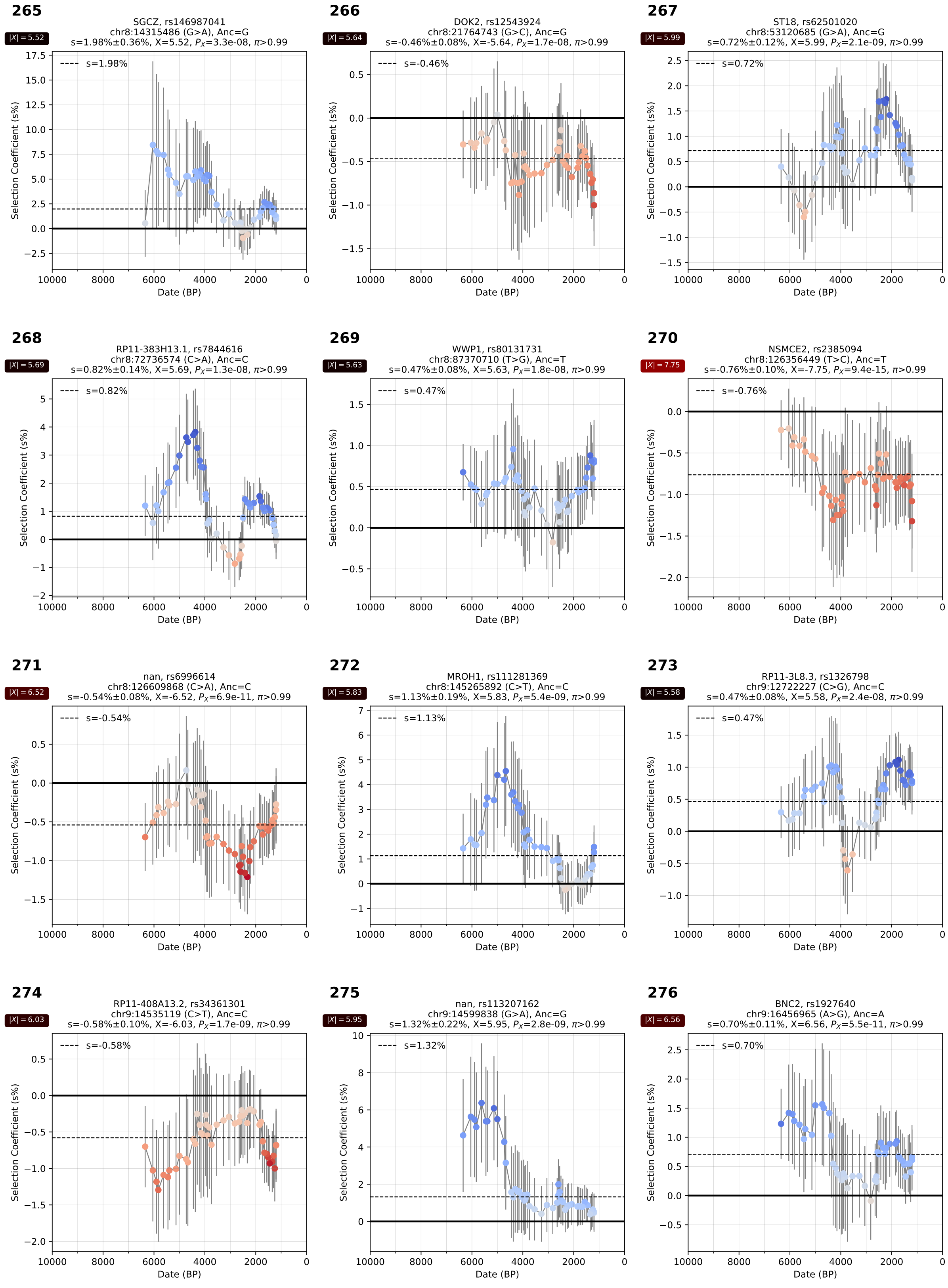

Supplementary Figure S5.63: Selection coefficient over time.

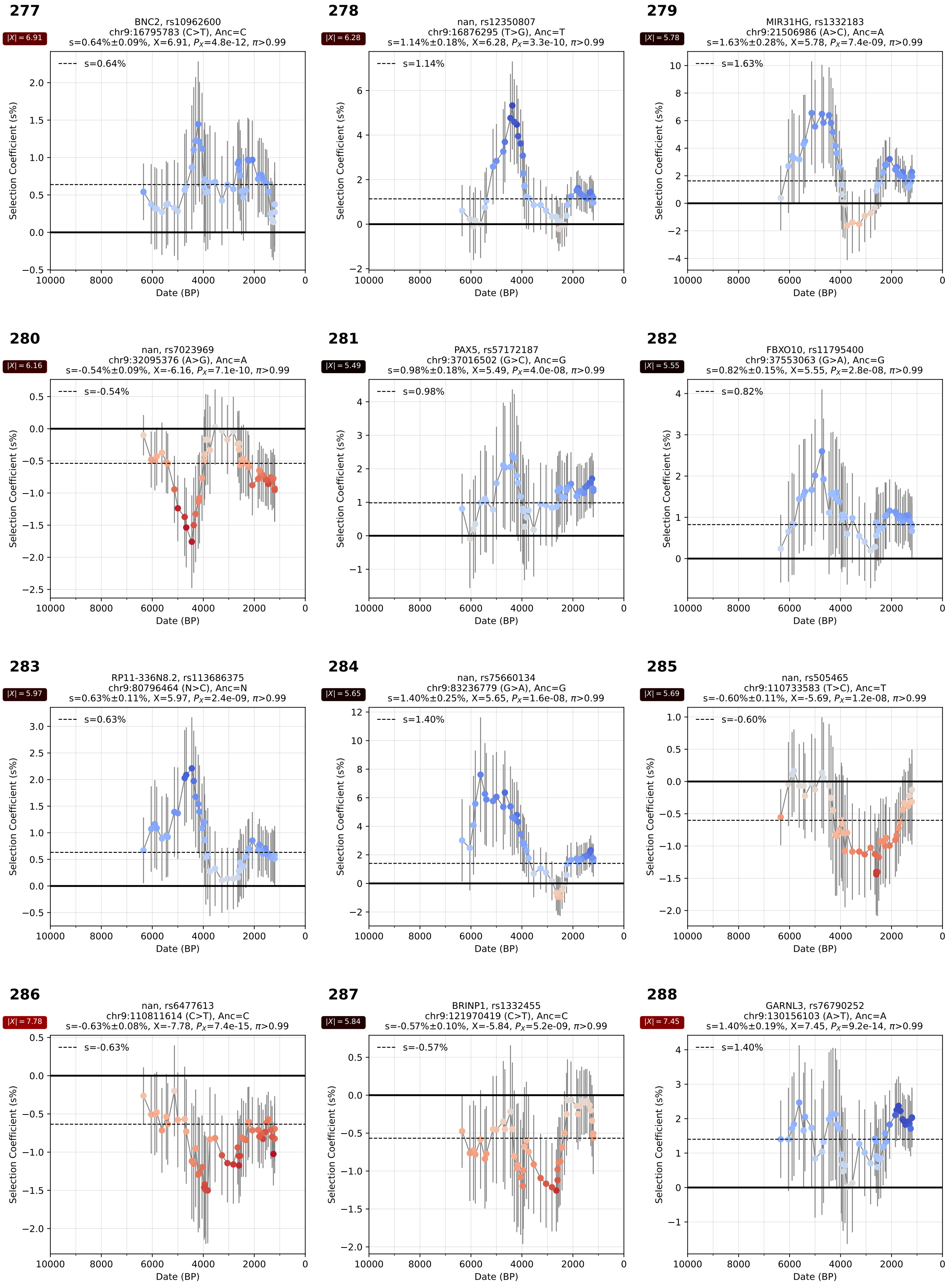

Supplementary Figure S5.64: Selection coefficient over time.

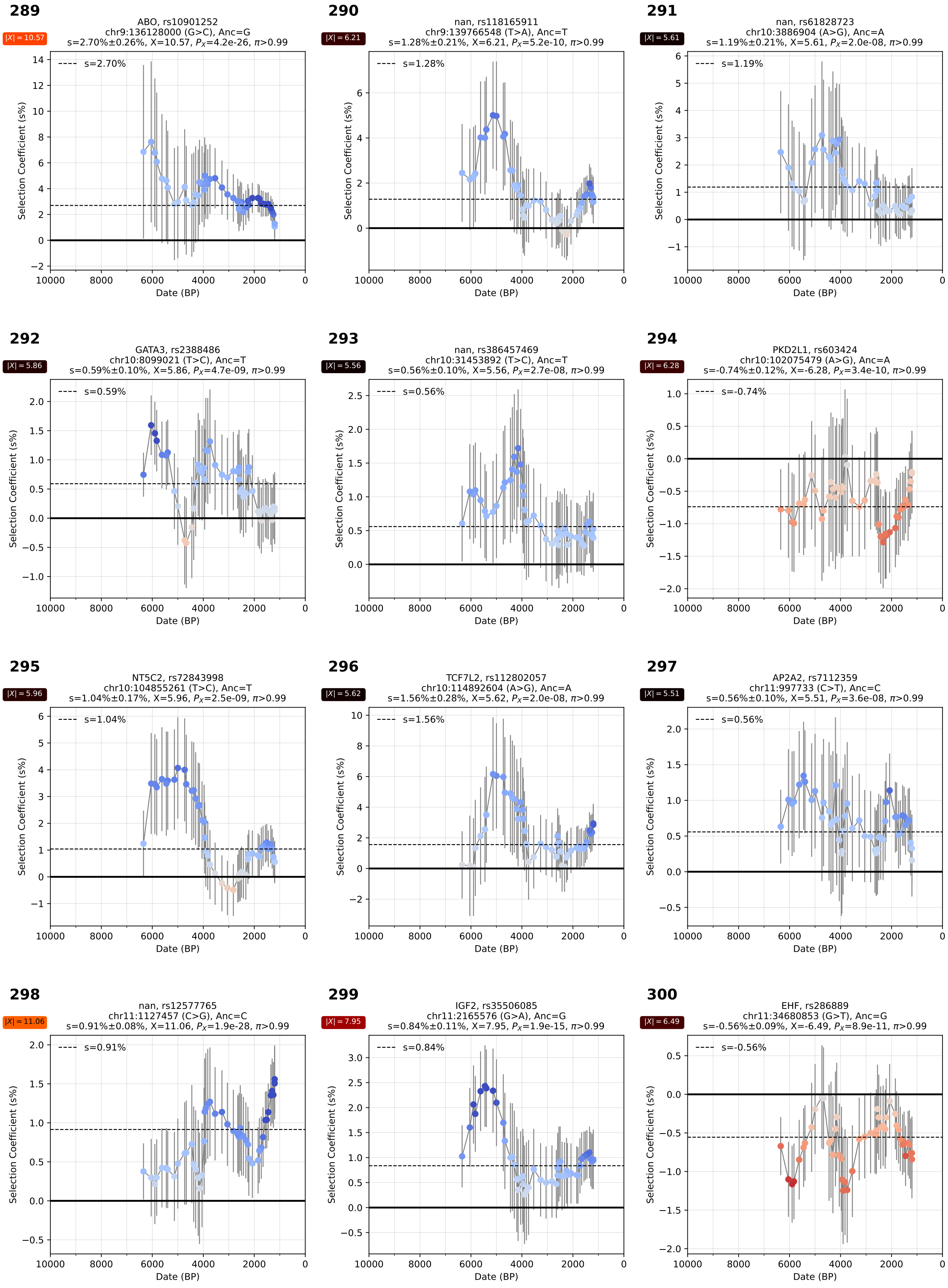

Supplementary Figure S5.65: Selection coefficient over time.

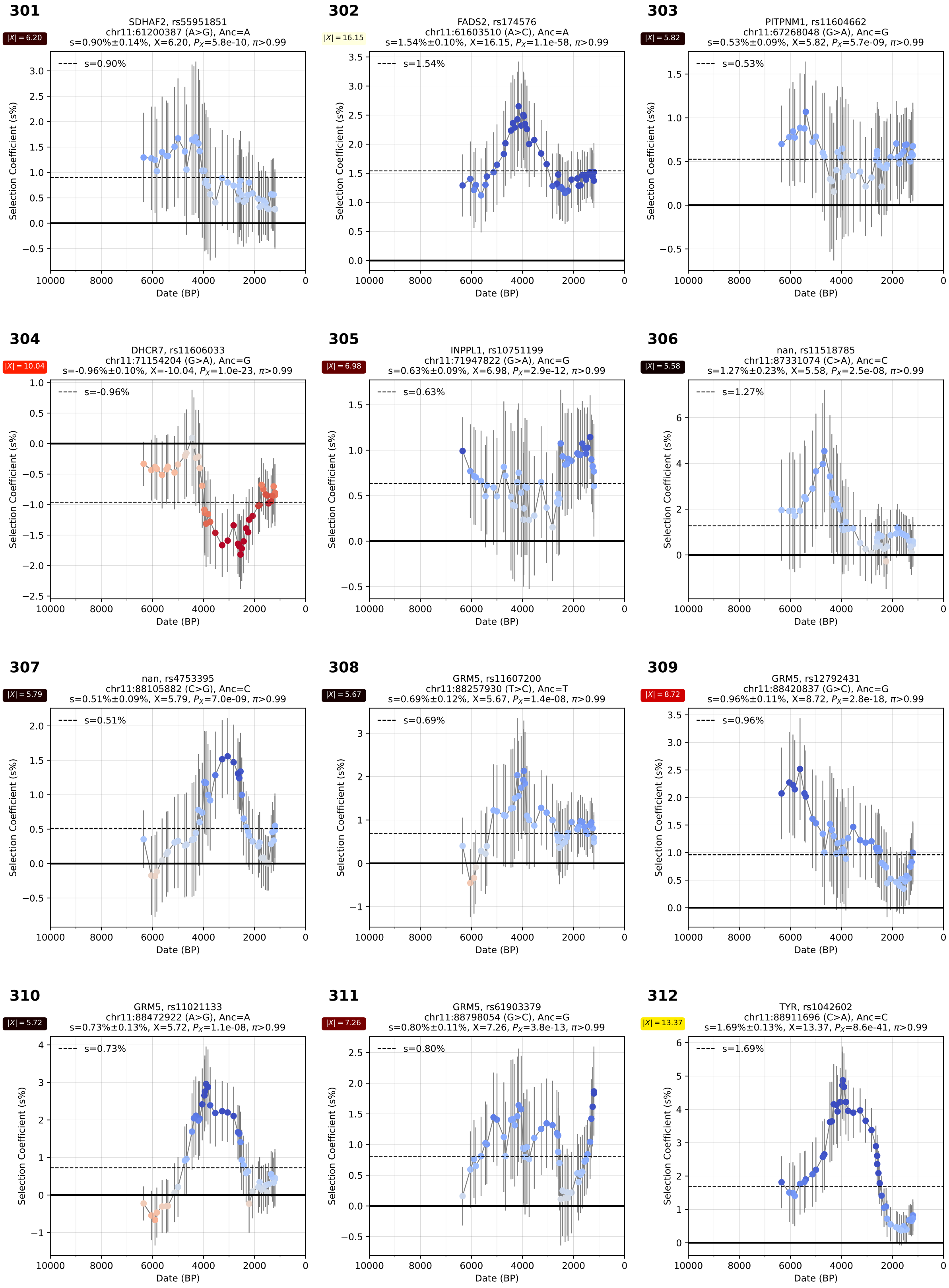

Supplementary Figure S5.66: Selection coefficient over time.

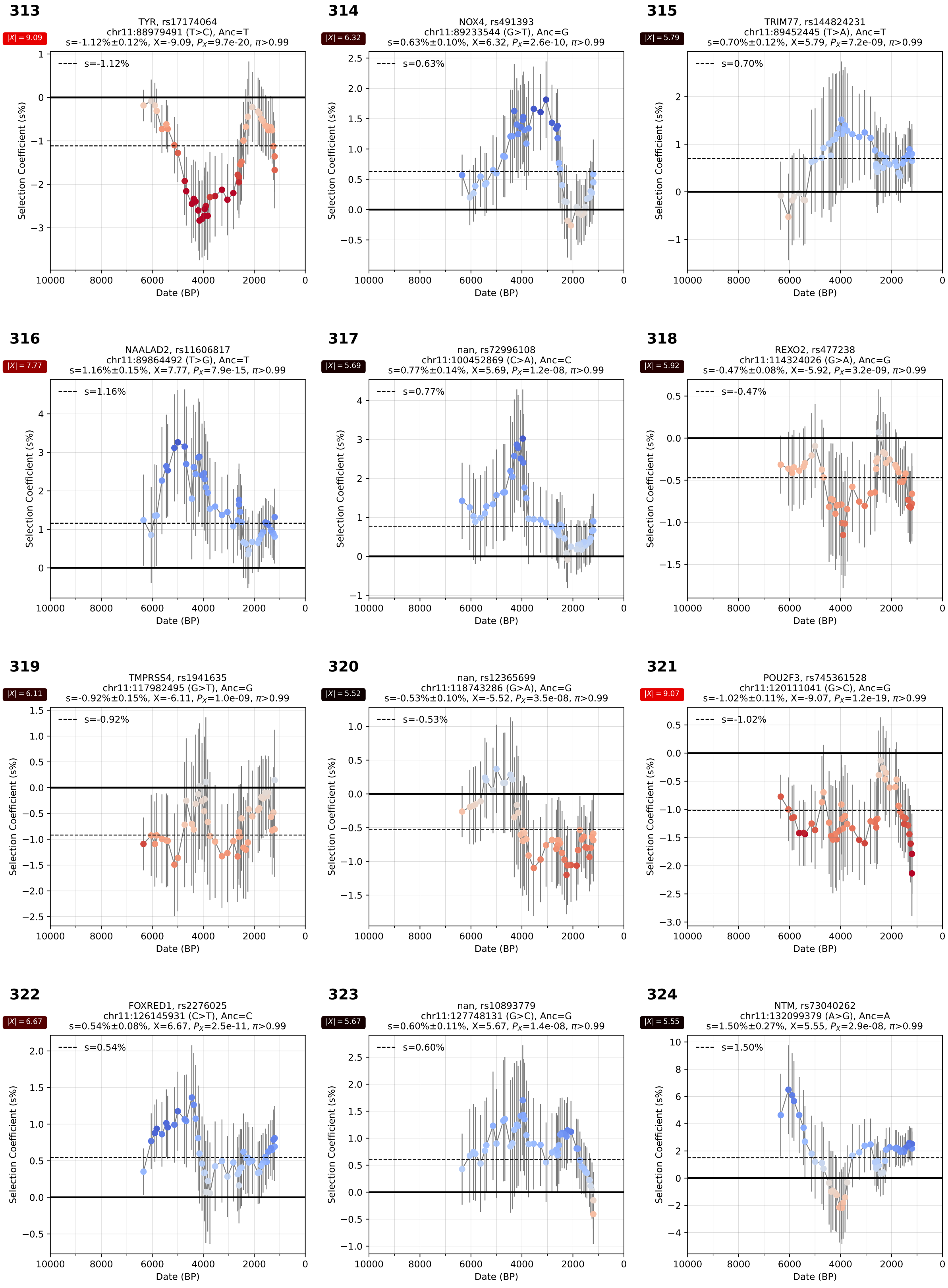

Supplementary Figure S5.67: Selection coefficient over time.

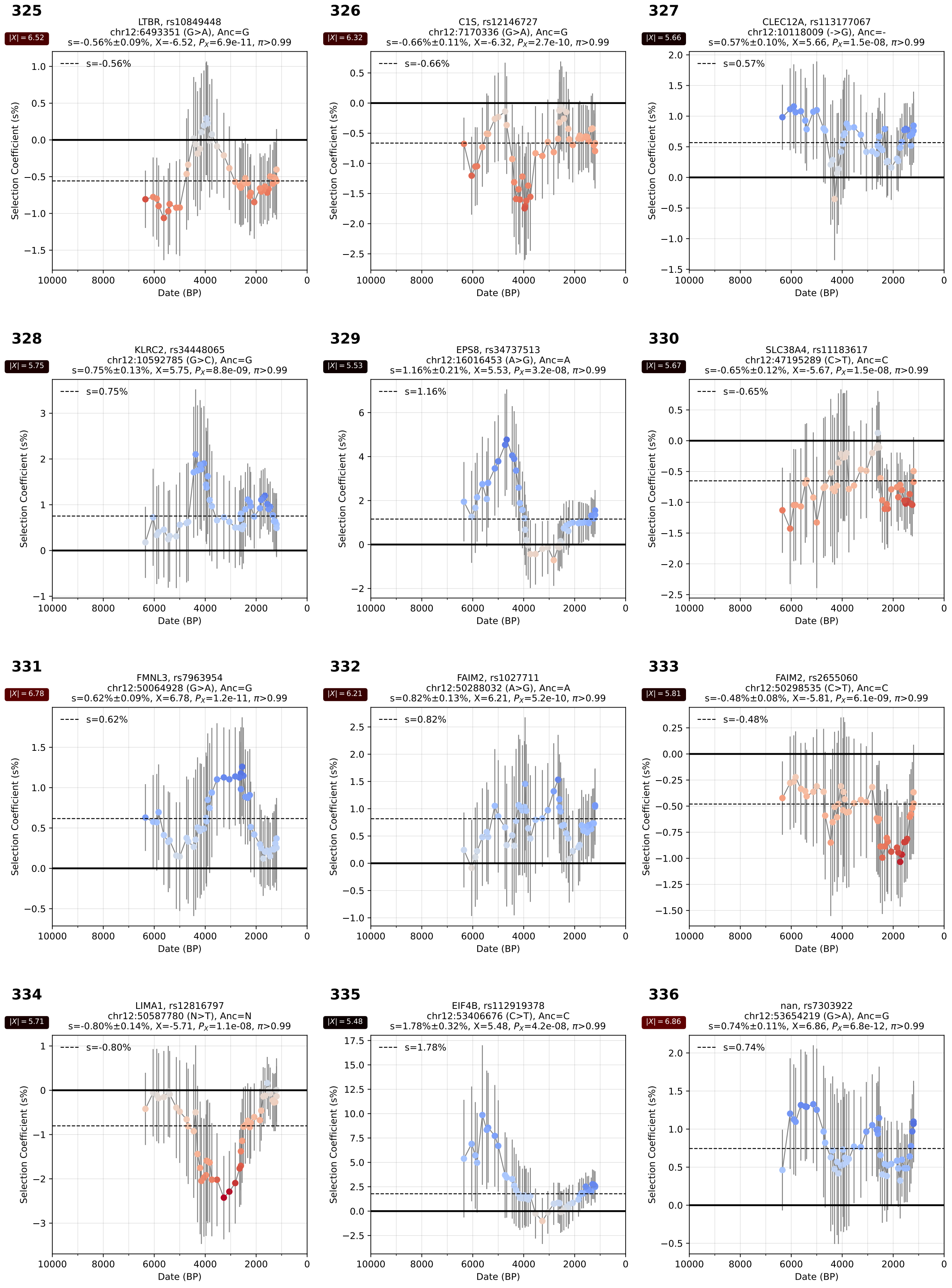

Supplementary Figure S5.68: Selection coefficient over time.

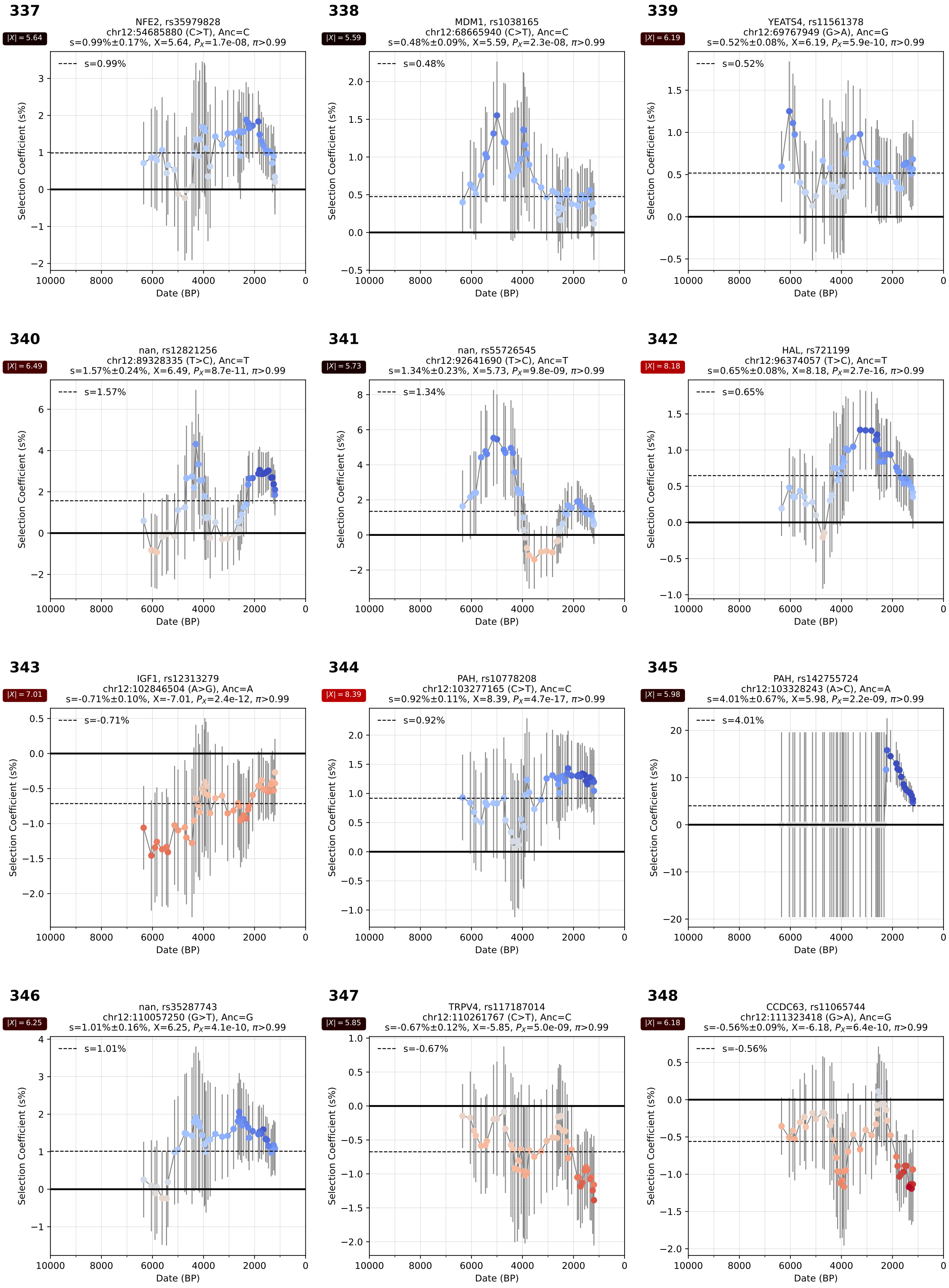

Supplementary Figure S5.69: Selection coefficient over time.

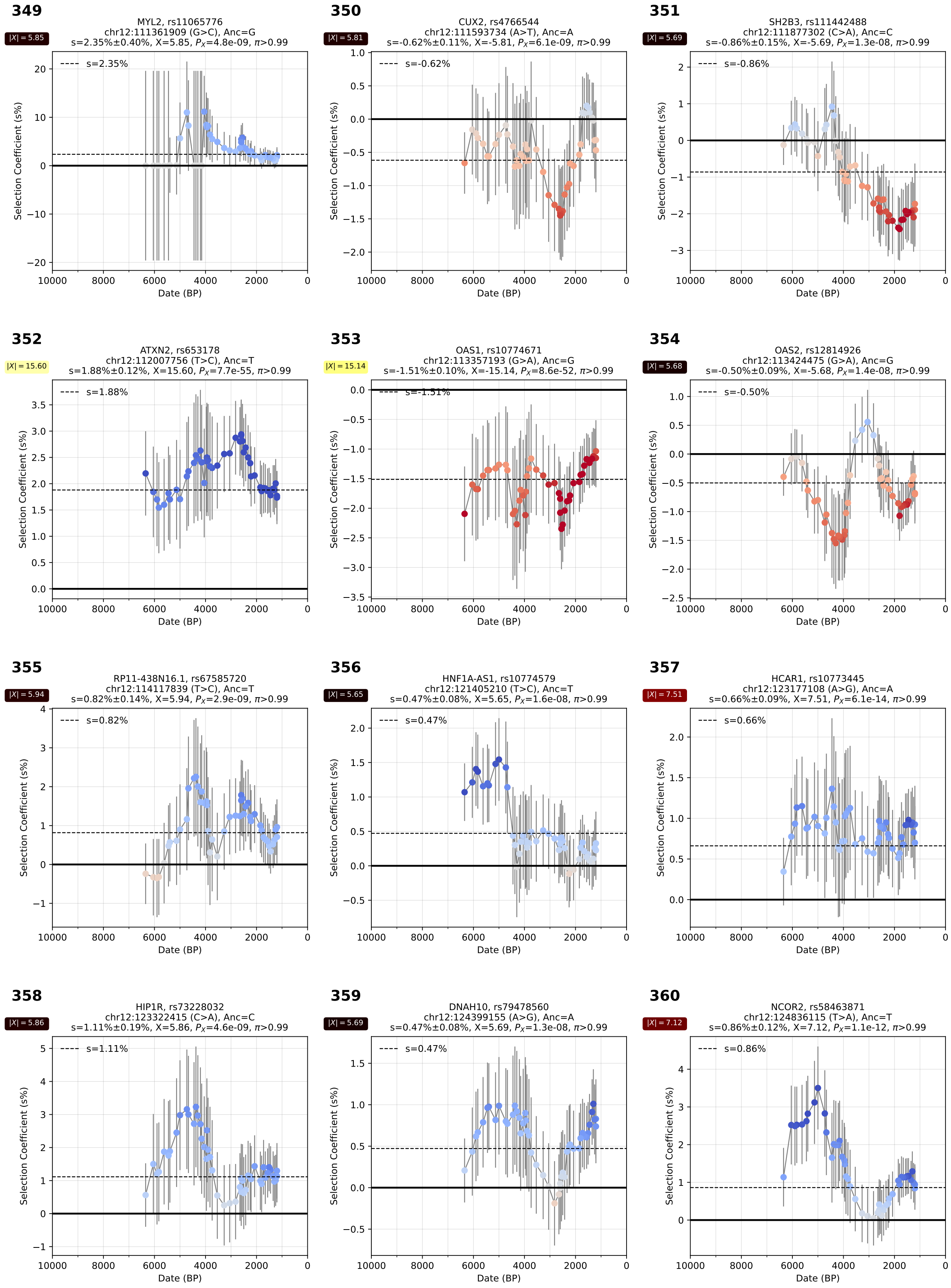

Supplementary Figure S5.70: Selection coefficient over time.

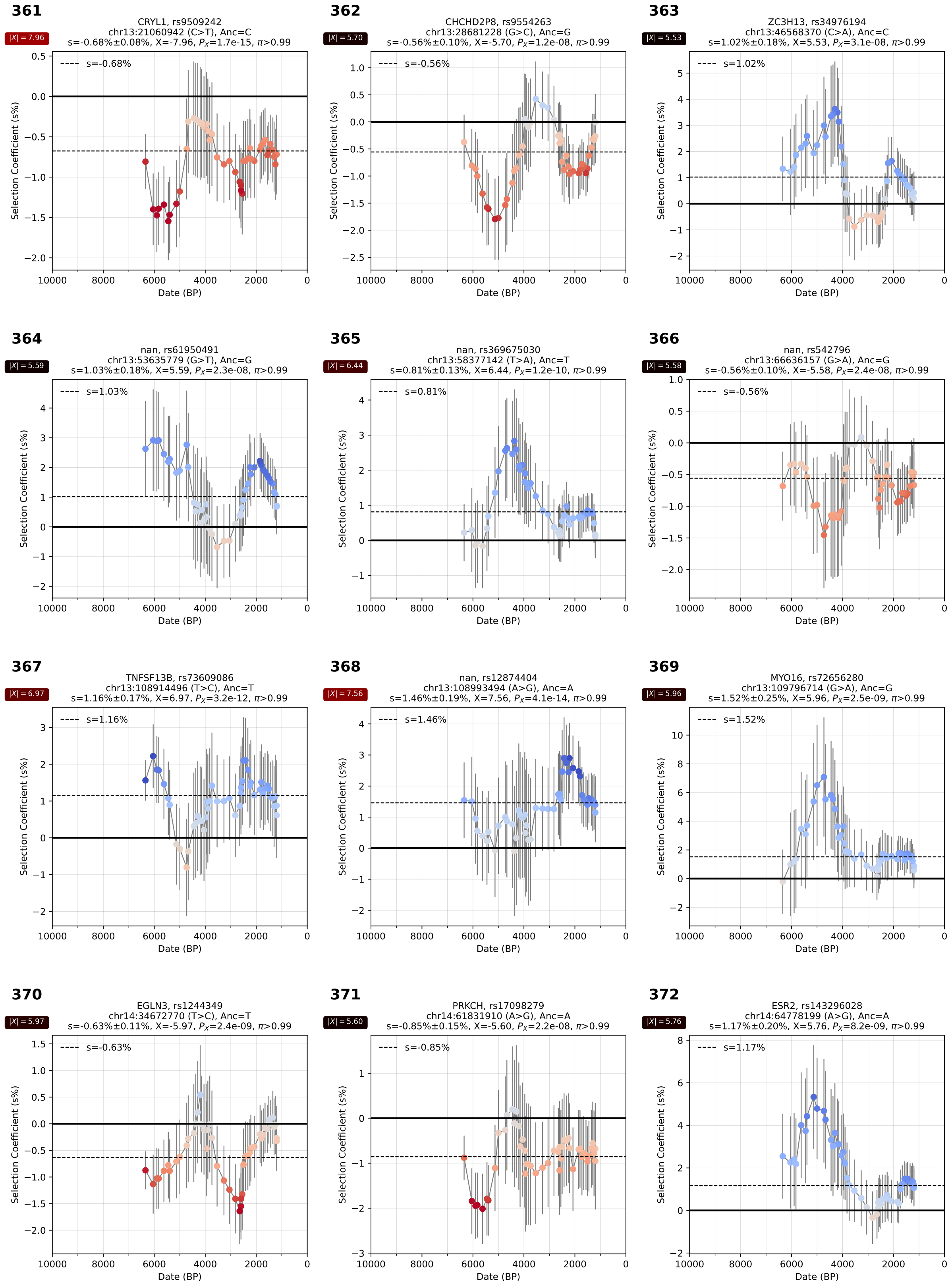

Supplementary Figure S5.71: Selection coefficient over time.

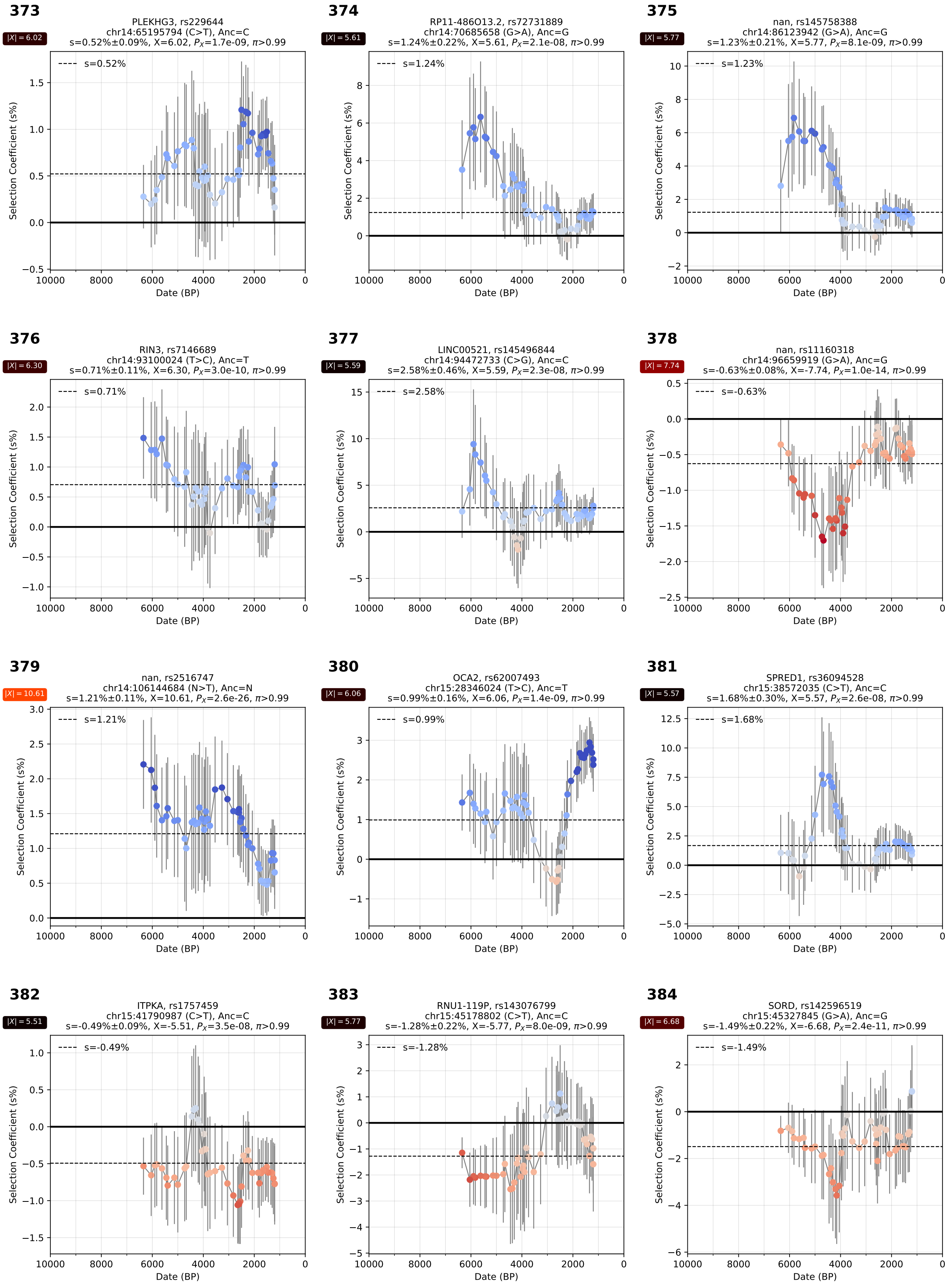

Supplementary Figure S5.72: Selection coefficient over time.

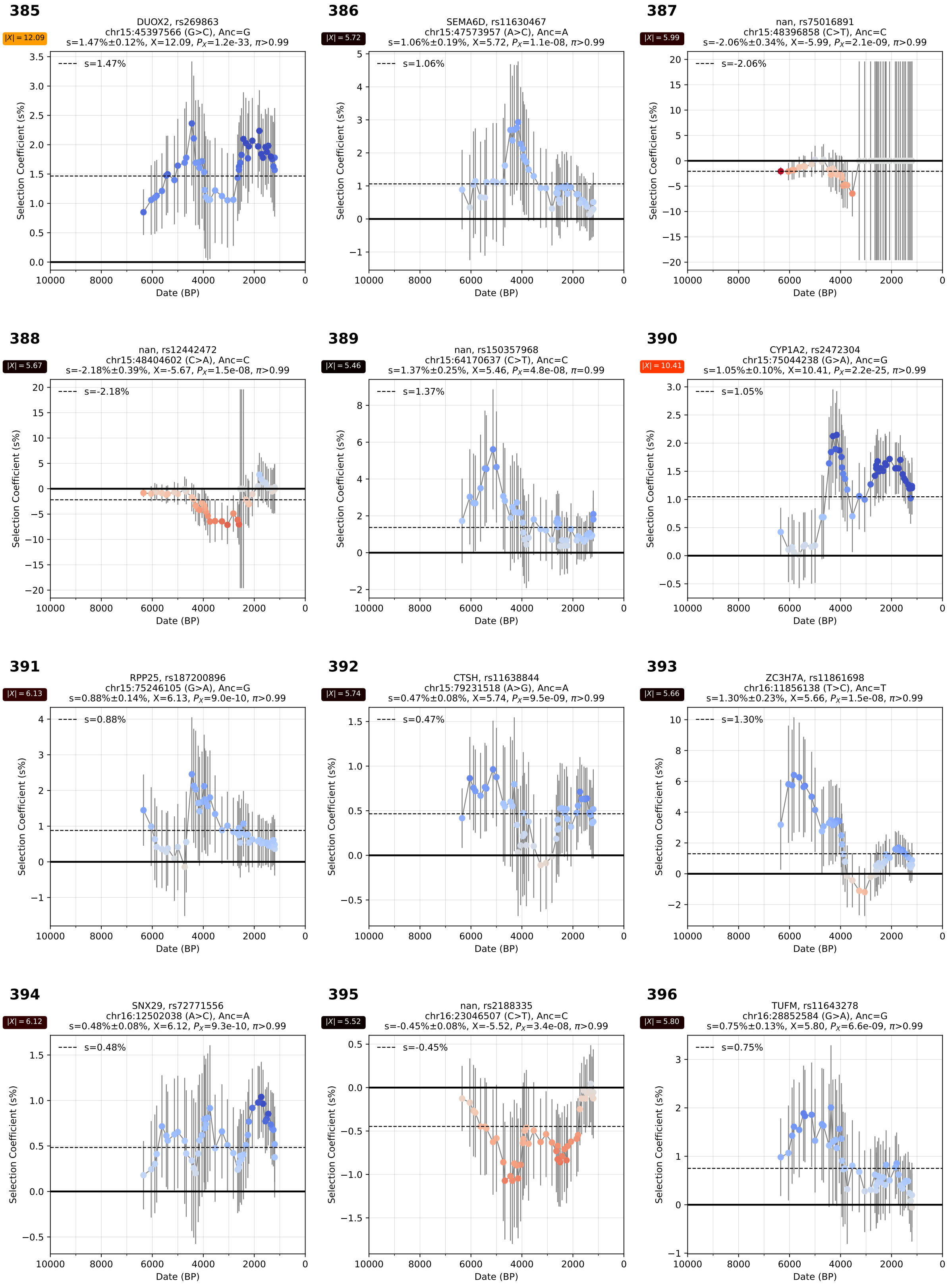

Supplementary Figure S5.73: Selection coefficient over time.

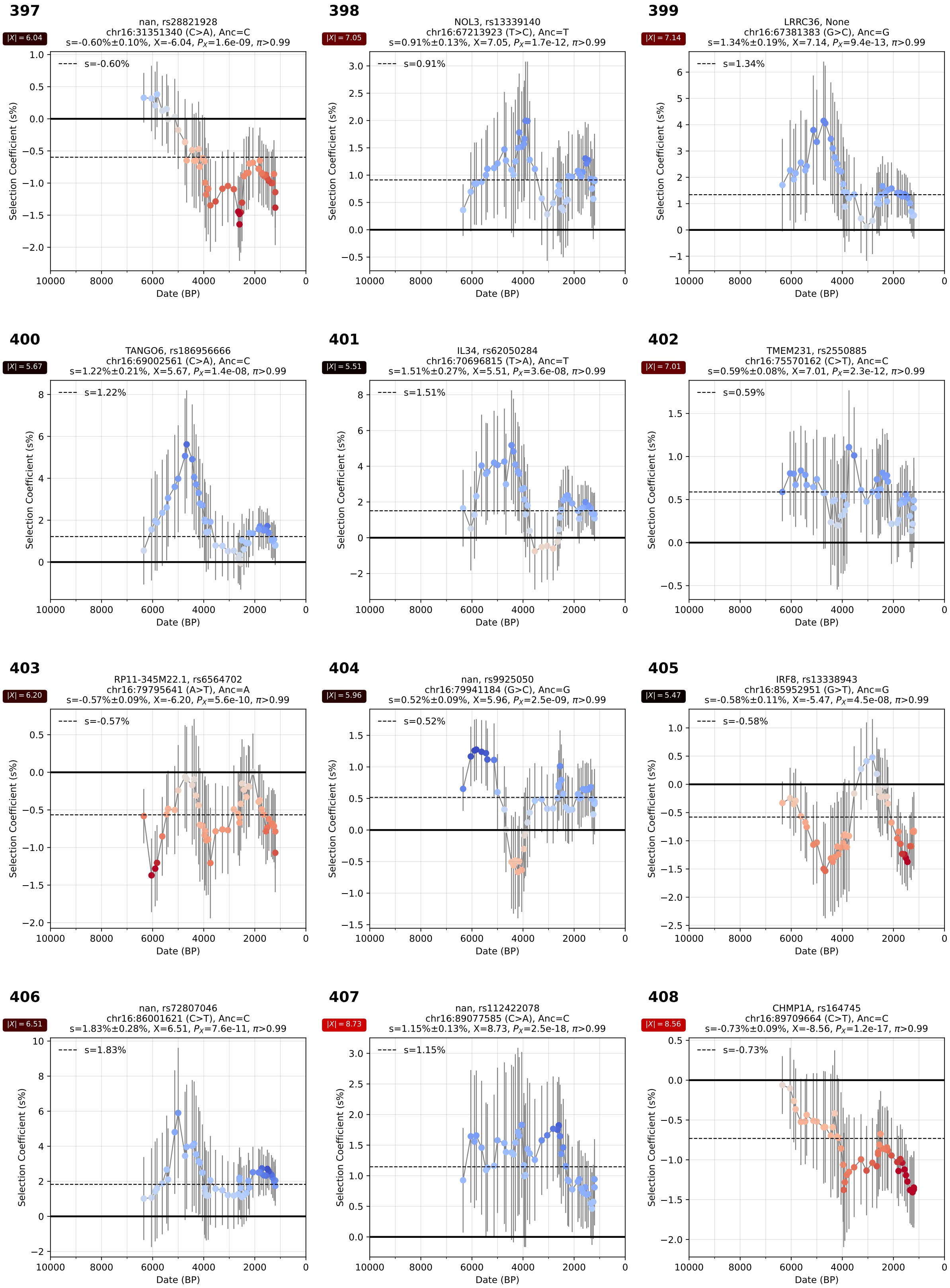

Supplementary Figure S5.74: Selection coefficient over time.

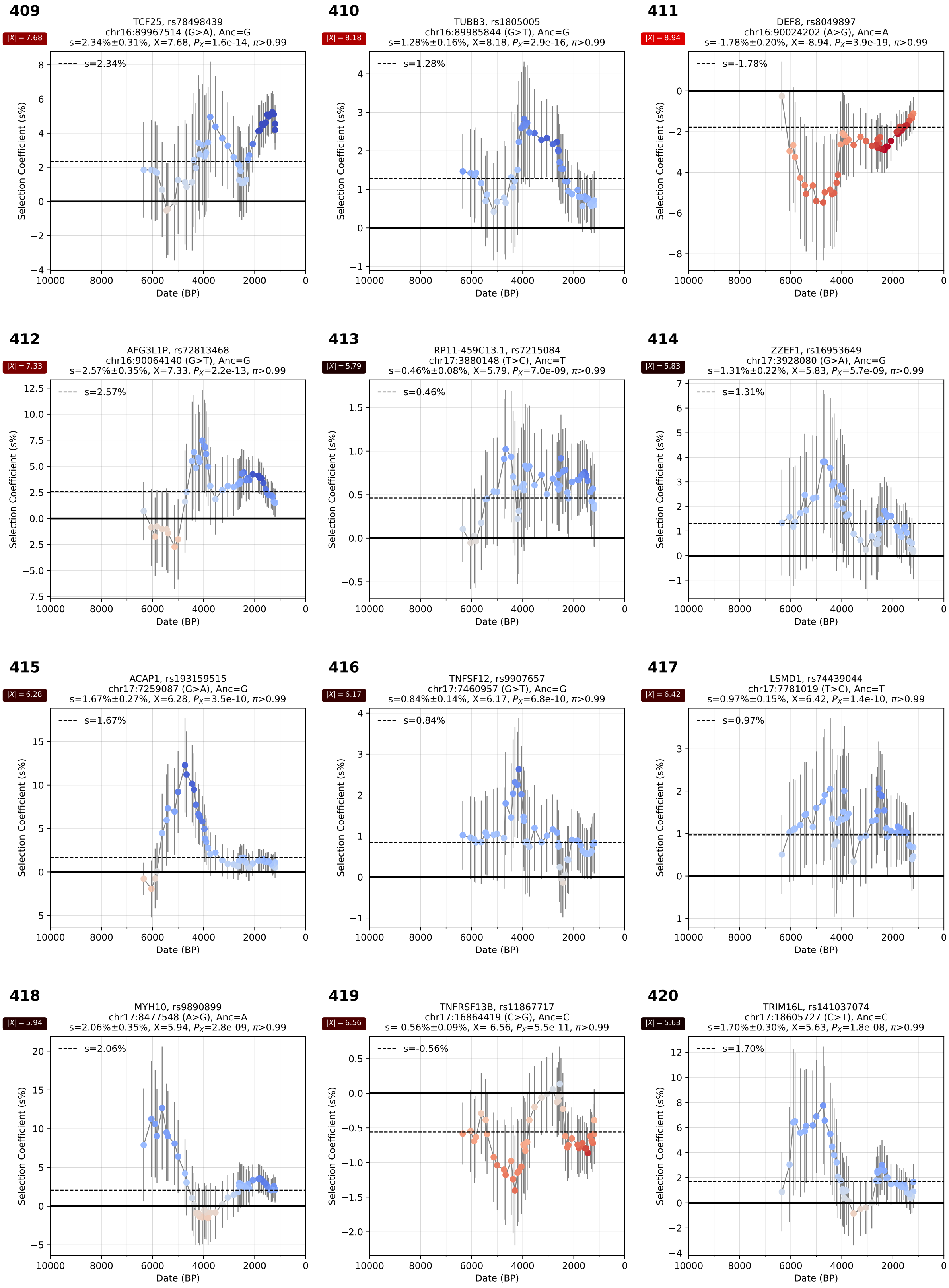

Supplementary Figure S5.75: Selection coefficient over time.

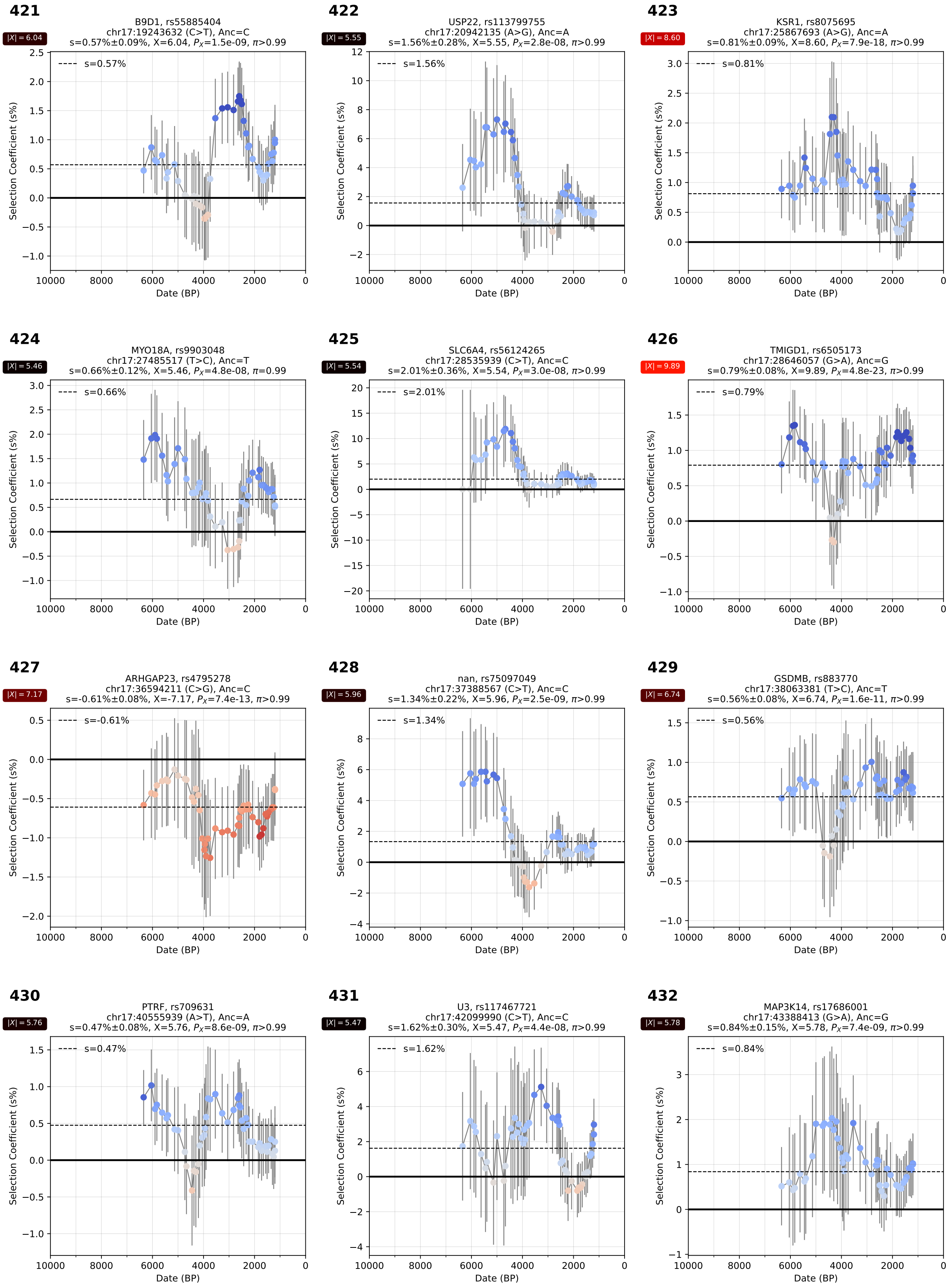

Supplementary Figure S5.76: Selection coefficient over time.

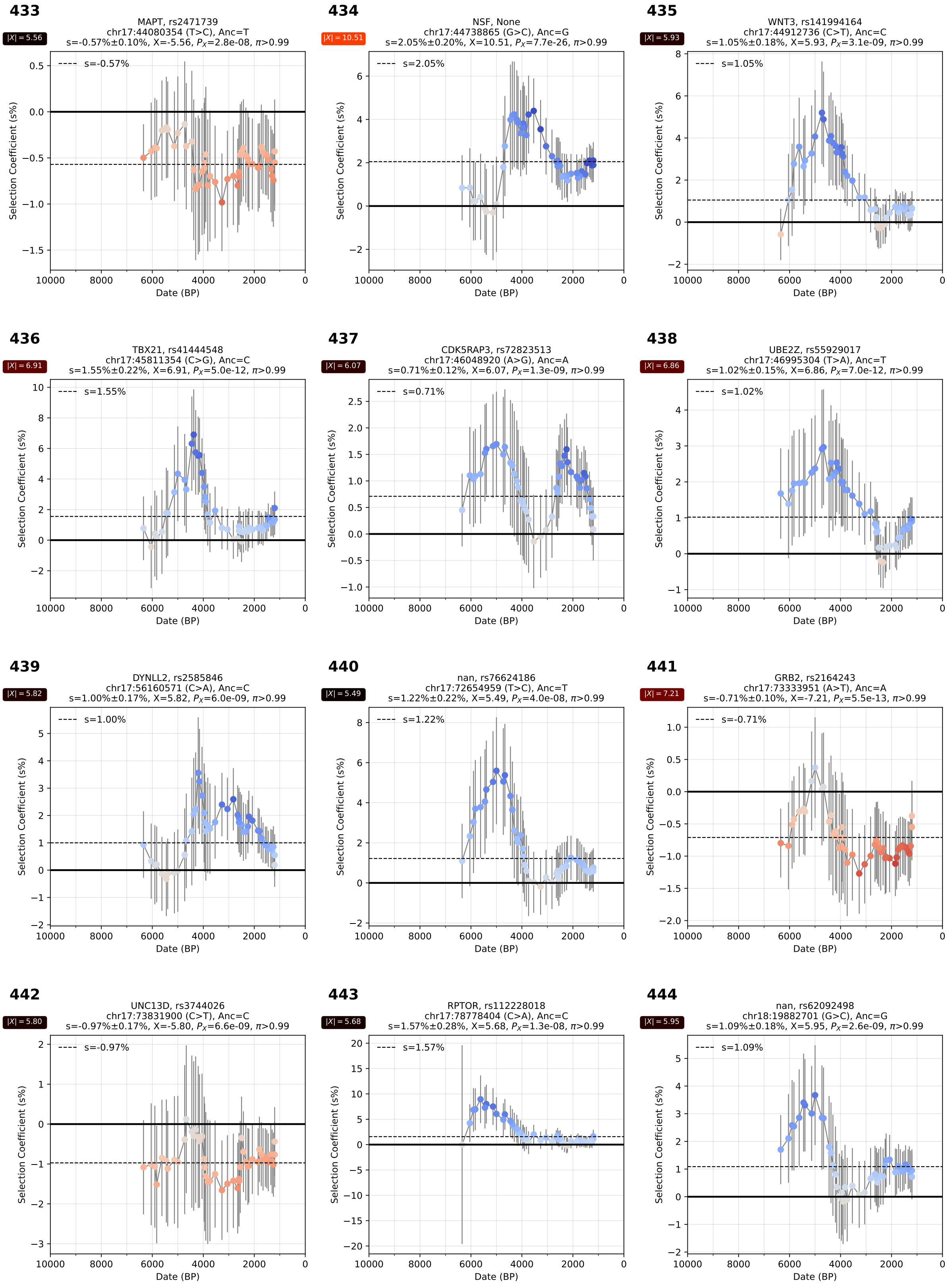

Supplementary Figure S5.77: Selection coefficient over time.

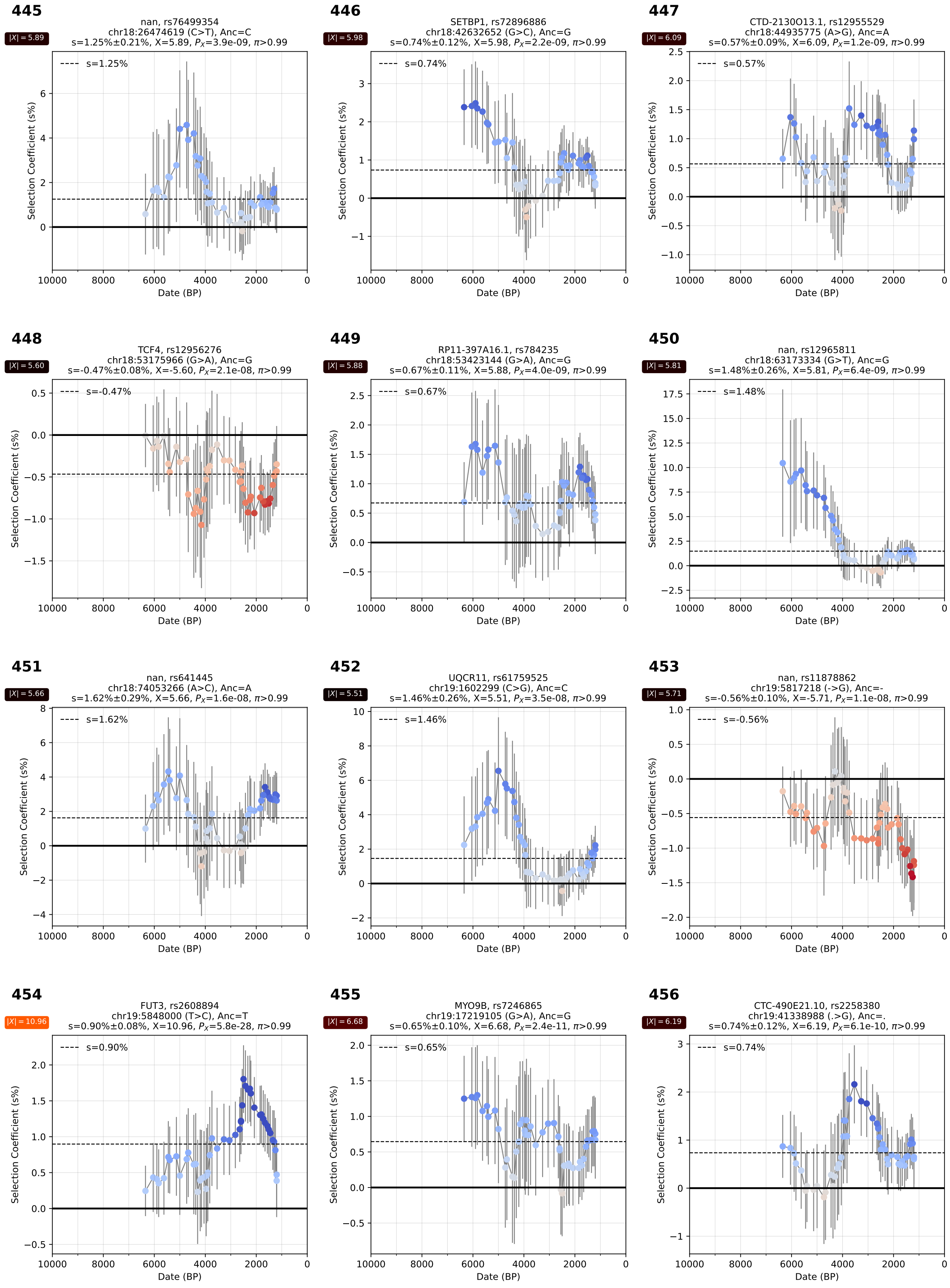

Supplementary Figure S5.78: Selection coefficient over time.

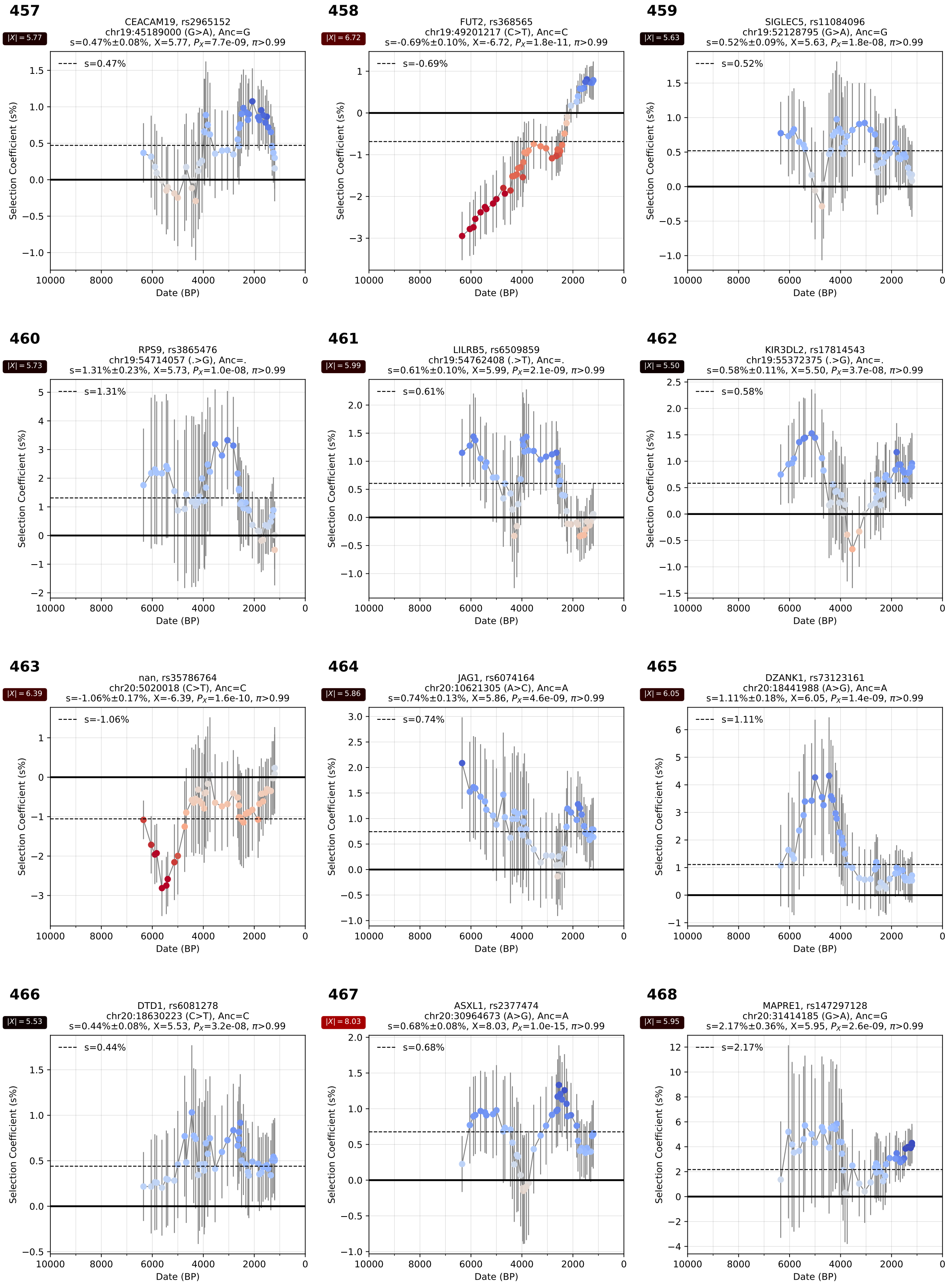

Supplementary Figure S5.79: Selection coefficient over time.

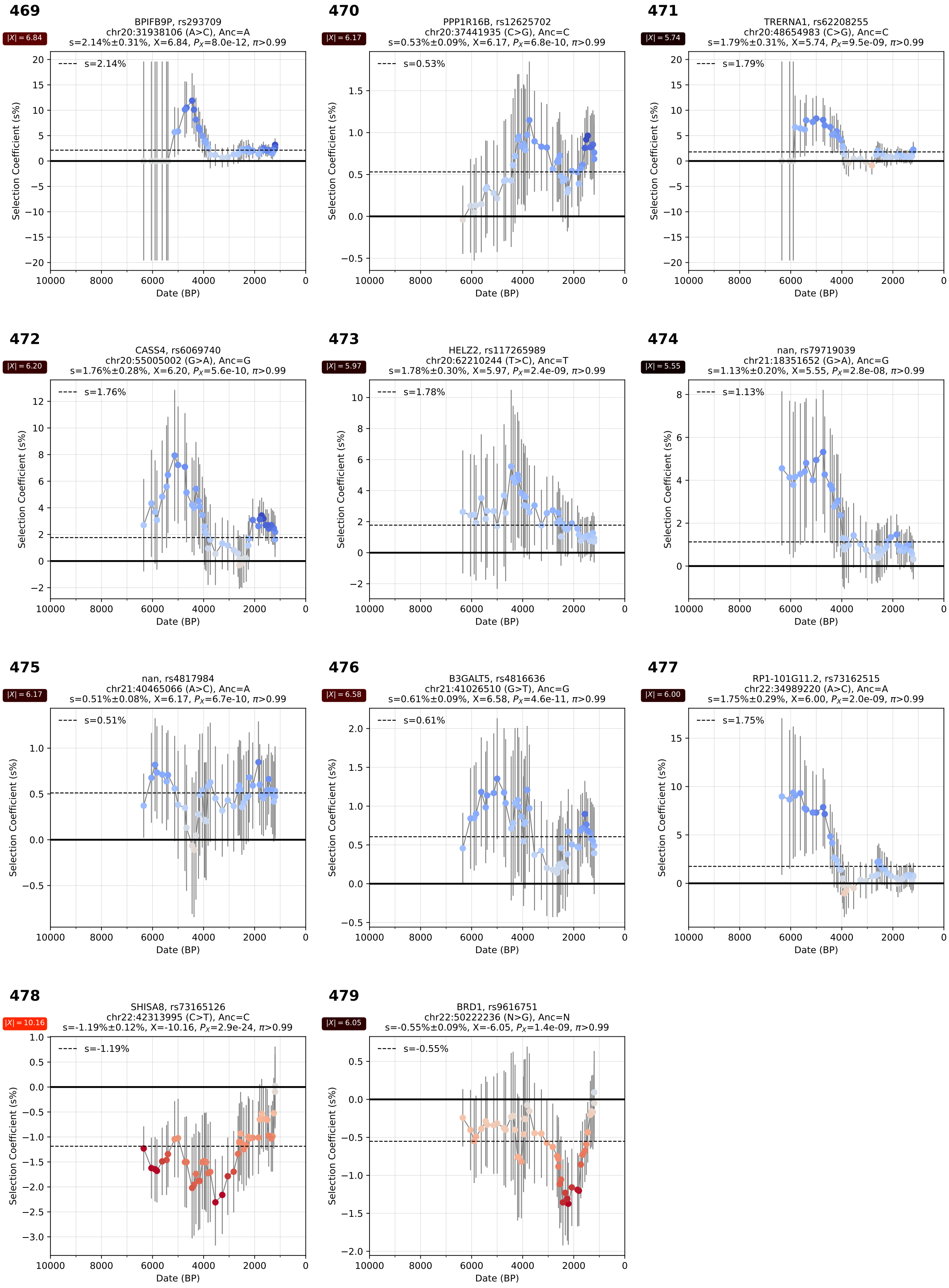

Supplementary Figure S5.80: Selection coefficient over time.

Supplementary Information section 6
Re-evaluation of results from previous studies

Overview
We evaluated candidate loci from five different genome scans for selection: four analyzing ancient DNA time transects (Mathieson et al. 2015[39], Le et al. 2022[40], Kerner et al. 2023[41], and Irving-Pease et al. 2024[7]) and one analyzing modern variation but sensitive to signals we might expect to be replicable in our ancient DNA time transect (Field et al. 2016[42]).

To enable this comparison, we used variants from the high-coverage (30x) 1000 Genomes Project[43] mapped to GRCh38, and remapped to positions in GRCh37/hg19 using CrossMap (v0.5.2)[44]. We used data from 2,504 unrelated individuals from the phase three panel of the 1000 Genomes Project as our imputation reference panel. We retained only the variants that passed all quality control filters from gnomAD (v2.1.1)[45], indicated by a PASS value in the FILTER column of the VCF file available for all chromosome sites on the gnomAD website. This filtering resulted in 52,382,872 biallelic variants. In some cases, the SNPs reported by five studies whose results we re-examined were not included in this reference panel and were excluded from our re-analysis. Additionally, some SNPs did not pass subsequent quality control (QC), as detailed in Supplementary Information section 1. We analyzed all SNPs present in the reference panel. However, the GLMM and allele frequency trajectory results for SNPs that failed QC may be unreliable and could be influenced by the artifacts that caused QC failure.

For each study, we re-evaluated the selection signals using our GLMM approach. The cumulative number of non-HLA signals identified as genome-wide significant in these studies and confirmed in our re-analysis with a posterior probability of π>99% is 18 (4% of the 410 non-HLA loci showing π>99% in our genome-wide scan). Of these, 8 were found in Mathieson et al., Field et al. added 0, Le et al. added 3, Kerner et al. added 0, and Irving-Pease et al. added 7. (Extended Data Table 1, Table S6.1). An additional 20 non-HLA loci reported as genome-wide significant in at least one of these five studies did not replicate at π>99% in our re-analysis (Extended Data Table 1).

Mathieson et al. 2015 analyzed whole genome data from 230 ancient Eurasian individuals who lived between 6500 BCE and 300 BCE and compared to data from modern Europeans from the 1000 Genomes Project. They found 12 genome-wide signals of selection with a significance exceeding a P-value threshold of 5e-8, two of which are from the HLA region. Of the 11 that pass our QC, we replicate 10 (π>99%) (the 11th is replicated at π=32%, and is almost certainly a real signal of selection as it is the blue eye-color variant at *OCA2*/*HERC2* which has been found to be subject to fluctuating selection which our methodology is not optimized to detect as we are explicitly testing for a scenario of a constant non-zero selection coefficient).

Field et al. 2016 analyzed 3195 contemporary individuals from the UK10K project to study signals of selection in the past 2000 years inferred from an unusually high density of singleton genetic variants associated with a tested allele, which can be evidence of a distortion of the gene as a result of selection. We applied our GLMM approach to three time transects: to all individuals in our

study, to individuals who lived before 2000 BP, and to individuals who lived after 2000 BP which is the time frame where Field et al. 2016 have particularly notable statistical power. There are 3 genome-wide signals of selection in Field et al. 2016 exceeding a P value threshold of 5e-8, and one of them (*LCT*) is replicated in our analysis (π>99%). The second locus, *HLA*, has a posterior probability of π=92% in our evaluation. There is no evidence for selection during the past 2000 years for the third candidate at the *WDFY4* locus using our analysis, and we hypothesize that this signal may be an artifact of incompletely corrected population structure due to ancestry derived from steppe pastoralists, an issue known to have caused Field et al. 2016 to find false-positive signals of polygenic selection[46,47]. Using a less stringent threshold, out of 37 independent loci highlighted in Field et al. 2016 as passing the significance threshold of 1e-5, 35 pass our QC, and only three (*LCT*, *KITLG, BNC2*) show a strong signal of selection (π>99%).

Le et al. 2022 analyzed 1291 European individuals from the past 10,000 years to identify selection signals in the Neolithic, Bronze Age, and Historical periods. They found 25 selection signals across 24 loci, of which 22 loci could be retested in our analysis (two were not present in the imputation reference panel), and we replicated 9 in our full time transect (π>99%). We also separately analyzed each of the three epochs to mimic the approach in Le et al. 2022, and only replicated 7 of 23 signals for which the associated SNPs passed QC. In the Neolithic we replicated 1 out of 10. In the Bronze Age we replicated 1 out of 7 (we could not retest two SNPs that were absent in the imputation reference panel). In the Historical period we replicated 5 out of 6.

Kerner et al. 2023 analyzed 2879 Eurasian individuals from the past 10,000 years to screen for signals of selection. They highlighted a list of 139 SNPs, with 89 potentially showing evidence of positive selection and 50 showing evidence of negative selection on the derived allele. As the authors point out in the transparent peer review records published alongside their study (Supplementary Document S2 of that study), only 3 of these 139 signals of selection (*LCT*/*MCM6*, HLA, and *SLC45A2*) pass a formal genome-wide significant threshold with a P-value significance threshold of 5e-8; all three are replicated in our analysis. We believe that the great majority of the 139 highlighted selection signals are false positives due to not applying a threshold for genome-wide statistical significance: out of 125 candidate SNPs that pass QC, only 14 are genome-wide significant in our re-analysis (π>99%); an additional 9 loci had probable evidence of selection in our re-analysis (50%<π<99%). When we visually inspect the SNPs where there is a failure to replicate, the great majority show no visual evidence for selection in our time-transect. In particular, shifts in frequency at these non-replicated variants often appear to be in the direction expected based on pre-existing allele frequency differences between source populations like steppe pastoralists, European farmers, and European hunter-gatherers (which would be expected to produce a substantial allele frequency shift following mixture of these group if not corrected).

Irving-Pease et al. 2024 analyzed 1518 West Eurasians over the past 15,000 years and used the CLUES methodology[48,49] to identify 21 genome-wide significant loci with at least one genome-wide significant signal (P-value threshold of 5e-8) across five ancestry categories: pan-ancestry (ALL), Western hunter-gatherers (WHG), Eastern hunter-gatherers (EHG), Caucasus hunter-gatherers (CHG), and Anatolian farmers (ANA). Of the 21 candidate loci identified, our analysis found that 14 had at least one SNP among five candidates per locus listed by Irving-Pease et al. 2024 with genome-wide significant posterior probability (π>99%); an additional 3 loci had

probable evidence of selection in our re-analysis ($50\%<\pi<99\%$). Our analysis increases the chance that some or all of the remaining 4 may be false-positives.

Table S6.1: Summary of genome-wide significant signals of selection across five studies. SNPs within 100 kb of each other are considered a single locus, except for HLA, which is treated as one locus regardless of distance for this reanalysis. For each study, a value of 1 indicates that the locus is reported as genome-wide significant, and 0 otherwise. The Panel ID corresponds to the identifier used in the supplementary figures below to mark each panel, starting with the first letter of the corresponding study. (In Mathieson et al. 2015, there are two signals from the HLA region, while Le et al. 2022 excluded the HLA region from their Table 1, despite its evidence of significant selection across all epochs.)

Variant ID	RSID	Panel ID	Gene Name	Locus	Mathieson et al. 2015	Field et al. 2016	Le et al. 2022	Kerner et al. 2023	Irving-Pease et al. 2024	π
1_22704191_T_G	rs11799474	I1		1	0	0	0	0	1	0.21
1_150596411_C_A	rs7517	L3	*ENSA*	2	0	0	1	0	0	0.54
1_181018799_A_C	rs10797666	L16	*MR1*	3	0	0	1	0	0	0.04
1_202143512_A_G	rs12401678	L4	*PTPRVP*	4	0	0	1	0	0	0.34
1_230854999_C_T	rs7555650	L14	*AGT*	5	0	0	1	0	0	0.23
2_100604753_G_C	rs56127672	I7	*AFF3*	6	0	0	0	0	1	0.82
2_102824201_G_A	rs2310239	L2	*IL1RL2*	7	0	0	1	0	0	>0.99
2_136608646_G_A	rs4988235	M1	*MCM6* *LCT*	8	1	0	0	0	0	>0.99
2_136608646_G_A	rs4988235	L18			0	0	1	0	0	>0.99
2_136608646_G_A	rs4988235	K1			0	0	0	1	0	>0.99
2_136608646_G_A	rs4988235	I11			0	0	0	0	1	>0.99
2_136707982_T_C	rs6754311	F1			0	1	0	0	0	>0.99
3_46954614_A_C	rs201652298	I16		9	0	0	0	0	1	>0.99
4_38745482_T_C	rs10008032	I21	*TLR10* *TLR1*	10	0	0	0	0	1	>0.99
4_38776107_T_G	rs11096955	L22			0	0	1	0	0	>0.99
4_38815502_A_C	rs4833103	M7			1	0	0	0	0	>0.99
4_145506871_C_T	rs6537298	I29		11	0	0	0	0	1	0.27
5_33951693_C_G	rs16891982	M5	*SLC45A2*	12	1	0	0	0	0	>0.99
5_33951693_C_G	rs16891982	L11			0	0	1	0	0	>0.99
5_33952106_C_T	rs185146	K3			0	0	0	1	0	>0.99
5_33958959_C_A	rs28777	I32			0	0	0	0	1	>0.99
5_131675864_A_G	rs272872	M4	*SLC22A4*	13	1	0	0	0	0	>0.99
5_131705458_C_G	rs2631367	I39	*SLC22A5*		0	0	0	0	1	>0.99
5_176836532_A_G	rs1801020	I43	*F12*	14	0	0	0	0	1	>0.99
6_28322296_A_G	rs6903823	M10	*HLA**	15	1	0	0	0	0	>0.99
6_28819880_A_G	rs9257267	F2			0	1	0	0	0	0.92
6_29919779_C_A	rs7747253	I47			0	0	0	0	1	>0.99
6_30746519_G_T	rs3130673	K8			0	0	0	1	0	>0.99
6_32132233_G_A	rs2269424	M9			1	0	0	0	0	>0.99

8_9619909_T_A	rs35231275	I53	*TNKS*	16	0	0	0	0	1	0.71
8_130981907_C_A	rs10956504	L5	*FAM49B*	17	0	0	1	0	0	0.14
8_132664365_A_C	rs7010408	I58		18	0	0	0	0	1	0.25
9_136137657_C_T	rs8176693	I63	*ABO*	19	0	0	0	0	1	>0.99
10_49924070_T_C	rs76203261	F3	*WDFY4*	20	0	1	0	0	0	0.54
11_18167630_A_G	rs4256954	L17	*RP11-113D6.6*	21	0	0	1	0	0	<0.01
11_27679916_C_T	rs6265	L9	*BDNF*	22	0	0	1	0	0	0.04
11_61569830_C_T	rs174546	M2	*FADS1* *FADS2*	23	1	0	0	0	0	>0.99
11_61571478_T_C	rs174550	L19			0	0	1	0	0	>0.99
11_61603510_C_A	rs174576	I67			0	0	0	0	1	>0.99
11_71153459_C_A	rs11603330	L21	*DHCR7*	24	0	0	1	0	0	>0.99
11_71165625_A_G	rs7944926	M8	*NADSYN1*		1	0	0	0	0	>0.99
11_88515022_A_G	rs7119749	M6	*GRM5*	25	1	0	0	0	0	>0.99
12_112007756_C_T	rs653178	M3	*ATXN2*	26	1	0	0	0	0	>0.99
12_112007756_C_T	rs653178	L20			0	0	1	0	0	>0.99
12_112007756_C_T	rs653178	I74			0	0	0	0	1	>0.99
14_103867320_A_G	rs4906319	L13	*MARK3*	27	0	0	1	0	0	0.36
15_28365618_A_G	rs12913832	M11	*HERC2*	28	1	0	0	0	0	0.32
15_28386626_C_T	rs11636232	L12			0	0	1	0	0	>0.99
15_75077367_C_A	rs1378942	I80	*CSK*	29	0	0	0	0	1	>0.99
16_54231250_A_G	rs2010410	L8		30	0	0	1	0	0	0.05
16_69969299_C_G	rs62053262	I82	*WWP2*	31	0	0	0	0	1	0.61
16_80036594_G_T	rs4073089	L7		32	0	0	1	0	0	0.06
17_37355093_C_T	rs57944517	L6	*RPL19*	33	0	0	1	0	0	0.07
17_37388567_C_T	rs75097049	I89			0	0	0	0	1	>0.99
17_44199290_T_C	rs4792830	I95	*KANSL1*	34	0	0	0	0	1	>0.99
19_49206603_C_T	rs281377	L1	*FUT2*	35	0	0	1	0	0	>0.99
19_57499627_G_A	rs62132568	I99		36	0	0	0	0	1	0.05
21_43679554_C_T	rs915843	L15	*ABCG1*	37	0	0	1	0	0	0.12
22_22027348_C_T	rs1669125	L10	*PPIL2*	38	0	0	1	0	0	0.02
22_42340844_T_C	rs1023500	I104	*CENPM*	39	0	0	0	0	1	>0.99

Re-evaluation of results from Mathieson et al. 2015

We evaluated 12 SNPs identified as candidates for selection from Extended Data Table 3 of Mathieson et al. 2015[39]. In our re-evaluation of these 12 SNPs, 10 SNPs (rs4988235, rs16891982, rs2269424, rs174546, rs4833103, rs653178, rs7944926, rs7119749, rs272872, rs6903823) showed a compelling signal of selection with posterior probability π>99% in our analysis. One SNP, rs12913832 at the *OCA2*/*HERC2* locus, showed a signal of selection with posterior probability π=32%, and is likely a real signal of selection as this is the blue eye color variant which other work has shown has been subject to fluctuating selection over space and time, a scenario not tested for in our methodology which explicitly assumes a constant selection coefficient over space and time. The last SNP, rs1979866, did not pass quality control in our analysis. It showed a 5.4% mismatch (P = 4.8e-8) between the genotypes of 450 individuals with both Shotgun and 1240k sequences. Any variant with an error rate greater than 5% or a P value for error less than 1e-5 is filtered out (Supplementary Information section 1). It also had a posterior probability π=2%, so we believe it may be a false-positive due to data artifact (Figure S6.1). Two SNPs, rs6903823 and rs2269424, are from the HLA region.

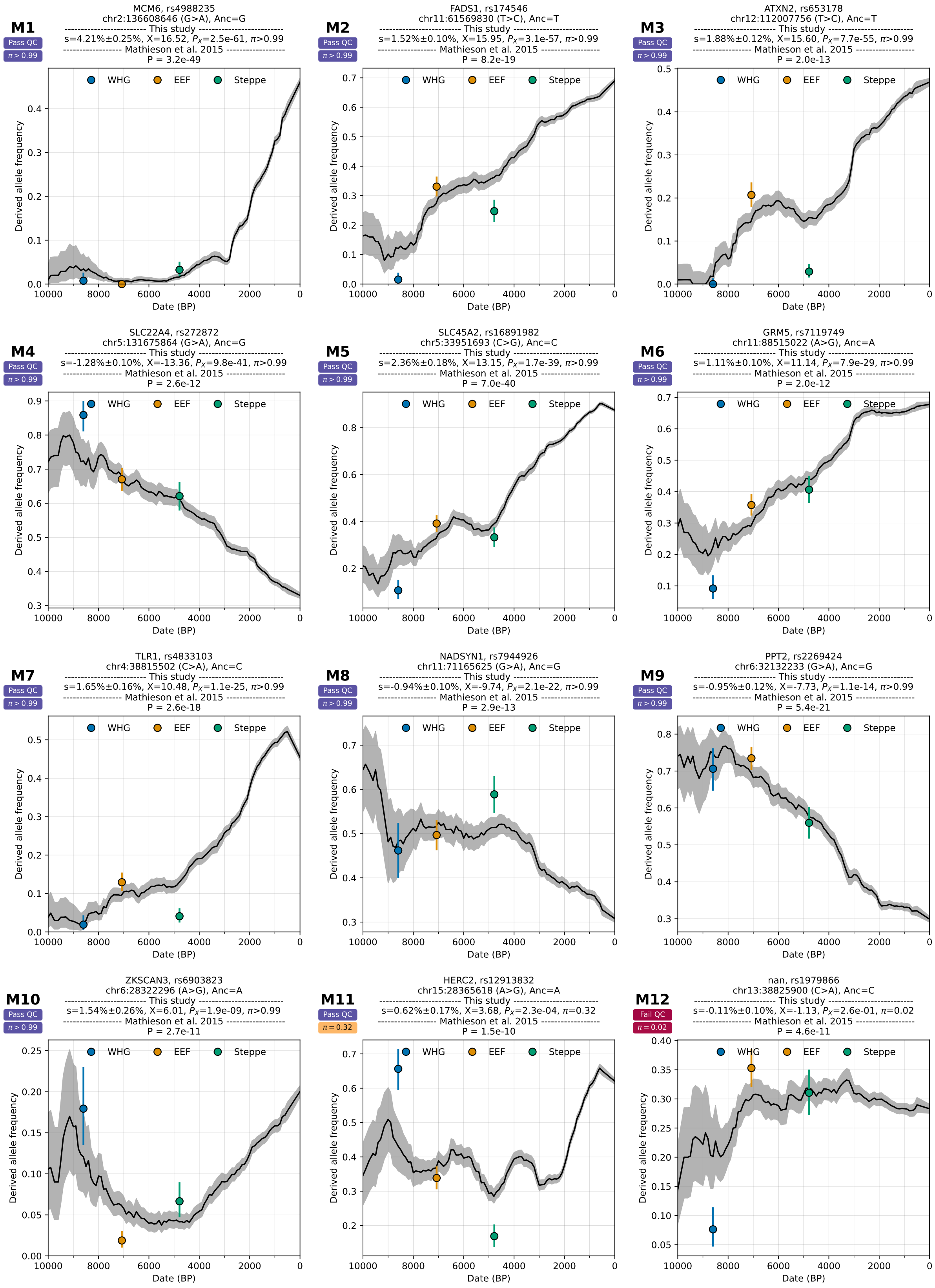

Supplementary Figure S6.1: Re-evaluating 12 signals of selection from Mathieson et al. 2015.

Re-evaluation of results from Field et al. 2016

The singleton density score (SDS) from Field et al. 2016[42] was devised to capture signals of recent selection with particular statistical power during the past 2000 years. This method does not use ancient DNA and is entirely based on patterns of variation in contemporary populations. Here, we evaluated 37 independent tagging SNPs with a p-value of SDS (P_{SDS}) less than 1e-5. We used the PLINK clumping option, prioritizing SNPs by P_{SDS} and using clump_kb = 10 Mbp and clump_r2 = 0.05. SNPs that do not exist in the imputation reference panel are dropped. These independent SNPs are shown in a Manhattan plot of the SDS score (Figure S6.2).

Out of 37 SNPs tagging independent loci with P_{SDS}<1e-5, those at only three loci (*LCT* (F1), *KITLG* (F17), *BNC2* (F19)) produced a strong signal in our ancient DNA time transect analysis (π>99%). The *LCT* signal is one of three genome-wide significant SDS loci (P_{SDS}<5e-8) reported in Field et al. The second locus, *HLA* (F2), has a posterior probability of π=92% in our evaluation. The third locus, *WDFY4* (F3), has a posterior probability of π=54%, with time transect analysis suggesting a greater probability selection before 2000 years BP (π_B=73%) than after (π_A=2%). The frequency of the tagging variant for *WDFY4* (F3) is around 5% in Steppe pastoralists and near zero in Western Hunter-Gatherers (WHG) and Early European Farmers (EEF). Its allele frequency increased rapidly with the arrival of Steppe in Europe and remained stable afterward. This allele frequency trajectory, along with our formal GLMM analysis, suggests that this signal may be an artifact of Steppe admixture, and that the significant SDS signal may be due to unresolved population structure (Figures S6.3-S6.6)

The methodology in Field et al. 2016 is profoundly different from ours and uses a different type of data (not ancient DNA). Thus, while the two scans are maximally powered in the same time period, it is possible and even likely that the Field et al. 2016 methodology is sensitive to some genuine signals that our ancient DNA time transect study misses.

However, the far lower replication rates in the list of highlighted SNPs from Field et al. with less compelling P-values (5e-8 < P_{SDS} < 1e-5) (2 of 34) than for the SNPs with strong P-values (P_{SDS} < 5e-8) raises the possibility that most of the list of 37 SNPs highlighted from Field et al. 2016 were false-positives likely due unresolved population structure and the threshold for including a SNPs in the list was not stringent enough.

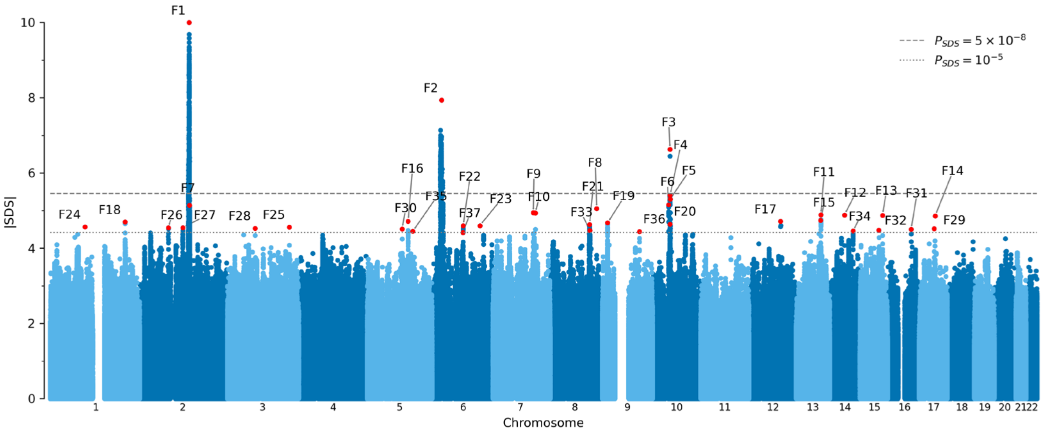

Figure S6.2: Manhattan plot of SDS score from Field et al. 2016. Red circles indicate 37 independent tagging SNPs with P_{SDS} < 1e-5. Each SNP is annotated with the panel name in the following Figures S6.3-S6.6.

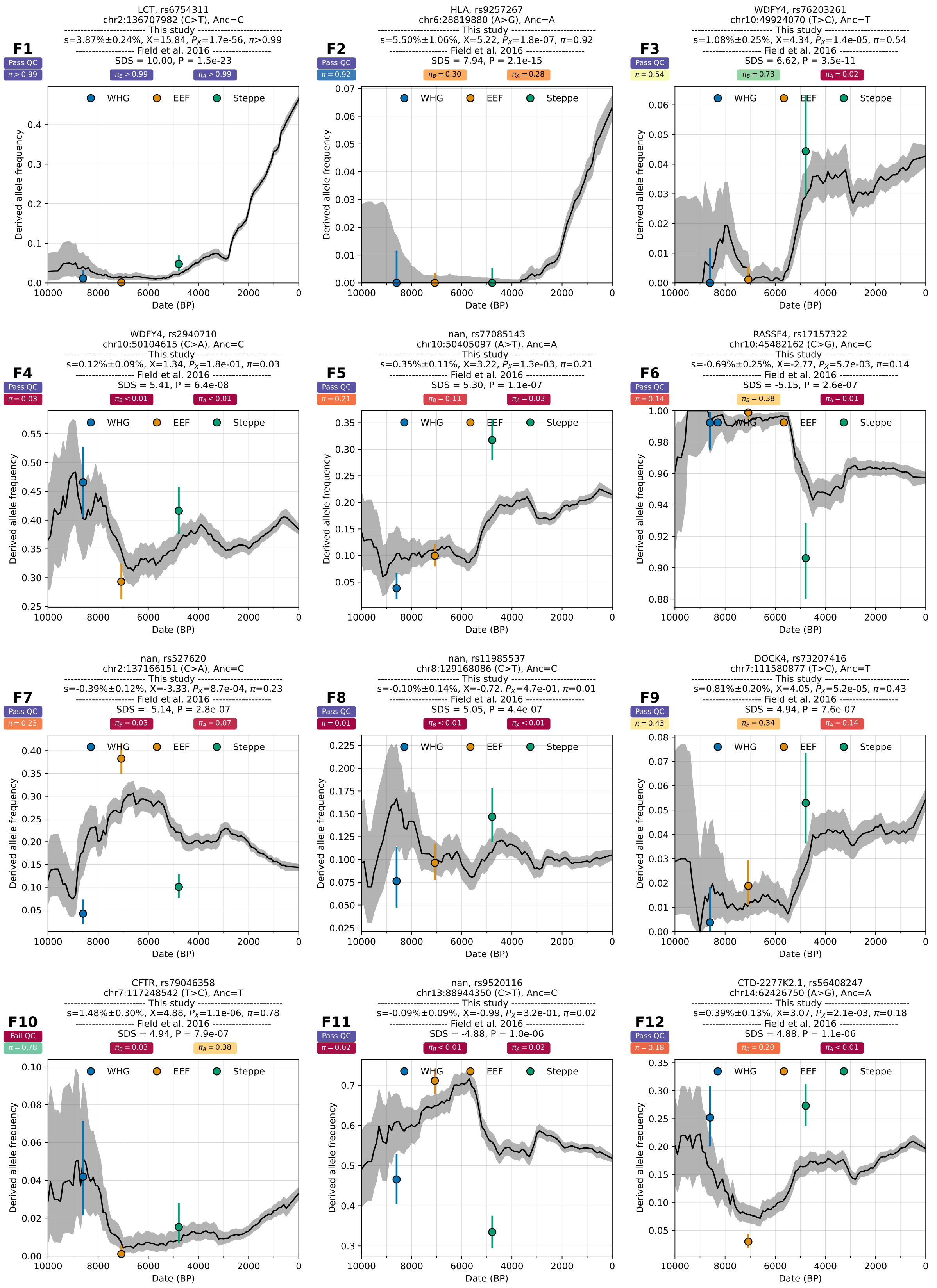

Supplementary Figure S6.3: Re-evaluating selection signals from Fields et al. 2016. A and B refer to After and Before 2000 BP.

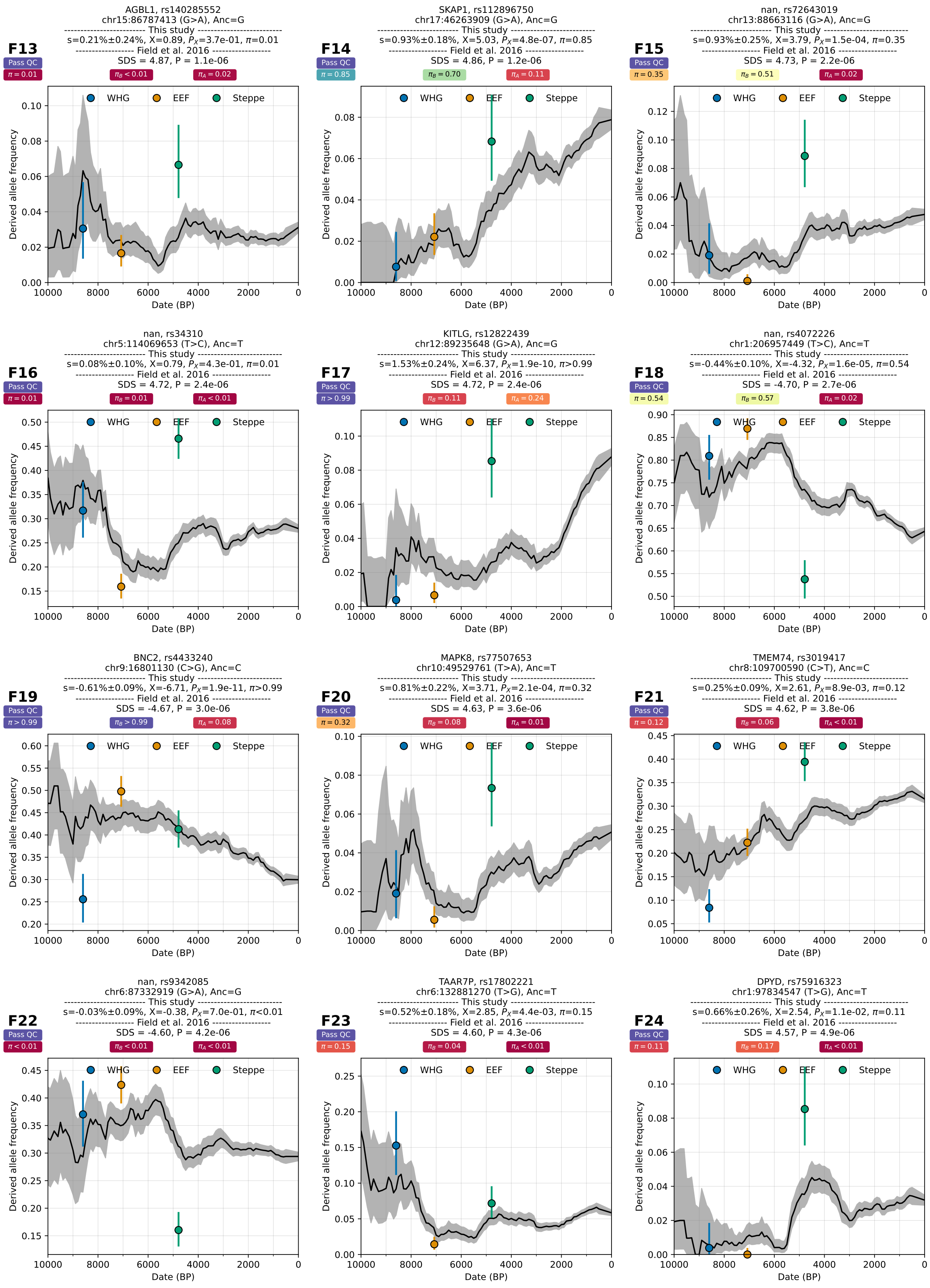

Supplementary Figure S6.4: Re-evaluating selection signals from Fields et al. 2016. A and B refer to After and Before 2000 BP.

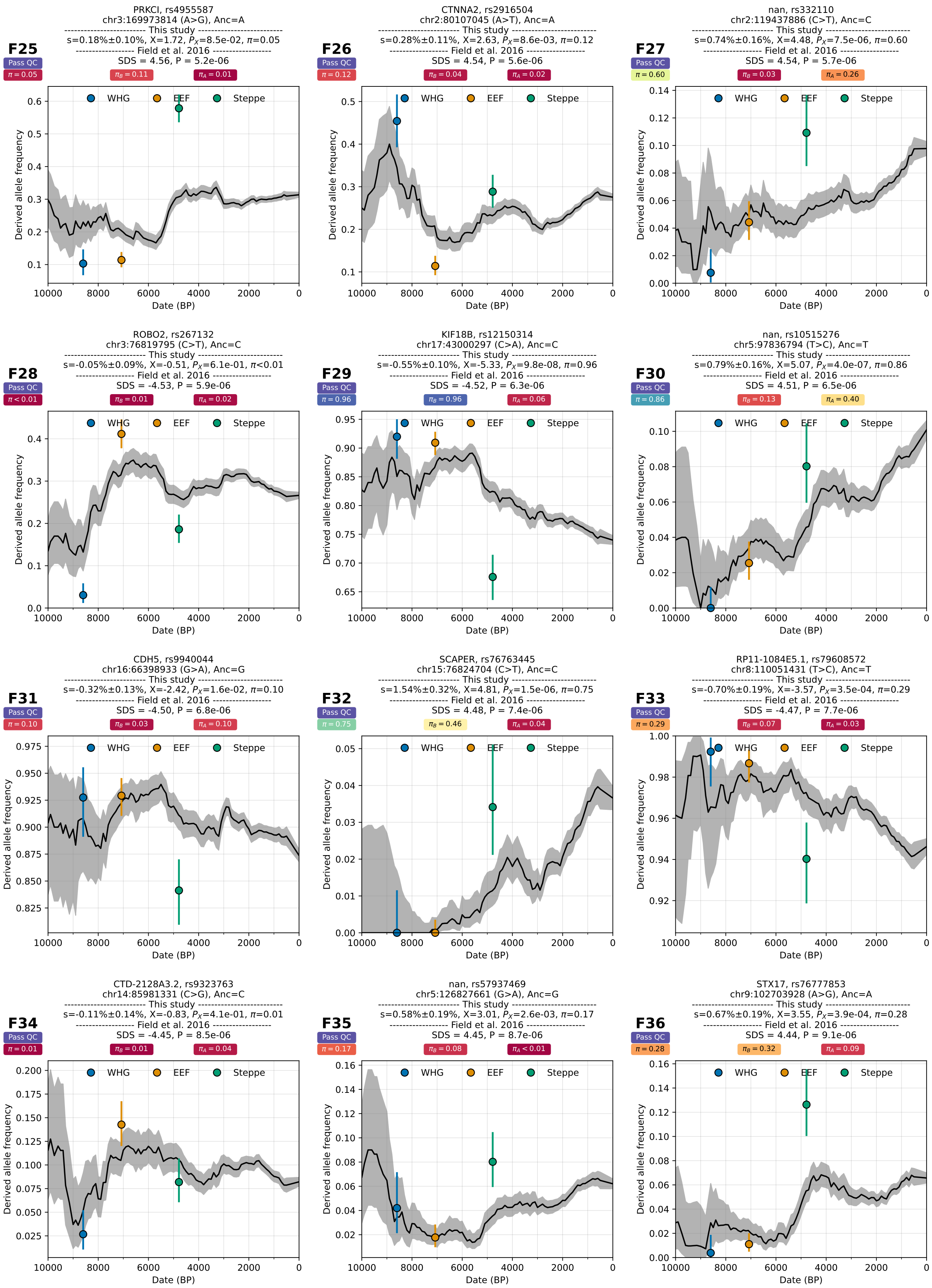

Supplementary Figure S6.5: Re-evaluating selection signals from Fields et al. 2016. A and B refer to After and Before 2000 BP.

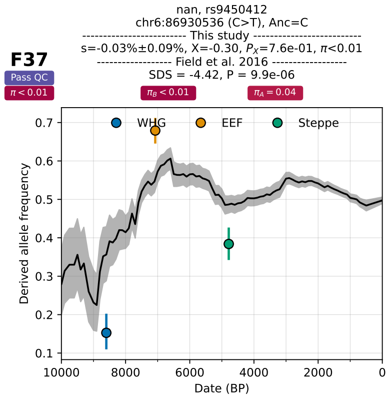

Supplementary Figure S6.6: Re-evaluating selection signals from Fields et al. 2016. A and B refer to After and Before 2000 BP.

Re-evaluation of results from Le et al. 2022

We evaluated 25 signals of selection at 24 loci detected during the Neolithic (N), Bronze Age (B), and Historical (H) periods, from Table 1 of Le et al. 2022[40]. Two of these signals are from SNP rs16891982 at the *SLC45A2* locus, which appear as a significant signal in both the Bronze Age and Historical periods. Two of these 24 SNPs are not present in the imputation reference panel and, therefore, are not re-evaluated here. This leaves 22 SNPs to re-evaluate, of which 9 produce strong signals (π>99%) in our full time transect analysis. We also analyzed three different time transects separately using the GLMM approach to mimic the three time periods of Le et al. 2022.

From the Neolithic period, we evaluated 10 candidate SNPs (rs281377, rs2310239, rs7517, rs12401678, rs4073089, rs57944517, rs10956504, rs2010410, rs6265, rs1669125). Only rs281377 at *FUT2* showed a compelling signal of selection (π_N>99%) during the Neolithic period (Figure S6.7).

From the Bronze Age period, two candidate SNPs (rs143482314, rs117124595) from Le et al. 2022 with signals of selection are not in the 1000 Genomes Project SNP set and were not analyzed here. Of the remaining 7 candidate SNPs (rs16891982, rs11636232, rs4906319, rs7555650, rs915843, rs10797666, rs4256954), only rs16891982 at *SLC45A2* showed a strong signal of selection (π_B>99%) during the Bronze Age period (Figure S6.8).

From the Historical period, we evaluated 6 candidate SNPs (rs16891982, rs4988235, rs174550, rs653178, rs11603330, rs11096955) from Le et al. 2022. Five showed strong signals of selection (π_H>99%), with only rs11096955 having a lower posterior probability (π_H=15%) for selection (Figure S6.9). All these 6 showed strong signals of selection (π_B>99%) during the earlier Bronze Age period as well, with only rs16891982 at the *SLC45A2* locus (Figure S6.8, panel L11) being reported as a Bronze Age-specific signal.

Some co-authors of this study are also co-authors of Le et al. 2022. In an updated analysis, which includes additional measures to control for uncertainty in admixture proportions, allele frequency uncertainty in the source and target populations, and stochasticity in sampling, Le et al. now report 22 genome-wide significant hits at 21 loci. Of these, 17 validate with >99% posterior probability in our analysis (2 of 4 in the Neolithic, 3 of 6 in the Bronze Age, and 12 of 12 in the Historical Period) (Vagheesh Narasimhan, personal communication).

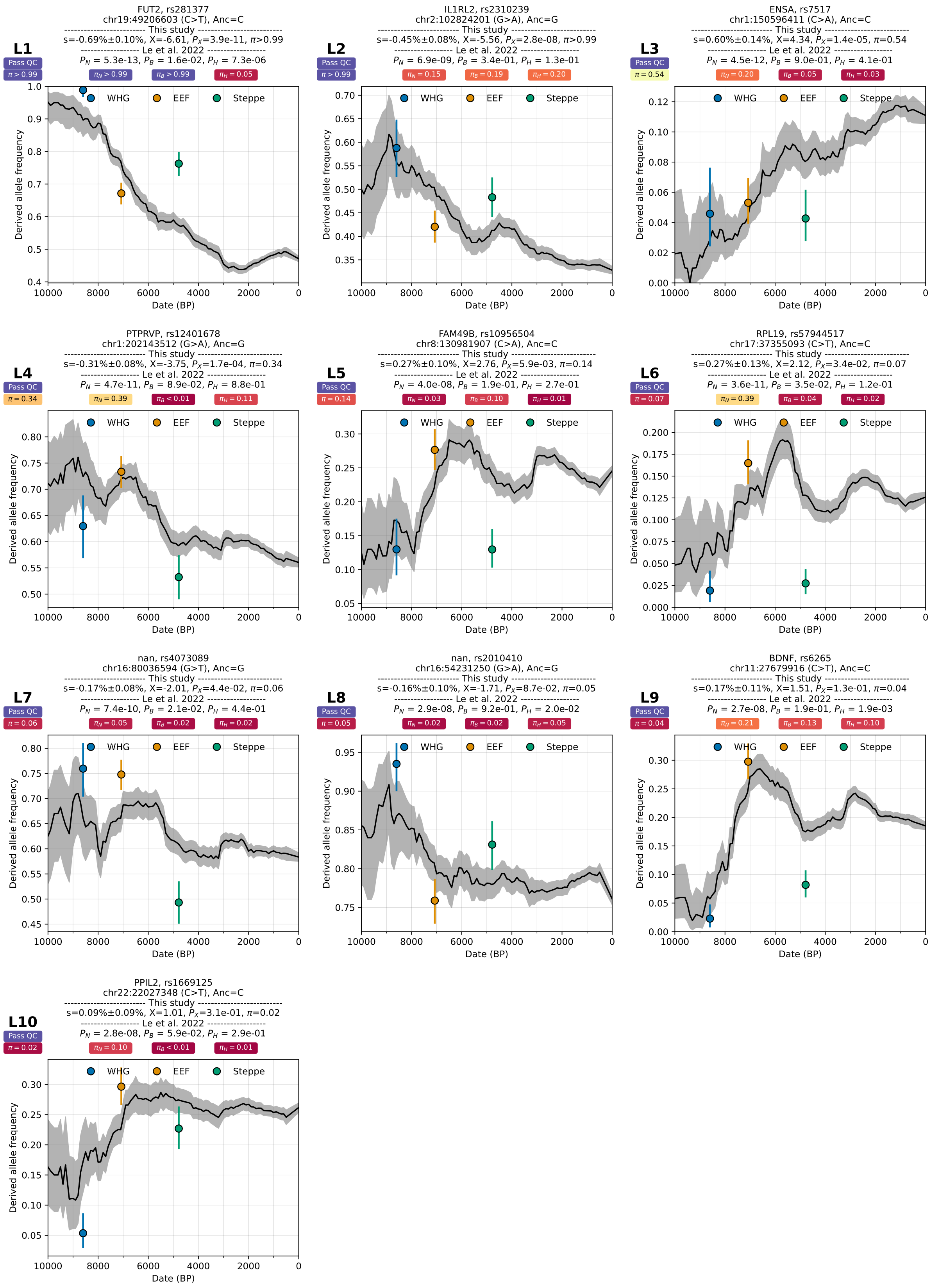

Supplementary Figure S6.7: Re-evaluating signals of selection from Le et al. 2022 from the Neolithic (N) period.

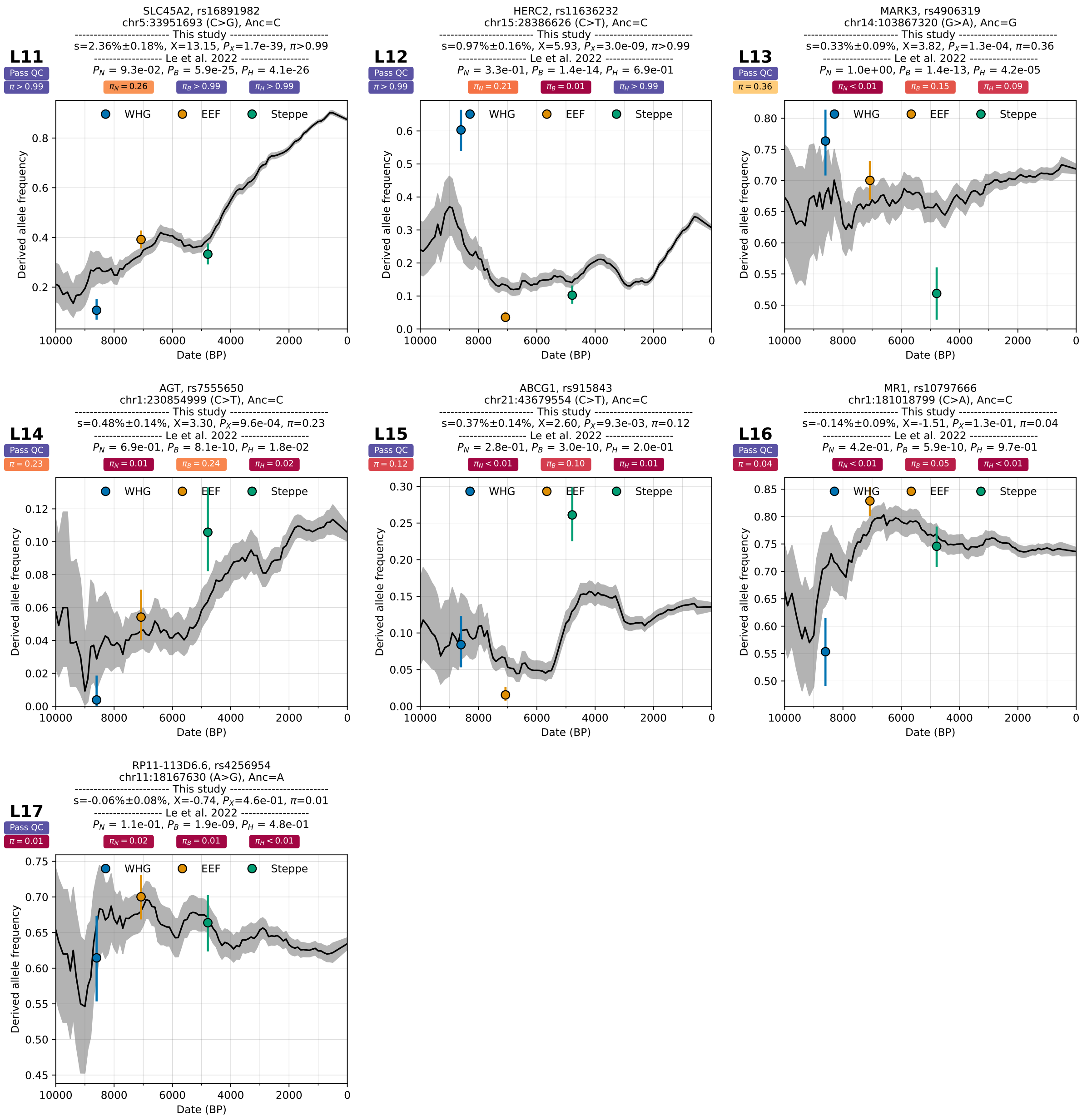

Supplementary Figure S6.8: Re-evaluating signals of selection from Le et al. 2022 from the Bronze Age (B).

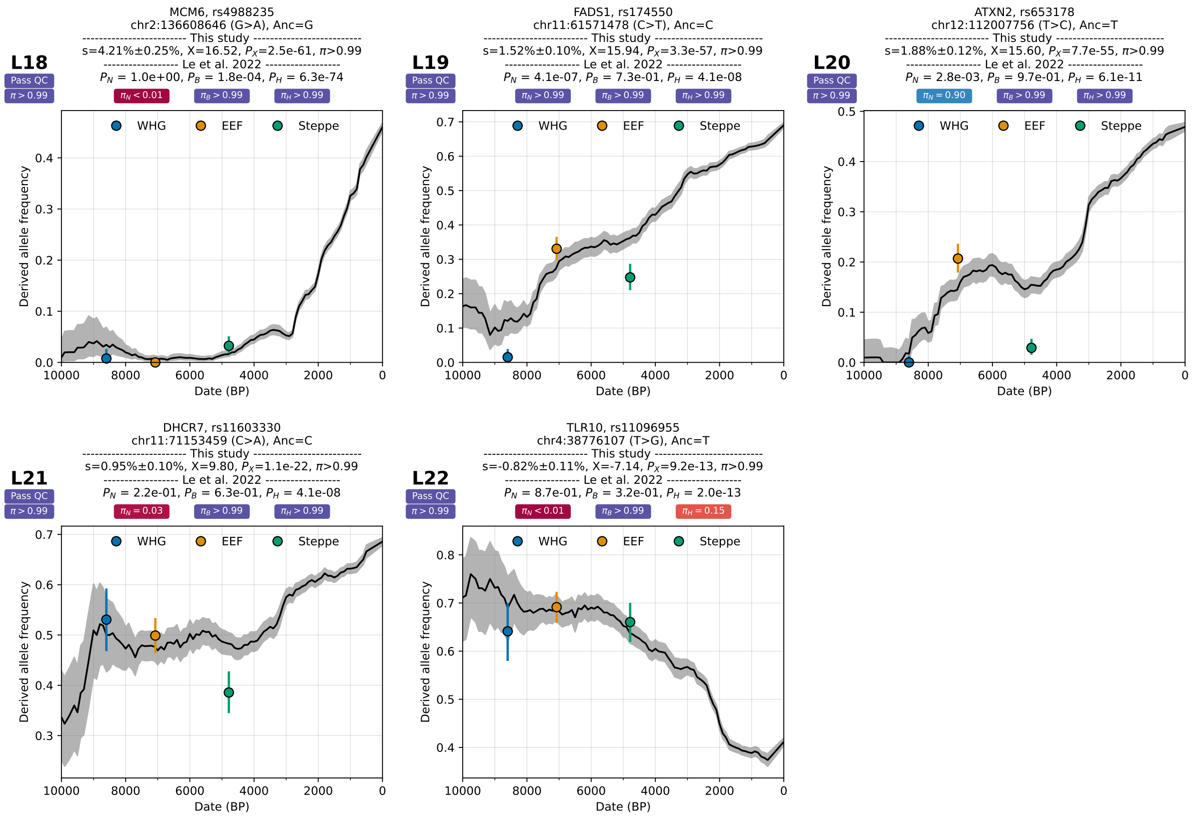

Supplementary Figure S6.9: Re-evaluating signals of selection from Le et al. 2022 for the Historical (H) period. Panel L11 from Supplementary Figure S6.8 is a candidate signal of selection for both the Bronze Age and the Historical period.

Re-evaluation of results from Kerner et al. 2023

We evaluated 89 SNPs identified as candidates for positive selection on the derived allele from Table S2 of Kerner et al. 2023[41], along with 50 other candidate missense SNPs for negative selection from Table S7 of the same study. Only three candidate positive selection SNPs (rs4988235 at the *LCT* locus, rs185146 at the *SLC45A2* locus, and rs3130673 at the HLA locus) meet the genome-wide significant P-value threshold of 5e-8 for 'pbeta' test statistics of Kerner et al. 2023, and all three are replicated in our analysis with posterior probability (π>99%; Figure S6.10). In our analysis, only 14 of 125 candidates passing QC show strong evidence for selection (π>99%), while an additional 9 are probable signals of selection in our re-analysis (50%<π<99%) This suggests that most of the candidates are false positives, likely due to unresolved population structure and data artifacts (Figures S6.11-S6.23).

These 139 SNPs are listed in Table S6.2. Three positive selection candidate SNPs (rs11125238, rs34969536, rs4717903) are not in the 1000 Genomes Project reference panel, and therefore we did not analyze them. Four positive selection and seven negative selection candidate SNPs did not pass quality control (QC) in our study.

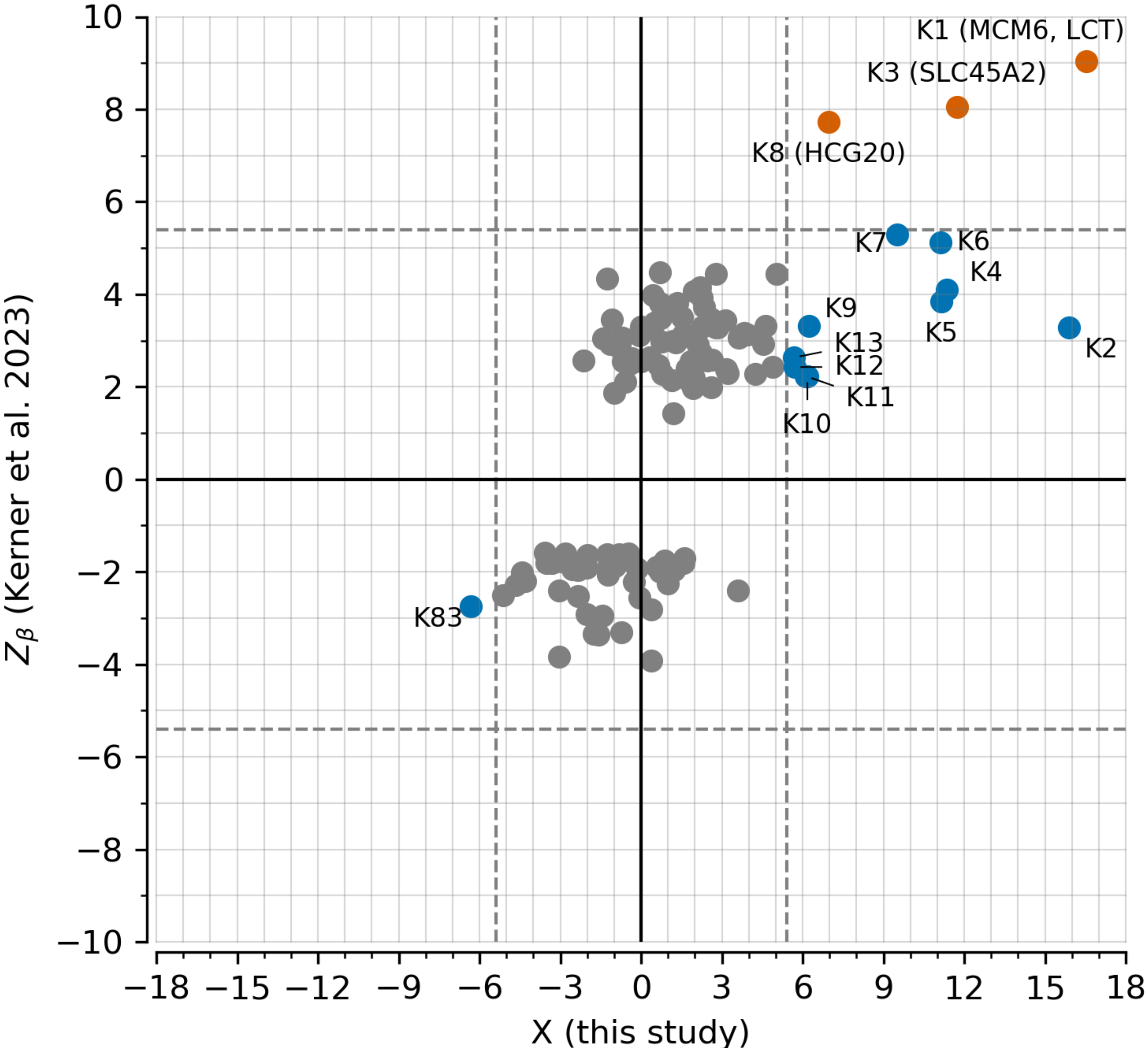

Figure S6.10: Comparison of Z_β from Kerner et al. (2023) for positive and negative selection candidates with the X score from this study. Labels indicate the panel ID for frequency trajectory plots in the supplementary figures below. The dashed gray line represents ±5.45, marking the classic genome-wide p-value threshold of 5e-8.

Table S6.2: List of SNPs from Kerner et al. 2023 analyzed in this study. Panel ID is the identifier used in the supplementary figures below to mark each panel. The Variant ID is defined as the CHROM_POS_REF_ALT using the human genome reference assembly version hg19/GRCh37.

RSID	Panel ID	Variant ID	Ancestral Allele	Gene name	Selection Type	QC	Kerner et al. 2023		π
							P_{sel}	P_{β}	
rs4988235	K1	2_136608646_G_A	G	*MCM6*	Positive	Pass	<2.0e-6	1.6E-19	>0.99
rs174537	K2	11_61552680_G_T	T	*TMEM258*	Positive	Pass	2.7E-05	1.0E-03	>0.99
rs185146	K3	5_33952106_C_T	C	*SLC45A2*	Positive	Pass	<2.0e-6	8.7E-16	>0.99
rs4705844	K4	5_131365784_A_C	A	*AC034228.2-IL3*	Positive	Pass	<2.0e-6	4.3E-05	>0.99
rs10765770	K5	11_88513636_G_A	G	*GRM5*	Positive	Pass	2.0E-06	1.2E-04	>0.99
rs10008492	K6	4_38765720_C_T	T	*RNA5SP158-TLR10*	Positive	Pass	1.2E-05	3.1E-07	>0.99
rs4792831	K7	17_44206665_G_A	G	*RNU7-101P*	Positive	Pass	1.1E-05	1.2E-07	>0.99
rs3130673	K8	6_30746519_G_T	G	*HCG20*	Positive	Pass	<2.0e-6	1.1E-14	>0.99
rs11674302	K9	2_102887128_T_C	T	*AC007248.6-IL1RL1*	Positive	Pass	2.8E-05	9.4E-04	>0.99
rs11042594	K10	11_2117403_A_G	G	*H19-IGF2*	Positive	Pass	8.5E-04	2.6E-02	>0.99
rs7141996	K11	14_93094298_G_A	G	*RIN3*	Positive	Pass	8.5E-04	2.6E-02	>0.99
rs1229758	K12	7_114229139_G_A	G	*FOXP2*	Positive	Pass	3.9E-04	1.5E-02	>0.99
rs36083353	K13	4_42041692_A_T	A	*SLC30A9*	Positive	Pass	2.1E-04	8.5E-03	>0.99
rs10095927	K14	8_10729177_G_T	G	*RP11-177H2.2-XKR6*	Positive	Pass	<2.0e-6	8.7E-06	0.85
rs10513801	K15	3_185822353_T_G	T	*ETV5*	Positive	Pass	5.1E-04	1.5E-02	0.79
rs11057805	K16	12_125246993_G_A	G	*NCOR2-SCARB1*	Positive	Pass	2.6E-05	8.9E-04	0.66
rs8022676	K17	14_77825746_A_C	C	*RP11-493G17.4*	Positive	Pass	9.1E-05	3.5E-03	0.63
rs17605165	K18	16_11297562_C_T	C	*RP11-396B14.2*	Positive	Pass	8.0E-04	2.3E-02	0.5
rs8033465	K19	15_69773781_G_T	G	*RP11-279F6.1*	Positive	Pass	6.9E-05	1.7E-03	0.4
rs389663	K20	6_117868051_T_C	T	*DCBLD1*	Positive	Pass	4.0E-05	1.5E-03	0.36
rs72700902	K21	1_150633347_C_T	C	*GOLPH3L*	Positive	Pass	5.8E-05	2.2E-03	0.31
rs10507766	K22	13_69940025_A_G	A	*LINC00401-SRSF1P1*	Positive	Pass	5.6E-04	2.1E-02	0.21
rs55846849	K23	5_460710_G_A	G	*EXOC3*	Positive	Pass	6.2E-04	1.7E-02	0.2
rs13307276	K24	7_150061525_T_C	T	*REPIN1*	Positive	Pass	2.0E-05	6.0E-04	0.2
rs713217	K25	11_69579070_T_C	T	*RP11-300I6.5-AP001888.1*	Positive	Pass	3.0E-05	1.0E-03	0.15
rs10496379	K26	2_104602547_A_T	A	*RP11-76I14.1*	Positive	Pass	3.0E-05	1.0E-03	0.14
rs10515671	K27	5_151845342_A_G	A	*NMUR2-CTC-550M4.1*	Positive	Pass	1.6E-05	9.0E-06	0.14
rs1531212	K28	19_13951830_G_A	G	*MIR24-2*	Positive	Pass	3.0E-04	9.8E-03	0.12
rs10841899	K29	12_8716873_G_A	G	*RP11-561P12.5*	Positive	Pass	1.3E-03	4.7E-02	0.12
rs721992	K30	10_61539402_G_T	G	*LINC00948-CCDC6*	Positive	Pass	1.7E-05	5.2E-04	0.12
rs4280687	K31	4_156105105_C_T	C	*RBM46-RP11-92A5.2*	Positive	Pass	2.4E-04	1.0E-02	0.1
rs1055472	K32	12_125510104_G_A	A	*BRI3BP*	Positive	Pass	7.0E-06	2.0E-04	0.09
rs62054458	K33	17_13672024_C_T	C	*COX10-AS1*	Positive	Pass	2.7E-05	9.8E-04	0.08
rs3858036	K34	9_2968107_G_A	G	*CARM1P1*	Positive	Pass	<2.0e-6	8.6E-05	0.08
rs2974658	K35	5_2960267_G_T	G	*RP11-35O7.1*	Positive	Pass	1.8E-04	6.3E-03	0.08
rs9840198	K36	3_184770686_C_T	C	*VPS8*	Positive	Pass	<2.0e-6	3.2E-05	0.08
rs13009356	K37	2_8530522_G_A	G	*LINC00299-AC011747.3*	Positive	Pass	2.4E-04	1.0E-02	0.07
rs7422	K38	14_89884022_A_C	A	*FOXN3*	Positive	Pass	9.3E-05	3.7E-03	0.07
rs4922511	K39	10_48402818_T_C	C	*RBP3-GDF2*	Positive	Pass	3.7E-04	1.2E-02	0.07
rs1076005	K40	18_5973037_C_T	C	*L3MBTL4*	Positive	Pass	5.1E-05	1.9E-03	0.06
rs7958347	K41	12_113325061_C_T	C	*RPH3A*	Positive	Pass	<2.0e-6	4.8E-05	0.06
rs586554	K42	9_22796540_T_C	C	*RP11-399D6.2*	Positive	Pass	8.8E-04	2.9E-02	0.06
rs1047616	K43	17_25642522_G_A	A	*WSB1*	Positive	Pass	1.4E-03	4.9E-02	0.06
rs6694101	K44	1_2020489_C_T	C	*PRKCZ*	Positive	Pass	4.9E-04	1.7E-02	0.06
rs3780710	K45	9_132942794_G_A	G	*NCS1*	Positive	Pass	3.5E-04	1.1E-02	0.05
rs12666876	K46	7_155417249_G_A	G	*AC009403.2*	Positive	Pass	4.9E-04	3.5E-02	0.05
rs75770273	K47	6_53877078_C_T	C	*ERHP2*	Positive	Pass	8.1E-05	2.0E-03	0.05
rs34404720	K48	2_86879419_A_G	A	*CHMP3*	Positive	Pass	6.0E-04	1.7E-02	0.04
rs6698312	K49	1_164206815_G_T	G	*U3-NMNAT1P2*	Positive	Pass	4.5E-05	1.7E-03	0.04
rs6900553	K50	6_10231925_C_T	C	*RNU6ATAC21P-RP1-290I10.2*	Positive	Pass	<2.0e-6	4.7E-04	0.04
rs3003615	K51	9_130997892_G_A	G	*DNM1*	Positive	Pass	6.5E-05	2.4E-03	0.03
rs2426652	K52	20_55294098_A_G	A	*AL133232.1*	Positive	Pass	3.0E-06	1.4E-04	0.03
rs10809509	K53	9_1162013_A_C	A	*RPS27AP14*	Positive	Pass	8.4E-05	3.1E-03	0.03
rs8079769	K54	17_9588455_G_A	G	*USP43*	Positive	Pass	2.0E-05	2.4E-04	0.03
rs10188894	K55	2_6643733_A_G	A	*AC021021.2*	Positive	Pass	<2.0e-6	1.4E-05	0.03
rs12580172	K56	12_1623989_G_A	G	*LINC00942-WNT5B*	Positive	Pass	6.1E-03	1.6E-01	0.02
rs11884056	K57	2_85339839_C_T	C	*LSM3P3-TCF7L1*	Positive	Pass	1.1E-04	2.7E-03	0.02
rs12715075	K58	3_2620454_C_T	T	*CNTN4*	Positive	Pass	4.8E-04	3.3E-02	0.02
rs4405041	K59	9_99088478_T_G	G	*SLC35D2*	Positive	Pass	9.0E-05	3.4E-03	0.02
rs12033048	K60	1_246429681_T_C	T	*SMYD3*	Positive	Pass	2.1E-05	5.6E-04	0.02
rs62436708	K61	7_1323037_C_T	C	*AC073094.4-MICALL2*	Positive	Pass	1.7E-03	6.2E-02	0.02
rs176482	K62	7_105730038_G_A	G	*SYPL1*	Positive	Pass	6.1E-04	2.3E-02	0.01
rs3957465	K63	5_5817703_G_A	G	*KIAA0947-CTC-471C19.1*	Positive	Pass	9.9E-05	4.5E-03	0.01
rs12711473	K64	16_87224293_A_G	G	*C16orf95*	Positive	Pass	7.9E-05	2.9E-03	0.01
rs8031453	K65	15_91108674_A_C	A	*CRTC3*	Positive	Pass	2.1E-05	4.8E-04	0.01
rs62043998	K66	16_81921203_C_T	C	*PLCG2*	Positive	Pass	1.6E-05	7.6E-06	0.01
rs4374563	K67	3_31776980_G_A	G	*OSBPL10*	Positive	Pass	3.0E-06	1.4E-04	<0.01
rs1364095	K68	16_79566150_G_C	G	*RP11-467I17.1-MAF*	Positive	Pass	6.0E-05	2.2E-03	<0.01

rs11059425	K69	12_128441207_T_G	T	*LINC00507-RP11-349K16.1*	Positive	Pass	9.0E-06	1.1E-02	<0.01
rs2631934	K70	8_21052978_G_A	A	*AC021613.1-RP11-24P4.1*	Positive	Pass	1.6E-04	1.4E-02	<0.01
rs1009543	K71	10_3319474_A_G	A	*RP11-195B3.1-RP11-482E14.1*	Positive	Pass	1.0E-03	3.6E-02	<0.01
rs2291897	K72	3_33419422_C_T	C	*FBXL2*	Positive	Pass	2.2E-05	6.9E-04	<0.01
rs4745827	K73	10_65659657_A_G	A	*RP11-170M17.2*	Positive	Pass	<2.0e-6	6.8E-05	<0.01
rs35345724	K74	7_136627956_A_G	A	*KRT8P51*	Positive	Pass	4.1E-04	1.3E-02	<0.01
rs10841952	K75	12_22289304_A_G	A	*ST8SIA1*	Positive	Pass	2.2E-04	9.0E-03	<0.01
rs2619105	K76	10_118977123_C_A	C	*KCNK18-RP11-501J20.5*	Positive	Pass	<2.0e-6	8.8E-03	<0.01
rs458552	K77	9_4833437_C_T	C	*RCL1*	Positive	Pass	2.1E-04	8.3E-03	<0.01
rs11189359	K78	10_99517616_C_A	C	*ZFYVE27*	Positive	Pass	<2.0e-6	1.2E-03	<0.01
rs4863449	K79	4_189987905_C_T	C	*RP11-818C3.1-RP11-706F1.1*	Positive	Pass	5.1E-05	1.9E-03	<0.01
rs8103030	K80	19_23568661_G_A	G	*CTB-175P5.1*	Positive	Pass	5.4E-05	1.4E-03	<0.01
rs9314061	K81	5_164760466_C_T	C	*CTB-181F24.1-CTC-535M15.2*	Positive	Pass	2.4E-04	1.1E-02	<0.01
rs10501651	K82	11_87527561_G_A	G	*RP11-665E10.5*	Positive	Pass	2.9E-05	9.7E-04	<0.01
rs12146727	K83	12_7170336_G_A	G	*C1S*	Negative	Pass	5.0E-04	6.0E-03	>0.99
rs3775291	K84	4_187004074_C_T	C	*TLR3*	Negative	Pass	1.2E-03	1.2E-02	0.89
rs2305637	K85	3_47045846_C_T	C	*NBEAL2*	Negative	Pass	2.0E-03	2.2E-02	0.68
rs7722711	K86	5_75906851_T_C	T	*IQGAP2*	Negative	Pass	4.9E-03	4.4E-02	0.57
rs3814541	K87	9_109689752_C_T	C	*ZNF462*	Negative	Pass	2.5E-03	2.8E-02	0.53
rs1051489	K88	5_32400266_A_G	A	*ZFR*	Negative	Pass	1.4E-03	1.6E-02	0.3
rs2366926	K89	5_89988504_A_G	A	*GPR98*	Negative	Pass	9.8E-03	1.1E-01	0.29
rs4916685	K90	5_89979698_C_T	C	*GPR98*	Negative	Pass	6.0E-03	6.9E-02	0.28
rs1064583	K91	6_116446576_A_G	G	*COL10A1*	Negative	Pass	6.2E-03	7.1E-02	0.23
rs17545756	K92	7_150732812_C_T	C	*ABCB8*	Negative	Pass	1.8E-03	1.6E-02	0.18
rs948962	K93	11_76919478_C_A	A	*MYO7A*	Negative	Pass	<2.0e-6	1.2E-04	0.18
rs1799977	K94	3_37053568_A_G	A	*MLH1*	Negative	Pass	6.8E-03	7.6E-02	0.16
rs1124649	K95	2_27260469_G_A	G	*TMEM214*	Negative	Pass	9.6E-03	1.1E-01	0.14
rs11209026	K96	1_67705958_G_A	G	*IL23R*	Negative	Pass	7.3E-03	7.0E-02	0.13
rs2274654	K97	9_98691137_T_C	T	*ERCC6L2*	Negative	Pass	5.0E-03	5.1E-02	0.11
rs868738	K98	10_115381747_G_A	G	*NRAP*	Negative	Pass	4.4E-03	5.0E-02	0.09
rs2298260	K99	9_21029330_T_C	T	*PTPLAD2*	Negative	Pass	1.1E-03	1.1E-02	0.09
rs678892	K100	15_55632859_G_T	T	*PIGB*	Negative	Pass	2.8E-04	3.5E-03	0.06
rs2298316	K101	10_101147692_G_A	G	*CNNM1*	Negative	Pass	5.6E-03	5.6E-02	0.06
rs34899	K102	5_95091201_A_G	G	*RHOBTB3*	Negative	Pass	9.1E-03	1.0E-01	0.06
rs13009282	K103	2_68364478_T_C	T	*WDR92*	Negative	Pass	4.0E-05	8.5E-04	0.05
rs11568591	K104	17_48761053_G_A	G	*ABCC3*	Negative	Pass	9.8E-03	8.7E-02	0.04
rs77491573	K105	12_124288264_G_A	G	*DNAH10*	Negative	Pass	7.9E-03	7.1E-02	0.04
rs11080134	K106	17_29161503_A_G	A	*ATAD5*	Negative	Pass	3.9E-05	7.8E-04	0.04
rs3803716	K107	16_24802325_C_T	C	*TNRC6A*	Negative	Pass	2.6E-04	3.2E-03	0.03
rs16885	K108	6_16306751_G_A	G	*ATXN1*	Negative	Pass	9.3E-03	1.1E-01	0.02
rs4935502	K109	10_55955444_T_G	T	*PCDH15*	Negative	Pass	4.8E-03	4.9E-02	0.02
rs34536443	K110	19_10463118_G_C	G	*TYK2*	Negative	Pass	4.5E-03	3.9E-02	0.02
rs2287059	K111	2_10717806_C_T	C	*NOL10*	Negative	Pass	5.5E-03	6.5E-02	0.02
rs2275477	K112	1_36886117_C_T	C	*OSCP1*	Negative	Pass	5.3E-03	6.3E-02	0.02
rs2232607	K113	20_36993333_A_G	A	*LBP*	Negative	Pass	3.1E-03	2.5E-02	0.02
rs2276774	K114	3_122646828_A_G	A	*SEMA5B*	Negative	Pass	5.9E-03	6.0E-02	0.02
rs8052655	K115	16_67409180_G_A	G	*LRRC36*	Negative	Pass	8.9E-03	7.9E-02	0.01
rs3829765	K116	14_58605790_G_A	G	*C14orf37*	Negative	Pass	9.2E-03	1.0E-01	0.01
rs2271694	K117	10_71874784_C_T	C	*AIFM2*	Negative	Pass	3.0E-04	9.2E-04	0.01
rs45559835	K118	5_155935708_G_A	G	*SGCD*	Negative	Pass	5.3E-03	4.6E-02	<0.01
rs2270856	K119	2_234741808_C_T	C	*MROH2A*	Negative	Pass	6.9E-03	5.9E-02	<0.01
rs2303291	K120	2_24431184_C_T	C	*ITSN2*	Negative	Pass	9.6E-03	1.1E-01	<0.01
rs12609039	K121	19_11348960_G_A	G	*DOCK6*	Negative	Pass	2.4E-05	8.6E-05	<0.01
rs10083789	K122	16_23080634_C_A	C	*USP31*	Negative	Pass	4.0E-04	5.0E-03	<0.01
rs2306541	K123	12_133428242_G_A	G	*CHFR*	Negative	Pass	2.3E-03	2.6E-02	<0.01
rs16853333	K124	2_168108032_G_A	G	*XIRP2*	Negative	Pass	6.2E-03	5.3E-02	<0.01
rs11204546	K125	1_248059712_T_C	C	*OR2W3*	Negative	Pass	1.0E-03	1.1E-02	<0.01
rs8176635	K126	9_136152009_G_A	G	*ABO*	Positive	Fail	5.4E-05	1.4E-03	0.35
rs4077347	K127	16_29036915_G_A	G	*LAT-CTB-134H23.2*	Positive	Fail	5.2E-03	1.8E-01	0.18
rs17080528	K128	3_49389842_C_T	C	*GPX1*	Positive	Fail	1.9E-05	5.6E-04	0.16
rs11150556	K129	16_83270541_T_C	T	*CDH13*	Positive	Fail	5.2E-04	2.0E-02	0.02
rs61732547	K130	9_113233652_C_T	C	*SVEP1*	Negative	Fail	<2.0e-6	1.4E-07	>0.99
rs3732380	K131	3_39307562_C_T	C	*CX3CR1*	Negative	Fail	1.6E-05	4.5E-06	0.97
rs2291375	K132	3_105264129_G_A	G	*ALCAM*	Negative	Fail	4.0E-05	7.2E-05	0.49
rs113575767	K133	11_125765587_C_T	C	*PUS3*	Negative	Fail	1.7E-05	5.1E-06	0.33
rs1042311	K134	22_46627780_C_T	C	*PPARA*	Negative	Fail	2.9E-04	8.5E-04	0.14
rs7594497	K135	2_55872538_T_C	T	*PNPT1*	Negative	Fail	6.8E-05	3.0E-04	0.08
rs16945138	K136	17_11556248_C_T	C	*DNAH9*	Negative	Fail	8.4E-05	2.7E-04	0.02
rs11125238	NA	NA	G	*RNU6-439P-RPL7P13*	Positive	NA	3.1E-05	1.0E-03	NA
rs34969536	NA	NA	A	*AC008984.6*	Positive	NA	1.0E-04	2.6E-03	NA
rs4717903	NA	NA	C	*GTF2I*	Positive	NA	4.6E-04	2.8E-02	NA

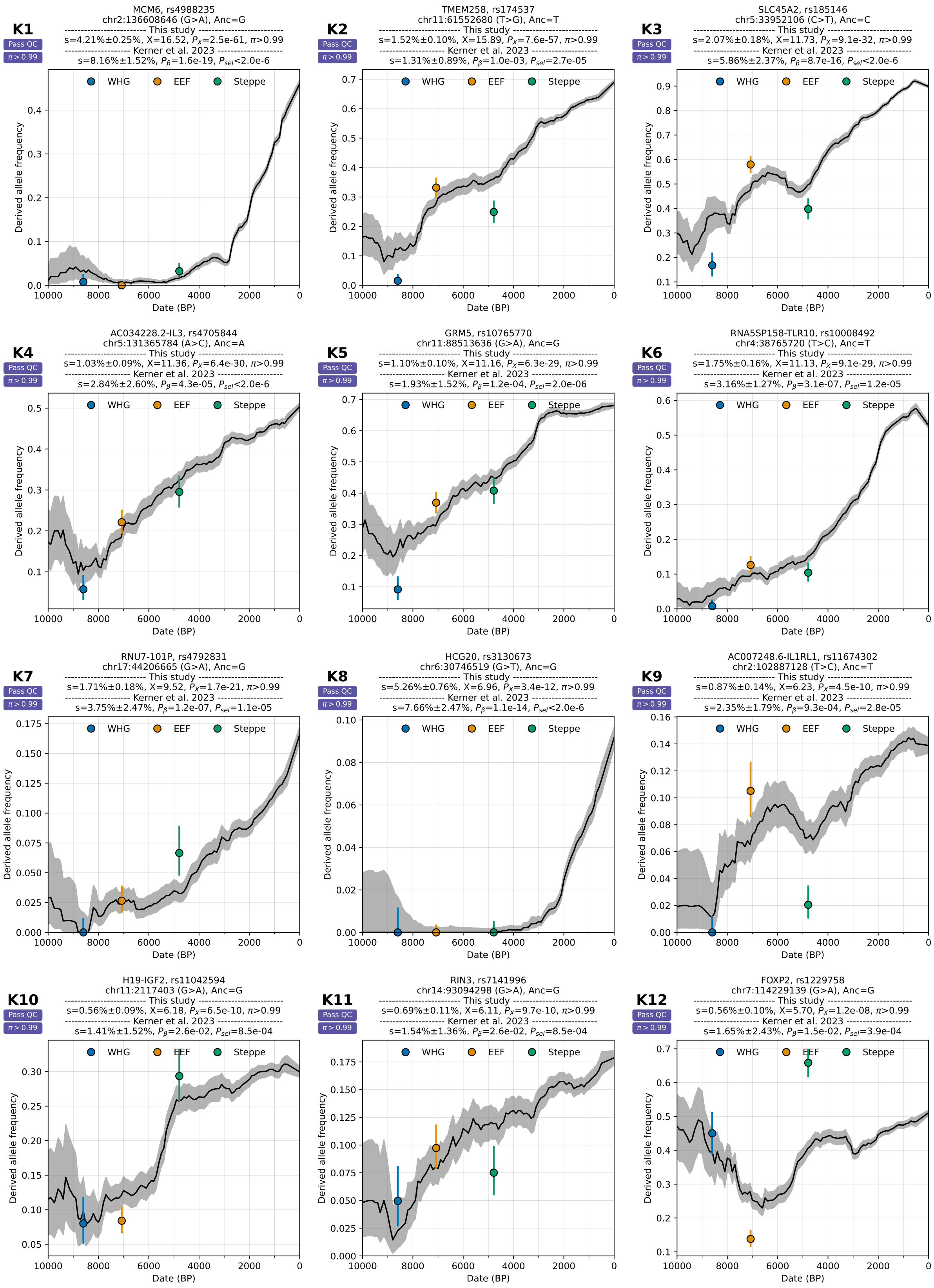

Supplementary Figure S6.11: Positive selection cases from Kerner et al. 2023 that passed QC.

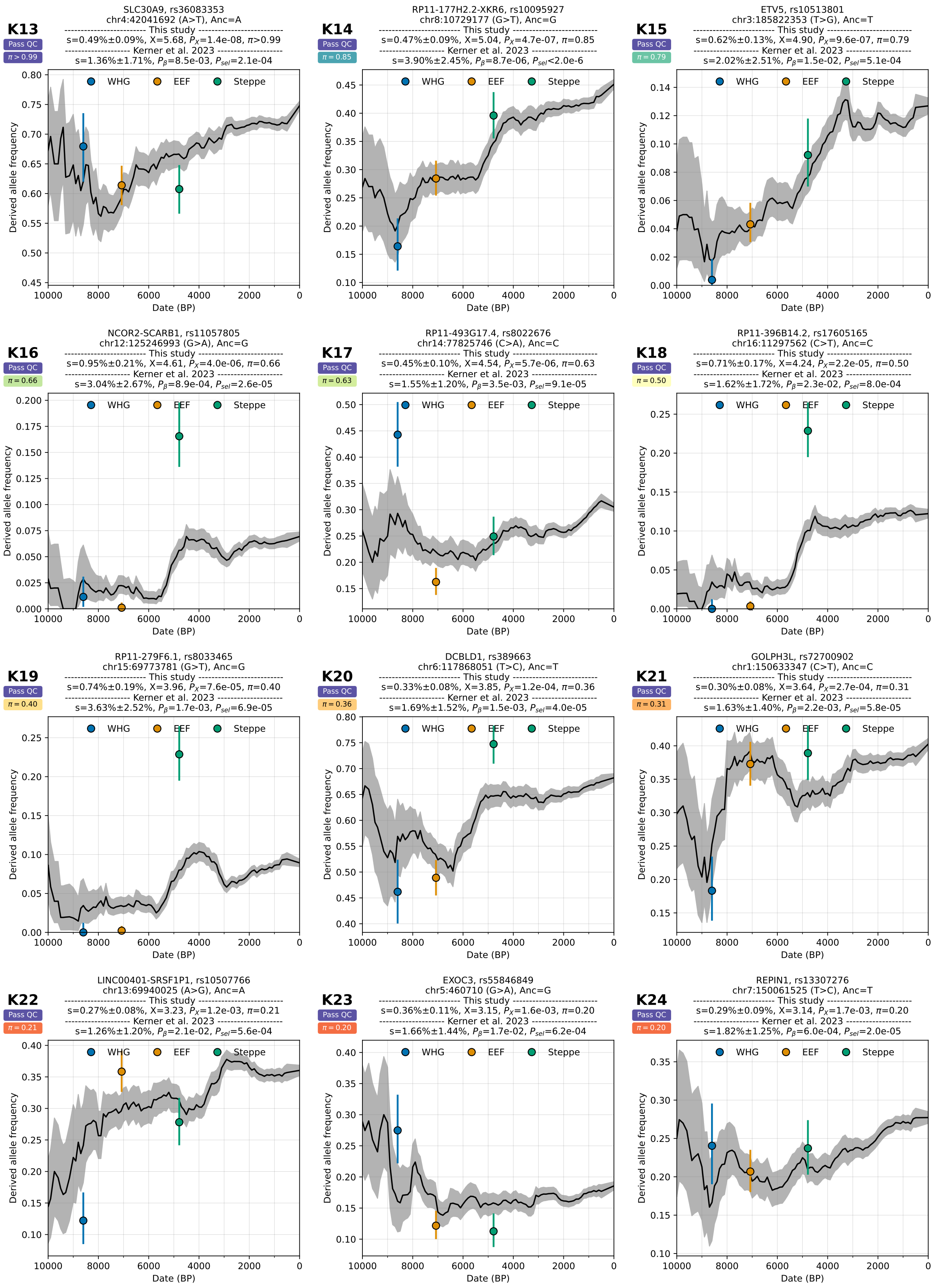

Supplementary Figure S6.12: Positive selection cases from Kerner et al. 2023 that passed QC.

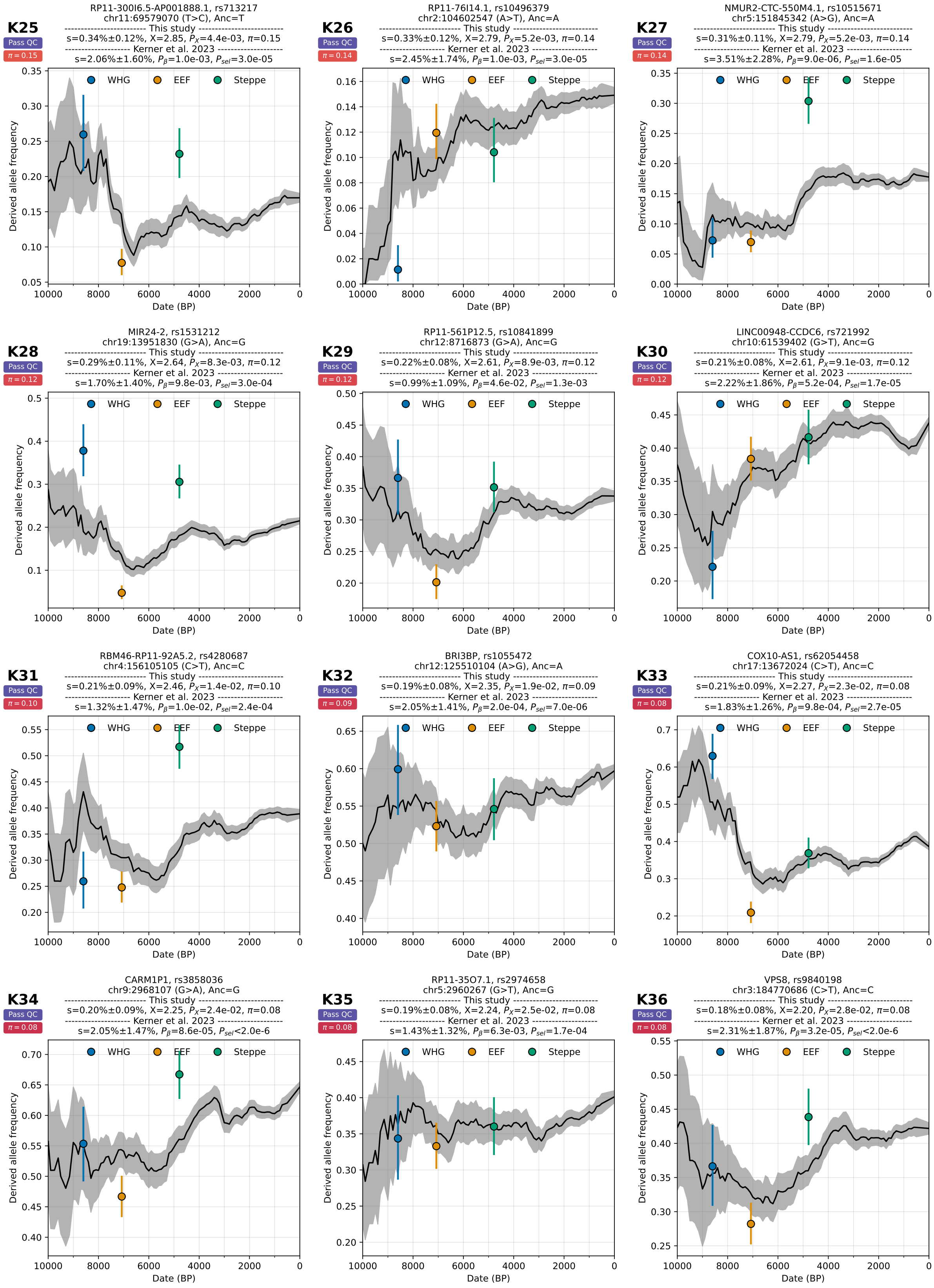

Supplementary Figure S6.13: Positive selection cases from Kerner et al. 2023 that passed QC.

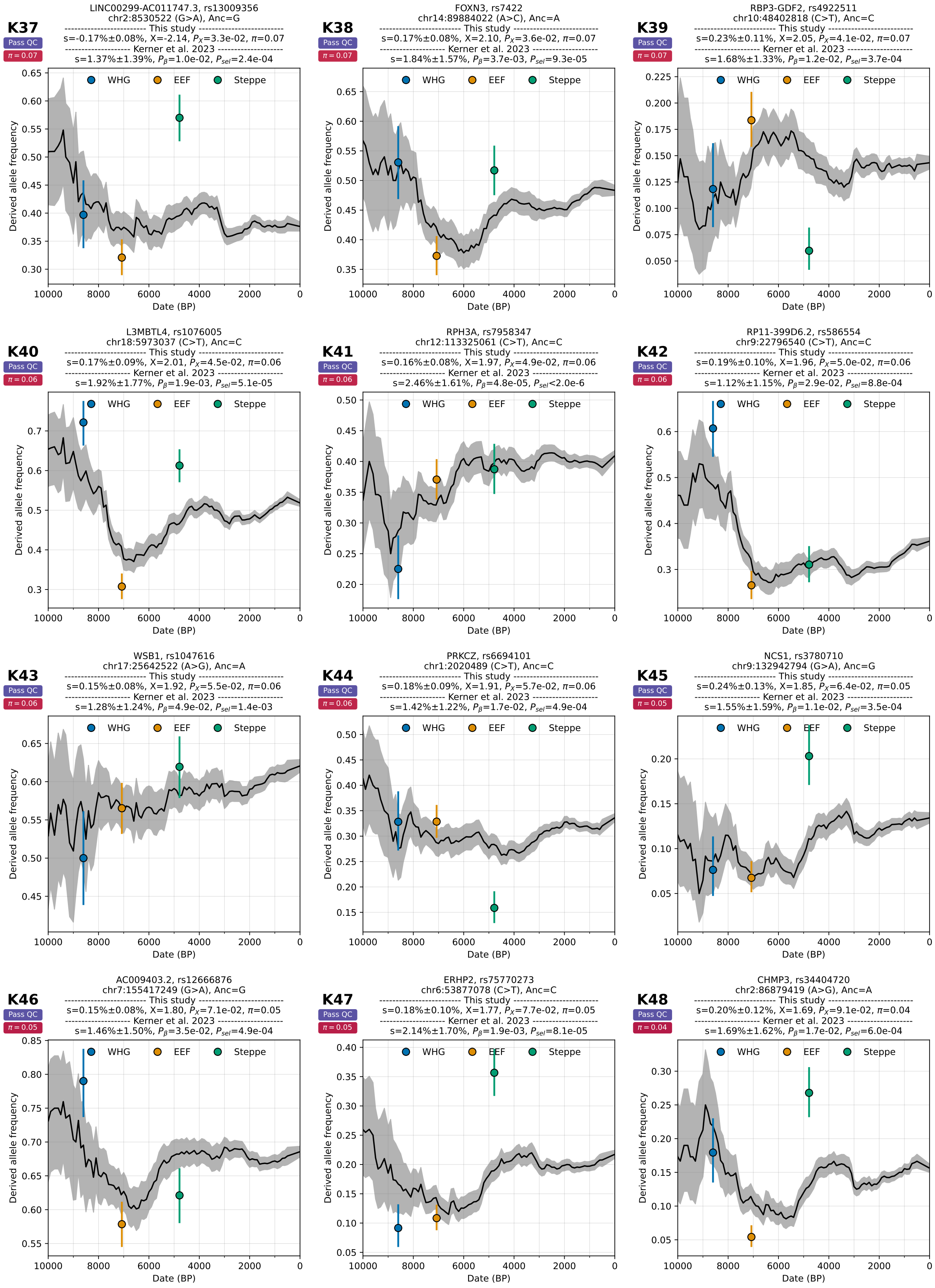

Supplementary Figure S6.14: Positive selection cases from Kerner et al. 2023 that passed QC.

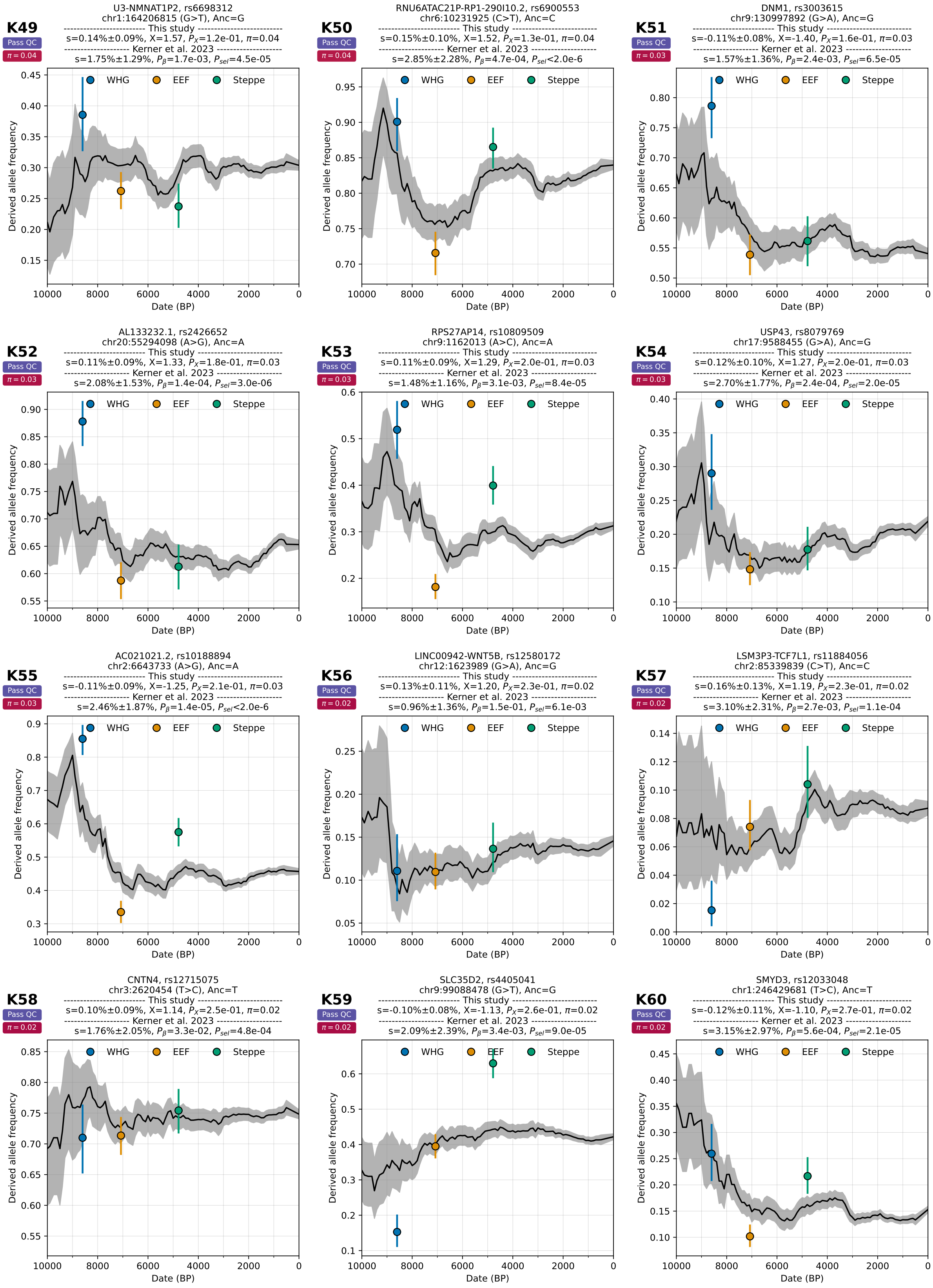

Supplementary Figure S6.15: Positive selection cases from Kerner et al. 2023 that passed QC.

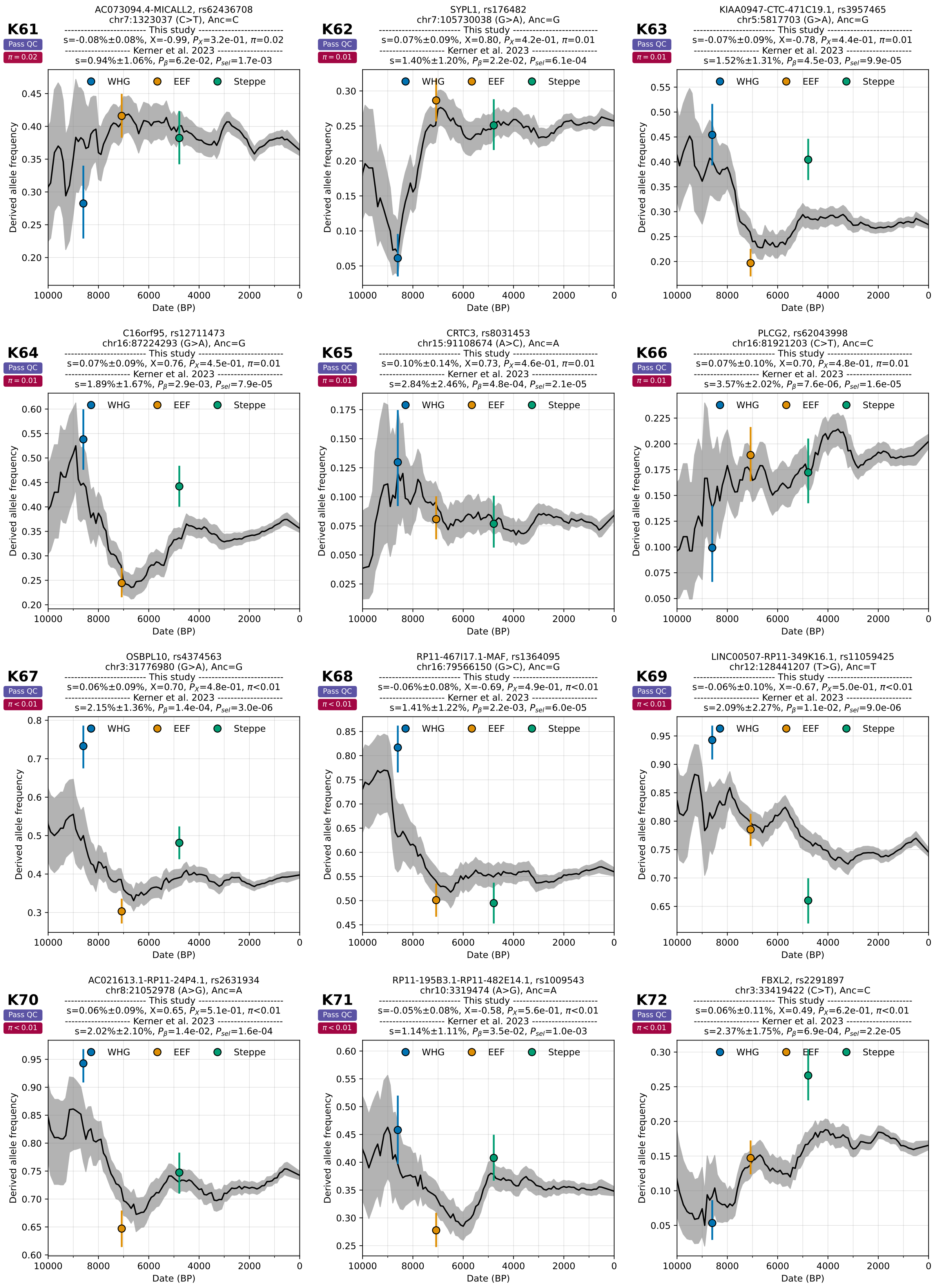

Supplementary Figure S6.16: Positive selection cases from Kerner et al. 2023 that passed QC.

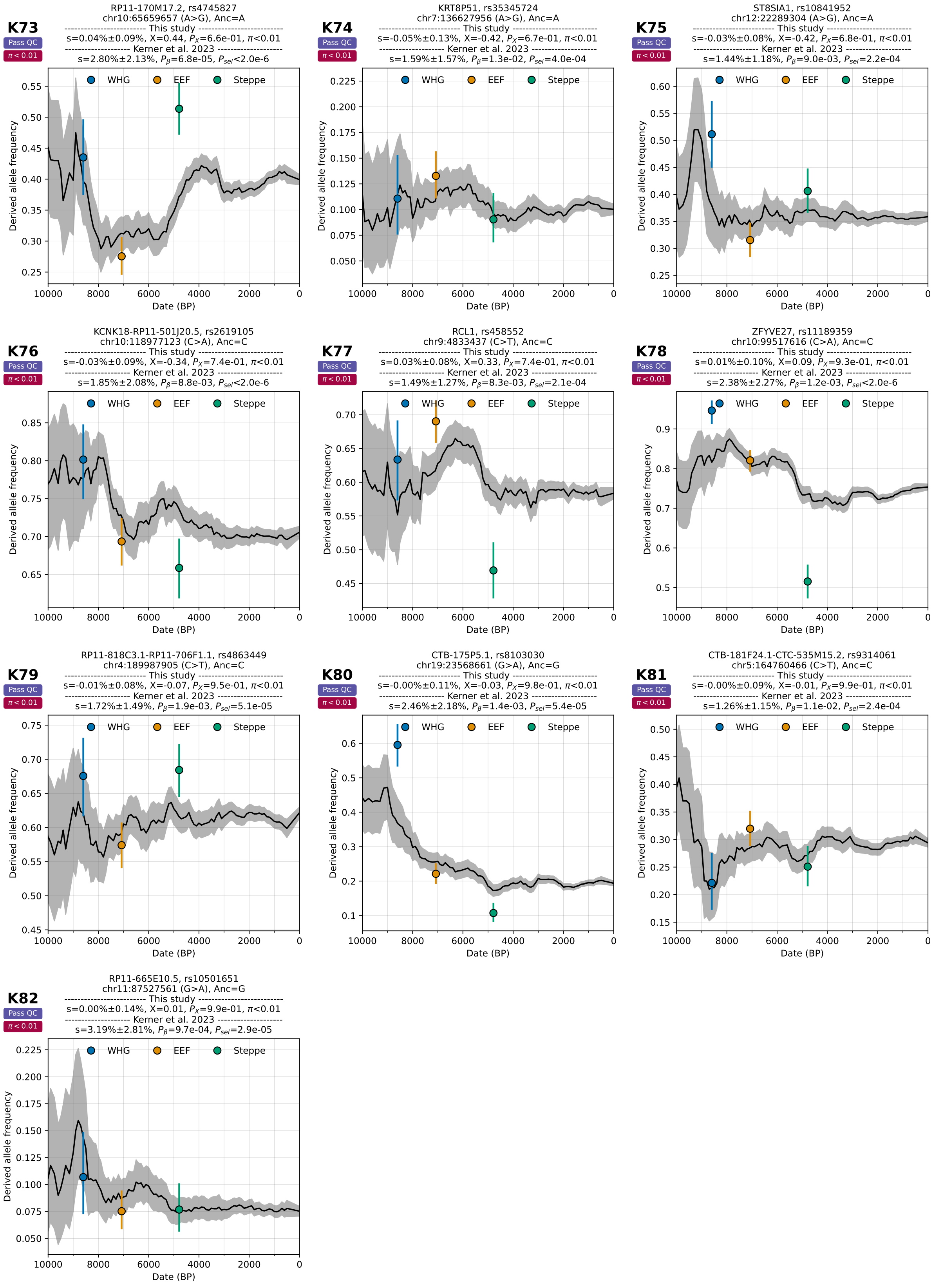

Supplementary Figure S6.17: Positive selection cases from Kerner et al. 2023 that passed QC.

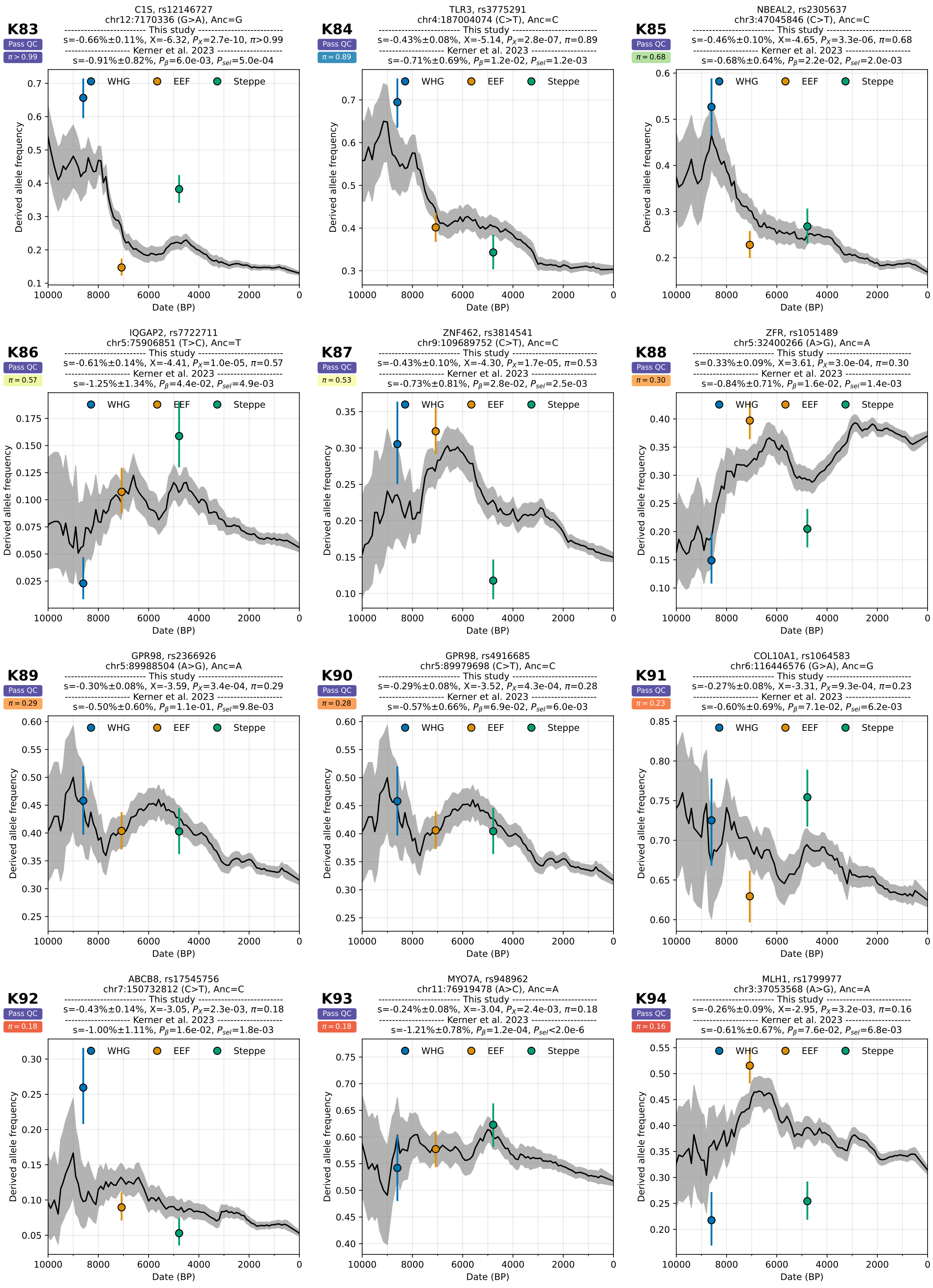

Supplementary Figure S6.18: Negative selection cases from Kerner et al. 2023 that passed QC.

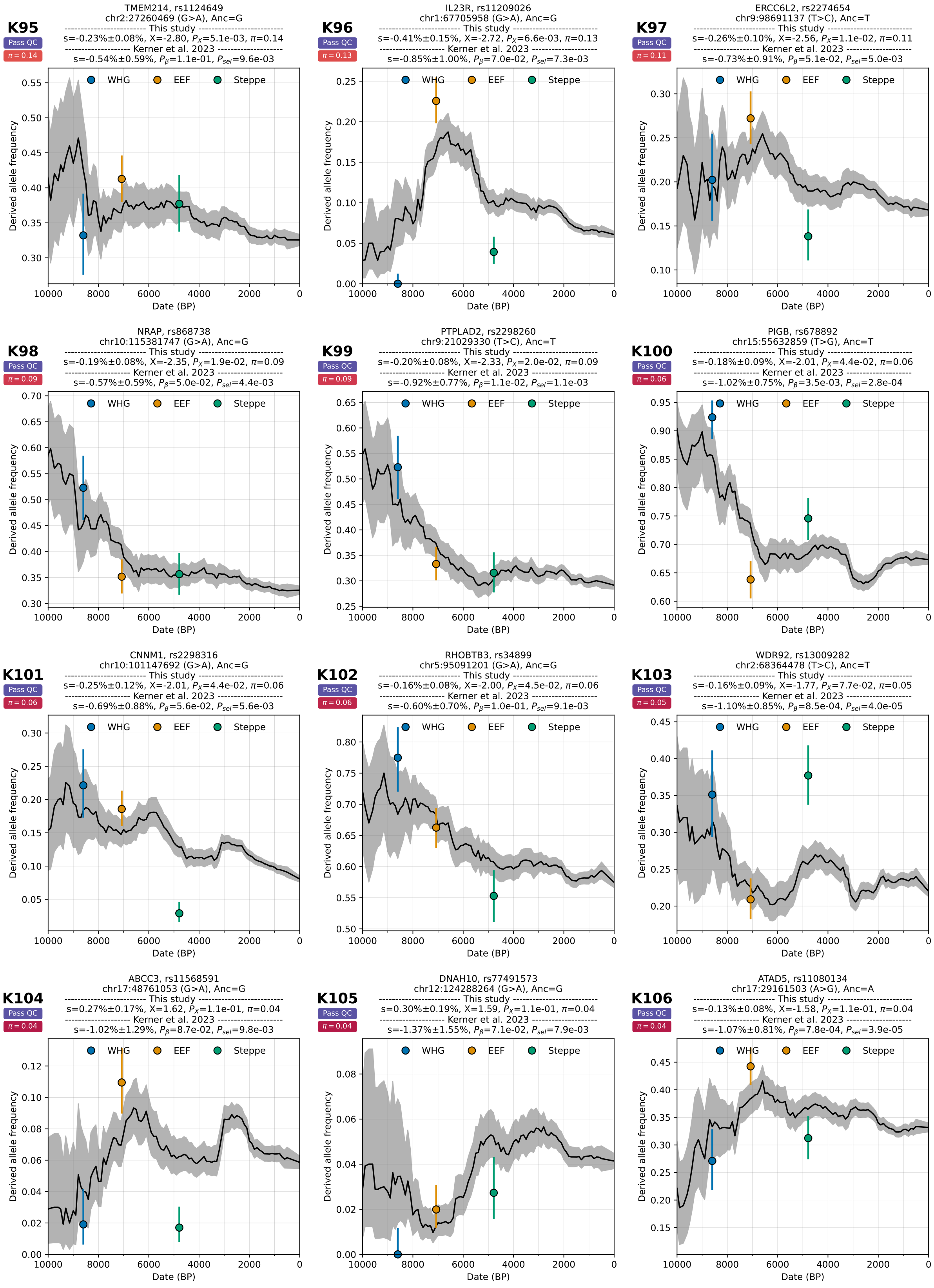

Supplementary Figure S6.19: Negative selection cases from Kerner et al. 2023 that passed QC.

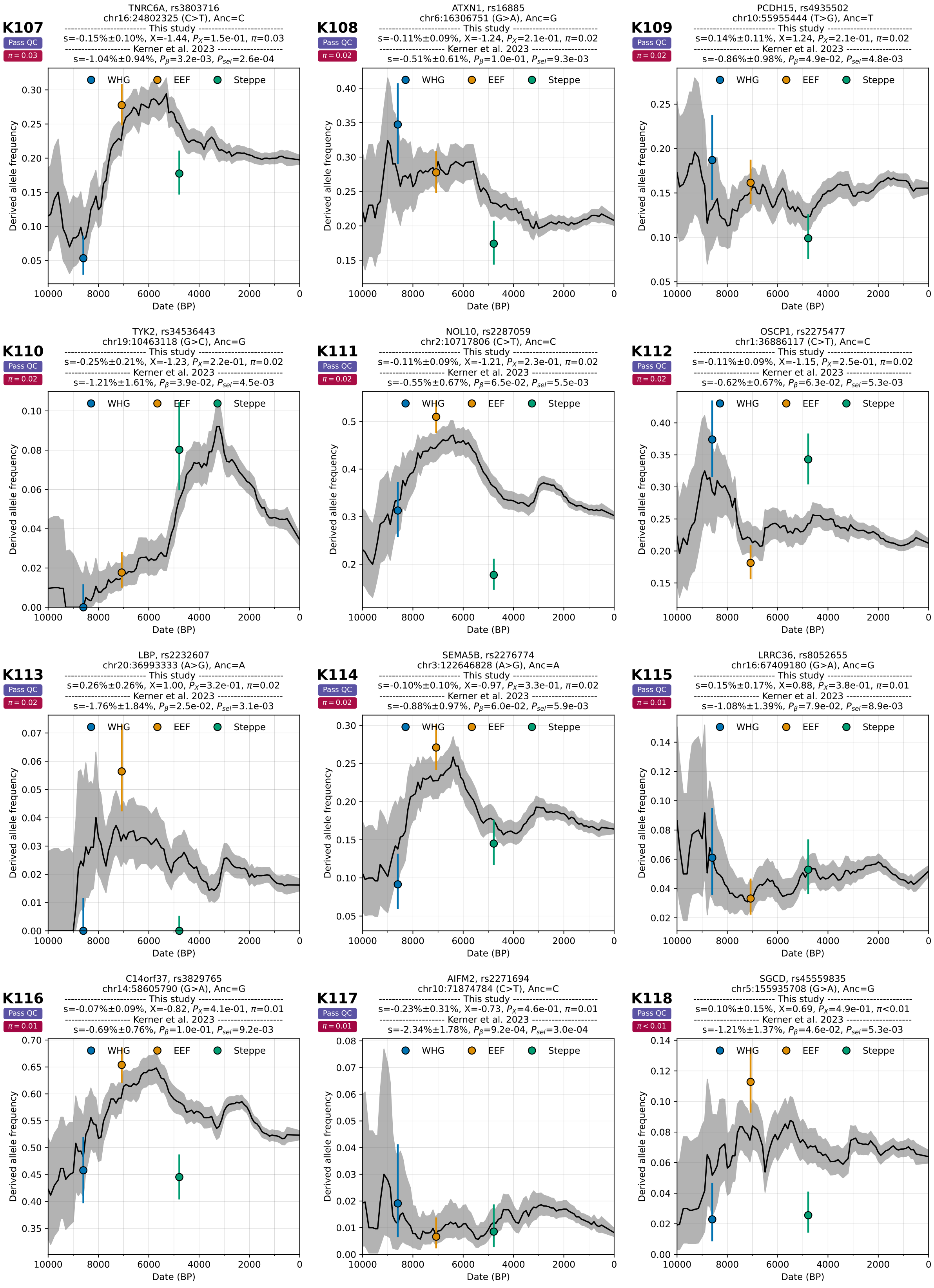

Supplementary Figure S6.20: Negative selection cases from Kerner et al. 2023 that passed QC.

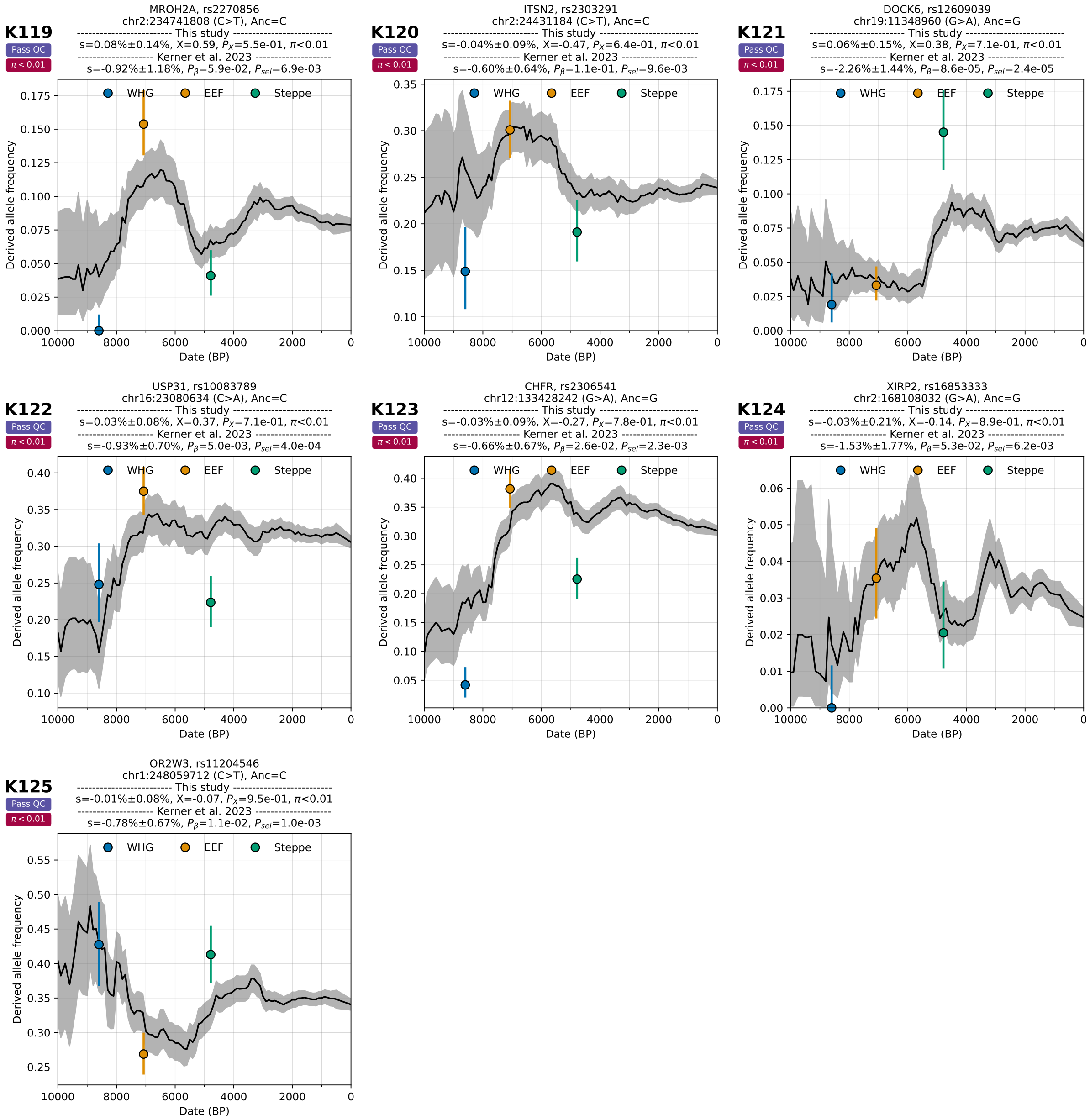

Supplementary Figure S6.21: Negative selection cases from Kerner et al. 2023 that passed QC.

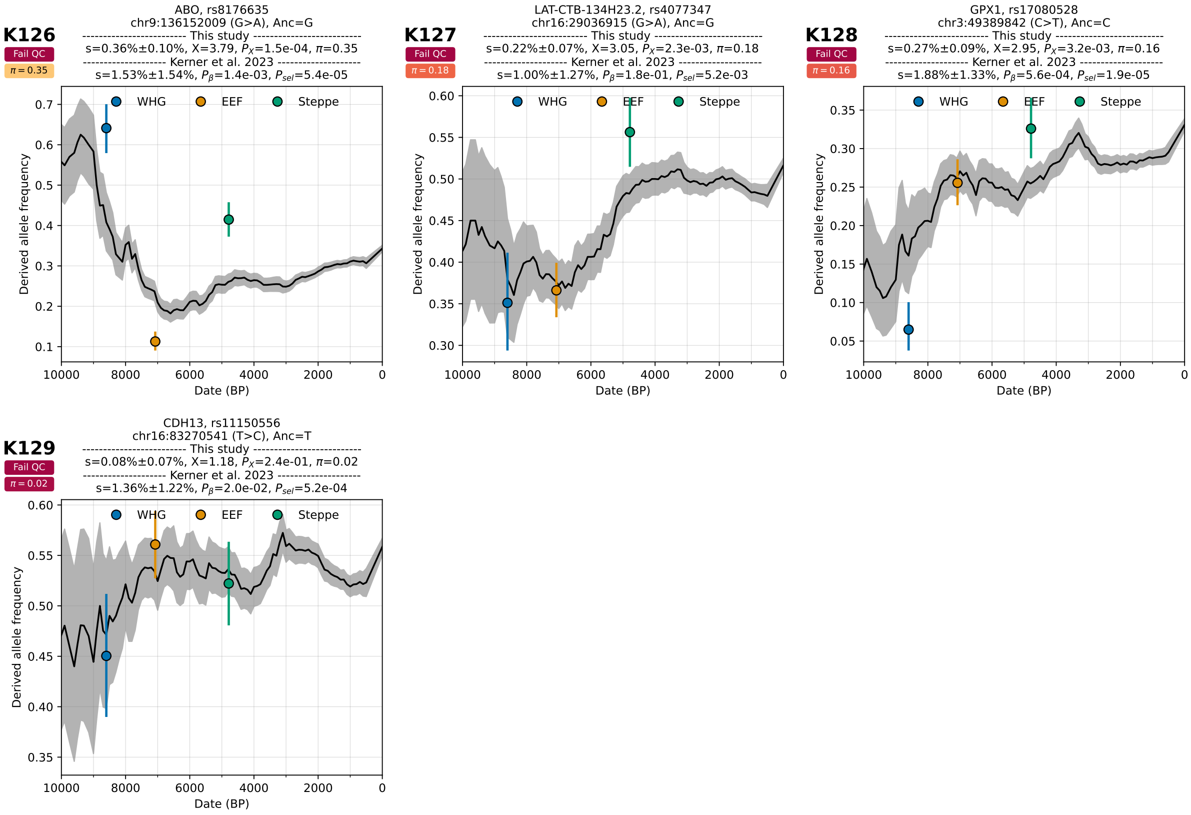

Supplementary Figure S6.22: Positive selection cases from Kerner et al. 2023 that failed QC.

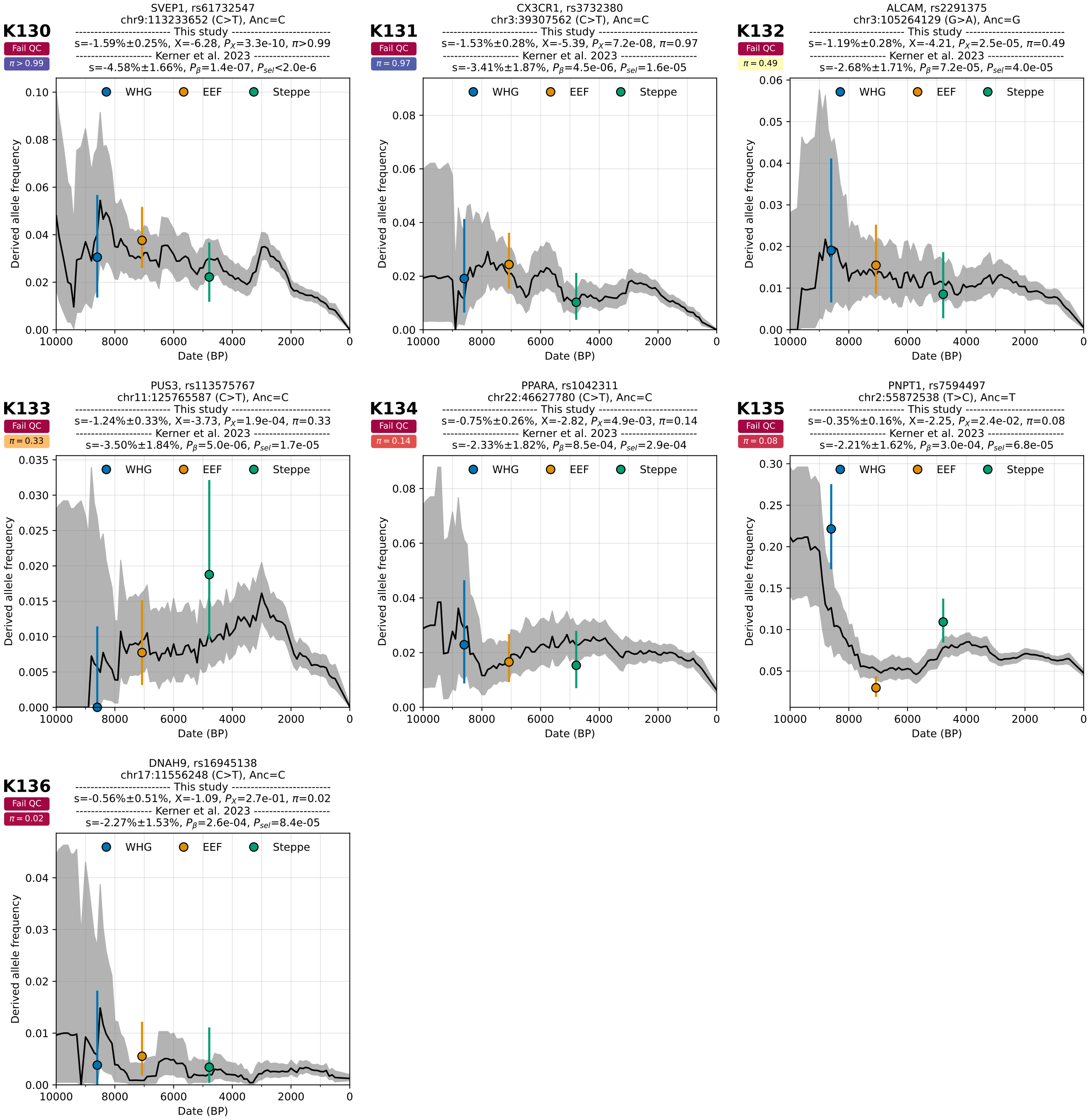

Supplementary Figure S6.23: Negative selection cases from Kerner et al. 2023 that failed QC.

Re-evaluation of results from Irving-Pease et al. 2024

We evaluated 21 selection signals from Figure 2a of Irving-Pease et al. 2024[7] and extracted summary statistics from Table S2.1.4 of that study. That table includes five sets of estimated selection coefficients for different ancestry categories: pan-ancestry analysis (ALL), Western hunter-gatherers (WHG), Eastern hunter-gatherers (EHG), Caucasus hunter-gatherers (CHG), and Anatolian farmers (ANA). For each SNP, we picked Z_{Top} as the Z score with the most significant value across these categories. In Figure S6.24, we compare Z_{Top} and X scores for the full time transect analysis. We evaluated five SNPs with the largest Z_{Top} for each locus (Figure S6.24). We picked these five SNPs for each locus because they are highlighted in the Extended Data Figures 1-10 and Supplementary Figures S56-S76, all from Irving-Pease et al. 2024. Re-evaluation of these SNPs is shown in our Figures S6.25-S6.29.

In interpreting our re-evaluation of the results from Irving-Pease et al. 2024, it is important to be cognizant of the fact that for each locus, we selected the SNP with the most significant signal of selection in our study out of five candidate SNPs per locus proposed by Irving-Pease et al. While this approach lacks control for multiple testing and thus does not provide an entirely fair comparison with the other four studies re-evaluated here—it is expected to overestimate the replication rate in Irving-Pease et al. 2024 relative to those other studies—we followed this approach to maintain consistency with the approach of Irving-Pease et al. 2024.

Of the 21 candidate loci identified by Irving-Pease et al. 2024, our analysis found that 14 had at least one SNP among five candidates per locus with significant posterior probability ($\pi > 99\%$). These loci include *MCM6* (peak 3), *CCDC12* (peak 4), *RNA5SP158* (peak 5), *SLC45A2* (peak 7), *IRF1* (peak 8), *SLC34A1* (peak 9), HLA (peak 10), *ABO* (peak 13), *FADS2* (peak 14), *ACAD10* (peak 15), *CYP1A1* (peak 16), *RAPGEFL1* (peak 18), *ARL17B* (peak 19), and *CENPM* (peak 21).

Additionally, *AFF3* (peak 2, SNP 2, $\pi = 82\%$), *TNKS* (peak 11, SNP 3, $\pi = 71\%$), and *WWP2* (peak 17, SNP 2, $\pi = 61\%$) showed some evidence of selection on at least one of the five candidate SNPs per locus passing QC.

For the remaining four loci—*RP11-415K20.1* (peak 1, SNP 1, $\pi = 21\%$), *KRT18P51* (peak 6, SNP 4, $\pi = 27\%$), *CTD-2008O4.1* (peak 12, SNP 5, $\pi = 25\%$), and *CTC-258N23.3* (peak 20, SNP 4, $\pi = 5\%$)—there is no strong evidence of selection, as all five SNPs per locus that passed QC had posterior probabilities below 27%.

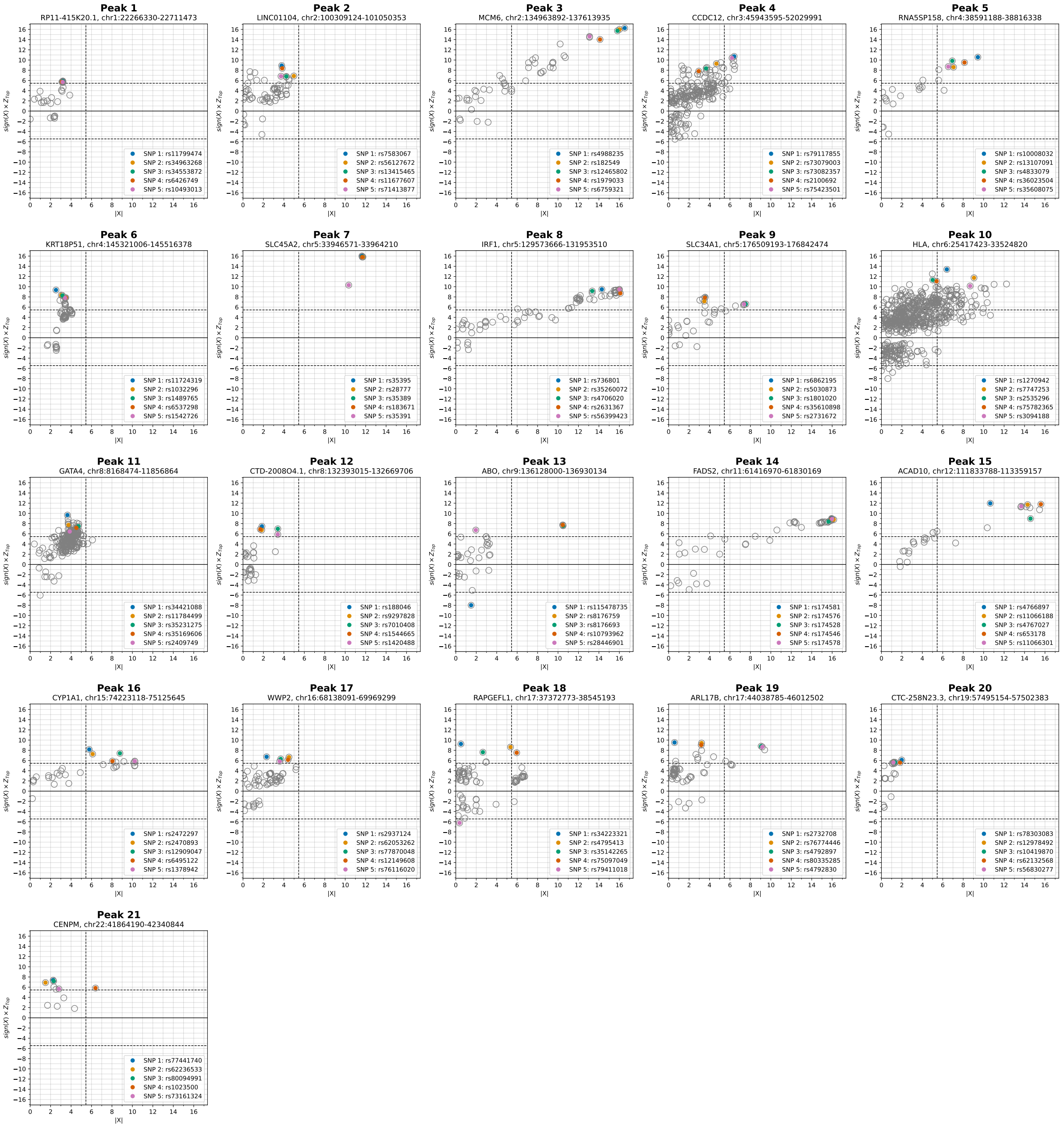

Supplementary Figure S6.24: Re-evaluating 21 candidate selective sweeps from Irving-Pease et al. (2024). Each marker represents a SNP, with the x-axis showing the absolute value of the X-score ($|X|$) from the current study. Irving-Pease et al. reported five sets of estimated selection coefficients for different ancestry categories: ALL, WHG, EHG, CHG, and ANA. For each SNP, we selected Z_{Top} (y-axis), the most significant Z score across these five categories. Then, we evaluated the five SNPs with the largest Z_{Top} for each locus, represented by markers in different colors, as shown in the legend of each panel. We picked these five SNPs for each locus because they are highlighted in the Extended Data Figures 1–10 and Supplementary Figures S56–S76, all from Irving-Pease et al. (2024). The y-axis is polarized using the sign of the X-statistic.

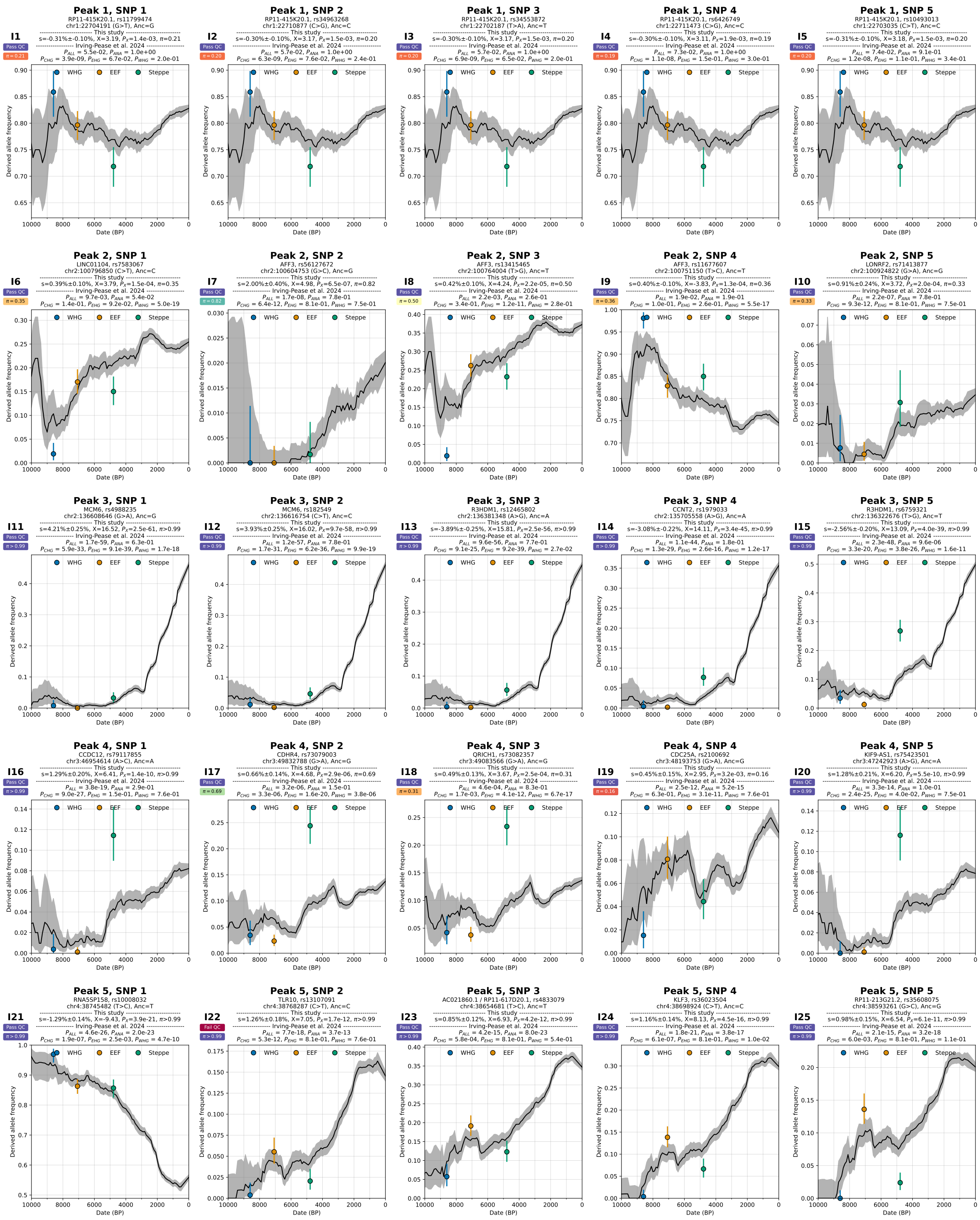

Supplementary Figure S6.25: Re-evaluating 21 candidate sweeps from Irving-Pease et al. 2024.

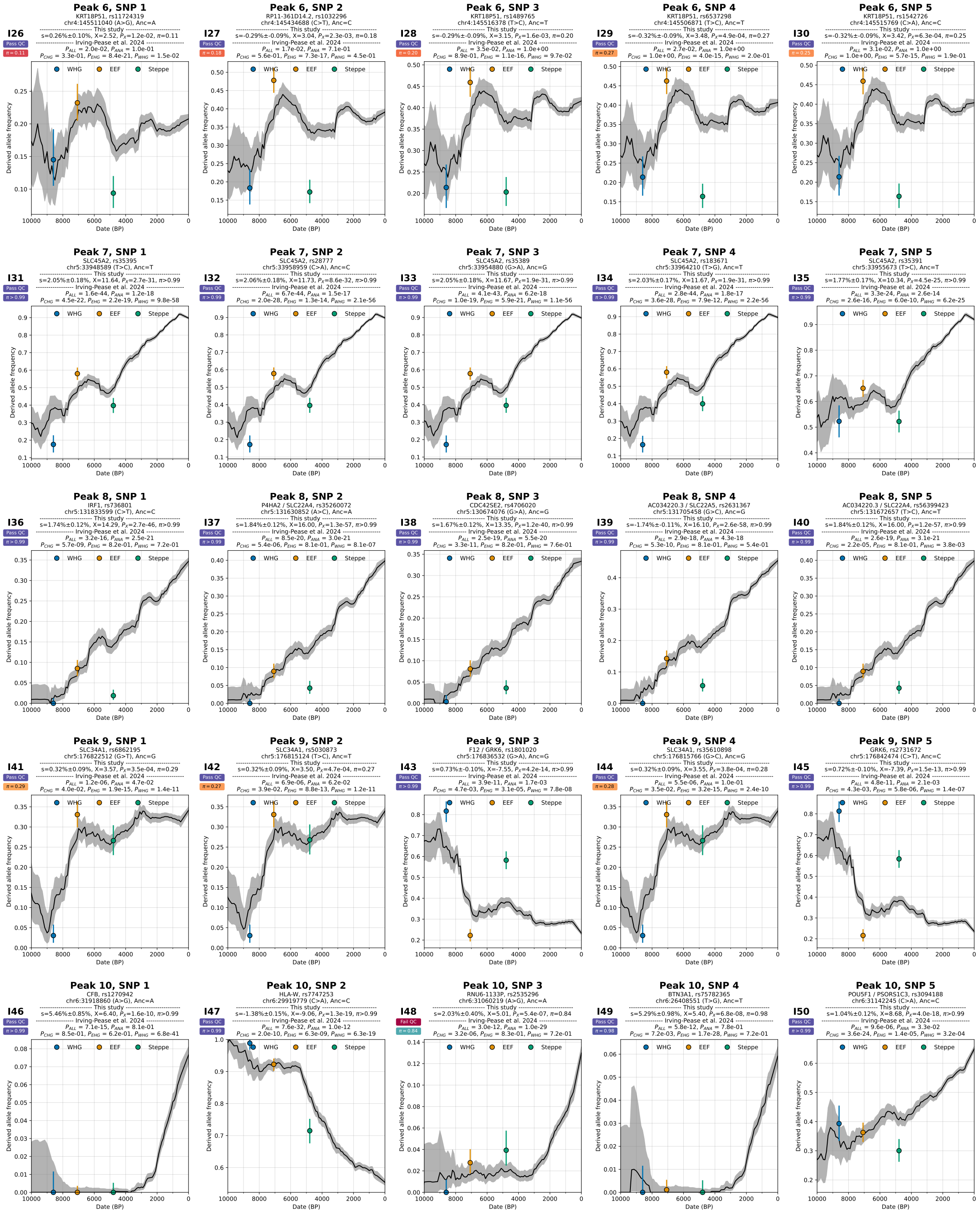

Supplementary Figure S6.26: Re-evaluating 21 candidate sweeps from Irving-Pease et al. 2024.

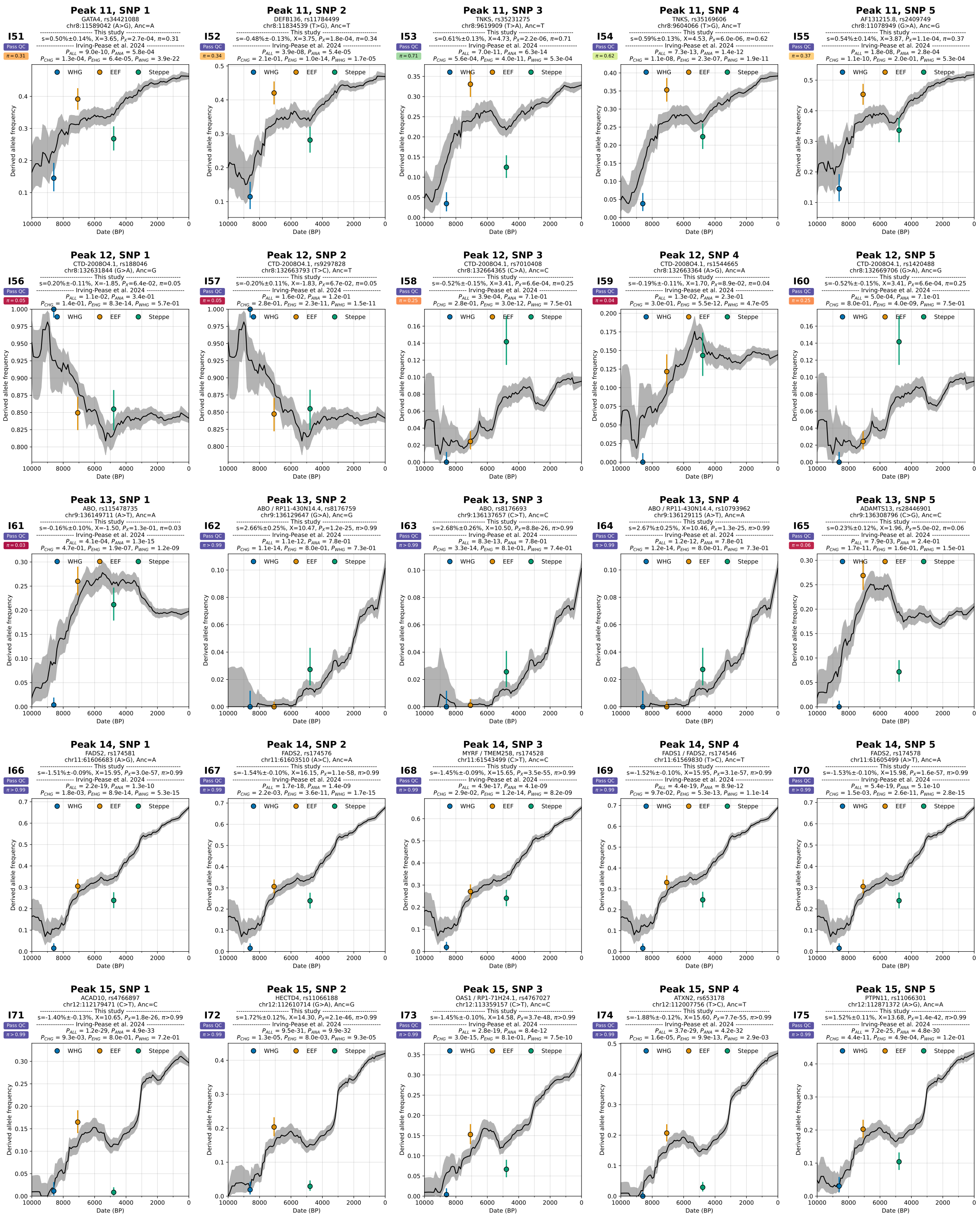

Supplementary Figure S6.27: Re-evaluating 21 candidate sweeps from Irving-Pease et al. 2024.

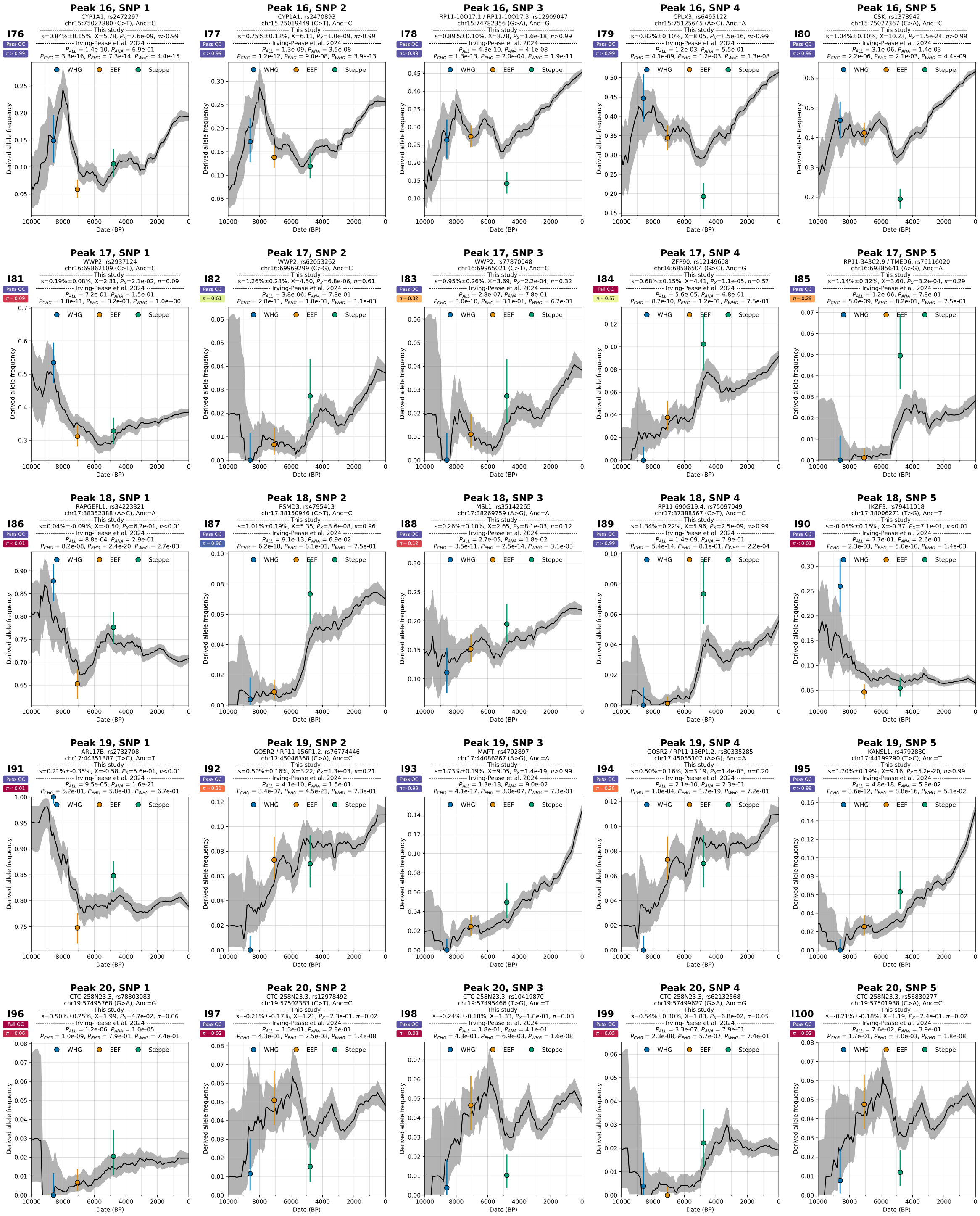

Supplementary Figure S6.28: Re-evaluating 21 candidate sweeps from Irving-Pease et al. 2024.

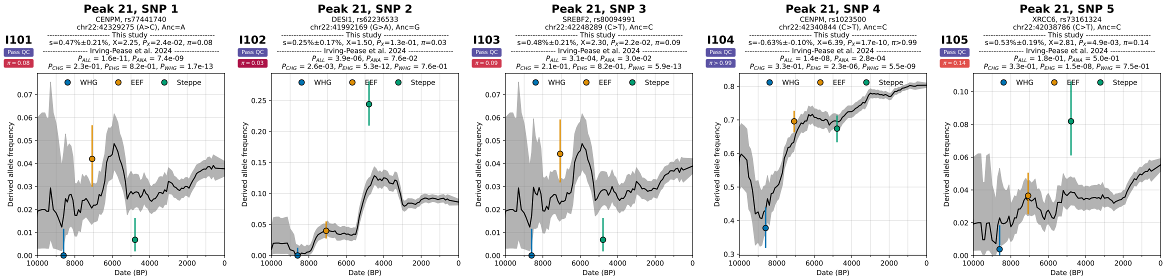

Supplementary Figure S6.29: Re-evaluating 21 candidate sweeps from Irving-Pease et al. 2024.

Supplementary Information section 7

A new picture of selection at the major risk factor for multiple sclerosis (MS)

Barrie et al. 2024[50] reported positive selection at the HLA-DRB1*15:01 allele tagged by the rs3135388 (G>A) variant, the strongest known genetic risk factor for multiple sclerosis (MS), and proposed that the elevated genetic risk for MS in Northern Europeans relative to other populations owes its origins at least in part due to the very high steppe pastoralist ancestry proportion in these populations. We confirm a strong signal of positive selection at this locus. However, we also identify features of the selection history at the locus missed by the previous study, and that together paint a qualitatively different story. The selection history was more complicated, and steppe ancestry was not in fact the main driver of the variant's frequency differences across Europe today.

First, we detect a period of strong negative selection from ~2000 years ago to the present (s = -2.1%, π>99%) (Figure 3). This period of negative selection has had a primary influence on the frequency of this variant in Europeans, was missed in Barrie et al. 2024, and followed the period of positive selection from ~6000 to ~2000 years ago (s = 4.2%, π>99%) that drove their finding.

Second, we show that the rise in frequency of this variant occurred initially in people without steppe ancestry living south of the Caucasus mountains, prior to the period of positive selection in Yamnaya steppe pastoralists around 5000 years ago that was the focus of the Barrie et al. 2024 study (Figure S7.1). We infer that the variant's frequency was around 9% (4.9%-13.8%; 95% confidence interval) 7000-5000 years ago south of the Caucasus mountains. Our finding of a first rise in frequency in association with Caucasus ancestry is entirely consistent with Figure 5c of Barrie et al. 2024 which infers that that the variant rose in frequency on a Caucasus ancestry background, one of the primary components of the ancestry of Yamnaya steppe pastoralists. However, our results go beyond that earlier study in showing that the rise in frequency is actual likely to have occurred south of the Caucasus mountains (Figures S7.1-S7.3), not in Eneolithic steppe hunter-gatherers in this period who also carried Caucasus ancestry.

Third, Barrie et al. 2024 observed that the frequency of the MS risk allele is highest among modern individuals in northern Europe with high steppe ancestry. They proposed that the steppe ancestry gradient, combined with environmental factors modulating genetic risk independent of genetics, address the long-standing debate regarding the north-south gradient in MS prevalence. However, our data reveal that the selection coefficient for this variant varies in both space and time, with the intensity of selection highest in northern populations compared to southern ones (Figure S7.3). For example, for time transects older than 3500 years ago, the selection coefficient in the northern region (N; 11.1 ± 2.5% s.d.) is approximately two times higher than that in the southwest region (SW; 6.1 ± 2.1% s.d.). The correlation between steppe ancestry and allele frequency in modern individuals is thus geographically confounded: the difference in selective pressure between the north and south after the spread of steppe pastoralists, not steppe ancestry, is consistent with driving the observed north-south gradient of this allele (Figures S7.1-S7.3).

qpAdm modeling

We used qpAdm[51,52] (v1700) to estimate ancestry proportions for each sample.

We applied a 4-way model based on the Fernandes et al. 2020[53], using the following populations:

Right: Mbuti.DG, Ust_Ishim, ElMiron, Vestonice16, MA1, Israel_Natufian, Jordan_PPNB, Russia_Samara_EBA_Yamnaya, Morocco_LN.SG

Left: Turkey_N, EHG, WHG, Iran_GanjDareh_N

We also applied a 3-way model from Patterson et al. 2022[54], using the following populations:

Right: OldAfrica, WHGB, Russia_Afanasievo, Turkey_N

Left: WHGA, Balkan_N, OldSteppe

Inference of allele frequency in ancestral populations

We estimate allele frequencies in the ancestral populations by maximizing the likelihood, incorporating the estimated qpAdm ancestry proportions and imputed genotypes of all individuals.

Assume a 4-way model with unknown allele frequencies p_A, p_B, p_C and p_D for the source populations A, B, C, and D, respectively. For each individual i, the latent allele frequency $\hat{p}_i$ is calculated as a weighted average based on ancestry proportions $q_{A,i}$, $q_{B,i}$, $q_{C,i}$, and $q_{D,i}$:

$$\hat{p}_i = q_{A,i}p_A + q_{B,i}p_B + q_{C,i}p_C + q_{D,i}p_D$$

The likelihood of observing the genotype g_i for each individual is then computed as:

$$P(g_i = 0 \mid \hat{p}_i) = (1 - \hat{p}_i)^2, \quad P(g_i = 1 \mid \hat{p}_i) = 2\hat{p}_i(1 - \hat{p}_i), \quad P(g_i = 2 \mid \hat{p}_i) = \hat{p}_i^2$$

The likelihood across all individuals is maximized to estimate allele frequencies in the sources.

$$L(\hat{p}_1, \hat{p}_2, \dots, \hat{p}_n) = \prod_{i=1}^{n} P(g_i \mid \hat{p}_i)$$

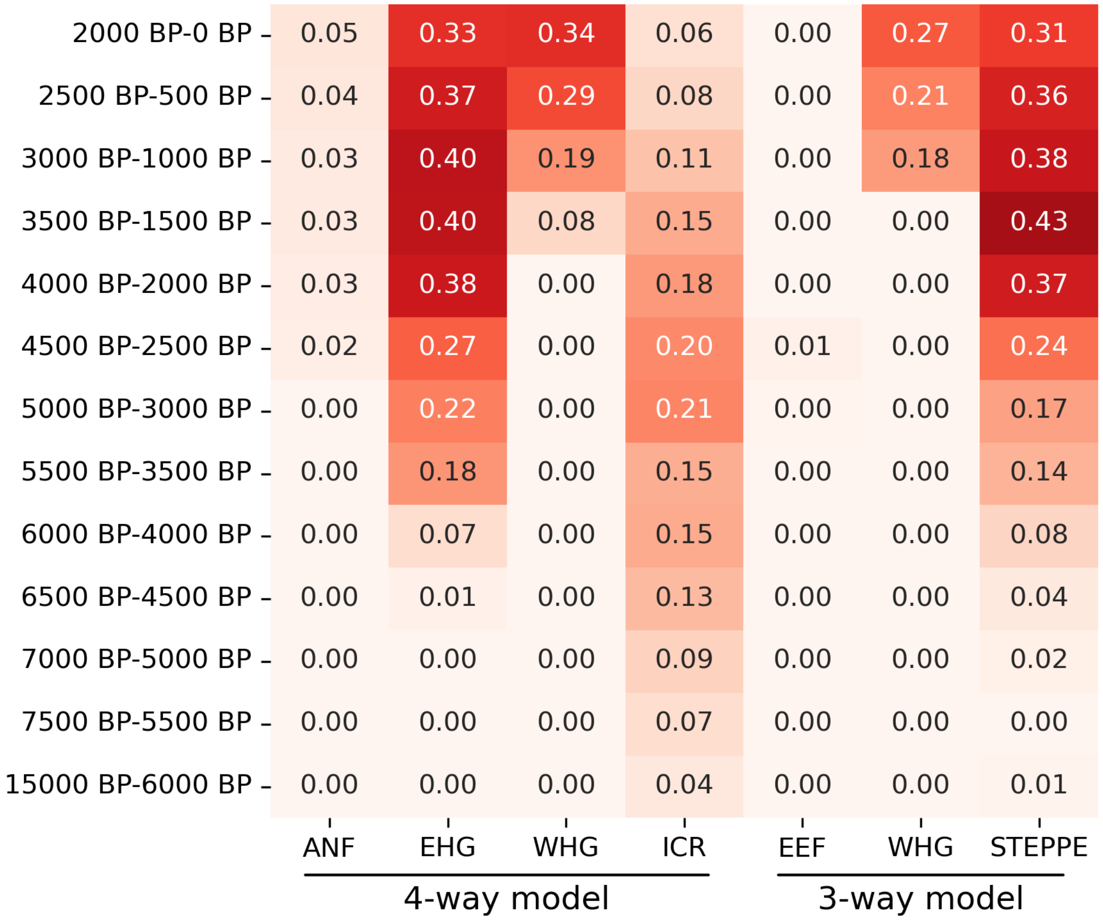

Figure S7.1: Maximum likelihood estimation of allele frequencies of rs3135388 (G>A) across different time transects for ancestral populations using the 4-way and 3-way qpAdm ancestry models: ANF (Anatolian neolithic farmer), WHG (Western hunter-gatherer), ICR (Iranian/Caucasian-related), EHG (Eastern hunter-gatherer), EEF (Early European farmer), and STEPPE (Steppe pastoralists). For each qpAdm model, only individuals with a model P-value greater than 0.05 were used for the maximum likelihood estimation of allele frequencies.

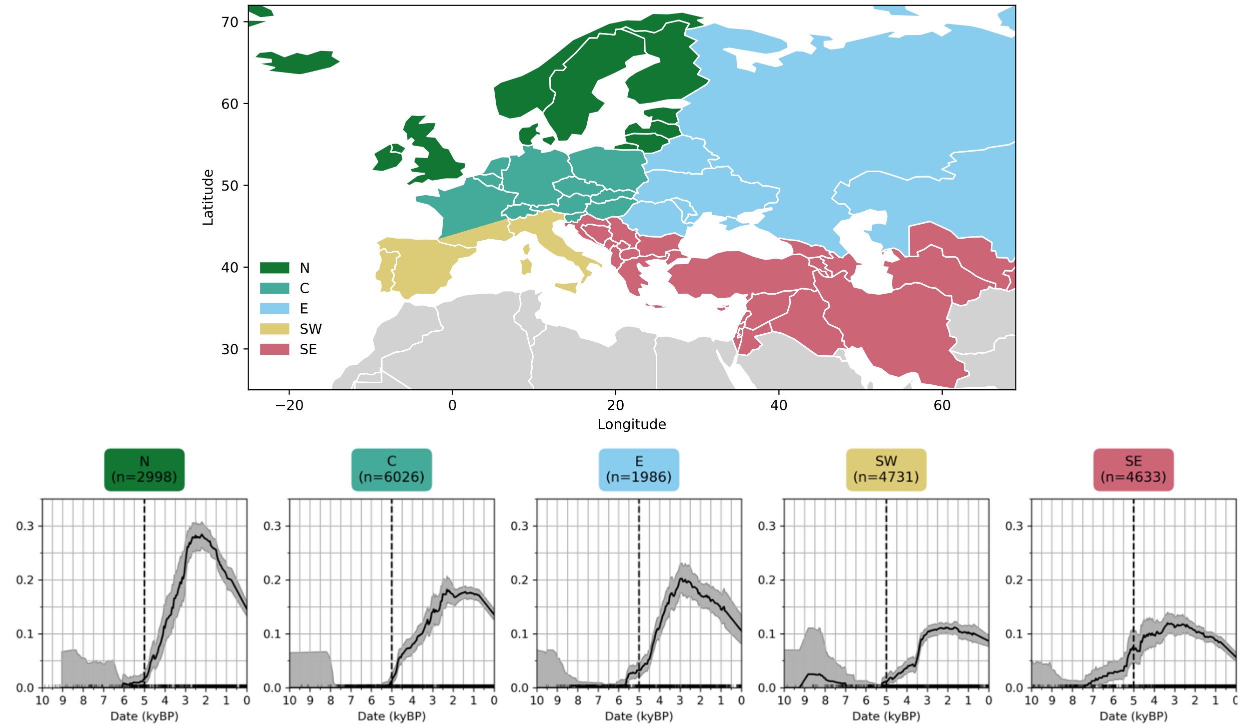

Figure S7.2: Allele frequency trajectory of rs3135388 (G>A) stratified by five geographic regions: N (Northern), C (Central), E (Eastern), SW (Southwest), and SE (Southeast).

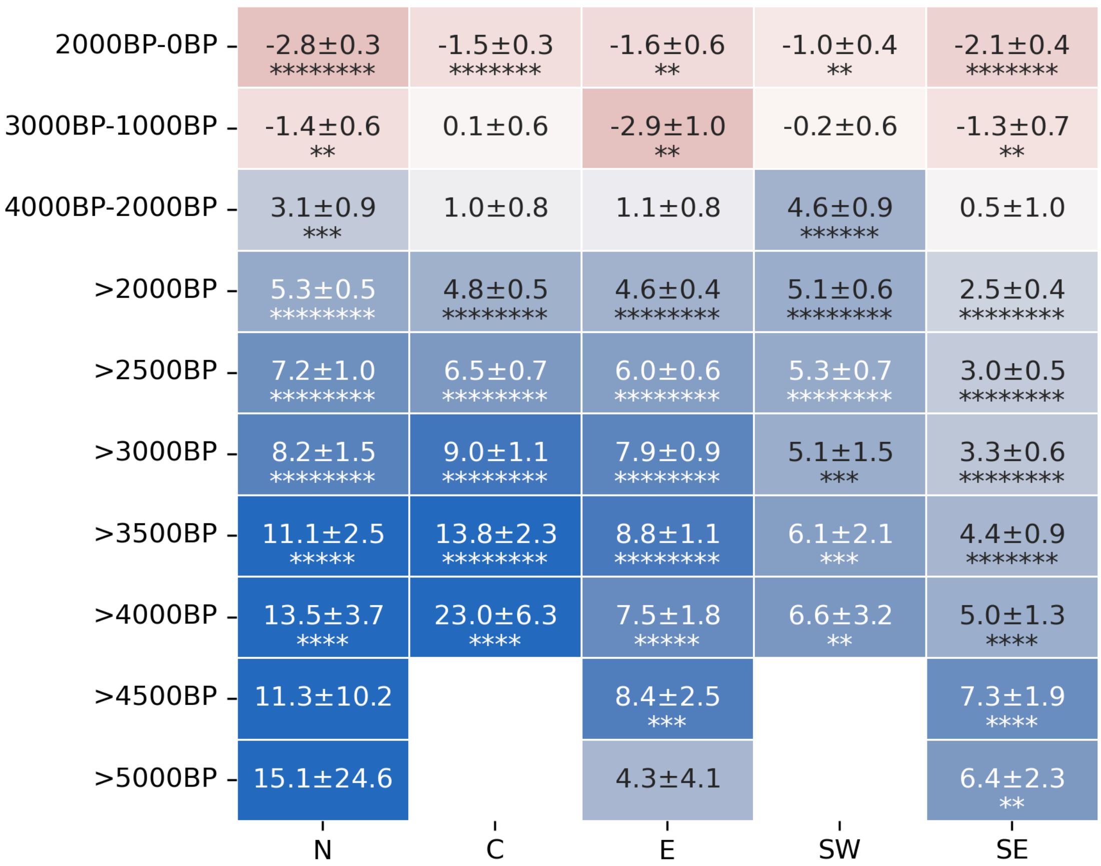

Figure S7.3: Selection coefficient of rs3135388 (G>A) across different time transects, stratified by five geographic regions: N (Northern), C (Central), E (Eastern), SW (Southwest), and SE (Southeast). Selection coefficient values are presented as percentages in the format (s ± s.d.). Each star represents the level of significance. The number of stars (n stars) indicates that the P value is less than 0.5×10^{-n}, while the absence of a star means the P value is greater than 0.05.

Supplementary Information section 8
A fast GLMM implementation - PQLseqPy

To analyze on the order of few thousands individuals in a more time-efficient manner, we re-implemented the PQLseq[55] algorithm in Python. This provides a faster implementation of a Generalized Linear Mixed Model (GLMM) with a logit link function and binomial family distribution, with added flexibility and significant performance improvements. Key features include: (1) Ability to calculate the null model (only intercept as a covariate); (2) Support for predefined variance components (fixed_tau argument); (3) orders-of-magnitude speedup compared to the original PQLseq implementation (Figure S8.1), and (4) improved numerical stability (regularization_factor argument).

We applied several programming techniques to improve computational speed. Specifically, we re-implemented the core algorithm entirely in efficient Python code. The original PQLseq used C++ for part of its algorithm but alternated between R and C++ at each iteration of numerical optimization, causing repetitive type casting and object duplication. By implementing the full core algorithm in Python, we avoided these issues and significantly reduced runtime. We refer to this faster extension of PQLseq as PQLseqPy.

To evaluate performance, we carried out a head-to-head comparison of computational speed and parameter estimation between PQLseq, PQLseqPy, and the specialized version. We randomly selected genotype data from 1,000 SNPs in 2,000 ancient DNA samples, along with sample dates and the genetic relatedness matrix. Each method was applied to analyze the association between allele frequency and sample dates, one SNP at a time, and we compared average runtime across SNPs.

Parameter estimates, including the fixed effect β and its standard error, the heritability parameter h^2, and the total variance component σ^2, were consistent between PQLseq and PQLseqPy. On average, PQLseqPy was about 54 times faster than PQLseq. For the specific application of interest in this study, where h^2 almost always converged to 1, fixing h^2 to 1 using the tau2_set_to_zero argument yielded an 82-fold speed increase compared to PQLseq.

These results (Figure S8.1) highlight both the efficiency and the accuracy of the PQLseqPy implementation. PQLseqPy is freely available from https://github.com/mokar2001/PQLseqPy.

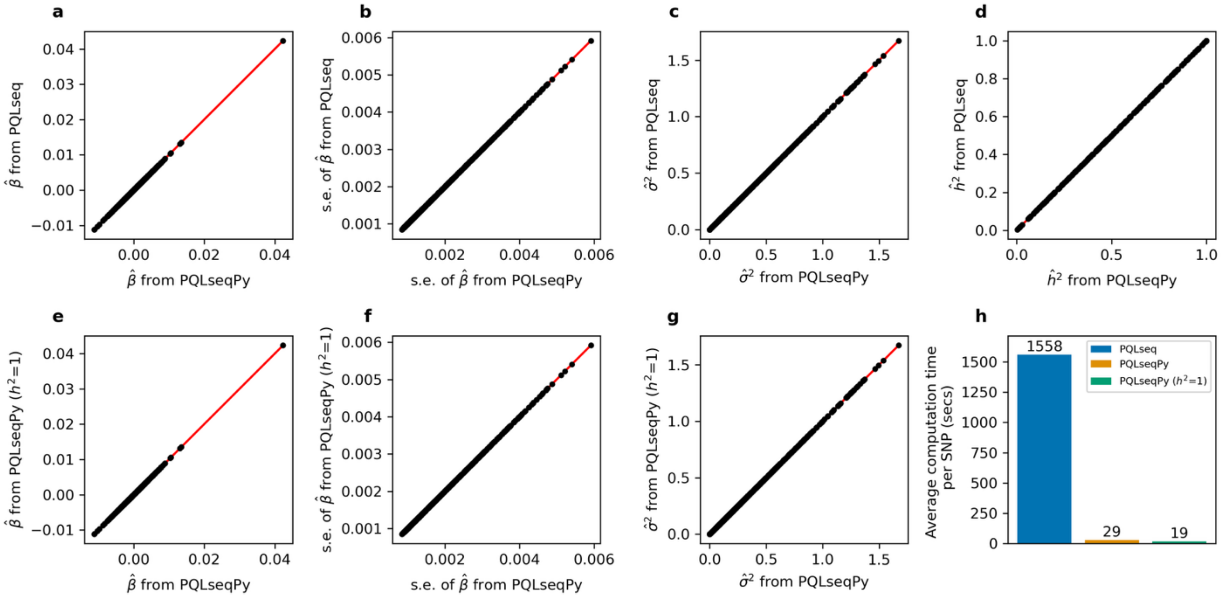

Figure S8.1: Performance of a faster re-implementation of PQLseq. Methods compared include the original implementation of PQLseq, the faster re-implementation PQLseqPy, and a modified version of PQLseqPy with the heritability parameter fixed at 1. (**a-g**) Scatter plots compare (**a,e**) estimates of β, (**b,f**) standard errors of β estimates, (**c,g**) estimates of the total variance component σ^2, and (d) estimates of the heritability parameter h^2. (**h**) Average computational time in seconds. Average memory usage was 694 MB for PQLseq and 771 MB for PQLseqPy. For all panels except d, we used real ancient DNA data consisting of 1,000 randomly selected SNPs from 2,000 individuals. For panel d, we generated simulations with LibSize = 100, $\sigma^2 = 1$, and 100 individuals, using a GRM for random ancient individuals as the covariance structure.

References

1. Marchini, J. & Howie, B. Genotype imputation for genome-wide association studies. *Nat. Rev. Genet.* **11**, 499–511 (2010).
2. Fu, Q. *et al.* A revised timescale for human evolution based on ancient mitochondrial genomes. *Curr. Biol. CB* **23**, 553–559 (2013).
3. Korneliussen, T. S., Albrechtsen, A. & Nielsen, R. ANGSD: Analysis of Next Generation Sequencing Data. *BMC Bioinformatics* **15**, 356 (2014).
4. Huang, Y. & Ringbauer, H. hapCon: estimating contamination of ancient genomes by copying from reference haplotypes. *Bioinforma. Oxf. Engl.* **38**, 3768–3777 (2022).
5. Manichaikul, A. *et al.* Robust relationship inference in genome-wide association studies. *Bioinforma. Oxf. Engl.* **26**, 2867–2873 (2010).
6. Haller, B. C. & Messer, P. W. SLiM 4: Multispecies Eco-Evolutionary Modeling. *Am. Nat.* **201**, E127–E139 (2023).
7. Irving-Pease, E. K. *et al.* The selection landscape and genetic legacy of ancient Eurasians. *Nature* **625**, 312–320 (2024).
8. Hayward, L. K. & Sella, G. Polygenic adaptation after a sudden change in environment. *eLife* **11**, e66697 (2022).
9. Buffalo, V. & Coop, G. Estimating the genome-wide contribution of selection to temporal allele frequency change. *Proc. Natl. Acad. Sci. U. S. A.* **117**, 20672–20680 (2020).
10. Loh, P.-R. *et al.* Reference-based phasing using the Haplotype Reference Consortium panel. *Nat. Genet.* **48**, 1443–1448 (2016).
11. Mudge, J. M. *et al.* GENCODE 2025: reference gene annotation for human and mouse. *Nucleic Acids Res.* **53**, D966–D975 (2025).

12. Hernandez, R. D. *et al.* Classic selective sweeps were rare in recent human evolution. *Science* **331**, 920–924 (2011).
13. Gazal, S. *et al.* Linkage disequilibrium-dependent architecture of human complex traits shows action of negative selection. *Nat. Genet.* **49**, 1421–1427 (2017).
14. Murphy, D. A., Elyashiv, E., Amster, G. & Sella, G. Broad-scale variation in human genetic diversity levels is predicted by purifying selection on coding and non-coding elements. *eLife* **12**, e76065 (2023).
15. Hermisson, J. & Pennings, P. S. Soft sweeps: molecular population genetics of adaptation from standing genetic variation. *Genetics* **169**, 2335–2352 (2005).
16. Koch, E. *et al.* Genetic association data are broadly consistent with stabilizing selection shaping human common diseases and traits. 2024.06.19.599789 Preprint at https://doi.org/10.1101/2024.06.19.599789 (2024).
17. Eyre-Walker, A. Genetic architecture of a complex trait and its implications for fitness and genome-wide association studies. *Proc. Natl. Acad. Sci.* **107**, 1752–1756 (2010).
18. Pritchard, J. K., Pickrell, J. K. & Coop, G. The genetics of human adaptation: hard sweeps, soft sweeps, and polygenic adaptation. *Curr. Biol. CB* **20**, R208-215 (2010).
19. Messer, P. W. & Petrov, D. A. Population genomics of rapid adaptation by soft selective sweeps. *Trends Ecol. Evol.* **28**, 659–669 (2013).
20. Boyko, A. R. *et al.* Assessing the evolutionary impact of amino acid mutations in the human genome. *PLoS Genet.* **4**, e1000083 (2008).
21. Zeng, J. *et al.* Widespread signatures of natural selection across human complex traits and functional genomic categories. *Nat. Commun.* **12**, 1164 (2021).

22. Mancuso, N. *et al.* The contribution of rare variation to prostate cancer heritability. *Nat. Genet.* **48**, 30–35 (2016).

23. Schoech, A. P. *et al.* Quantification of frequency-dependent genetic architectures in 25 UK Biobank traits reveals action of negative selection. *Nat. Commun.* **10**, 790 (2019).

24. Simon, A. & Coop, G. The contribution of gene flow, selection, and genetic drift to five thousand years of human allele frequency change. *bioRxiv* 2023.07.11.548607 (2024) doi:10.1101/2023.07.11.548607.

25. Wilson, B. A., Petrov, D. A. & Messer, P. W. Soft selective sweeps in complex demographic scenarios. *Genetics* **198**, 669–684 (2014).

26. Privé, F., Arbel, J. & Vilhjálmsson, B. J. LDpred2: better, faster, stronger. *Bioinforma. Oxf. Engl.* **36**, 5424–5431 (2021).

27. Nei, M. Analysis of gene diversity in subdivided populations. *Proc. Natl. Acad. Sci. U. S. A.* **70**, 3321–3323 (1973).

28. Yang, J., Zaitlen, N. A., Goddard, M. E., Visscher, P. M. & Price, A. L. Advantages and pitfalls in the application of mixed-model association methods. *Nat. Genet.* **46**, 100–106 (2014).

29. Nakagawa, S. & Schielzeth, H. A general and simple method for obtaining R2 from generalized linear mixed-effects models. *Methods Ecol. Evol.* **4**, 133–142 (2013).

30. Sousa da Mota, B. *et al.* Imputation of ancient human genomes. *Nat. Commun.* **14**, 3660 (2023).

31. Tajima, F. Statistical method for testing the neutral mutation hypothesis by DNA polymorphism. *Genetics* **123**, 585–595 (1989).

32. Braverman, J. M., Hudson, R. R., Kaplan, N. L., Langley, C. H. & Stephan, W. The hitchhiking effect on the site frequency spectrum of DNA polymorphisms. *Genetics* **140**, 783–796 (1995).

33. Bulik-Sullivan, B. K. *et al.* LD Score regression distinguishes confounding from polygenicity in genome-wide association studies. *Nat. Genet.* **47**, 291–295 (2015).

34. Purcell, S. *et al.* PLINK: a tool set for whole-genome association and population-based linkage analyses. *Am. J. Hum. Genet.* **81**, 559–575 (2007).

35. Zhou, X. & Stephens, M. Genome-wide efficient mixed-model analysis for association studies. *Nat. Genet.* **44**, 821–824 (2012).

36. Ronen, R. *et al.* Predicting Carriers of Ongoing Selective Sweeps without Knowledge of the Favored Allele. *PLoS Genet.* **11**, e1005527 (2015).

37. Akbari, A. *et al.* Identifying the Favored Mutation in a Positive Selective Sweep. *Nat. Methods* **15**, 279–282 (2018).

38. Fu, Y. X. Statistical properties of segregating sites. *Theor. Popul. Biol.* **48**, 172–197 (1995).

39. Mathieson, I. *et al.* Genome-wide patterns of selection in 230 ancient Eurasians. *Nature* **528**, 499–503 (2015).

40. Le, M. K. *et al.* 1,000 ancient genomes uncover 10,000 years of natural selection in Europe. *BioRxiv Prepr. Serv. Biol.* 2022.08.24.505188 (2022) doi:10.1101/2022.08.24.505188.

41. Kerner, G. *et al.* Genetic adaptation to pathogens and increased risk of inflammatory disorders in post-Neolithic Europe. *Cell Genomics* **3**, 100248 (2023).

42. Field, Y. *et al.* Detection of human adaptation during the past 2000 years. *Science* **354**, 760–764 (2016).

43. Byrska-Bishop, M. *et al.* High-coverage whole-genome sequencing of the expanded 1000 Genomes Project cohort including 602 trios. *Cell* **185**, 3426-3440.e19 (2022).

44. Zhao, H. *et al.* CrossMap: a versatile tool for coordinate conversion between genome assemblies. *Bioinforma. Oxf. Engl.* **30**, 1006–1007 (2014).

45. Karczewski, K. J. *et al.* The mutational constraint spectrum quantified from variation in 141,456 humans. *Nature* **581**, 434–443 (2020).

46. Berg, J. J. *et al.* Reduced signal for polygenic adaptation of height in UK Biobank. *eLife* **8**, e39725 (2019).

47. Sohail, M. *et al.* Polygenic adaptation on height is overestimated due to uncorrected stratification in genome-wide association studies. *eLife* **8**, e39702 (2019).

48. Stern, A. J., Wilton, P. R. & Nielsen, R. An approximate full-likelihood method for inferring selection and allele frequency trajectories from DNA sequence data. *PLoS Genet.* **15**, e1008384 (2019).

49. Vaughn, A. H. & Nielsen, R. Fast and Accurate Estimation of Selection Coefficients and Allele Histories from Ancient and Modern DNA. *Mol. Biol. Evol.* **41**, msae156 (2024).

50. Barrie, W. *et al.* Elevated genetic risk for multiple sclerosis emerged in steppe pastoralist populations. *Nature* **625**, 321–328 (2024).

51. Haak, W. *et al.* Massive migration from the steppe was a source for Indo-European languages in Europe. *Nature* **522**, 207–211 (2015).

52. Harney, É., Patterson, N., Reich, D. & Wakeley, J. Assessing the performance of qpAdm: a statistical tool for studying population admixture. *Genetics* **217**, iyaa045 (2021).

53. Fernandes, D. M. *et al.* The Spread of Steppe and Iranian Related Ancestry in the Islands of the Western Mediterranean. *Nat. Ecol. Evol.* **4**, 334–345 (2020).

54. Patterson, N. *et al.* Large-Scale Migration into Britain During the Middle to Late Bronze Age. *Nature* **601**, 588–594 (2022).

55. Sun, S. *et al.* Heritability estimation and differential analysis of count data with generalized linear mixed models in genomic sequencing studies. *Bioinforma. Oxf. Engl.* **35**, 487–496 (2019).
